# Quorum Sensing Coordinates Carbon and Nitrogen Metabolism to Optimize Public Goods Production in *Pseudomonas fluorescens* 2P24

**DOI:** 10.1002/advs.202412224

**Published:** 2025-01-31

**Authors:** Jie Li, Mengxue Nie, Hongguang Ma, Xuanying Tao, Yanxia Sun, Xinyue Tu, Pingping Zhang, Li‐Qun Zhang, Rong Jia, Yong‐Xing He, Nannan Zhang, Honghua Ge

**Affiliations:** ^1^ School of Life Sciences Anhui University Hefei 230601 China; ^2^ Institute of Health Sciences and Technology Institutes of Physical Science and Information Technology Anhui University Hefei 230601 China; ^3^ College of Plant Protection China Agricultural University Beijing 100083 China; ^4^ Ministry of Education Key Laboratory of Cell Activities and Stress Adaptations School of Life Sciences Lanzhou University Lanzhou 730000 China; ^5^ School of Veterinary Medicine and Biosecurity Lanzhou University Lanzhou 730000 China

**Keywords:** carbon and nitrogen metabolism, gene transcription, public goods, quorum sensing, transcription factor

## Abstract

The coordination of public and private goods production is essential for bacterial adaptation to environmental changes. Quorum sensing (QS) regulates this balance by mediating the trade‐off between the communal benefits of “public goods,” such as siderophores and antibiotics, and the individual metabolic needs fulfilled by “private goods,” such as intracellular metabolites utilized for growth and survival. *Pseudomonas fluorescens* 2P24 harbors a LasI/LasR‐type QS system, MupI/MupR, which regulates mupirocin production through signaling molecules. This study explores how QS coordinates carbon and nitrogen metabolism to optimize the production of key secondary metabolites, including 2,4‐diacetylphloroglucinol (2,4‐DAPG), mupirocin, and siderophores, which serve as public goods. Loss of QS disrupts this balance by enhancing the Krebs cycle, denitrification, pyruvate anaplerosis, and ammonium assimilation, lead to halted 2,4‐DAPG and mupirocin synthesis and increased siderophore production. In the absence of QS, elevated siderophore production compensates for iron acquisition, ensuring rapid cellular growth. Under nutrient‐limited or high cell density conditions, MupR regulates carbon and nitrogen fluxes to sustain public goods production. These findings highlight QS as a key environmental sensor that fine‐tunes resource allocation, bacterial fitness, and adaptation to ecological and nutritional conditions, suggesting the potential for QS‐targeted approaches to enhance antibiotic production and agricultural sustainability.

## Graphical abstract

*Pseudomonas fluorescens* 2P24 utilizes a LasI/LasR‐type quorum sensing (QS) system, MupI/MupR, to regulate carbon and nitrogen metabolism for optimal production of secondary metabolites as public goods, including 2,4‐diacetylphloroglucinol, mupirocin, and siderophores. QS acts as a sensor, regulating metabolic fluxes to maintain energy and nutrient homeostasis, enabling facultative cooperation, and supporting long‐term adaptation to environmental changes.

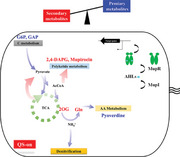

## 1 Introduction

Quorum sensing (QS) is a pivotal mechanism of bacterial cell‐to‐cell communication, relying on bacterial population density and coordination of social behavior.^[^ ^1^ ^]^ It involves the production of extracellular signaling molecules called autoinducers, which accumulate as cell‐population density increases.^[^ ^2^ ^]^ Bacteria use the QS system to regulate the expression of genes associated with the production of public goods, orchestrating a range of microbial behaviors such as bioluminescence, biofilm formation, mobility, virulence factor production, antibiotic synthesis, siderophore production, and diverse protease secretion.^[^ ^3^, ^4^ ^]^ However, producing these public goods imposes a metabolic burden on microbial communities, affecting the balance between communal benefits and individual costs in sophisticated environments.^[^ ^5^, ^6^ ^]^ Strains that do not produce public goods, often referred to as “cheaters,” tend to produce private goods, benefiting from the public goods produced by others without bearing the cost of production.^[^ ^7^ ^]^ Cheaters can be controlled by cooperator cells through various policing mechanisms, yet in some cases, nonproducing strains are not penalized and continue to participate in community cooperation.^[^ ^8^, ^9^ ^]^ This behavior is elucidated by the Black Queen Hypothesis, which provides a cost‐benefit selection framework suggesting that the loss of costly functional genes can improve growth efficiency within a microbial community.^[^ ^10^, ^11^, ^12^ ^]^

The function of QS and its role in enhancing cooperative behavior remains a topic of debate. While QS is conventionally perceived as a cell density‐dependent mechanism that facilitates the production and sharing of public goods among bacterial communities, recent studies posit QS as a sensor for individual bacteria cells to monitor signal levels and adapt to their physical environment.^[^ ^13^, ^14^ ^]^ In this context, individual cells produce and monitor signal levels to infer their local physical constraints. In crowded conditions, QS may help maintain homeostatic primary metabolism, balancing the production of public goods and private goods and addressing trade‐offs inherent in microbial communities. The acyl‐homoserine lactone (AHL) system has been extensively studied in the QS system from Gram‐negative bacteria.^[^ ^15^, ^16^ ^]^ It involves *LuxI*‐type enzymes that synthesize autoinducers like AHLs by catalyzing the transfer of an acyl group, bound to acyl carrier protein (ACP) from fatty acid biosynthesis, to S‐adenosylmethionine, enabling the formation of AHLs that interact with LuxR‐type transcriptional regulators to modulate QS target genes.^[^ ^17^ ^]^ AHLs are small lipid‐based signaling molecules in the QS systems of Gram‐negative bacteria, consisting of a homoserine lactone ring covalently linked to an acyl side chain, with the acyl group typically ranging from C4 to C18 carbons, either saturated or unsaturated, and these structural variations influence the specificity of AHLs for their corresponding LuxR‐type receptors, thereby affecting the sensitivity and functionality of the QS signaling cascade.^[^ ^16^ ^]^ AHLs are closely intertwined with the metabolism of bacterial substances, influencing both anabolism and catabolism. This metabolic interplay, primarily involving carbon, nitrogen, and sulfur metabolism, enables bacteria to rapidly adapt to harsh environments, such as nutrient scarcity and extreme survival conditions.^[^ ^18^ ^]^

For instance, in the rice pathogen *Burkholderia glumae*, the QS system TofI/R activates the expression of the transcription factor QsmR to downregulate *pstI* expression and slow glucose uptake. Simultaneously, the QsmR from *B. glumae* negatively regulates pathways, such as glycolysis, amino acids and nucleic acids biosynthesis, energy metabolism, and nitrogen metabolism. In addition, the QsmR upregulated the Krebs cycle in *B. glumae*.^[^ ^19^, ^20^ ^]^ Similarly, *Yersinia pestis* expresses a typical QS system for downregulating the glucose and nucleic acid metabolism while upregulating the Krebs cycle.^[^ ^21^ ^]^ The opportunistic human pathogen *Pseudomonas aeruginosa* uses LasI/R and RhlI/R to positively regulate the Krebs cycle, amino acids and nucleic acid metabolism, and negatively regulate denitrification.^[^ ^22^, ^23^ ^]^ These observations highlight that cell density, often linked to nutrient‐limited conditions, plays a pivotal role in QS‐regulated metabolic adjustments. The role of QS in regulating metabolic responses under environmental stress is indeed complex and how it intricately coordinates primary and secondary metabolism pathways to ensure survival in various sophisticated growth conditions remains uncertain. The heterogeneity in QS among bacteria like *Pseudomonas*, *Vibrio*, and *Xanthomonas* emphasizes a “division of labor” that allows for performing distinct tasks and shared resource utilization, ultimately benefiting overall community fitness.^[^ ^12^, ^24^, ^25^ ^]^ Still, questions persist about how QS manages homeostatic metabolism to balance the trade‐offs between primary metabolism and secondary metabolism, adapting to nutritional dynamics within microbial communities.

The study of QS‐mediated coordination of bacterial metabolism and social behaviors is important for understanding how this cooperative communication system adapts to enhance fitness. *Pseudomonas fluorescens* 2P24, isolated from the wheat rhizosphere, produces various secondary metabolites, including 2,4‐diacetylphloroglucinol (2,4‐DAPG), known for its potent inhibitory effects against a broad spectrum of soil‐borne pathogens.^[^ ^26^ ^]^ In addition to 2,4‐DAPG, the genome of *P. fluorescens* 2P24 also contains gene clusters for mupirocin, siderophore pyoverdine, and hydrogen cyanide biosynthetic gene clusters, highlighting the significant role of these secondary metabolites in determining bacterial fitness. Notably, *P. fluorescens* 2P24 harbors a LasI/LasR acyl homoserine lactone‐dependent QS system within the mupirocin biosynthesis gene cluster, designated as MupI/MupR. In *P. fluorescens* NCIMB 10586, the LasI homolog MupI synthesizes a signaling molecule that activates MupR, modulating the expression of genes responsible for mupirocin biosynthesis, thereby adding an additional layer of complexity to bacterial communication and regulation of secondary metabolite production.^[^ ^27^ ^]^ Here, we examined the role of QS‐mediated regulation of secondary metabolites production in *P. fluorescens* 2P24 by modulating the metabolic capabilities of microbial communities.

By integrating the transcriptome, metabolome, and ChIP‐seq analysis, we investigated impact of QS on the metabolic flux and gene transcription involved in secondary metabolite production in *P. fluorescens* 2P24. The loss of the QS function drastically alters the expression of genes involved in the Krebs cycle, fatty acid, amino acid metabolism, and denitrification, decreases the key Krebs cycle intermediate 2‐oxoglutarate (2‐OG), completely inhibit the production of carbon‐based secondary metabolites like AHL, 2,4‐DAPG, and mupirocin, while increase nitrogen‐based pyoverdine production. In conditions of high cell density, MupR acts as a metabolic brake, restricting the Krebs cycle and adjusting metabolic flux from carbon to nitrogen metabolism, resulting in higher level of 2‐OG compared to the loss of QS function mutant, which indicate QS as environmental sensor for orchestrating the balance between the primary metabolism and secondary metabolism. In the absence of QS control, increased pyoverdine production serves as a complementary system for bacterial growth, ensuring sufficient iron acquisition which is vital for cellular processes and overall fitness. The ecological and nutritional environment profoundly influences the spectrum of secondary metabolites produced by bacteria, with QS regulation markedly enhancing this diversity. By understanding how MupR‐mediated metabolic regulation governs the production of these metabolites, this study will bridge fundamental QS function with its practical applications, highlighting the potential of QS‐targeted approaches to optimize microbial metabolism for antibiotic production or promote sustainability in agricultural ecosystems.

## 2 Results

### 2.1 QS Positively Regulated Polyketide 2,4‐DAPG and Mupirocin and Negatively Regulated Pyoverdine

**Figure 1:**
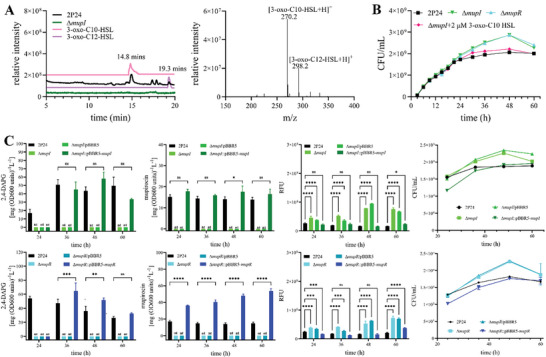
The regulation of cell growth and public goods production of *P. fluorescens* 2P24 by QS. A) HPLC and LC‐MS analysis of AHLs produced by *P. fluorescens* 2P24. HPLC represents the samples of an extract prepared from the culture medium of *P. fluorescens* 2P24, Δ*mupI* mutant strain, the standard chemicals of 3‐oxo‐C10‐HSL and 3‐oxo‐C12‐HSL (left). Further analysis of MS/MS spectra confirmed the identity of 3‐oxo‐C10‐HSL with *m*/*z* 270.2 and 3‐oxo‐C12‐HSL with *m*/*z* 298.2 extracted from the culture medium of *P. fluorescens* 2P24 (right). B) Difference of growth curves (colony‐forming units [CFU] per mL) between *P. fluorescens* 2P24 and the QS null mutants. CFU were determined by performing serial dilutions for plating on LB agar. The QS null mutants grew more rapidly at the late exponential and the early stationary phase than did the wild type. The initial cell turbidity was 0.1 at 600 nm. C) Quantitative analysis of 2,4‐DAPG, mupirocin, and pyoverdine production, the cell growth of Δ*mupI* (top panel) and Δ*mupR* (bottom panel) mutants compared with wide type *P. fluorescens* 2P24. Error bars denote standard deviations (*n* = 3). Statistical analyses were performed using the *t*‐test and two‐way ANOVA, *p* < 0.0001 is displayed as ****p < 0.001 is displayed as ***p < 0.01 is displayed as **p < 0.05 is displayed as *.

To assess the impact of QS on the metabolism of *P. fluorescens* 2P24, we monitored the growth of the wild‐type 2P24, the Δ*mupR*, and the Δ*mupI* mutant strains in KB medium. The molecular basis of QS was further explored using Foldseek^[^ ^28^ ^]^ to identify the homologs of MupI in the Protein Data Bank (PDB).^[^ ^29^ ^]^ The analysis revealed the highest similarity to LasI from *P. aeruginosa* PAO1, with a sequence identity of 55.2% and a root mean square deviation (RMSD) of 2.58 Å, indicating substantial structural conservation. LasI from *P. aeruginosa* is involved in the constitutive production of the QS signaling molecule 3‐oxo‐C12‐HSL, which activates the LasR receptor and regulates QS‐dependent genes in *P. aeruginosa*.^[^ ^30^ ^]^ This structural similarity suggests that MupI play an analogous role in *P. fluorescens* 2P24. LC‐MS analysis confirmed that MupI predominantly synthesizes 3‐oxo‐C10‐HSL, with lesser amounts of 3‐oxo‐C12‐HSL also present (**Figure** **1**A). Interestingly, the QS null mutants, the mutant Δ*mupR* and the mutant Δ*mupI*, exhibited accelerated growth during the late exponential phase compared to the wild type strain (WT) (Figure 1B). Supplementation of 2 × 10^−6^ m of exogenous 3‐oxo‐C10‐HSL restored the growth rate of the mutant Δ*mupI* strain to that WT level, suggesting that QS regulates the growth restraint during stationary phases, particularly under nutrient scarcity.

2,4‐DAPG has been previously characterized as a key secondary metabolite produced by *P. fluorescens* 2P24.^[^ ^26^ ^]^ Further genome analysis using antiSMASH^[^ ^31^ ^]^ identified additional biosynthetic gene clusters for mupirocin and pyoverdine, highlighting the metabolic versatility of this strain. Notably, mupirocin biosynthetic genes share 85% similarity with those in *P. fluorescens* NCIMB 10586, while pyoverdine genes exhibit 50% similarity to those in *P. protegens* Pf‐5. Metabolite quantification using LC‐MS and HPLC confirmed mupirocin synthesis (Figure S1A, Supporting Information), while pyoverdine was characterized by intrinsic fluorescence and the Chrome Azurol S (CAS) assay for siderophore activity (Figure S1B, Supporting Information).

We measured these public goods production in *P. fluorescens* 2P24, QS‐deficient strains Δ*mupR* and Δ*mupI*, and their complemented counterparts, Δ*mupR*::pBBR5‐*mupR* and Δ*mupI*::pBBR5‐*mupI* (Figure 1C). In *P. fluorescens* 2P24, mupirocin maintains a steady concentration postsynthesis, whereas 2,4‐DAPG levels rise during early growth phases and decline at higher cell densities (Figure S2A,B, Supporting Information). However, the QS null mutants did not produce either mupirocin or 2,4‐DAPG, indicating a critical role of QS in its synthesis. Compared to the wild‐type strain, pyoverdine production was higher in the QS null mutants Δ*mupR* and Δ*mupI* (Figure S2C, Supporting Information). Complementation of the QS‐null mutants fully restored the production of all public goods, with Δ*mupR*::pBBR5‐*mupR* showing significantly enhanced mupirocin production compared to the wild type. Furthermore, the biofilm produced by the QS‐deficient strains Δ*mupR* and Δ*mupI* was significantly reduced compared to the wild‐type (Figure S3, Supporting Information), highlighting the essential role of MupI/MupR QS system in regulating the production of public goods.

Mupirocin and 2,4‐DAPG, polyketide antibiotics produced by *P. fluorescens* 2P24, are synthesized through polyketide biosynthesis pathways involving the iterative condensation of carboxylic acid building blocks like acetyl‐CoA or malonyl‐CoA, catalyzed by polyketide synthase (PKS) enzymes.^[^ ^32^, ^33^, ^34^ ^]^ The biosynthesis of pyoverdine, a siderophore crucial for iron acquisition and microbial competition, is mediated by non‐ribosomal peptide synthetases (NRPSs).^[^ ^35^ ^]^ NRPSs are large multifunctional enzymes that assemble peptide chains using amino acid precursors.^[^ ^36^ ^]^ During pyoverdine biosynthesis, carbon resources are directed toward essential amino acid precursors, such as l‐lysine, d‐aspartic acid, and l‐threonine, to ensure the availability of building blocks for siderophore synthesis.^[^ ^37^ ^]^ The biosynthesis of polyketide antibiotics like mupirocin and 2,4‐DAPG diverts acyl‐CoA fluxes away from central carbon metabolism, while pyoverdine production reallocates carbon toward essential amino acid precursors.

In the absence of QS regulation, the overproduction of pyoverdine in QS‐deficient mutants may compensate for the lack of mupirocin and 2,4‐DAPG production, providing a competitive advantage in iron‐limited environments. However, this increased pyoverdine synthesis may disrupt the cooperative behaviors typically mediated by QS, such as the collective production of other public goods like polyketide antibiotics. These shifts may affect the overall viability and fitness of bacterial communities, with potential trade‐offs arising from the metabolic cost of excessive siderophore production. The metabolic costs of producing large amounts of pyoverdine could reduce the overall fitness of QS‐deficient mutants, underscoring the delicate balance between resource allocation and the benefits of public goods production. This indicates that QS in *P. fluorescens* 2P24 could prioritize specific metabolic pathways, regulating microbial energy metabolism to balance growth with the production of public goods critical for community fitness and survival.

### 2.2 MupR Is a Transcriptional Regulator for the Coordination of Primary Carbon and Nitrogen Metabolism

To elucidate the regulatory mechanisms mediated by MupR in *P. fluorescens* 2P24, we used ChIP‐seq analysis combined with promoter fusion reporter systems and RT‐qPCR. Results from four independent ChIP‐seq experiments and KEGG enrichment analysis are summarized in Table S3 and Figure S4 (Supporting Information). Validation of these targets was achieved through EGFP‐based promoter fusion assays and RT‐qPCR. We discovered that MupR binds to the promoters of the gene clusters responsible for mupirocin and 2,4‐DAPG biosynthesis, but not to those of the pyoverdine cluster. This indicates that MupR may directly regulate the gene transcription associated with mupirocin and 2,4‐DAPG production. Combined with ChIP‐seq analysis and operon‐mapper prediction, we evaluated the transcriptional units of the mupirocin and 2,4‐DAPG biosynthetic clusters and genes of the relative fluorescence of plasmid‐borne EGFP reporter fusions in 2P24 and Δ*mupR* strains. MupR positively regulates key functions involved in the biosynthesis of antibiotics 2,4‐DAPG (Figure S5A, Supporting Information) and mupirocin (Figure S5B, Supporting Information). Within the *mup* gene cluster, seven promoters exhibited significantly reduced expression and one showed slightly decreased expression in the mupR mutant compared to the wide type 2P24 strain, indicating activation by MupR. In addition, RT‐qPCR analysis confirmed the downregulation of these eight target genes). Within the *phl* cluster, differential expression patterns were observed, *phlF* promoter was upregulated, *phlA* was significantly downregulated, and *phlD* showed no change in the Δ*mupR* mutant compared to wild‐type 2P24, suggesting the role of MupR in both repression and activation of transcription within this cluster. However, upon further RT‐qPCR analysis, all four genes were downregulated.

**Figure 2:**
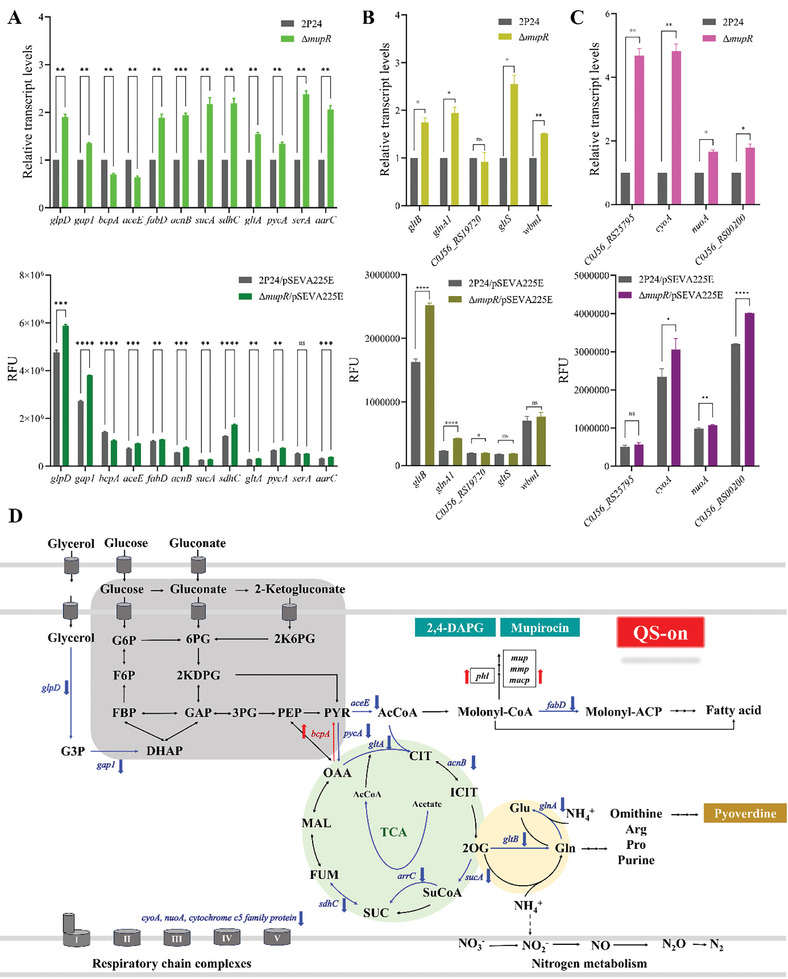
The transcription factor MupR plays a key role in regulating carbon metabolism, amino acid metabolism, and the respiratory chain in *P. fluorescens* 2P24. A) Relative expression level of *glpD*, *gap1*, *bcpA, aceE, fadD, acnB, sucA, sdhC, gltA, pycA, sera*, and *aarC* as ChIP‐seq targets from carbon metabolism were quantified by RT‐PCR (top panel). The differentially expressed regulatory genes were evaluated in WT and Δ*mupR* strains, in which the reporter gene *egfp* was placed under the control of *glpD*, *gap1*, *bcpA, aceE, fadD, acnB, sucA, sdhC, gltA, pycA, serA*, and *aarC* promoters (bottom panel). B) Relative expression level of *gltB*, *glnA1*, *C0J56_RS19720, gltS*, and *wbmI* as ChIP‐seq targets from the amino acid metabolism were quantified by RT‐PCR (top panel). The differentially expressed regulatory genes were evaluated in WT and Δ*mupR* strains, in which the reporter gene *egfp* was placed under the control of *gltB*, *glnA1*, *C0J56_RS19720, gltS*, and *wbmI* promoters (bottom panel). C) Relative expression level of *C0J56_RS25795, cyoA*, *nuoA*, and *C0J56_RS00200* as ChIP‐seq targets from the respiratory chain were quantified by RT‐PCR (top panel). The differentially expressed regulatory genes were evaluated in WT and Δ*mupR* strains, in which the reporter gene *egfp* was placed under the control of *gltB*, *glnA1*, *C0J56_RS19720, gltS*, and *wbmI* promoters (bottom panel). The fluorescence activities were measured, and illustrated the relative fluorescent units (RFU) that normalized to a 1 ml culture with OD_600_ = 1. Error bars denote standard deviations (*n* = 3). Statistical analyses were performed using the *t*‐test and two‐way ANOVA, *p* < 0.0001 is displayed as ****p < 0.001 is displayed as ***p < 0.01 is displayed as **p < 0.05 is displayed as *. D) This schematic represents the influence of MupR on the genes, metabolites, and biological pathways in *P. fluorescens* 2P24, integrating data from ChIP‐seq analyses. It visually categorizes genes as activated (red) or repressed (blue) by QS, and arrows indicate the direction of metabolic flux influenced by MupR. This diagram also proposes a general model for flux distribution in *P. fluorescens* 2P24.

Further analysis of ChIP‐seq data focused on key enzymes from TCA cycle, revealing extensive regulation by MupR (**Figure** **2**A). MupR negatively regulates key functions involved in the TCA cycle. MupR directly binds to the promoters of several critical enzymes including citrate synthase (*gltA*), pyruvate carboxylase subunit A (*pycA*), aconitate hydratase B (*acnB*), 2‐oxoglutarate dehydrogenase subunit E1 (*sucA*), and succinate dehydrogenase b556 subunit (*sdhC*), leading to increased transcriptional levels in the Δ*mupR* strain and suggesting that QS can slow down the TCA cycle. Conversely, MupR upregulates oxaloacetate decarboxylase (*bcpA*), a carboxy‐lyase that converts oxaloacetate into pyruvate,^[^ ^38^ ^]^ indicating a shift in metabolic fluxes and potentially altering energy production pathways. MupR repressed genes involved in glycerol metabolism, specifically glycerol‐3‐phosphate dehydrogenase (*glpD*) and glyceraldehyde‐3‐phosphate dehydrogenase (*gap1*). In addition, it negatively regulates succinyl‐CoA: CoA‐transferase (*aarC*), which plays a role in detoxifying acetate by converting it into acetyl‐CoA and succinate,^[^ ^39^ ^]^ further illustrating QS involved in regulating both primary and secondary metabolic pathways. Interestingly, there are pyruvate metabolisms subject to negative regulation by MupR. MupR binds to the promoter of pyruvate carboxylase (*pycA*) and pyruvate dehydrogenase E1 component (*aceE*), repressing its transcription, which is crucial for converting pyruvate to oxaloacetate and acyl‐CoA respectively,^[^ ^40^, ^41^ ^]^ an anaplerotic reaction replenishing TCA cycle intermediates. The malonyl CoA‐acyl carrier protein transacylase (*fabD*) that is responsible for malonyl‐CoA precursor flux to fatty acid biosynthesis,^[^ ^42^ ^]^ is repressed and limited by the QS system. The glutamine synthetase (GS)/glutamate synthase (GOGAT) cycle is critical for nitrogen assimilation,^[^ ^43^ ^]^ the expression of glutamine synthetase (*glnA1*) and glutamate synthase large subunit (*gltB*) was repressed by MupR, indicating a QS regulation of nitrogen assimilation (Figure 2B). This repression of gene transcription related to nitrogen assimilation may decrease the availability of amino acid precursors, potentially leading to a reduction in pyoverdine production. MupR downregulated the transcript levels of respiration chain genes *cyoA*, *nuoA*, cytochrome c5 family protein (Figure 2C), further suggests a metabolic adjustment toward enhanced respiratory chain.

MupR repressed the genes involved in TCA cycle and nitrogen assimilation while simultaneously promoting the synthesis of key secondary metabolites like 2,4‐DAPG and mupirocin. In contrast, MupR regulation of gene involved in nitrogen assimilation and pyoverdine production is suppressed, likely to accommodate slower bacterial growth and reduced iron acquisition needs under nutrient‐limited conditions (Figure 2D). MupR regulates the transcription of genes related to the key carbon and nitrogen metabolisms, potentially controlling metabolic fluxes toward public goods production. It suggests that MupR is not merely a passive responder to stress but actively regulates metabolic pathways to maintain energy and nutrient homeostasis, playing a vital role in the bacterium's long‐term adaptation to dynamic environments.

### 2.3 Δ*mupR* Affects Gene Transcription, Extending Not Only to Public Goods but Also from Carbon Metabolism to Nitrogen Metabolism

**Figure 3:**
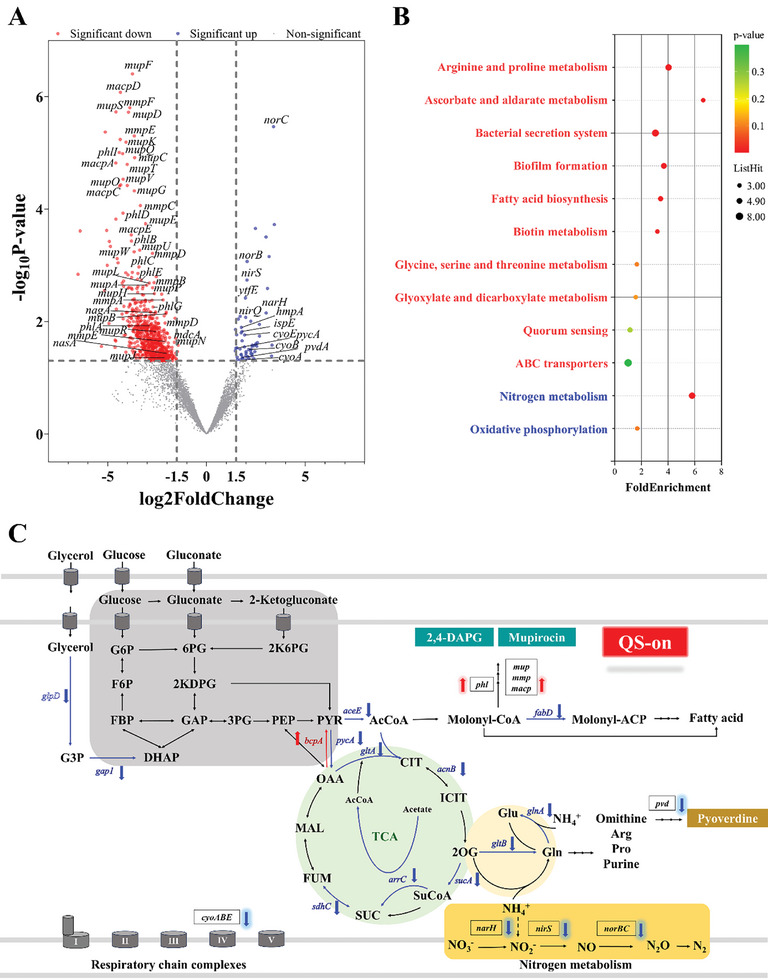
MupR from *P. fluorescens* 2P24 as a QS transcription factor controls the transcriptional regulation of genes related to public goods extending from carbon metabolism to nitrogen metabolism. A) The comparative transcriptomic analysis of the Δ*mupR* and WT strains. Volcano plot of DEGs with 664 downregulated genes (red dot) and 61 upregulated genes (blue dot) in the Δ*mupR* strain compared with WT strain. The gray dots refer to the genes with no significant expression change. B) GO enrichment analysis of the differentially expressed proteins in the Δ*mupR* strain. The enriched GO terms with *p* < 0.05 were displayed. C) Summary of MupR affecting transcription of genes and related biological pathways in *P. fluorescens* 2P24. DEGs were indicated in black borders. The red up arrows and the blue down arrows indicate MupR‐mediated upregulated and downregulated genes, respectively. Metabolites abbreviations are as follows: G6P, glucose‐6‐phosphate; F6P, fructose‐6‐phosphate; FBP, fructose‐1,6bisphosphate; DHAP, dihydroxyacetone phosphate; 6PG, 6‐phosphogluconate; 2KDPG, 2‐keto‐3deoxy‐6‐phosphogluconate; 2K6PG, 2‐keto‐6‐phosphogluconate; GAP, glyceraldehyde‐3‐phosphate; 3PG, 3‐phosphoglycerate; PEP, phosphoenolpyruvate; Pyr, pyruvate; AcCoA, acetyl‐coenzyme A; OAA, oxaloacetate; CIT, citrate; ICT, isocitrate; 2OG, 2‐ketoglutarate; SUC, succinate; SuCoA, succinate‐CoA; FUM, fumarate; MAL, malate. Gene abbreviations refer to the encoded enzymes, namel*y: phl*, 2,4‐DAPG biosynthetic genes; *mup*, mupirocin biosynthetic genes; *pvd*, pyoverdine biosynthetic gene. *cyoABE;* aerobic quinol oxidase bo3 subunits; *narH*, nitrate reductase; *nirQS*, nitrite reductases; and *norBC*, nitric oxide reductase.

Given the substantial impact of MupR on growth and public goods production at high cell densities in *P. fluorescens* 2P24, we aimed to explore its effects through global transcriptomic analysis. Three biological replicates of wild‐type 2P24 and Δ*mupR* were cultured to the early stationary phase. Total RNA was isolated and subjected to RNA‐seq, yielding 16.36–16.57 million reads per replicate. 41% of the genome was expressed in WT cells. Differential gene expression analysis was conducted for all transcripts that mapped onto the *P. fluorescens* 2P24 genome. From the refined data set of 5546 genes, 725 genes were differentially regulated in the Δ*mupR* strain (*p*‐value < 0.05, log_2_ fold change > |1.5|), corresponding to 13% of the genome. Specifically, 61 genes were upregulated and 664 genes were downregulated in the Δ*mupR* compared to WT, as detailed in **Figures** **3**A and S5 (Supporting Information). Notably, among the downregulated genes were 33 genes from the mupirocin biosynthetic gene cluster and 7 genes from the 2,4‐DAPG cluster. Conversely, five genes involved in pyoverdine biosynthesis were upregulated. Gene Ontology (GO) and Kyoto Encyclopedia of Genes and Genomes (KEGG) pathway enrichment analyses of downregulated genes in Δ*mupR* revealed significant enrichment in crucial biological pathway. Notably, the metabolism pathways associated with amino acid metabolism, carbohydrate metabolism, fatty acid biosynthesis, ABC transporters, and Type II and Type III secretion systems were prominently affected (Figure 3B and Table S5, Supporting Information).

Dihydroxy‐acid dehydratase plays a role in the biosynthesis of the branched‐chain amino acids valine, leucine, and isoleucine, as well as in the biosynthesis of pantothenate and coenzyme A (CoA),^[^ ^44^ ^]^ is downregulated in the Δ*mupR* mutant. The glycerate kinase, which converts glycerate to 2‐phospho‐glycerate in the nonphosphorylated Entner‐Doudoroff pathway,^[^ ^45^ ^]^ is notably downregulated in the Δ*mupR* mutant. In contrast, the transcription of pyruvate carboxylase, catalyzing the ATP‐dependent carboxylation of pyruvate to oxaloacetate thus enhancing the TCA cycle,^[^ ^46^ ^]^ was increased. In the Δ*mupR* mutant, there was a notable upregulation of genes involved in aerobic respiration and denitrification, indicating a significant metabolic shift (Figure 3B). This upregulation includes genes encoding the aerobic quinol oxidase bo3 subunits (*cyoABE*)^[^ ^47^ ^]^ and components of the denitrification pathway such as nitrate reductase‐related *narH*, nitrite reductases‐related *nirQS*, nitric oxide reductase (*norBC*), and a Crp/Fnr family transcriptional regulator.^[^ ^48^ ^]^ In addition, the upregulation of *ytfE* and *hmpA*, which encode proteins associated with the bacterial response to oxidative and nitrosative stress and play roles in nitric oxide detoxification under aerobic conditions,^[^ ^49^, ^50^ ^]^ further suggesting a metabolic adjustment toward enhanced respiratory chain in the Δ*mupR* mutant. The significant downregulation and upregulation of these genes involved in public goods production, carbon metabolism, and nitrogen metabolism in the Δ*mupR* mutant were verified by RT‐qPCR (Figure S6, Supporting Information).

The Δ*mupR* mutant enhances gene transcription related to aerobic respiration, likely increasing ATP production through oxidative phosphorylation, which provides more energy to support rapid cellular growth. Concurrently, the upregulation of denitrification pathways facilitates the conversion of nitrate to nitrogen gas, influencing the bacterial redox state by balancing electron flow and maintaining redox homeostasis of Δ*mupR* mutant. This process also affects nitrogen cycle‐related substances, such as nitrate and nitrite, which are reduced and processed within the cell. Together, these metabolic adjustments to energy production compensate for the increased energy demands required for rapid cellular growth in the absence of QS control, with the upregulated denitrification pathway playing a pivotal role in maintaining cellular homeostasis (Figure 3C).

Under QS control at high bacterial density, alterations of gene transcription in the TCA cycle that could slow energy production, along with shifts in carbohydrate and fatty acid metabolism, may redirect central carbon intermediates to enhance the synthesis of key carbon‐based secondary metabolites, such as 2,4‐DAPG and mupirocin. These metabolic adjustments, coupled with the upregulation of the denitrification pathway, reflect a re‐prioritization of cellular energy production and metabolic process. Simultaneously, the downregulation of amino acid assimilation pathways reduces the availability of essential precursors for the synthesis of key nitrogen‐based secondary metabolites, such as siderophore, further contributing to the rewiring of both carbon and nitrogen metabolism.

### 2.4 Quorum‐Sensing Regulator MupR Binding Site Is Required for Control of Gene Activation and Repression

**Figure 4:**
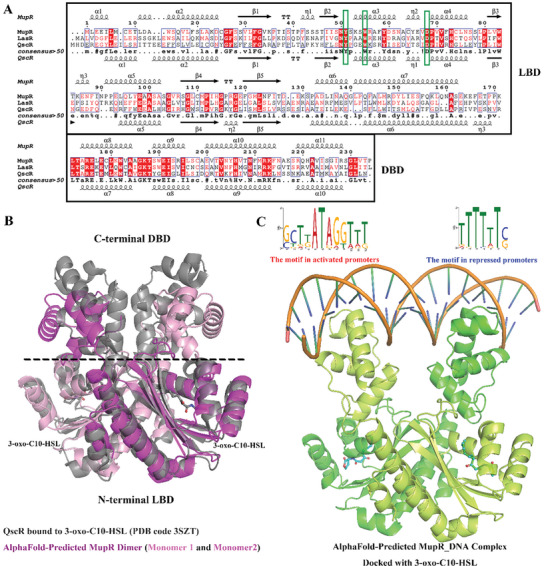
MupR is a member of the LuxR family of transcription factors. A) Sequence alignment of MupR with LuxR family members LasR and QscR from *P. aeruginosa*. Ligand‐binding domain (LBD) and DNA‐binding domain (DBD) are boxed. Residues involved in hydrogen‐bonding interactions with AHLs were shown in green boxes, showing the conserved key amino acids that mediate AHL binding across the LuxR family. B) The cartoon representation of AlphaFold3 predicted model of MupR dimer model. The two monomers are colored in purple and pink, respectively. The MupR superimposed on QscR in complex with 3‐oxo‐C10‐HSL (pdb code 3SZT), which is colored in gray. The orientation of the LBDs in MupR and QscR is similar. C) The model MupR_DNA complex with docked 3‐oxo‐C10‐HSL features monomers in green and lemon, with bound 2,4‐DAPG depicted in cyan. Top panel show alignments of DNA binding motifs for activated and repressed MupR targets promoters from ChIP‐seq data.

Similar to other LuxR‐family regulators, MupR comprises an N‐terminal ligand‐binding domain (LBD) and a C‐terminal DNA‐binding domain (DBD), exhibiting 41% and 29% sequence similarity with LasR and QscR from *P. aeruginosa*, respectively (**Figure** **4**A). Structural modeling of MupR, predicted using AlphaFold3,^[^ ^51^ ^]^ reveals a domain‐swapping homodimer configuration. The N‐terminal LBD facilitates dimerization and binds AHLs, while the C‐terminal DBD features a canonical helix‐turn‐helix motif essential for recognizing dyad symmetry in DNA. The LBD is structured as an α‐β‐α sandwich comprising beta strands β1–β5 and alpha helices α1–α5. MupR superimposes onto QscR of *P. aeruginosa* (pdb code 3SZT) with RMSD value of 4.358 Å, indicating structural similarity. Moreover, 3‐oxo‐C10‐HSL is accommodated within a binding cavity of MupR, similar to the interaction of 3‐oxo‐C10‐HSL observed in QscR (Figure 4B). A structural model of MupR dimer was docked with 3‐oxo‐C10‐HSL by AutoDock vina and the AHL binding site was compared with those in LasR and QscR from *P. aeruginosa* (Figure S7, Supporting Information). Within the N‐terminal LBD, the conserved residues Tyr51, Trp55, and Asp68, which are critical for AHL binding in LasR and QscR from *P. aeruginosa*, are also present in MupR. These residues form key interactions with the AHL molecule, underscoring the functional importance of this binding configuration in the QS mechanism.

This AHL‐binding and the subsequent DNA‐binding are crucial for regulatory role of MupR in gene expression, which is evidenced by validated binding sites on the promoters of target genes, leading to gene activation or repression. As summarized in Table S4 (Supporting Information), the distance between the binding sites and the transcriptional start sites (TSS) for each target gene is listed. MupR predominantly binds upstream of the TSS for most target genes (*mmpB*, *mupF*, *macpC*, *mupO*, *macpE*, *mupI*, *glpD*, *glnA1*, *gltB*, *acnB*, *fabD*, *aceE*, *gltA*, *aarC*, *nuoA*, *cyoA*). Interestingly, MupR bind around the ‐10 or ‐35 box of most target genes within the mupiroicn biosynthetic cluster (*mmpB*, *mupF*, *macpC*, *mupO*, *macpE*, and *mupI*), leading to significant activation of transcription for these genes. For the target genes associated with amino acid metabolism (*glnA1* and *gltB*), MupR bound to the upstream remote to the TSS, to repress the transcription of these genes. For the target genes related to carbon metabolism and respiratory chain, MupR exhibits more complex binding patterns. It binds upstream of the TSS for genes such as *glpD*, *acnB*, *fabD*, *aceE*, *gltA*, *aarC*, *nuoA*, and *cyoA*) or downstream of the TSS for genes such as *sdhC*, *sucA* and C0J56_RS00200, to repress the transciption of these genes. While for the target genes within the 2,4‐DAPG biosynthetic gene cluster (*phlA* and *phlF*), MupR binds downstream of the TSS, which activated *phlA* and repressed *phlF*, respectively. The validated binding sites of MupR at the promoters of genes were classified into two groups for gene activation and repression, respectively. Using AlphaFold3,^[^ ^51^ ^]^ a structural model of MupR_DNA complex was generated and then docked with 3‐oxo‐C10‐HSL by AutoDock vina. Analysis with the MEME (multiple EM for motif elicitation) algorithm revealed two types of binding motifs derived from upregulated and downregulated MupR ChIP‐seq targets (Figure 4C; Table S4 and Figure S8, Supporting Information). Deleting these core MupR binding motifs from target gene promoters altered their transcriptional activity. Assessment using plasmid‐borne EGFP reporter fusions in both wild‐type 2P24 and Δ*mupR* strains showed these modifications hindered activation and partially alleviated repression of EGFP expression, confirming the critical nature of these motifs in gene regulation (Figure S9, Supporting Information). This study fundamentally advances our understanding of the molecular mechanisms behind QS regulation in *P. fluorescens* 2P24, emphasizing the critical interaction between MupR and its DNA targets within the context of bacterial community.

### 2.5 MupR Elicits the Readjustment of Metabolic Fluxes, Rerouting Carbon Flux to Nitrogen Metabolic Pathways

To further elucidate the role of MupR in the metabolic network of *P. fluorescens* 2P24, key metabolites were quantified using LC‐MS/MS, comparing the metabolic profiles of the Δ*mupR* with its WT strain during the stationary phase. A total of 257 metabolites significantly differed in abundance between Δ*mupR* and WT (the *p*‐value < 0.05, variable importance in the projection (VIP) > 1 the metabolites are listed in Table S6 and Figure S10 (Supporting Information). The results confirmed a significant reduction in the levels of mupirocin and 2,4‐DAPG in the mutant Δ*mupR* strain, aligning with earlier HPLC analyses. Notably, pyoverdine could not be quantified due to its unidentified molecular structure.

**Figure 5:**
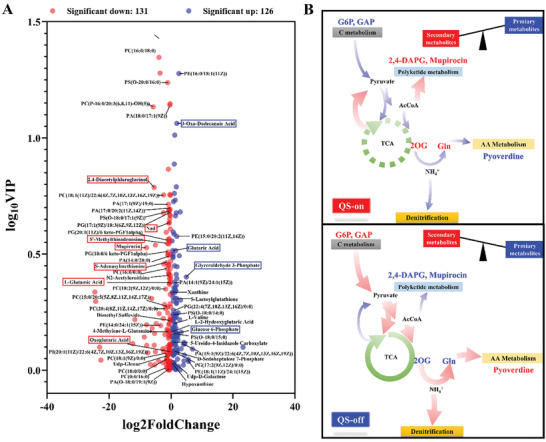
In vivo metabolic flux distribution in *P. fluorescens* 2P24 controlled by QS. A) Volcano plot of the metabolic profile with 131 downregulated metabolites (red dot) and 126 upregulated metabolites (blue dot) in the Δ*mupR* strain compared with WT strain. The horizontal axis represents log_2_fold change > |0| in metabolite content, and the vertical axis represents log_10_VIP > 0, the *p*‐value < 0.05. B) Schematic illustration show that QS moderates the tricarboxylic acid (TCA) cycle and restricts denitrification pathways, maintaining cellular energy homeostasis to balance the production of public goods and private goods in *P. fluorescens* 2P24. In addition, metabolites are color‐coded to show increases (red) or decreases (blue) in metabolic flux, depending on QS activity. This diagram also proposes a general model for flux distribution in *P. fluorescens* 2P24, detailing how QS modulation affects metabolic pathways.

The deletion of *mupR* was associated with increased levels of glucose‐6‐phosphate (G6P) and glycerol 3‐phosphate (G3P), crucial metabolites in the glycolysis, gluconeogenesis, and glycerophospholipid biosynthesis pathways (**Figure** **5**A). In addition, glutarate levels were elevated in the Δ*mupR* mutant, suggesting its catabolism through the glutarate hydroxylation and the glutaryl‐CoA dehydrogenation pathways, facilitating its entry into the TCA cycle. These changes indicate a potential redirection of carbon flux toward the glycolysis pathways and TCA cycle, underscoring a broader regulatory role for MupR beyond secondary metabolite synthesis. Conversely, metabolites central to nitrogen assimilation, such as 2‐oxoglutarate (2OG) and glutamate (Glu), were diminished in the mutant strain. This suggests a possible role for MupR in coordinating nitrogen metabolism, potentially through the regulation of ammonium assimilation. Moreover, nicotinamide adenine dinucleotide (NAD) levels were reduced in the Δ*mupR* mutant strain, impacting redox homeostasis and energy metabolism due to changes in the NADH/NAD ratio. This dysregulation highlights the multifaceted influence of MupR on bacterial metabolism. Phospholipid metabolism also appeared altered, with an increase in phosphatidylethanolamine (PE) and decreases in phosphatidylglycerol (PG), phosphatidylcholine (PC), phosphatidylserine (PS), and phosphatidic acid (PA), suggesting that *mupR* deletion affects membrane lipid composition. The increase in PE might be an adaptive response to maintain membrane integrity under altered metabolic conditions caused by the absence of MupR.

Collectively, these findings highlight the broad impact of the *mupR* deletion on metabolic pathways in *P. fluorescens* 2P24, affecting secondary metabolite production, carbon and nitrogen metabolism, phospholipid metabolism, and cellular energetics, which could significantly influence bacterial membrane composition and overall cellular physiology.

## 3 Conclusion 

Bacterial collective sensing, often mediated through QS, enables individual bacteria to produce and detect signaling molecules, thereby synchronizing its behavior across the community. QS systems differ among bacterial species, reflecting evolutionary adaptations to specific ecological niches. In *P. aeruginosa*, a complex QS network organized into multiple interconnected systems that utilize both AHLs and quinolone signals, with the Las system primarily controlling virulence factors via the LasR receptor, the Rhl system regulating biofilm formation and motility through the RhlR receptor, and the PQS system functioning independently to influence iron acquisition and antibiotic resistance.^[^ ^52^ ^]^ In contrast, *P. fluorescens* utilizes the simpler PcoI/PcoR system,^[^ ^53^ ^]^ primarily involved in biocontrol, while *B. glumae* employs the TofI/TofR system and additional regulatory genes like qsmR, expanding its QS network beyond AHL signaling to include virulence factors.^[^ ^54^ ^]^ The evolutionary origin and maintenance of QS are controversial. While some propose that QS evolved as a form of social communication to enhance collective behavior and fitness,^[^ ^2^, ^55^ ^]^ others suggest it serves as a sensor system that allows bacteria to monitor and respond to a complex array of environmental signals, thereby enabling adaptive responses that are critical for survival, competition, and cooperation within ecological niches.^[^ ^56^, ^57^, ^58^ ^]^

QS is fundamentally about sensing cell density, enabling bacteria to coordinate behaviors based on the population density^[^ ^2^ ^]^ However, it functions not merely as an ON‐OFF switch but as a sophisticated tuning mechanism, enhancing their response to varying physical environments.^[^ ^58^ ^]^ QS can finely tune various metabolic pathways related to nutrient acquisition and utilization.^[^ ^18^ ^]^ QS in *B. glumae* acts as a metabolic brake, slowing down primary metabolism in crowded conditions to ensure homeostasis.^[^ ^20^ ^]^ This involves the coordinated regulation of glucose uptake, energy production, and nucleotide biosynthesis, preventing metabolic imbalances and ensuring efficient resource utilization. In *P. aeruginosa*, QS leads to significant metabolic alterations, including TCA cycle changes and amino acid and fatty acid metabolism.^[^ ^59^ ^]^ QS inhibitors like resveratrol can significantly alter the metabolome of *P. aeruginosa*, affecting pathways related to oxidative stress and energy metabolism.^[^ ^60^ ^]^ Histidine metabolic imbalance impacts the expression of type III secretion genes through QS pathways in *P. aeruginosa*, linking nitrogen metabolism to virulence factor production.^[^ ^61^ ^]^ The integration of AI‐2‐based QS with metabolic cues in *Escherichia coli* influences the expression of genes related to amino acid uptake and catabolism, ensuring efficient utilization of environmental amino acids.^[^ ^62^ ^]^ Similarly, QS in *Vibrio harveyi* controls the expression of genes involved in nutrient uptake, including those related to sugar transport and the biosynthesis of methionine and thiamine.^[^ ^63^ ^]^ Thus, QS can finely tune bacterial metabolism to optimize nutrient acquisition and utilization, ensuring that the bacterial community can adapt to changing environmental conditions and maintain optimal growth and survival. The mechanisms of QS‐regulated cooperation in the production of public goods, particularly in the context of the metabolism involved in synthesizing carbon‐ and nitrogen‐based secondary metabolites, have yet to be fully demonstrated in bacterial communities.

We demonstrate that the QS system in *P. fluorescens* 2P24 imposes metabolic restrictions by regulating the expression of genes and metabolites, thereby maintaining the production of secondary metabolites within a cooperative carbon and nitrogen metabolism (Figure 5B). We integrated transcriptome, metabolome, and ChIP‐seq data to dissect the QS‐regulated metabolic network for secondary metabolites production in *P. fluorescens* 2P24. MupR decreases the levels of G6P, G3P and d‐sedoheptulose 7‐phosphate, which are associated with glycerol metabolism and pentose phosphate pathway, were significantly higher in Δ*mupR* mutant. Similarly, concentrations of UDP‐d‐galactose, glutaric acid, l‐2‐hydroxyglutaric acid, 3‐oxo‐dodecanoic acid, as well as fatty acid metabolites such as (S)‐3‐hydroxyoctanoic acid, 10‐hydroxy capric acid, 3s‐hydroxy‐dodecanoic acid were significantly decreased in Δ*mupR* mutant. Meanwhile, the concentration of S‐adenosylmethionine (SAM) and 5′‐methylthioadenosine (5′‐MTA) that related to AHLs biosynthesis were substantially decreased in Δ*mupR* mutant (Figure 5A). When QS is active, MupR represses transcriptional levels of *aceE*, *gltA*, *pycA*, *acnB*, *sucA*, and *sdhC* genes which encode the key enzymes from the TCA cycle. MupR reduces the level of glutarate, which can be converted to glutaryl‐CoA and succinate by succinyl‐CoA transferase, with the resulting succinate being fed into the TCA cycle for energy production. MupR downregulates malonyl CoA‐acyl carrier protein transacylase *fabD* for reducing the fatty acid synthesis and keeps phospholipids PE at a low level while increasing PG, PC, PS, and PA. PE is the major phospholipid in Gram‐negative bacteria.^[^ ^64^ ^]^ This implies that acetyl‐CoA and malonyl‐CoA can be accumulated, facilitating the QS system to synthesize polyketide antibiotics such as 2,4‐DAPG and mupirocin. These results indicate that QS allows bacteria to restrict the TCA cycle and enhance the biosynthesis of lipids and amino acids, as these pathways are interconnected with central carbon metabolism.

MupR repressed the gene transcriptional level of *sucA* gene, which encoded the 2‐oxoglutarate dehydrogenase E1 subunit responsible for converting 2OG to succinyl‐CoA.^[^ ^65^ ^]^ However, MupR does not affect isocitrate dehydrogenase, which is involved in the oxidative decarboxylation of isocitrate to 2OG. This indicates that these two steps of the TCA cycle work together to accumulate and increase 2OG levels. Indeed, metabolomic analysis showed that the levels of the key metabolite 2OG in the TCA cycle and Glu, were increased under the QS system. 2OG acts as a signaling molecule of nitrogen limitation, and regulates the activity of enzymes involved in nitrogen assimilation.^[^ ^66^ ^]^ MupR represses the genes *glnA1* and *gltB* in GS‐GOGAT pathway for ammonium assimilation and amino acid synthesis. The GS‐GOGAT pathway in bacteria is critical for assimilating ammonium into glutamine and glutamate, linking nitrogen metabolism with central carbon metabolism.^[^ ^67^, ^68^ ^]^ The accumulation levels of 2‐OG reflect the cellular nitrogen/carbon status. This downregulation of ammonium assimilation genes ensures a balanced metabolic flow, supporting the synthesis of both carbon‐ and nitrogen‐based public goods, thereby maintaining metabolic homeostasis.

MupR also downregulates genes involved in the denitrification pathway, including nitrate reductase‐related *narH*, nitrite reductases‐related *nirQS*, nitric oxide reductase (*norBC*), and a Crp/Fnr family transcriptional regulator downregulated by MupR. Additionally, MupR downregulates *ytfE*, which is associated with the bacterial response to oxidative and nitrosative stress,^[^ ^49^ ^]^ and *hmpA*, which plays a role in nitric oxide detoxification under aerobic conditions.^[^ ^50^ ^]^ These downregulated genes from the denitrification pathway imply a metabolic shift from energy‐intensive metabolism for cell growth toward energy‐efficient metabolism for the production of public goods. We also find that the QS system maintains a low ratio of NADH/NAD+, indicating sufficient electron transport chain and leading to energy production. These findings indicate that MupR slows down the TCA cycle or limits denitrification pathways to maintain homeostasis and functions as a metabolic brake to regulate energy production for cellular metabolism. Specifically, the downregulation of ammonium assimilation and amino acid synthesis may limit the availability of nitrogen precursors for siderophore synthesis. Concurrently, carbohydrate and fatty acid metabolism alterations shift central carbon intermediates toward the biosynthesis of key secondary metabolites such as 2,4‐DAPG and mupirocin, while decelerating energy production via the TCA cycle.

MupR plays a key role in regulating the balance between central metabolism and secondary metabolite production in *P. fluorescens* 2P24. By inhibiting respiration through the TCA cycle and denitrification pathways, MupR conserves energy and resources while promoting the synthesis of key carbon‐based secondary metabolites, including AHLs, 2,4‐DAPG and mupirocin. In contrast, the production of nitrogen‐based secondary metabolites, such as siderophores, is suppressed under nutrient‐limited conditions, reflecting a shift in metabolic priorities. This metabolic reorganization helps to balance cellular energy demands, ensuring optimal survival in fluctuating environments. Our findings demonstrate that QS functions as a multifaceted regulatory mechanism, enabling facultative cooperation and acting as a sophisticated sensor system that allows bacteria to monitor their intracellular carbon and nitrogen status. By coordinating carbon and nitrogen metabolic fluxes, QS helps maintain a balanced homeostasis of energy and nutrients, optimizing public goods in response to environmental changes, thereby supporting long‐term bacterial adaptation.

While the evolutionary origin of the QS system is debated, it is evident that the production of QS signal molecules, such as AHLs, is closely linked to intracellular carbon metabolism pathways. By modifying bacterial metabolic activities, the QS system mimics the effects of caloric restriction and induces the production of carbon‐based secondary metabolites and AHL signals. The metabolic feedback loops mediated by QS benefit individual bacterial cells and the microbial community by imposing stricter control over nutrient utilization and cellular energy consumption under high cell density conditions. In summary, the QS system may evolve from metabolic byproducts to an environmental sensor that ensures the homeostasis of carbon/nitrogen metabolism in individual cells under crowded conditions, making it a cooperative activity. By shedding light on the balance between private and public goods, our findings offer new insights into how QS coordinates metabolic fluxes in response to changing environmental conditions. This work addresses fundamental questions regarding the coexistence of cooperation and competition in microbial evolution and opens new avenues for exploring the dynamics of bacterial cell‐cell communication and its implications for microbial community behavior.

## 4 Experimental Section

### Bacterial Strains, Plasmids, Primers, Culture Conditions, and Chemical Compounds

The bacterial strains and plasmids used in this study are listed in Table S1 (Supporting Information). The primers used in this study are listed in Table S2 (Supporting Information). Bacteria were grown routinely in Luria‐Bertani (LB) broth (10 g L^−1^ tryptone, 5 g L^−1^ yeast extract, 10 g L^−1^ NaCl, pH 7.2) or LB agar (LB broth plus 1.5% agar), except that *P. fluorescens* strains were cultured at 28 °C, and *Escherichia coli* strains were cultured at 37 °C. For the synthesis of secondary metabolites or AHL extraction, *P. fluorescens* were grown in King's B (KB) medium with shaking at 200 rpm. Antibiotics used for selection were as follows: for *E. coli*, ampicillin at 100 µg mL, gentamicin at 15 µg mL^−1^, kanamycin at 30 µg mL^−1^ for *P. fluorescens*, ampicillin at 100 µg mL^−1^, gentamicin at 50 µg mL^−1^, kanamycin at 30 µg mL^−1^. 2,4‐DAPG was purchased from Toronto Research Chemicals (North York, Canada); Mupirocin ointment (containing 2% mupirocin) was purchased from pharmacy; all the AHLs including 3‐oxo‐C_6_‐HSL, 3‐oxo‐C_8_‐HSL, 3‐oxo‐C_10_‐HSL, and 3‐oxo‐C_12_‐HSL were purchased from Sigma‐Aldrich (Merck, Germany).

### Construction and Complementation of *P. fluorescens* 2P24 Mutants

For the generation of markerless genomic deletion mutants, a kanamycin‐resistance (*kanR*) selection and sucrose counterselection (*sacB*) process was used. This strategy provided specificity in gene deletions through homologous recombination while avoiding the retention of selection markers, ensuring a clean genomic background. The pK18mobsacB‐based deletion vector construct was transformed into chemically competent *E. coli* S17‐1λpir for intergenic conjugation with *P. fluorescens* 2P24. This plasmid has the pMB1 replicon, which does not function in *P. fluorescens*. Therefore, kanamycin selects for plasmids that were integrated into the *Pseudomonas* chromosome by homologous recombination. This vector also includes *sacB* that encodes levansucrase from *Bacillus subtilis*, which confers a lethal periplasmic sucrose polymerization in Gram‐negative bacteria. Biparental mating involved mixing overnight donor (*E. coli* S17‐1λpir) and acceptor (*P. fluorescens*) cultures in a 1:1 ratio and incubating on nonselective LB agar at 28 °C. Resulting colonies were selected on LB containing kanamycin and ampicillin for initial selection, followed by counterselection on 10% sucrose LB for double crossover mutants. About 50% of excisions should contain the mutation, because the chances of integration and excision events occurring on either homologous arm flanking mutations are equal. Mutants were confirmed by PCR using gene‐specific primers, ensuring the right size of PCR products and precise deletion of the target gene without affecting flanking regions.

Two DNA fragments flanking the *mupR* gene were amplified by PCR using primer pairs mupR‐F1/R1 to amplify 500 bp upstream of the ATG codon of *mupR* and mupR‐F2/R2 to amplify 500 bp downstream of the TAA stop codon of *mupR*. These fragments were joined by overlap extension PCR to create the mutator fragment for *mupR* gene deletion. The construct was digested with *EcoRI* and *HindIII* and ligated into pK18mobsacB to generate pK18‐ΔmupR, which was introduced into *P. fluorescens* 2P24. The deletion of mupR was confirmed by colony PCR using the external primers mupR‐F1 and mupR‐R2. PCR products were run on a 1% agarose gel to verify the correct size and purity, confirming successful deletion of the mupR gene. A similar strategy was used for *mupI* deletion, with primer pairs mupI‐F1/R1 and mupI‐F2/R2 amplifying the flanking regions and generating deletion mutant of *mupI*. The deletion construct, pK18‐ΔmupI, was confirmed by PCR after introduction into *P. fluorescens* 2P24. Sequential deletion of *mupR* and *mupI* was performed to generate the Δ*mupRΔmupI* double mutant. Internal deletions of *pvdL* (12476 bp) and *pvdDJI* (29049 bp) were created using a similar overlap extension PCR strategy with respective flanking primers *pvdL*‐F1/R1 and *pvdL*‐F2/R2 or *pvdDJI*‐F1/R1 and *pvdDJI* F2/R2. To ensure the specificity of the gene deletions, colony PCR was performed on several independent colonies for Δ*pvdL* and Δ*pvdDJI* mutants to verify the correct size of the PCR products. Stability of the mutants was ensured by growing the strains in nonselective medium for several generations. For each strain, the stability of the deletion was periodically checked by colony PCR.

For complementation, the complete open reading frames (ORFs) of target genes along with their upstream regulatory regions were PCR amplified and cloned into the broad‐host‐range vector pBBR5pemIK. For example, the *mupR* ORF and its upstream region (899 bp) were amplified using mupR‐C1/C2, digested with *KpnI* and *HindIII*, and ligated to pBBR5pemIK to create complementation construct pBBR5‐*mupR*. This construct was introduced into Δ*mupR* by biparental mating and the successful complementation was confirmed by PCR and phenotypic analysis. Complementation of Δ*mupI* and Δ*mupRΔmupI* was achieved using constructs carrying *mupI* or both *mupR* and *mupI*. To create a *P. fluorescens* strain expressing N‐terminally FLAG‐tagged MupR, a 3 × FLAG‐(GGGS)3 fragment was PCR amplified using mupRFLAG1/mupRFLAG2 and assembled with linearized pBBR5‐*mupR* using a seamless cloning kit (Sangon, China). This construct was introduced into Δ*mupR* by biparental mating. Successful expression of FLAG‐tagged *MupR* was confirmed by PCR and Western blotting using an anti‐FLAG antibody. The FLAG tag allowed for easy detection and isolation of the recombinant protein for subsequent functional and biochemical analyses.

### Biofilm Formation Assays

A biofilm detection experiment was performed as reported previously with minor modification.^[^ ^26^ ^]^ In brief, overnight bacterial culture was transferred to a 1.5‐mL EP tube containing 0.5 mL KB medium at an OD_600_ of 0.01 and cultured statically at 28 °C for 24 h. Crystal violet (0.1%) was used to stain biofilm adhered to the tubes for 20 min. The tubes were washed thoroughly with ddH_2_O until the waste liquid is colorless, and the remaining crystal violet was fully dissolved in 200 µL of 95% ethanol and the absorbance was detected at 570 nm.

### Quantification of Mupirocin and 2,4‐DAPG

*P. fluorescens* 2P24 and derivative strains were inoculated at a starting OD_600_ of 0.02 in 50 mL King's B medium and cultured for 60 h. After 24 h of the fermentation culture, 1 mL of culture broth was collected every 12 h and centrifuged at 12 000 rpm for 10 min, then the supernatant was filtered through 0.22 µm MCE syringe filter and further analyzed by high‐performance liquid chromatography (LC‐2030plus, SHIMADZU, Japan) equipped with a SHIMSEN Superb II C18 column (4.6 mm × 150 mm, 4.6 µm, SHIMADZU, Japan). To quantify the production of Mupirocin, sample fractions were separated isocratically by elution with 40:60 (vol/vol) acetonitrile‐NH_4_H_2_PO_4_ (5 g L^−1^) over 10 min at 230 nm with a flow rate of 1 mL min^−1^. To quantify the production of DAPG, separation was achieved with mobile phase water/acetonitrile gradient (5%–100% acetonitrile with 0.1% TFA) over 15 min at 270 nm with a flow rate of 1 mL min^−1^. Compared with the standard profile of Mupirocin and DAPG, quantification was performed by integrating the area under the curve at the corresponding wavelength. Data was processed with GraphPad Prism version 10.2.1 (GraphPad Software, USA). Three independent experiments were performed and the error bars were calculated.

### Detection and Measurement of Pyoverdine Production

To visualize the fluorescence of pyoverdine production in liquid cultures, strains were inoculated in 50 mL King's B medium, then different time‐point culture broths were collected and compared their fluorescences under UV light. Alternatively, the liquid version of the CAS assay was used, where 100 µL cell‐free supernatant or fresh King's B medium as a control reference was added to an equal volume of CAS assay solution in a 96‐well plate. After 2 h of static incubation at room temperature, the OD_630_ of the cell‐free supernatant (A) or fresh King's B medium (Ar) was then measured using a microplate reader (SpectraMax iD3, Molecular Devices, USA). Pyoverdine induces a color change in CAS assay solution and pyoverdine production can thus be quantified by the following formula: 1‐A÷Ar. Pyoverdine‐based fluorescence of *P. fluorescens* 2P24 and derivative strains were quantified using a microplate reader by recording emission at 447 nm upon excitation at 400 nm. Pyoverdine production levels (relative fluorescence units, RFU) were scaled by dividing fluorescence reading by OD_600_. Assays were performed in at least three duplicates.

### Chromatin Immunoprecipitation and ChIP‐seq Analysis

Chromatin immunoprecipitation (ChIP) assay was performed as previously described but with some variations.^[^ ^69^ ^]^ For ChIP, four biological replicates of *P. fluorescens* 2P24 and derivative strains were grown under the conditions specified in the experimental design. Δ*mupR*::pBBR5‐3xFLAG‐*mupR* strains were grown in three 20 mL volumes of KB medium at 28 °C for 16 h. DNA was cross‐linked to transcription factors with 1% formaldehyde, and the cross‐linking was stopped by 125 × 10^−3^ m glycine. The harvest cells were washed twice with 20 mL Tris buffer (20 × 10^−3^ m Tris‐HCl pH 7.5, 150 × 10^−3^ m NaCl), then resuspended in 500 µL IP buffer (15 × 10^−3^ m HEPES pH 8.0, 125 × 10^−3^ m NaCl, 1 × 10^−3^ m EDTA, 1% Triton X‐100, 0.1% sodium deoxycholate, 1 tablet cocktail‐inhibitors (Roche)). Chromosomal DNA was sheared by sonication to fragments ranging from 100 to 500 bp on average, which was confirmed by agarose gel electrophoresis. The samples were centrifuged to collect the supernatant at 12 000 rpm at 4 °C for 10 min, after which 50 µL of each was set aside for total DNA extraction (input). Then 40 µL anti‐FLAG magnetic beads (Biolinkedin, L‐1011) were washed with 1 mL five times and added to the remaining supernatant. The mixtures were then incubated on a rotating wheel at 4 °C overnight. The samples were placed on a magnetic separator to collect the pellets, and the collected pellets were sequentially washed four times with IP buffer and twice with TE buffer (10 × 10^−3^ m Tris‐HCl pH 8.0, 1 × 10^−3^ m EDTA). The pellets and 50 µL input samples (set aside earlier) were incubated with 100 µL elution buffer A (50 × 10^−3^ m Tris‐HCl pH 7.6, 10 × 10^−3^ m EDTA, 1% SDS) at 65 °C for 10 min. The pellets were collected by magnetic separator and re‐exported with 150 µL elution buffer B (10 × 10^−3^ m Tris‐HCl pH 8.0, 1 × 10^−3^ m EDTA, 0.67% SDS). The two‐step elution samples were combined (IP sample) and incubated at 65 °C overnight to reverse the cross‐links. An equal volume of proteinase K solution (0.4 mg mL^−1^ proteinase K and 0.04 mg mL^−1^ glycogen in TE buffer) was added to the IP samples and incubated at 58 °C for 2 h. Then 55 µL 4 m LiCl was added to the samples and mix well. The DNA was then precipitated, dissolved in 25 µL nuclease‐free water, and stored for further analysis.

ChIP‐seq libraries were prepared and sequenced on Illumina Hiseq or Novaseq by GENEWIZ, Inc. (Suzhou, China). Raw sequencing data in FASTQ format were processed using Cutadapt (v1.9.1) to remove adapter sequences, low‐quality bases (below Q20), and PCR artifacts. The clean reads were aligned to the genomic DNA sequence of *P. fluorescens* 2P24 (Accession No. NZ_CP025542.1) using the bowtie2 software (v2.2.6). The enriched peaks were identified using MACS2 (v2), and annotated using ChIPseeker (v3.11). Finally, MEME Suite analysis was performed to identify the MupR binding motif.

### RNA‐Seq Analysis

Overnight cultures of *P. fluorescens* 2P24 and Δ*mupR* mutant were inoculated into 50 mL KB medium at a final OD_600_ of 0.02. For RNA‐seq sampling, each strain was set up with three parallel replicates. After 20 h of incubation at 28 °C with shaking at 200 rpm, *P. fluorescens* 2P24 and Δ*mupR* mutant was collected at the late exponential phase (OD_600_ = 4) by centrifuging at 4 °C. RNA extraction and transcriptome sequencing was conducted by OE Biotech (Shanghai, China). The samples with an RNA integrity number of ≥7 subjected to the subsequent analysis. After removing rRNAs and fragmenting RNAs, cDNAs were synthesized and further processed for library construction. Then these library sequencing were performed on the Illumina HiSeq 2500 (San Diego, CA).

Sequenced reads containing the adapter, poly‐N and low‐quality bases (*Q* < 20) were cleaned by Trimmomatic (v0.36). The clean reads were mapped onto the complete genome of the *P. fluorescence* 2P24 (GenBank Accession No. NZ_CP025542.1) using Rockhopper 2 (v2). The mRNA abundance of genes was calculated using the reads per kilobase per million mapped reads (RPKM) method. The biological replicates of *P. fluorescens* 2P24 and the Δ*mupR* mutant exhibited strong inter‐repeat correlation, with Pearson correlation coefficients exceeding 0.9, confirming the reliability and consistency of the transcriptomic data. The differential gene expression between *P. fluorescence* 2P24 and Δ*mupR* mutant (Δ*mupR* mutant vs wild‐type 2P24) was analyzed using the DeSeq2 normalized method on a bioconductor platform. The transcripts with the adjusted *P*‐value < 0.05 and the absolute fold change ≥ 1.5 deemed as the differentially expressed genes (DEGs). Further enrichment analyses including GO and KEGG were performed for these DEGs.

### Purification and LC‐MS Analysis of AHLs

For AHL extraction, 10 mL overnight cultured *P. fluorescens* 2P24 were inoculated into four 200 mL volumes of King's B medium, incubated at 28 °C with shaking at 200 rpm for different times, Δ*mupI* mutant was also inoculated as control. The whole 200 mL supernatant was extracted twice with equal volumes of ethyl acetate, then the combined extracts were evaporated to 5 mL and ultimately to dryness by lyophilization. Samples were dissolved in 500 µL LC/MS grade ethyl acetate.

AHLs were then analyzed by high‐resolution mass spectrometry (HRMS). The LC‐MS measurements were carried out on an Accela UPLC system (Thermo Scientific, Germany) equipped with an Accucore C18 column (2.6 µm, 100 × 2.1 mm) coupled with a TSQ Quantis mass spectrometer (Thermo Scientific, Germany) with an electrospray ionization (ESI) source. The ESI was used in positive mode with the positive ion spray voltage set at 3.5 kV, the ion transfer tube temperature at 275 °C and the vaporizer temperature at 75 °C. The scan range of MS was set to m/z 100–500 with a scan rate of 1000 Da s^−1^. LC method was used as follows: flow rate = 0.1 mL min^−1^, linear gradient 20%–100% MeCN in water containing 0.1% formic acid over 30 min.

### Reverse Transcription‐Quantitative PCR

*P. fluorescens* 2P24 and mutants were grown under the same conditions as those used for 2,4‐DAPG and mupirocin quantification, and 1 mL culture broth was collected after 20 h incubation. Total bacterial RNA was extracted from *P. fluorescens* using a bacteria RNA extraction kit (Vazyme, China). RNA samples were reverse‐transcribed to cDNAs using the HiScript III RT SuperMix for qPCR (+gDNA wiper) (Vazyme, China) following the manufacturer's protocol. Real‐time PCR assays were performed on ABI QuantStudio 6 (Thermo Scientific, Germany) with the AceQ Universal SYBR qPCR Master Mix (Vazyme, China). All gene threshold cycle (*C_T_*) values were normalized to those of 16S rRNA, and the expression levels of genes were calculated by the 2^‐ΔΔCT^ method. For RT‐qPCR assays, experiments were set up in triplicate, with three technical repeats per sample.

### Metabolome Analysis

*P. fluorescens* 2P24 and Δ*mupR* mutant strain were cultured in 50 mL KB medium for 36 h, with seven parallel replicates for each strain. Culture aliquots (2 mL) were collected and normalized to the same OD_600_. Intracellular metabolites of *P. fluorescens* 2P24 and Δ*mupR* mutant were extracted as follows. The cell pellets were resuspended in 1 mL cold methanolic solution of internal standards (4 µg mL^−1^) plus 200 µL chloroform, then the mixtures were ultrasonicated for 3 min and extracted at 60 Hz for 20 min, all the extraction procedures were performed in ice‐water. After centrifugation at 12 000 rpm, 4 °C for 10 min, the supernatants were collected and volatilized to dryness, then dissolved in 300 µL methanol: water (v/v = 4:1) and filtered with 0.22 µm strainers. The metabolite profile was analyzed with ACQUITY UPLC system (Waters, USA) tandem Q‐Exactive mass spectrometer system (Thermo Scientific, Germany), equipped with an ACQUITY UPLC HSS T3 column (1.8 µm, 100 mm × 2.1 mm). Water (0.1% formic acid) and acetonitrile (0.1% formic acid) were used as mobile phase, with a linear gradient of 5% acetonitrile to 100% acetonitrile in water containing 0.1% formic acid at a flow rate of 0.35 mL min^−1^. Both ESI‐positive and ESI‐negative ion modes were scanned ranging from 100 to 1200 m/z, and data‐dependent acquisition (DDA) mode was applied to acquire data.

The raw data were processed using Progenesis QI (v3.0, Waters, USA) based on public databases, including the Human Metabolome Database (HMDB), lipid maps (v2.3), and METLIN Database and LuMet‐Animal (3.0). To ensure data quality and reproducibility, multivariate and univariate statistical analyses were performed using the OECloud platform (https://cloud.oebiotech.com, OE Biotech, Shanghai, China). To reduce the impact of noise and high variance, the data were first normalized and logarithmically transformed. Principal component analysis (PCA) was then performed to explore the overall distribution of the samples and to identify potential outliers. A 95% confidence interval in the PCA score plot was used to define the threshold for outlier detection. For more detailed analysis, orthogonal projections to latent structures‐discriminant analysis (OPLS‐DA) were applied to model the first principal component. To assess the robustness and predictive ability of the OPLS‐DA model, 200 permutation tests were conducted. The model's performance was evaluated using the *R* ^2^ *Y* and *Q* ^2^ parameters derived from sevenfold cross‐validation. The *R* ^2^ *Y* value was 0.995, indicating strong model interpretability, while the *Q* ^2^ value was −0.655, suggesting the model's predictive accuracy.

Variable importance in projection (VIP) scores were calculated to assess the contribution of each metabolite to the model. Metabolites with VIP > 1 and *P*‐values < 0.05 (from Student's *t*‐test) were considered significantly altered. Finally, differential metabolites were selected based on VIP > 1 and *P*‐value < 0.05, and pathway enrichment analysis was performed using KEGG annotations to interpret the biological relevance of these changes.

### Transcriptional Reporter Assay

The plasmid pSEVA225E was used for transcriptional reporter assay, which contains a promoterless *egfp*. To construct this plasmid, the coding region of EGFP was amplified using primer pair 225egf‐F/225egfp‐R and the plasmid pEGFP‐N1 as a template. Then the resultant PCR fragment was digested with *Hin*dIII and *Spe*I, and ligated into pSEV225T (GenBank Accession No. KC847299.1) to generate the plasmid pSEVA225E. To verify the transcriptional regulation of MupR, promoters of potential target genes were fused to the promoterless *egfp*. These pSEVA225E‐based reporter constructs were transferred into *P. fluorescens* 2P24 and Δ*mupR* separately. Strains were cultured in 5 mL King's B medium at 28 °C with shaking. Culture broths were collected at different time points, and cell pellets were washed once with sterile water and resuspended in sterile water. Simultaneously, 200‐µL suspensions of each sample were transferred to a transparent 96‐well plate and a black 96‐well plate. Using a microplate reader (SpectraMax iD3, Molecular Devices, USA), the OD_600_ was measured to monitor the cell density, and the green fluorescens of bacteria were monitored by measuring emission at 535 nm with an excitation at 485 nm. For each measurement, the relative fluorescence unit (RFU) was scaled by dividing the green fluorescence value by the corresponding OD_600_. Each data point represents the average of three technical replicates from three independent biological replicates.

### Statistical Analysis

Statistical analyses were performed using GraphPad Prism version 10.2.1. A two‐way ANOVA with multiple comparisons test was used to assess significance between all groups. For comparisons between two means, a two‐tailed Student's *t*‐test was applied. Unless otherwise stated, data are presented as mean ± standard deviation (SD).

## Conflict of Interest

The authors declare no conflict of interest.

## Data Availability

Untarget metabolomic data used in this publication were deposited to EMBL‐EBI Metabolights database with the identifier MTBLS10731.The ChIP‐seq data files were submitted to the National Center for Biotechnology Information (NCBI) under Accession No. PRJNA1138317. The RNA‐seq data sets were submitted to the National Center for Biotechnology Information (NCBI) under Accession No. PRJNA1138253.

## Supporting information



Supporting Information

## Data Availability

The data that support the findings of this study are available in the supplementary material of this article.
