# Supplementary material for: Quorum Sensing Coordinates Carbon and Nitrogen Metabolism to Optimize Public Goods Production in Pseudomonas fluorescens 2P24
Source: Adv Sci (Weinh). 2025 Jan 31;12(12):2412224. doi: 10.1002/advs.202412224 (PMC11948153; doi:10.1002/advs.202412224)
Supplement: Supplementary file 1 — Supporting Information [file ADVS-12-2412224-s001.docx]

Supporting Information

**Quorum Sensing Coordinates Carbon and Nitrogen Metabolism to Optimize Public Goods Production in *Pseudomonas fluorescens* 2P24**

*Jie Li, Mengxue Nie, Hongguang Ma, Xuanying Tao, Yanxia Sun, Xinyue Tu, Pingping Zhang, Li-Qun Zhang, Rong Jia, Yong-Xing He, Nannan Zhang^*^and Honghua Ge^*^*

Jie Li, Mengxue Nie, Hongguang Ma, Xuanying Tao, Yanxia Sun, Xinyue Tu, Pingping Zhang, Rong Jia, Nannan Zhang and Honghua Ge

School of Life Sciences, Anhui University, Hefei, China

Email: zhangnn@ahu.edu.cn; hhge@ahu.edu.cn

Jie Li, Hongguang Ma and Honghua Ge

Institute of Health Sciences and Technology, Institutes of Physical Science and Information Technology, Anhui University, Hefei, China

1. mail: hhge@ahu.edu.cn

Li-Qun Zhang
College of Plant Protection, China Agricultural University, Beijing, China

Yong-Xing He

Ministry of Education Key Laboratory of Cell Activities and Stress Adaptations, School of Life Sciences, Lanzhou University, Lanzhou 730000, China

School of Veterinary Medicine and Biosecurity, Lanzhou University, Lanzhou 730000, China

**Fig S1**. (A) HPLC and LC-MS analysis of mupirocin produced by *P. fluorescens* 2P24. HPLC represents the samples of an extract prepared from the culture medium of *P. fluorescens* 2P24 and the standard chemicals of mupirocin (left). Further analysis of MS/MS spectra confirmed the identity of mupirocin with *m*/*z* 501.4 extracted from the culture medium of *P. fluorescens* 2P24 (right). (B) UV and fluorescence spectra of PVD and PVD-Fe from *P. fluorescens* 2P24 cultured in liqiud MKB medium. UV spectra of PVD (black line) and PVD-Fe (grey line), and fluorescent spectrum of PVD (green line) and PVD-Fe (yellow line). Comparison of the PVD phenotypic differences between *P. fluorescens* 2P24 and mutants cultured in liquid MKB medium by CAS plate.


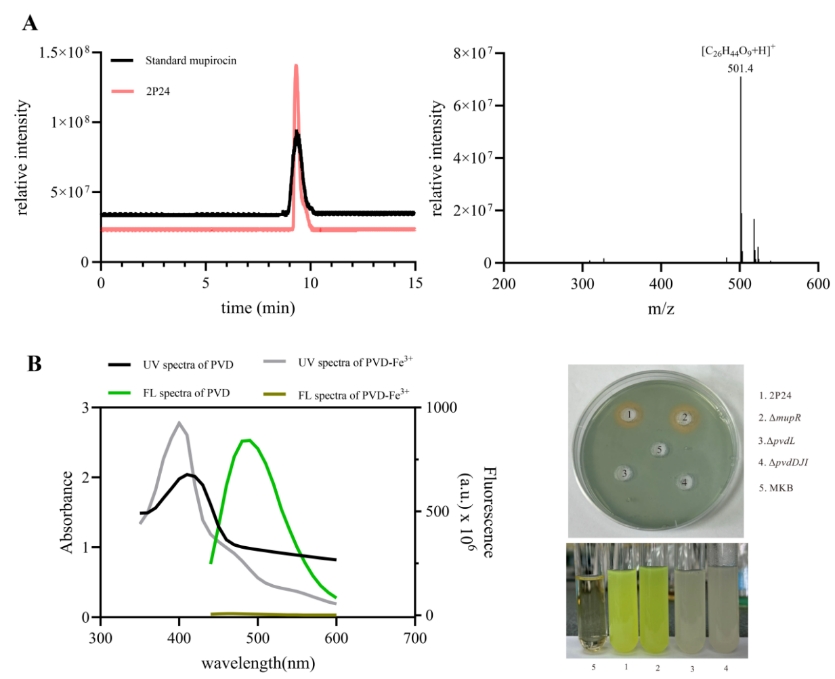


**Fig S2**. Dynamic monitoring of public goods production. (A) 2,4-DAPG, (B) mupirocin, (C) Pyoverdine. Samples were taken every 4 hours. Error bars denote standard deviations (n = 3).


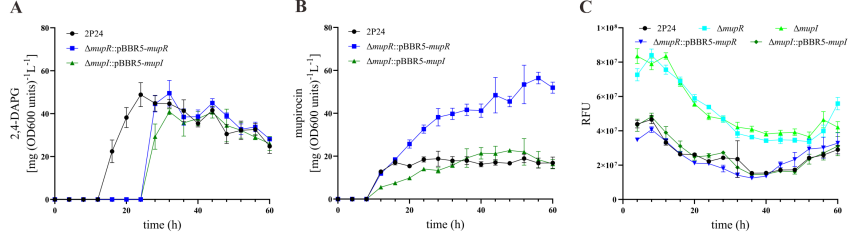


**Fig S3**. QS controlled biofilm formation. Biofilm formation phenotype of wild-type strain 2P24 and QS null mutants in KB medium. All experiments were performed in triplicate, and the mean values ± standard deviations are indicated. Statistical analyses were performed using the one-way ANOVA, *p* < 0.0001 is displayed as ****, *p* < 0.001 is displayed as ***.


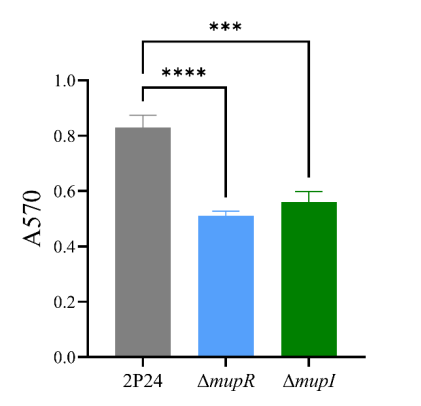


**Fig S4.** KEGG pathway enrichment analysis was conducted on MupR target genes identified from four biological replicates in ChIP-seq datasets. The analysis focused on KEGG Orthology (KO) terms, defining significantly enriched terms as those associated with peak-related genes having a *p*-value of less than 0.05.


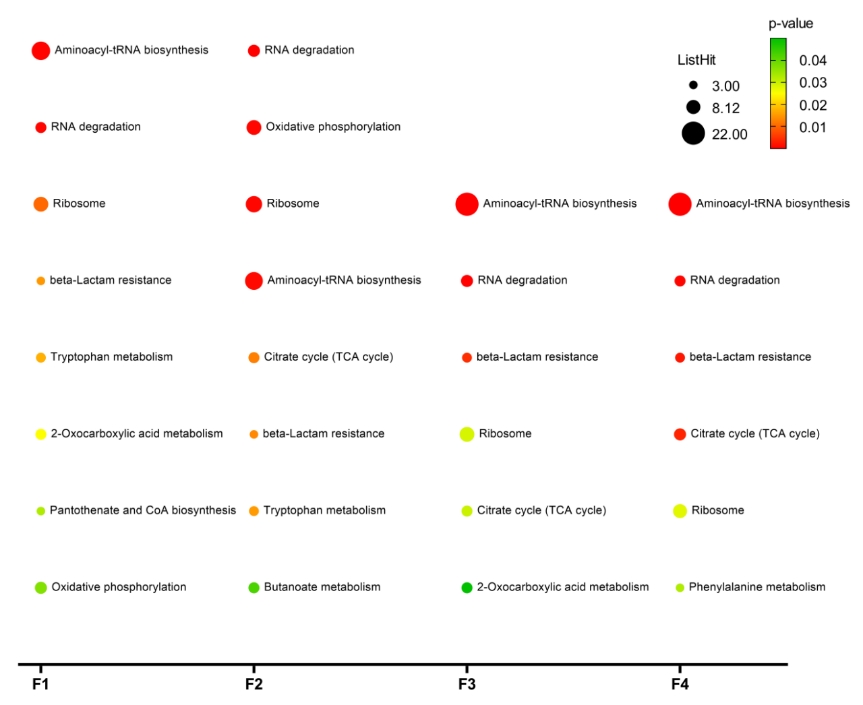


**Fig S5.** The transcription factor MupR activates gene transcription of the *phl* and *mup* biosynthetic gene clusters. (A) Schematic representation of operons in the *phl* gene cluster predicted by Operon-mapper prediction with the same color (top panel). Relative expression level of *phlF*, *phlA* and *phlD* as ChIP-seq targets from the *phl* gene cluster were quantified by RT-PCR (middle panel). The differentially expressed regulatory genes were evaluated in WT and Δ*mupR* strains, in which the reporter gene *egfp* was placed under the control of *phlF*, *phlA* and *phlD* promoters (bottom panel). (B) Schematic representation of operons in the *mup* gene cluster predicted by Operon-mapper prediction with the same color (top panel). Relative expression level of *mupZ*, *mmpB*, *mupF, macpC, mupO, macpE, mupR* and *mupI* as ChIP-seq targets from the *mup* gene cluster were quantified by RT-PCR (middle panel). The differentially expressed regulatory genes were evaluated in WT and Δ*mupR* strains, in which the reporter gene *egfp* was placed under the control of *mupZ*, *mmpB*, *mupF, macpC, mupO, macpE, mupR* and *mupI* promoters (bottom panel). The fluorescence activities were measured, and the bars illustrate the relative fluorescent units (RFU) normalized to a 1 ml culture with OD_600_= 1. Error bars denote standard deviations (n = 3). Statistical analyses were performed using the t test and two-way ANOVA, *p* < 0.0001 is displayed as ****, *p* < 0.001 is displayed as ***, *p* < 0.01 is displayed as **, *p* < 0.05 is displayed as *.


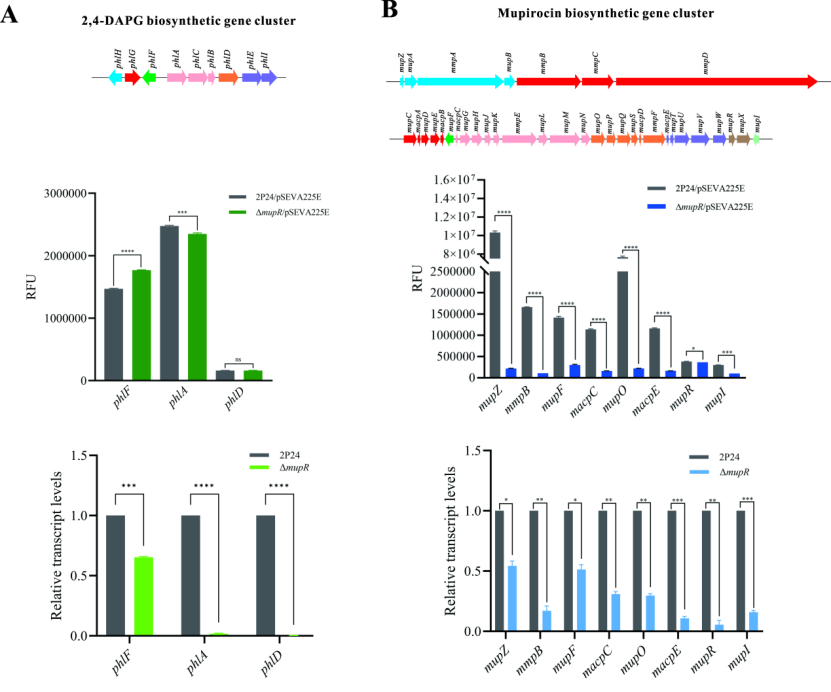


**Fig S6.** Verification of transcriptome data by RT-qPCR. (A) down-regulated genes in the Δ*mupR* strain compared with WT strain, (B) up-regulated in the Δ*mupR* strain compared with WT strain. Error bars denote standard deviations (n = 3). Statistical analyses were performed using the two-way ANOVA, *p* < 0.0001 is displayed as ****, *p* < 0.001 is displayed as ***, *p* < 0.01 is displayed as **, *p* < 0.05 is displayed as *.


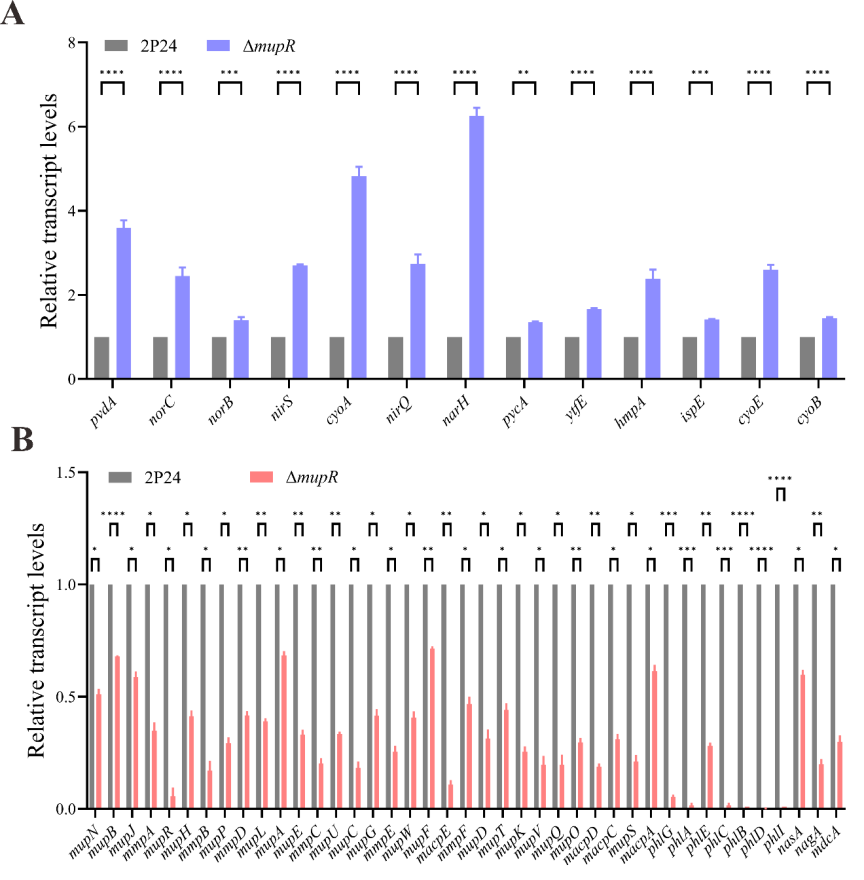


**Fig S7.** A structural model of MupR dimer docked with 3-oxo-C10-HSL and comparison of the AHL binding site was compared with those in LasR and QscR from *P. aeruginosa.* (A) A close-up view of the interaction mode between the AlphaFold-predicted MupR (shown in transparent green) and the docked 3-oxo-C10-HSL (shown in cyan). Key residues around the 3-oxo-C10-HSL binding site are highlighted in green, are shown in stick, illustrating the potential interactions. (B) A close-up view of the interaction mode in QscR bound to 3-oxo-C12-HSL (PDB code 6CC0) (shown in transparent grey). The binding site for 3-oxo-C12-HSL and surrounding critical residues are shown in grey sticks. (C) A close-up view of the interaction mode in LasR bound to 3-oxo-C12-HSL (PDB code 2UV0) (shown in transparent orange). The 3-oxo-C12-HSL binding site and surrounding residues are depicted in orange sticks. All structure figures were prepared with PyMOL.


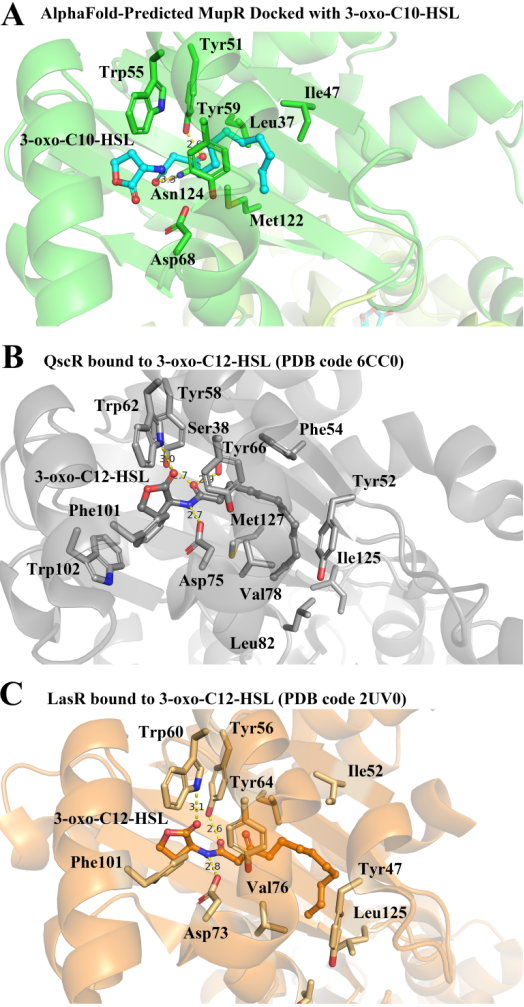


**Fig S8.** ChIP-seq reveals MupR binding sites present in *phl* and *mup* gene clusters indicating direct control by MupR. The top track in each Integrative Genome Viewer image shows the MupR ChIP enriched (MupR+) binding profiles in proximity to genes of interest. A track with the input DNA (input) is shown as a negative control.


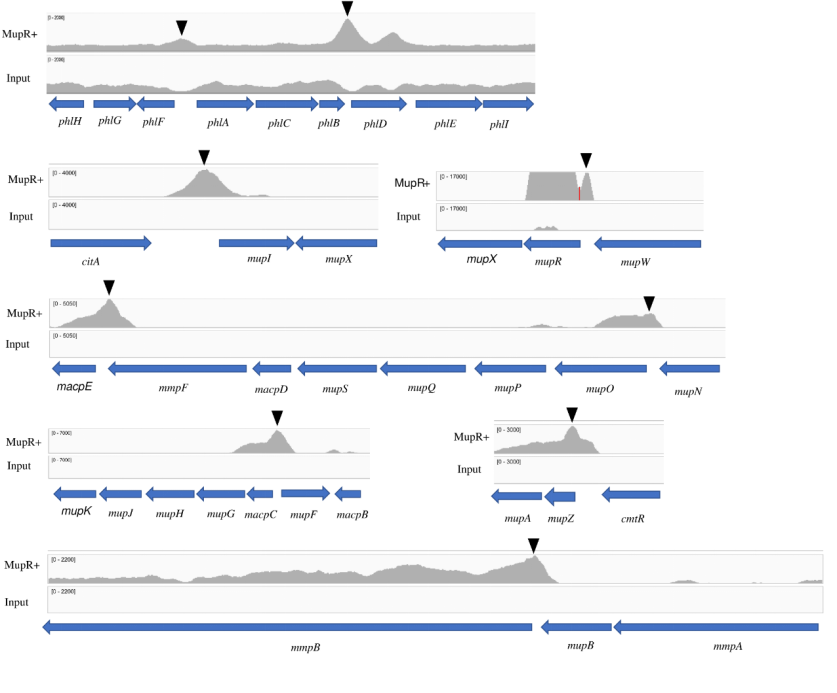


**Fig S9.** Evaluation of transcriptional activity in wild-type and *ΔmupR* strains following deletion of core MupR binding motifs from target gene promoters. (A) Analysis of differentially expressed genes activated under promoters lacking MupR binding motifs, monitored *via* EGFP reporter gene expression. (B) Comparative expression analysis of repressed genes in both strains, using promoters modified to exclude core MupR binding motifs. The fluorescence activities were measured, and the bars illustrate the relative fluorescent units (RFU) normalized to a 1 ml culture with OD_600_= 1. Error bars denote standard deviations (n = 3). Statistical analyses were performed using the t test and two-way ANOVA, *p* < 0.0001 is displayed as ****, *p* < 0.001 is displayed as ***, *p* < 0.01 is displayed as **, *p* < 0.05 is displayed as *.


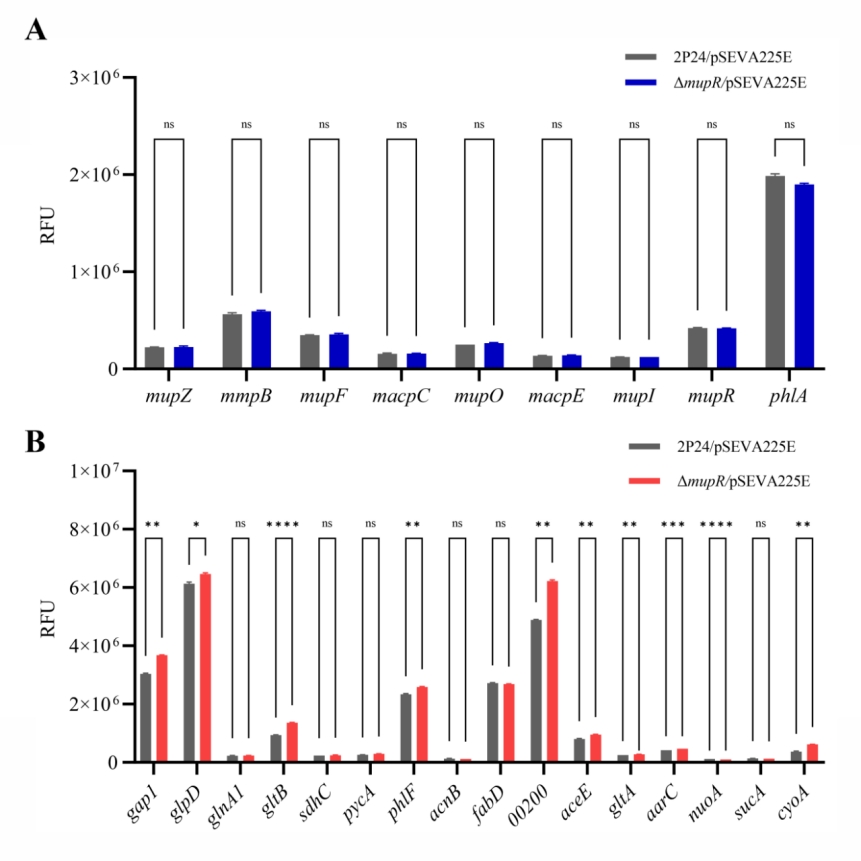


**Fig S10**. The heatmap showed the abundance of metabolites in different clusters from each sample.


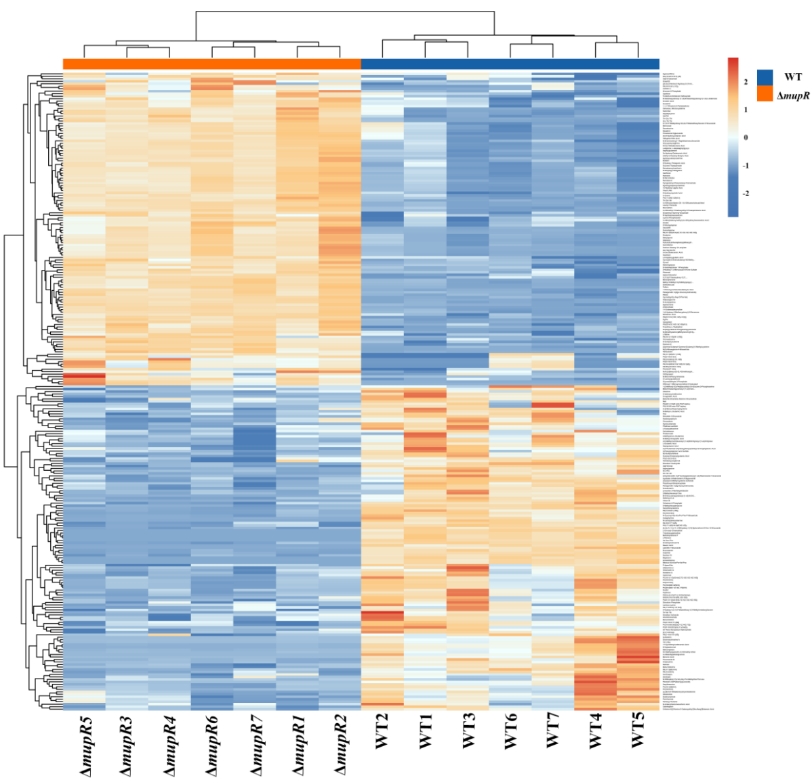


**Table S1. Bacterial strains and plasmids used in this study**.

| Strains and plasmids | Genotype and characteristics | Reference and source |
| --- | --- | --- |
| **Strains** |  |  |
| ***P. fluorescens*** |  |  |
| 2P24 | Wild type; Ap^r^ | (1) |
| Δ*mupR* | *mupR* gene in-frame deletion in strain 2P24; Ap^r^ | This study |
| Δ*mupI* | *mupI* gene in-frame deletion in strain 2P24; Ap^r^ | This study |
| Δ*pvdL* | *pvdL* gene in-frame deletion in strain 2P24; Ap^r^ | This study |
| Δ*pvdIDJ* | *mupIDJ* gene in-frame deletion in strain 2P24; Ap^r^ | This study |
| Δ*mupR/*pBBR5 | Δ*mupR* with plasmid pBBR5pemIK; Gm^r^ | This study |
| Δ*mupR::pBBR5*-*mupR* | Δ*mupR* with plasmid *pBBR5*-*mupR*; Gm^r^ | This study |
| Δ*mupI/*pBBR5 | Δ*mupI* with plasmid pBBR5pemIK; Gm^r^ | This study |
| Δ*mupI::pBBR5*-*mupI* | Δ*mupI* with plasmid *pBBR5*-*mupI*; Gm^r^ | This study |
| Δ*mupR/*pBBR5-3 x FLAG | Δ*mupR* with plasmid pBBR5-3 x FLAG; Gm^r^ | This study |
| Δ*mupR/*pBBR5-3 x FLAG-*mupR* | Δ*mupR* with plasmid pBBR5-3 x FLAG-*mupR*; Gm^r^ | This study |
| Δ*phlD* | *phlD* gene in-frame deletion in strain 2P24; Ap^r^ | Lab stock |
| Δ*mmpD* | *mmpD* gene in-frame deletion in strain 2P24; Ap^r^ | This study |
| ***E. coli*** |  |  |
| S17-1 λpir | RP4-2(Km::Tn7,Tc::Mu-1) pro-82 LAMpir recA1 endA1 thiE1 hsdR17 creC510 | Weidi Biotechnology Co., Ltd |
| HB101/pRK2013 | HB101 contains helper plasmid pRK2013 | (2) |
| **plasmid** |  |  |
| pK18*mobsacB* | Broad-host-range gene replacement vector; sacB^+^ Km^r^ | (3) |
| pBBR5pemIK | Broad-host-range cloning vector; Gm^r^ | Addgene |
| pK18-Δ*mupR* | pK18*mobsacB* containing *mupR* with an internal deletion | This study |
| pK18-Δ*mupI* | pK18*mobsacB* containing *mupI* with an internal deletion | This study |
| pK18-Δ*pvdL* | pK18*mobsacB* containing *pvdL* with an internal deletion | This study |
| pK18-Δ*pvdIDJ* | pK18*mobsacB* containing *pvdIDJ* with an internal deletion | This study |
| pBBR5-*mupR* | pBBR5pemIK carrying *mupR* driven by its own promoter | This study |
| pBBR5-*mupI* | pBBR5pemIK carrying *mupI* driven by its own promoter | This study |
| pBBR5-3 x FLAG | pBBR5pemIK carrying sequence encoding 3 x FLAG-(Gly_4_Ser)_3_ driven by *mupR* promoter | This study |
| pBBR5-3 x FLAG-*mupR* | pBBR5pemIK carrying sequence encoding 3 x FLAG-(Gly_4_Ser)_3_-MupR driven by *mupR* promoter | This study |
| pSEVA225E | pSEVA225T with *lacZ* substitute by *egfp* | Lab stock |
| pSEVA225E-PmupZ | promoter of *mupZ* fused with a promoterless *egfp* | This study |
| pSEVA225E-PmmpB | promoter of *mmpB* fused with a promoterless *egfp* | This study |
| pSEVA225E-PmupF | promoter of *mupF* fused with a promoterless *egfp* | This study |
| pSEVA225E-PmacpC | promoter of *macpC* fused with a promoterless *egfp* | This study |
| pSEVA225E-PmupO | promoter of *mupO* fused with a promoterless *egfp* | This study |
| pSEVA225E-PmacpE | promoter of *macpE* fused with a promoterless *egfp* | This study |
| pSEVA225E-PmupR | promoter of *mupR* fused with a promoterless *egfp* | This study |
| pSEVA225E-PmupI | promoter of *mupI* fused with a promoterless *egfp* | This study |
| pSEVA225E-PphlF | promoter of *phlF* fused with a promoterless *egfp* | This study |
| pSEVA225E-PphlA | promoter of *phlA* fused with a promoterless *egfp* | This study |
| pSEVA225E-PphlD | promoter of *phlD* fused with a promoterless *egfp* | This study |
| pSEVA225E-PphlE | promoter of *phlE* fused with a promoterless *egfp* | This study |
| pSEVA225E-PfadD | promoter of *fadD* fused with a promoterless *egfp* | This study |
| pSEVA225E-PglpD | promoter of *glpD* fused with a promoterless *egfp* | This study |
| pSEVA225E-PgltA | promoter of *gltA* fused with a promoterless *egfp* | This study |
| pSEVA225E-PaceE | promoter of *aceE* fused with a promoterless *egfp* | This study |
| pSEVA225E-Pgap1 | promoter of *gap1* fused with a promoterless *egfp* | This study |
| pSEVA225E-PsucA | promoter of *sucA* fused with a promoterless *egfp* | This study |
| pSEVA225E-PC0J56_RS01565 | promoter of *C0J56_RS01565* fused with a promoterless *egfp* | This study |
| pSEVA225E-PfabD | promoter of *fabD* fused with a promoterless *egfp* | This study |
| pSEVA225E-PC0J56_RS10665 | promoter of *C0J56_RS10665* fused with a promoterless *egfp* | This study |
| pSEVA225E-PpycA | promoter of *pycA* fused with a promoterless *egfp* | This study |
| pSEVA225E-PC0J56_RS00995 | promoter of *C0J56_RS00995* fused with a promoterless *egfp* | This study |
| pSEVA225E-PC0J56_RS21195 | promoter of *C0J56_RS21195* fused with a promoterless *egfp* | This study |
| pSEVA225E-PwbpA | promoter of *wbpA* fused with a promoterless *egfp* | This study |
| pSEVA225E-PilvB | promoter of *ilvB* fused with a promoterless *egfp* | This study |
| pSEVA225E-PalgD | promoter of *algD* fused with a promoterless *egfp* | This study |
| pSEVA225E-PsdhC | promoter of *sdhC* fused with a promoterless *egfp* | This study |
| pSEVA225E-PacpP | promoter of *acpP* fused with a promoterless *egfp* | This study |
| pSEVA225E-PC0J56_RS16940 | promoter of *C0J56_RS16940* fused with a promoterless *egfp* | This study |
| pSEVA225E-PC0J56_RS16615 | promoter of *C0J56_RS16615* fused with a promoterless *egfp* | This study |
| pSEVA225E-PacnB | promoter of *acnB* fused with a promoterless *egfp* | This study |
| pSEVA225E-PhcnA | promoter of *hcnA* fused with a promoterless *egfp* | This study |
| pSEVA225E-PgbpA | promoter of *gbpA* fused with a promoterless *egfp* | This study |
| pSEVA225E-PgltB | promoter of *gltB* fused with a promoterless *egfp* | This study |
| pSEVA225E-PglnA1 | promoter of *glnA*1 fused with a promoterless *egfp* | This study |
| pSEVA225E-PC0J56_RS19720 | promoter of *C0J56_RS19720* fused with a promoterless *egfp* | This study |
| pSEVA225E-PgltS | promoter of *gltS* fused with a promoterless *egfp* | This study |
| pSEVA225E-PwbmI | promoter of *wbmI* fused with a promoterless *egfp* | This study |
| pSEVA225E-PC0J56_RS25795 | promoter of *C0J56_RS25795* fused with a promoterless *egfp* | This study |
| pSEVA225E-PcyoA | promoter of *cyoA* fused with a promoterless *egfp* | This study |
| pSEVA225E-PC0J56_RS19015 | promoter of *C0J56_RS19015* fused with a promoterless *egfp* | This study |
| pSEVA225E-PC0J56_RS00200 | promoter of *C0J56_RS00200* fused with a promoterless *egfp* | This study |
| pSEVA225E-PC0J56_RS00120 | promoter of *C0J56_RS00120* fused with a promoterless *egfp* | This study |
| pSEVA225E-PsspA | promoter of *sspA* fused with a promoterless *egfp* | This study |
| pSEVA225E-PC0J56_RS21180 | promoter of *C0J56_RS21180* fused with a promoterless *egfp* | This study |
| pSEVA225E-PlpxO | promoter of *lpxO* fused with a promoterless *egfp* | This study |
| pSEVA225E-PC0J56_RS11055 | promoter of *C0J56_RS11055* fused with a promoterless *egfp* | This study |
| pSEVA225E-PzipA | promoter of *zipA* fused with a promoterless *egfp* | This study |
| pSEVA225E-PcheY | promoter of *cheY* fused with a promoterless *egfp* | This study |
| pSEVA225E-PftsH | promoter of *ftsH* fused with a promoterless *egfp* | This study |
| pSEVA225E-PgacA | promoter of *gacA* fused with a promoterless *egfp* | This study |
| pSEVA225E-PC0J56_RS18475 | promoter of *C0J56_RS18475* fused with a promoterless *egfp* | This study |
| pSEVA225E-PC0J56_RS17670 | promoter of *C0J56_RS17670* fused with a promoterless *egfp* | This study |
| pSEVA225E-PnarX | promoter of *narX* fused with a promoterless *egfp* | This study |
| pSEVA225E-PC0J56_RS12215 | promoter of *C0J56_RS12215* fused with a promoterless *egfp* | This study |
| pSEVA225E-PntrC1 | promoter of *ntrC*1 fused with a promoterless *egfp* | This study |
| pSEVA225E-PC0J56_RS07595 | promoter of *C0J56_RS07595* fused with a promoterless *egfp* | This study |
| pSEVA225E-PC0J56_RS06300 | promoter of *C0J56_RS06300* fused with a promoterless *egfp* | This study |
| pSEVA225E-PiscR | promoter of *iscR* fused with a promoterless *egfp* | This study |

**Table S2. Primers used in this study**

| Primers | Sequence (5’ to 3’) | Purpose |
| --- | --- | --- |
| mupR-F1 | CCGGAATTCGGCGGTTTTTCCGACAAAGA | Construction of markerless Δ*mupR* mutant |
| mupR-R1 | CATGTCACCAACTTTATGCG |  |
| mupR-F2 | CGCATAAAGTTGGTGACATGAACCGAAGAAAAGCCACAGTCCTTC |  |
| mupR-F2 | CCCAAGCTTACGGTTCACATGGGGCCCGA |  |
| mupI-F1 | AAAGAATTCCCTGAACTTTTTCTTGAGTA | Construction ofmarkerless Δ*mupI* mutant |
| mupI-R1 | CTCCAGATAGTCTGGTGAAA |  |
| mupI-F2 | TTTCACCAGACTATCTGGAGAGCTTGCTGACCACCAAAAA |  |
| mupI-R2 | AAAGGATCCGCAGCCCGCGATGAGTGTGC |  |
| pvdL-F1 | AAAGGATCCGTGATGCGTGCCGCGATCTT | Construction of markerless Δ*pvdL* mutant |
| pvdL-R1 | CACCACACCCTGCCCTGGGG |  |
| pvdL-F2 | CCCCAGGGCAGGGTGTGGTGTTCGAATGCAGCACGGTGCA |  |
| pvdL-R2 | AAATCTAGAATGGATGGAGAGCCACCATG |  |
| pvdL-v1 | ATGACCGACGCGTTCGAACT | PCR verification of the Δ*pvdL*  mutant |
| pvdL-v2 | TGATCGACAGCAAACGCTCC |  |
| pvdL-v3 | AACTCGACCGCAAGGCCTTG |  |
| pvdL-v4 | CTACAATCCTTCCAATTCCG |  |
| pvdDJI-F1 | AAAGAATTCATGACGAGTGCCTTGAACAG | Construction of markerless Δ*pvdDJI* mutant |
| pvdDJI-R1 | CAGTTGGAGAAAATGGCCCA |  |
| pvdDJI-F2 | TGGGCCATTTTCTCCAACTGGATGGGAAAACCGCTGAACT |  |
| pvdDJI-R2 | AAAAAGCTTGCACGAGGCCAAGGTGCTGC |  |
| pvdDJI-v1 | TCAGTTGCCTTGCGCAACCT | PCR verification of the Δ*pvdDJI* mutant |
| pvdDJI-v2 | ATGAATGCTGAAGACGCCTT |  |
| pvdDJI-v3 | GAGCCGCACCGCTCCCGGCA |  |
| pvdDJI-v4 | CTACAATCCTTCCAATTCCG |  |
| mmpD-F1 | AAATCTAGAACAGCAGCTTGATGATCTGC | Construction of markerless Δ*mmpD* mutant |
| mmpD-R1 | GCGACCATTCGCGCCCTGGC |  |
| mmpD-F2 | GCCAGGGCGCGAATGGTCGCTATTCGCGCACTGGCCGGCG |  |
| mmpD-R2 | AAAAAGCTTCTTTCACGCCATGCTGATGC |  |
| mmpD-v1 | ATGGACCCCCAGCAACGGAT | PCR verification of the Δ*mmpD*  mutant |
| mmpD-v2 | TCATTGGGTTGCAGCCTCGT |  |
| mmpD-v3 | ATGGTGGATGGCGCTCAGTG |  |
| mmpD-v4 | ATTGACCGTCTCGACCGCTT |  |
| glpD-F1 | AAATCTAGAGTCATATTTCCCGTTGTCCT | Construction of markerless Δ*glpD* mutant |
| glpD-R1 | AATGACGGCGATGTCGTAGA |  |
| glpD-F2 | TCTACGACATCGCCGTCATTAAGCTGCAACAGTATCTGGA |  |
| glpD-R1 | AAAAAGCTTTACTTCGAAGTGCCTGGTAG |  |
| gap1-F1 | AAAGAATTCGCTGTCGGCGGTCAGCAGCA | Construction of markerless Δ*gap*1 mutant |
| gap1-R1 | GCCATAGATCGAGCTCACCA |  |
| gap1-F2 | TGGTGAGCTCGATCTATGGCCAGGTGGTTCGCGTGATGGA |  |
| gap1-R2 | AAAAAGCTTGGTCGAGGTGCGCACCACCG |  |
| gltB-F1 | AAATCTAGATGTCGCCAGCGTCCAACAGG | Construction of markerless Δ*gltB* mutant |
| gltB-R1 | TATCAGGCCGAAACCGCAGT |  |
| gltB-F2 | ACTGCGGTTTCGGCCTGATAAAGCCCAAGGCTGCCAACCT |  |
| gltB-R2 | AAAAAGCTTCGCGCCGAAACCGTCGTTGA |  |
| glnA1-F1 | AAAGAATTCGAAAAACGCTTCCACGGTTT | Construction of markerless Δ*glnA*1 mutant |
| glnA1-R1 | GAAATCAAGGTTCGCACCTT |  |
| glnA1-F2 | AAGGTGCGAACCTTGATTTCGAAGCGCAGATCAATCCACT |  |
| glnA1-R2 | AAAAAGCTTCATCCGTACCGGGCGGCTCT |  |
| mupR-C1 | AAAGGTACCACCATTGATCAGCTCATTTA | *MupR* complementation |
| mupR-C2 | AAAAAGCTTTCAGGGCGTGACCAGGCCTG |  |
| mupI-C1 | AAAGGTACCACACACCAAATCCCAGTCAG | *MupI* complementation |
| mupI-C2 | AAAAAGCTTTTAACCACCGTTGACGGCGT |  |
| mupRFLAG1 | ATGGACTACAAAGACCATGACGGTGATTATAAAGATCATGACATCGATTACAAGGATGACGATGACAAG | N terminal 3xFLAG fused mupR  complementation |
| MupRFLAG2 | ACTACCGCCACCGCCAGAGCCACCTCCGCCTGAACCGCCTCCACCCTTGTCATCGTCATCCTTGT |  |
| pBBR5-F1 | TCATGGTCTTTGTAGTCCATTTTAACCTCCTTGTCGTTGT |  |
| pBBR5-R1 | GCTCTGGCGGTGGCGGTAGTATGCTGGAAGAAATTCTGAT |  |
| pBBR5-F2 | CCGCCTGAACCGCCTCCACCGGGCGTGACCAGGCCTGAAC | Construction of pBBR5-3 x FLAG |
| pBBR5-R2 | ACAAGGATGACGATGACAAGGATATCGAATTCACTAGTGA |  |
| R-mupR1 | AAAGAATTCACCATTGATCAGCTCATTTAAT | Construction of *egfp* based reporter |
| R-mupR2 | AAAAAGCTTTTTAACCTCCTTGTCGTTGTG |  |
| R-mupI1 | AAAGAATTCACACACCAAATCCCAGTCAG | Construction of *egfp* based reporter |
| R-mupI2 | AAAAAGCTTGTCGACATCCTCGGCTTCGA |  |
| R-mmpB1 | AAAGGATCCAGGCAGTCGTCTCACAAGGG | Construction of *egfp* based reporter |
| R-mmpB2 | AAAAAGCTTGTTCAGTATCGACCCTTGGA |  |
| R-mupZ1 | AAAGAATTCCTACTGATCTCTTGAACTGA | Construction of *egfp* based reporter |
| R-mupZ2 | AAAAAGCTTCTTTCCACCGTTGCGTTAGA |  |
| R-mupF1 | AAAGAATTCTTATCTATCCTTTAGCCGGC | Construction of *egfp* based reporter |
| R-mupF2 | AAAAAGCTTCGATAGCGATCCTTGGGTGT |  |
| R-mupO1 | AAAGAATTCATTTACCTATCAGTGCTTAT | Construction of *egfp* based reporter |
| R-mupO2 | AAAAAGCTTCGCTGCTCTCCCGAATGGCG |  |
| R-macpE1 | AAAGAATTCCCCCTGTCCGGCAAGTATTG | Construction of *egfp* based reporter |
| R-macpE2 | AAAAAGCTTCTGGGCCTCCTATGGATGCC |  |
| R-PmacpC1 | AAAGAATTCCGATAGCGATCCTTGGGTGT | Construction of *egfp* based reporter |
| R-PmacpC2 | AAAAAGCTTTTATCTATCCTTTAGCCGGC |  |
| R-phlD1 | AAAGAATTCCAAACTTAGACGCCGCCATA | Construction of *egfp* based reporter |
| R-phlD2 | AAAAAGCTTGACAAGTCCTCGGCTAAAAG |  |
| R-phlF1 | AAAGAATTCGTGTACATCCTCCAGATTCC | Construction of *egfp* based reporter |
| R-phlF2 | AAAAAGCTTAATTCAAAATCTCCAATATG |  |
| R-phlE1 | AAAGAATTCCAGACCGGATAAAACACCAG | Construction of *egfp* based reporter |
| R-phlE2 | AAAAAGCTTGTTCTTTCCCCTTCTTTCCC |  |
| R-phlA1 | AAAGAATTCAATTCAAAATCTCCAATATG | Construction of *egfp* based reporter |
| R-phlA2 | AAAAAGCTTGTGTACATCCTCCAGATTCC |  |
| R-glpD-1 | AAAGGATCCACCTGAGCAAGCACCGGCGA | Construction of *egfp* based reporter |
| R-glpD-2 | AAAAAGCTTCGGCTGGCCTCGGGCTCTTT |  |
| R-sucA-1 | AAAGAATTCTTCAGCTGCTGTACCCGTAG | Construction of *egfp* based reporter |
| R-sucA-2 | AAAAAGCTTGCTTGGTCACCCTCGATTAG |  |
| R-fadD-1 | AAAGAATTCCCTGAGTTGGTATCGGAACA | Construction of *egfp* based reporter |
| R-fadD-2 | AAAAAGCTTTTACCAACCTCTCTCAATCC |  |
| R-RS10665-1 | AAAGAATTCATAGCTTGATGGCCTTTACC | Construction of *egfp* based reporter |
| R-RS10665-2 | AAAAAGCTTATGAGTTCCACCAATGGAAT |  |
| R-RS01565-1 | AAAGAATTCCGACAGAACTCTCGAACAAT | Construction of *egfp* based reporter |
| R-RS01565-2 | AAAAAGCTTCTGCGTAAACCTGTGTCCCG |  |
| R-RS00995-1 | AAAGGATCCCGCTCACTGCTTCACCACTA | Construction of *egfp* based reporter |
| R-RS00995-2 | AAAAAGCTTGAAGGATTATCTCGGGCTGG |  |
| R-RS19720-1 | AAAGAATTCTTTTTAAGGCTCCTGTTTTC | Construction of *egfp* based reporter |
| R-RS19720-2 | AAAAAGCTTGAGAGGGCCTTCTGGTTATC |  |
| R-RS21195-1 | AAAGAATTCAAGGGTTTCTCACTTTCTTT | Construction of *egfp* based reporter |
| R-RS21195-2 | AAAAAGCTTATACCCATCAGCGCTCTATT |  |
| R-pycA-1 | AAAGAATTCTTTCTAAGCACTCTCGACCG | Construction of *egfp* based reporter |
| R-pycA-2 | AAAAAGCTTTTGAAGGTTCCCTTGAGCCG |  |
| R-gltA-1 | AAAGAATTCGATGGCCAGGCTGACGAAGA | Construction of *egfp* based reporter |
| R-gltA-1 | AAAAAGCTTGTGGCCTCCTATTAATGCTT |  |
| R-glnA1-1 | AAAGAATTCGAAAAAAAAGCGCGCGAACG | Construction of *egfp* based reporter |
| R-glnA1-2 | AAAAAGCTTATGTCCTCCGGGTGGCTTCG |  |
| R-gap1-1 | AAAGAATTCGGAAGGTTGAGCTCCAGCGT | Construction of *egfp* based reporter |
| R-gap1-2 | AAAAAGCTTCGCTTCTGCGAGAGCTTCAC |  |
| R-gacA-1 | AAAGAATTCAGCCTCGGATATCGCGCCGA | Construction of *egfp* based reporter |
| R-gacA-2 | AAAAAGCTTTCGTGTAATGCCCGTACGGA |  |
| R-gltB-1 | AAAGAATTCAGATTCGGCGGCCTTACCCA | Construction of *egfp* based reporter |
| R-gltB-2 | AAAAAGCTTAGACACTTTCTCACCAGGCT |  |
| R-aceE-1 | AAAGAATTCGGTCTATCCTTGATCGGCGC | Construction of *egfp* based reporter |
| R-aceE-2 | AAAAAGCTTGGCTTGCTCCAGGGCGGAAA |  |
| R-fabD-1 | AAAGAATTCGCGTTTTCGTCCGACAATGC | Construction of *egfp* based reporter |
| R-fabD-1 | AAAAAGCTTGAAAACAAAGCCCCTAATGA |  |
| R-gltS-1 | AAAGGATCCCGATCAAAACTTCGTTTCAA | Construction of *egfp* based reporter |
| R-gltS-2 | CCCAAGCTTGAGTGTTTATCTTTTTATAT |  |
| R-wbpA-1 | AAAGGATCCCCGGGAAACAAGGGGGGCGA | Construction of *egfp* based reporter |
| R-wbpA-2 | CCCAAGCTTCAACCAAAACACTCCCAATG |  |
| R-wbmI-1 | AAAGAATTCTTCCCTTCGTGGAAGTCGAG | Construction of *egfp* based reporter |
| R-wbmI-2 | CCCAAGCTTCAACTCTGGCGTTCCTGAAA |  |
| R-RS25795-1 | AAAGAATTCCGCTCTCTCCTGGGTTGATT | Construction of *egfp* based reporter |
| R-RS25795-2 | CCCAAGCTTGAGCCACTCCAATGGGACAA |  |
| R-ilvB-1 | AAAGAATTCGCTGTTTCCTTGTCATGGGT | Construction of *egfp* based reporter |
| R-ilvB-2 | CCCAAGCTTCTCACCGCCAGATAAAAGCT |  |
| R-cyoA-1 | AAAGAATTCACCGCCTCGATGAAACGCCC | Construction of *egfp* based reporter |
| R-cyoA-2 | CCCAAGCTTGTTGCAGCCTCCCAGCAACA |  |
| R-sspA-1 | AAAGAATTCTTTCGCTTCCGCTTTCAAAA | Construction of *egfp* based reporter |
| R-sspA-2 | CCCAAGCTTTCAGTCCTCTCCCATCAGCT |  |
| R-algD-1 | AAAGAATTCGGCATAACCTTATATCGCCT | Construction of *egfp* based reporter |
| R-algD-2 | CCCTCTAGACGCATTTACCTCTGTTTTTC |  |
| R-sdhC-1 | AAAGAATTCGTGGCCTCCTATTAATGCTT | Construction of *egfp* based reporter |
| R-sdhC-2 | CCCAAGCTTGATGGCCAGGCTGACGAAGA |  |
| R-RS21180-1 | AAAGAATTCCAGCGGCAACTCGCGGGGAC | Construction of *egfp* based reporter |
| R-RS21180-2 | CCCAAGCTTGGCATGGACCTCACACTCGT |  |
| R-acpP-1 | AAAGAATTCATGTGACGGATTGCTTCAAA | Construction of *egfp* based reporter |
| R-acpP-2 | CCCTCTAGAACCTTGTTTTCACTCCTAAT |  |
| R-RS19015-1 | AAAGGATCCAACGCTCATATTCGCGATCC | Construction of *egfp* based reporter |
| R-RS19015-2 | CCCAAGCTTGCTGGAAATCCTTAACAGAG |  |
| R-RS18475-1 | AAAGAATTCGAACATGACTCCCTTTATTG | Construction of *egfp* based reporter |
| R-RS18475-2 | CCCAAGCTTTATTATCCGACCACGTGTTT |  |
| R-RS17670-1 | AAAGAATTCTGTTGTTTTCCTCGTCGGTA | Construction of *egfp* based reporter |
| R-RS17670-2 | CCCAAGCTTAATGCTTATTTACCTCTGGC |  |
| R-RS16940-1 | AAAGAATTCGTCGCTCCATGACTACTGCC | Construction of *egfp* based reporter |
| R-RS16940-2 | CCCAAGCTTGAGAATCTCCTAACCAAGTC |  |
| R-RS16615-1 | AAAGAATTCGACAAACCTCCCGGGCAAGG | Construction of *egfp* based reporter |
| R-RS16615-2 | CCCAAGCTTGACGAATGATCCCGTTGTGT |  |
| R-lpxO-1 | AAAGAATTCAGGTTTATCCTGCGGAACAT | Construction of *egfp* based reporter |
| R-lpxO-2 | CCCAAGCTTGGAATCCGTCACAATGCAGG |  |
| R-narX-1 | AAAGAATTCCACGATGCACTCCGTCAGTT | Construction of *egfp* based reporter |
| R-narX-2 | CCCTCTAGATGACGCGGGCCTGTGGGGCA |  |
| R-acnB-1 | AAAGAATTCAGGAAAAAGCACACGCAAAA | Construction of *egfp* based reporter |
| R-acnB-2 | CCCAAGCTTGCGCGTCGGAGGCTTTTTGA |  |
| R-hcnA-1 | AAAGAATTCGGGCGGTACCGTGTGGGGAA | Construction of *egfp* based reporter |
| R-hcnA-2 | CCCAAGCTTACTGCTCTTCCTTGAAGGTT |  |
| R-RS12215-1 | AAAGAATTCCCGCCGCCGATCTATTGACG | Construction of *egfp* based reporter |
| R-RS12215-2 | CCCAAGCTTAATGATTACTCCAATCAAAA |  |
| R-RS11055-1 | AAAGAATTCGCATTCTTGAATCCGGGTCT | Construction of *egfp* based reporter |
| R-RS11055-2 | CCCAAGCTTTTCATTCACCTCGTTTAGTT |  |
| R-zipA-1 | AAAGAATTCGGCACGTGTTGTAGGGCATT | Construction of *egfp* based reporter |
| R-zipA-2 | CCCAAGCTTGTAATCCCGTGCCTCTATAA |  |
| R-cheY-1 | AAAGAATTCGGCCTGCTCAGGATTATTGC | Construction of *egfp* based reporter |
| R-cheY-2 | CCCAAGCTTTCTTGCTTCTCTCGCCTACA |  |
| R-ntrC1-1 | AAAGAATTCTTTAAGTTAATCCGAGCTCG | Construction of *egfp* based reporter |
| R-ntrC1-2 | CCCAAGCTTTAACAAAACCGGAAGGAACT |  |
| R-RS07595-1 | AAAGAATTCAGCCGGCTGGCCGCCTGTTT | Construction of *egfp* based reporter |
| R-RS07595-2 | CCCAAGCTTGATGGTTGACGGCTTCCTGA |  |
| R-RS06300-1 | AAAGAATTCGTACGGTGACTCCTGGGGAA | Construction of *egfp* based reporter |
| R-RS06300-2 | CCCAAGCTTACTCTCTTGCTGAGCGACGG |  |
| R-iscR-1 | AAAGAATTCACTGAGGCGCCTGCATCGCG | Construction of *egfp* based reporter |
| R-iscR-2 | CCCTCTAGAGGCGTATCGGCCTTTTGTAG |  |
| R-ftsH-1 | AAAGAATTCGAGGTTTTTCGACCGGGGCG | Construction of *egfp* based reporter |
| R-ftsH-2 | CCCAAGCTTTTAACTACCCTCTGAAGCAA |  |
| R-gbpA-1 | AAAGAATTCTCGCAACCCTGCGTCACCCA | Construction of *egfp* based reporter |
| R-gbpA-2 | CCCAAGCTTATAAATTCCAGACTAGTCAT |  |
| R-RS00200-1 | AAAGAATTCGGGATTTCCGATGTGGGCGG | Construction of *egfp* based reporter |
| R-RS00200-2 | CCCAAGCTTAAAACGACCTTTTCAGGGTT |  |
| R-RS00120-1 | AAAGAATTCCGTAACCCCCAACAACAAAT | Construction of *egfp* based reporter |
| R-RS00120-2 | CCCAAGCTTTTTTCACGCCCTGCATTTAG |  |
| q-rpoD1 | CGCCAAGAAGTACACCAACC | RT-qPCR |
| q-rpoD2 | GCGACGGTATTCGAACTTGT |  |
| q-mupZ1 | GGAAATCTATCGCGATGCCC |  |
| q-mupZ2 | AGAACTGCGGCTTCATGAAC |  |
| q-mmpB1 | GGTGTACCTCGAAATGGCAC |  |
| q-mmpB2 | CTACGCCAGGCAAAGAACTC |  |
| q-mupF1 | ACATTTGTATTACCGGCGCC |  |
| q-mupF2 | GGACGGGCATTATTGGTGAC |  |
| q-macpC1 | TGAAACATACCCGCGAAACC |  |
| q-macpC2 | GGCCAGTTCACCGATATTGC |  |
| q-mupO1 | CGTGATGAAGTGGATGCCTG |  |
| q-mupO2 | GGAACTCGAAGTCTTTGGCC |  |
| q-macpE1 | TCACTCATCAGACCGTCCAG |  |
| q-macpE2 | TACTGCAACCGTAGAGACCC |  |
| q-mupR1 | GAAACCCACGATCAGTACGC |  |
| q-mupR2 | CGCCTCATACAATTGCAGCT |  |
| q-mupI1 | AGACTATCTGGAGGGCATGC |  |
| q-mupI2 | AACATGTAAGGACCGGTCGT |  |
| q-phlF1 | GAGCCCTCACACGCATAAAG |  |
| q-phlF2 | TGCTCGCTTTCACTCTCGTA |  |
| q-phlA1 | GATTGGGCTCGGACAAGAAC |  |
| q-phlA2 | CGATTTGGGCCTTCGTGAAT | RT-qPCR |
| q-phlD1 | AATCGAGCCAATGACTTCGC |  |
| q-phlD2 | CTCGCTATCAGGCAGGAAGT |  |
| q-phlE1 | GAAAGACGCATGATCACGCT |  |
| q-phlE2 | GGAGGGATAGCAGGGTGATC |  |
| q-pycA-1 | TGAAGAACTCGAACAGGCCT |  |
| q-pycA-2 | CAGTCACGCTCGAACAGATG |  |
| q-fadD-1 | GACCAACACCCTGCTCAATC |  |
| q-fadD-2 | TTCGGTCAACCCATAGCCTT |  |
| q-gltB-1 | AGTGCTGCAAATCAAGGTGG |  |
| q-gltB2 | CGAAGATCAGTTGCGACAGG |  |
| q-glpD-1 | GCCAAGTTCATTCGTGACGA |  |
| q-glpD-2 | CGGTGTATTCGCGATCAGTC |  |
| q-gltA-1 | CCGTTTCACCTTTGACCCAG |  |
| q-gltA-2 | AGGTCTTCAACTGCTCGTGA |  |
| q-aceE-1 | ATGACCGAAGAGCAACAGGA |  |
| q-aceE-2 | CAGGATCTTCGCTGCTTCAC |  |
| q-gap1-1 | ACTCCTACACCAACGACCAG |  |
| q-gap1-2 | GGTTGAGAATGGCCATCGAC |  |
| q-glnA1-1 | GTGAAGTTCAACACGCTGGT |  |
| q-glnA1-2 | GTGAAGTTCAACACGCTGGT |  |
| q-sucA-1 | CGATTTCCATCCACGGTGAC |  |
| q-sucA-2 | CTGGATCATTTTCGCGACGT |  |
| q-RS01565-1 | GCTCCAACGACGACATCTTC |  |
| q-RS01565-2 | GGATGTTCTCGTGGATGTGC |  |
| q-fabD-1 | CGCATTCGTCTTTCCAGGAC |  |
| q-fabD-2 | TTGTCGGTTTGATTGAGGCG |  |
| q-RS10665-1 | CCCATCAAGATTTGCGTCGT |  |
| q-RS10665-2 | AGGGTGATCAGAGCGAAGTC |  |
| q-gacA-1 | CTGCGAAGAAGATCCGTTCC |  |
| q-gacA-2 | AACCGACAATCATCAAGGCG |  |
| q-RS00995-1 | GATGTTCATCGACCAGCACC |  |
| q-RS00995-2 | ATGTAAATGTCGTGCAGCCC |  |
| q-RS19720-1 | CTACGGTGTCATGCTGGTTG |  |
| q-RS19720-2 | AAACTGACCGGGTTCTCGAT |  |
| q-RS21195-1 | AGACGACATGAGCTGGGAAA |  |
| q-RS21195-2 | GATGAACGATGTCCAAGCCC |  |
| q-gltS-1 | TCTCATCAAGCGTGTCCAGT |  |
| q-gltS-2 | GAGCTGACCATTCAAGAGCG |  |
| q-wbpA-1 | GAGCGAGAAGATCCGGGTAA |  |
| q-wbpA-2 | CAACTTCGTCATCTCGGCAG |  |
| q-wbmI-1 | GTTACATGGCGGCTTACCTG |  |
| q-wbmI-2 | TCACTGCGATCAATCAAGCG |  |
| q-RS25795-1 | TACTTTACGTACGACCCGGG |  |
| q-RS25795-2 | CAAGCGACTGAGTTCACTGG | RT-qPCR |
| q-ilvB-1 | GGTGTGTTGGGTATGGTTCG |  |
| q-ilvB-2 | CGTCTTTGATCTGCATCGGG |  |
| q-cyoA-1 | GAAACCTGATCATCACCGCC |  |
| q-cyoA-2 | CGTAACCCAGGGCAATGATG |  |
| q-sspA-1 | ATCGTACTGGCAGAGAAGGG |  |
| q-sspA-2 | TCCATCACCACAGTCGACTC |  |
| q-algD-1 | AACGTTGTCATCCCGATCCT |  |
| q-algD-2 | GAGGCTTTGTCGAACTCACC |  |
| q-sdhC-1 | CAAGTCGCTCGATTCGGAAG |  |
| q-sdhC-2 | AGACTCCTGCCAGAACGATC |  |
| q-RS21180-1 | TTAGCAGGCGACGTTGCCAT |  |
| q-RS21180-2 | TCGATCAGCAAGTTCGCCAA |  |
| q-acpP-1 | CGCGTCAAGAAAATCGTTGC |  |
| q-acpP-2 | TCGTCAGGAATCTCGGTCTC |  |
| q-RS19015-1 | GTTGGCCTGTGTGCTTTCAT |  |
| q-RS19015-2 | AGCATCGCGACCAGATAGAA |  |
| q-RS18475-1 | CTCAACGTGCTGAACCTGAC |  |
| q-RS18475-2 | CCGCTCAATGACGTTTTCCA |  |
| q-RS17670-1 | CCACTACGACAGCTTTTCCG |  |
| q-RS17670-2 | TTTCAAGTCCACGCCCAATG |  |
| q-RS16940-1 | CAGTTACGGACAGTGGTTGC |  |
| q-RS16940-2 | CTTTTCCGGGAAGTGTTGGG |  |
| q-RS16615-1 | GGGAGACTACAGCATCGTCA |  |
| q-RS16615-2 | ACTTTCATCACACCATCGCG |  |
| q-lpxO-1 | CATCATCCTGTTCTGCGACG |  |
| q-lpxO-2 | AAAAGGCCCATTTCTGCAGG |  |
| q-narX-1 | GCACCATGATCATCCTGCTG |  |
| q-narX-2 | GGTTCAGGGTCTTTTGCTGG |  |
| q-acnB-1 | TGGTTCGTTTCAAGGGCAAG |  |
| q-acnB-2 | TCCTTGGACAGCTTGATGGT |  |
| q-hcnA-1 | TGAAACCGTCCTCACTGTCA |  |
| q-hcnA-2 | GTTTCGATCTGCATGCCTGG |  |
| q-RS12215-1 | ATCGACCAAACCCACCAAGA |  |
| q-RS12215-2 | ACTACGCAACCAGGAATCGA |  |
| q-RS11055-1 | CAATCCTGAGCGCGCTTC |  |
| q-RS11055-2 | CGGCTGTTTTCACCACCTTT |  |
| q-zipA-1 | TTTCCCGGACGAGAACAAGA |  |
| q-zipA-2 | GCCATGGAGAACAACACCTC |  |
| q-cheY-1 | GCTCCCTATGTTGCACAGTG |  |
| q-cheY-2 | GACCACATAGCCGTTGACAC |  |
| q-ntrC1-1 | CCACGCACAAGAATCTCGAG |  |
| q-ntrC1-2 | GAGTTGAAGCGGATCGAACC |  |
| q-RS07595-1 | CACGCCGACACTGGATAAAG |  |
| q-RS07595-2 | AGCAGGCCTTCTTCATTCCT |  |
| q-RS06300-1 | ACACTGCCAATATCGTGCAC |  |
| q-RS06300-2 | CTCATGGGTCAGTTCCTCCA |  |
| q-iscR-1 | CCTGTCTTACCTTGAGCAGC |  |
| q-iscR-2 | AAATTCGTGGATCTGCAGGC |  |
| q-ftsH-1 | AAAGCAGAACACCGCGTATC |  |
| q-ftsH-2 | CTGATCAATGCACGCTTGGA |  |
| q-gbpA-1 | GCCCTGTCGACGGTAAAATC |  |
| q-gbpA-2 | GAGCACTTCGTTCGGATTCC |  |
| q-RS00200-1 | GTGTTTGTGGTGCCTGTCAT |  |
| q-RS00200-2 | CACTGGATGATGGCTCGGTA |  |
| q-RS00120-1 | GCCTTGGTCTCTTCCTTCCT |  |
| q-RS00120-2 | CGCCAAGATGAGACTTCTGC |  |

**Table S3. List of all regions of the genome significantly enriched for MupR binding as determined by ChIP-seq**

**Sample 1 vs input**

| geneID  (target genes) | Gene name | Position | Gene description | Fold_enrichment | -log10(pvalue) |
| --- | --- | --- | --- | --- | --- |
| C0J56_RS00005 | dnaA | inside | chromosomal replication initiator protein DnaA | 1.36462 | 11.70295 |
| C0J56_RS00120 | C0J56_RS00120 | upstream | ATPase | 7.79764 | 1636.60474 |
| C0J56_RS00200 | C0J56_RS00200 | upstream | Cytochrome c5 | 1.84771 | 51.37854 |
| C0J56_RS00210 | C0J56_RS00210 | upstream | thiol:disulfide interchange protein DsbA | 1.32624 | 9.17241 |
| C0J56_RS00430 | C0J56_RS00430 | inside | EamA family transporter | 10.54226 | 2713.5603 |
| C0J56_RS00470 | C0J56_RS00470 | upstream | RNA-directed DNA polymerase | 3.10484 | 251.54431 |
| C0J56_RS00595 | C0J56_RS00595 | upstream | Uncharacterized protein | 6.0308 | 1031.28577 |
| C0J56_RS00625 | C0J56_RS00625 | upstream | LysR family transcriptional regulator | 2.27166 | 105.75438 |
| C0J56_RS00775 | aspA | inside | aspartate ammonia-lyase | 1.7938 | 45.62275 |
| C0J56_RS00985 | C0J56_RS00985 | upstream | Uncharacterized protein | 11.42936 | 3090.38623 |
| C0J56_RS00995 | C0J56_RS00995 | upstream | Succinate CoA transferase | 2.10257 | 82.22563 |
| C0J56_RS01035 | C0J56_RS01035 | upstream | 16S ribosomal RNA | 17.27881 | 5846.3208 |
| C0J56_RS01275 | C0J56_RS01275 | upstream | Heme oxygenase-like protein | 2.92841 | 216.95053 |
| C0J56_RS01315 | C0J56_RS01315 | upstream | LapA family giant adhesin | 1.64677 | 19.29645 |
| C0J56_RS01315 | C0J56_RS01315 | inside | LapA family giant adhesin | 1.45134 | 31.40657 |
| C0J56_RS01565 | serA | upstream | D-3-phosphoglycerate dehydrogenase | 1.98004 | 66.67026 |
| C0J56_RS01620 | C0J56_RS01620 | upstream | Uncharacterized protein | 2.6711 | 169.90849 |
| C0J56_RS02470 | C0J56_RS02470 | inside | malate dehydrogenase | 1.3625 | 10.68046 |
| C0J56_RS02540 | gbpA | upstream | GlcNAc-binding protein A | 2.70295 | 175.50278 |
| C0J56_RS02615 | C0J56_RS02615 | upstream | Uncharacterized protein | 3.96254 | 443.40283 |
| C0J56_RS02620 | C0J56_RS02620 | inside | lipoprotein | 2.08297 | 79.65016 |
| C0J56_RS02625 | C0J56_RS02625 | upstream | Uncharacterized protein | 4.82023 | 668.41724 |
| C0J56_RS02830 | C0J56_RS02830 | upstream | acyl-CoA dehydrogenase | 1.48406 | 18.87378 |
| C0J56_RS02835 | putA | inside | bifunctional protein PutA | 1.84409 | 54.37059 |
| C0J56_RS02840 | putP | inside | sodium:proline symporter | 1.30009 | 7.69692 |
| C0J56_RS02845 | C0J56_RS02845 | upstream | Uncharacterized protein | 2.45545 | 133.8508 |
| C0J56_RS03210 | hflX | inside | GTPase HflX | 1.83498 | 51.79222 |
| C0J56_RS03245 | C0J56_RS03245 | upstream | tRNA-Leu | 1.86242 | 52.99729 |
| C0J56_RS03280 | C0J56_RS03280 | inside | Uncharacterized protein | 2.18071 | 100.07008 |
| C0J56_RS03340 | azu | upstream | Azurin | 4.09009 | 485.70169 |
| C0J56_RS03640 | C0J56_RS03640 | upstream | Toxin A | 2.57429 | 160.9438 |
| C0J56_RS03645 | C0J56_RS03645 | upstream | UPF0056 inner membrane protein | 5.00402 | 720.33594 |
| C0J56_RS03980 | C0J56_RS03980 | upstream | 16S ribosomal RNA | 18.63152 | 54.86218 |
| C0J56_RS04190 | C0J56_RS04190 | upstream | proline iminopeptidase | 1.63552 | 6539.02881 |
| C0J56_RS04220 | C0J56_RS04220 | inside | addiction module antidote protein, HigA family | 1.66592 | 31.92467 |
| C0J56_RS04375 | ssrA | upstream | transfer-messenger RNA | 2.63434 | 33.33308 |
| C0J56_RS04415 | smpB | inside | SsrA-binding protein | 1.6531 | 163.53722 |
| C0J56_RS04435 | C0J56_RS04435 | upstream | outer membrane protein assembly factor BamE | 1.87075 | 34.7101 |
| C0J56_RS04470 | carA | upstream | carbamoyl-phosphate synthase small chain | 3.7312 | 56.8353 |
| C0J56_RS04500 | ftsH | upstream | ATP-dependent zinc metalloprotease FtsH | 1.47224 | 417.38702 |
| C0J56_RS04505 | folP | upstream | dihydropteroate synthase | 1.3228 | 18.8877 |
| C0J56_RS04525 | C0J56_RS04525 | upstream | tRNA-Leu | 3.88962 | 10.99007 |
| C0J56_RS04545 | infB | inside | translation initiation factor IF-2 | 1.46918 | 455.56705 |
| C0J56_RS04735 | C0J56_RS04735 | inside | ABC transporter substrate-binding protein | 1.43023 | 21.78041 |
| C0J56_RS04785 | C0J56_RS04785 | upstream | Uncharacterized protein | 2.10733 | 16.27888 |
| C0J56_RS04860 | mreC | upstream | rod shape-determining protein MreC | 2.083 | 90.03298 |
| C0J56_RS04940 | ptsN | inside | nitrogen regulatory protein | 1.47513 | 91.83312 |
| C0J56_RS05010 | murA | inside | UDP-N-acetylglucosamine 1-carboxyvinyltransferase | 1.24913 | 20.17312 |
| C0J56_RS05060 | C0J56_RS05060 | inside | acetyltransferase | 1.19765 | 6.3881 |
| C0J56_RS05175 | C0J56_RS05175 | upstream | Reverse transcriptase domain-containing protein | 12.81882 | 4.22756 |
| C0J56_RS05245 | C0J56_RS05245 | upstream | Putative exported protein | 1.84205 | 3704.91968 |
| C0J56_RS05265 | iscR | upstream | Fe-S cluster assembly transcriptional regulator IscR | 1.23636 | 58.94231 |
| C0J56_RS05310 | rlmN | inside | Dual-specificity RNA methyltransferase RlmN | 1.44535 | 5.82704 |
| C0J56_RS05400 | guaA | upstream | GMP synthase [glutamine-hydrolyzing] | 1.65602 | 19.60783 |
| C0J56_RS05510 | rimM | inside | ribosome maturation factor RimM | 3.70081 | 39.13069 |
| C0J56_RS05885 | rpsB | upstream | 30S ribosomal protein S2 | 3.71673 | 441.77493 |
| C0J56_RS05925 | bamA | upstream | outer membrane protein assembly factor BamA | 1.59524 | 465.39444 |
| C0J56_RS05930 | C0J56_RS05930 | inside | Outer membrane chaperone skp | 1.38364 | 31.01452 |
| C0J56_RS05935 | lpxD | inside | UDP-3-O-acylglucosamine N-acyltransferase | 1.57954 | 13.76847 |
| C0J56_RS06040 | rpoS | upstream | RNA polymerase sigma factor RpoS | 1.40948 | 29.38081 |
| C0J56_RS06260 | recA | inside | protein RecA | 1.5477 | 15.6291 |
| C0J56_RS06300 | C0J56_RS06300 | upstream | LysR family transcriptional regulator | 3.7451 | 28.01631 |
| C0J56_RS06405 | C0J56_RS06405 | upstream | cold shock protein CapB | 2.17504 | 495.86508 |
| C0J56_RS06455 | C0J56_RS06455 | upstream | acyltransferase | 3.35173 | 107.20139 |
| C0J56_RS06550 | C0J56_RS06550 | inside | Uncharacterized protein | 1.23906 | 356.34503 |
| C0J56_RS06685 | arcA | inside | arginine deiminase | 4.78052 | 6.22005 |
| C0J56_RS06975 | C0J56_RS06975 | upstream | superoxide dismutase | 1.82206 | 783.44171 |
| C0J56_RS07020 | C0J56_RS07020 | upstream | Uncharacterized protein | 1.50966 | 67.98892 |
| C0J56_RS07125 | C0J56_RS07125 | upstream | secretion protein HlyD | 3.36277 | 3086.43066 |
| C0J56_RS07240 | yiaY | upstream | alcohol dehydrogenase | 1.55973 | 23.8931 |
| C0J56_RS07295 | C0J56_RS07295 | upstream | Uncharacterized protein | 2.72959 | 352.42593 |
| C0J56_RS07460 | C0J56_RS07460 | inside | sigma factor AlgU negative regulatory protein | 4.12121 | 37.11505 |
| C0J56_RS07595 | C0J56_RS07595 | upstream | phosphoethanolamine transferase | 1.23741 | 188.62367 |
| C0J56_RS07705 | C0J56_RS07705 | upstream | MFS transporter | 1.67475 | 684.51666 |
| C0J56_RS07715 | pxpB | inside | 5-oxoprolinase subunit PxpB | 1.44536 | 5.73172 |
| C0J56_RS07720 | C0J56_RS07720 | inside | Biotin-dependent carboxyltransferase | 1.32034 | 45.84877 |
| C0J56_RS07755 | C0J56_RS07755 | upstream | aspartyl beta-hydroxylase | 1.36549 | 22.21167 |
| C0J56_RS07760 | C0J56_RS07760 | upstream | DMT family transporter | 1.54984 | 11.23864 |
| C0J56_RS07875 | C0J56_RS07875 | upstream | Uncharacterized protein | 8.92735 | 12.90237 |
| C0J56_RS07875 | C0J56_RS07875 | inside | Uncharacterized protein | 3.42965 | 28.39933 |
| C0J56_RS07895 | C0J56_RS07895 | upstream | IS4 family transposase | 8.87098 | 343.35419 |
| C0J56_RS07960 | C0J56_RS07960 | upstream | Uncharacterized protein | 2.12893 | 2062.08057 |
| C0J56_RS07980 | C0J56_RS07980 | upstream | transcriptional regulator | 1.52245 | 2040.22363 |
| C0J56_RS08045 | C0J56_RS08045 | inside | Uncharacterized protein | 4.30516 | 89.48648 |
| C0J56_RS08060 | C0J56_RS08060 | inside | inner membrane protein | 1.68435 | 25.45074 |
| C0J56_RS08130 | C0J56_RS08130 | inside | Putative NAD(P)H nitroreductase | 1.43289 | 587.46173 |
| C0J56_RS08135 | C0J56_RS08135 | inside | aromatic-amino-acid aminotransferase | 1.65769 | 44.52998 |
| C0J56_RS08220 | ntrC1 | upstream | sigma-54-dependent Fis family transcriptional regulator | 6.6284 | 19.20587 |
| C0J56_RS08355 | cheY | upstream | chemotaxis protein CheY | 1.89918 | 39.51108 |
| C0J56_RS08425 | C0J56_RS08425 | upstream | heme exporter protein C | 1.44928 | 1374.98645 |
| C0J56_RS08470 | C0J56_RS08470 | upstream | Uncharacterized protein | 1.85976 | 72.03616 |
| C0J56_RS08605 | gyrA | upstream | DNA gyrase subunit A | 1.34007 | 22.62531 |
| C0J56_RS08640 | C0J56_RS08640 | upstream | lipoprotein | 3.94884 | 64.26696 |
| C0J56_RS08650 | C0J56_RS08650 | inside | LapA family protein | 13.41675 | 12.38909 |
| C0J56_RS08800 | C0J56_RS08800 | upstream | methyl-accepting chemotaxis protein | 2.95015 | 555.06598 |
| C0J56_RS08820 | C0J56_RS08820 | upstream | Class I SAM-dependent methyltransferase | 1.62416 | 3977.85474 |
| C0J56_RS08855 | oprI | upstream | major outer membrane lipoprotein | 4.20164 | 263.17514 |
| C0J56_RS08860 | C0J56_RS08860 | inside | Acetyltransferase-like protein | 1.52093 | 36.93293 |
| C0J56_RS08905 | C0J56_RS08905 | inside | Efp Translation elongation factor P | 1.51314 | 583.06873 |
| C0J56_RS08960 | C0J56_RS08960 | inside | ATP-dependent RNA helicase DeaD | 1.78067 | 24.33356 |
| C0J56_RS09030 | C0J56_RS09030 | inside | hybrid sensor histidine kinase/response regulator | 2.4742 | 23.71658 |
| C0J56_RS09085 | C0J56_RS09085 | upstream | lipoprotein | 1.89259 | 54.11489 |
| C0J56_RS09120 | C0J56_RS09120 | upstream | Sensor histidine kinase | 2.28023 | 156.84132 |
| C0J56_RS09240 | C0J56_RS09240 | inside | antibiotic synthesis protein MbtH | 1.48085 | 66.27108 |
| C0J56_RS09295 | C0J56_RS09295 | upstream | porin | 3.40774 | 132.07809 |
| C0J56_RS09420 | C0J56_RS09420 | upstream | Uncharacterized protein | 2.16462 | 22.06163 |
| C0J56_RS09480 | C0J56_RS09480 | upstream | Uncharacterized protein | 1.30704 | 367.46103 |
| C0J56_RS09515 | C0J56_RS09515 | upstream | GntR family transcriptional regulator | 1.98014 | 97.37801 |
| C0J56_RS09550 | zipA | upstream | cell division protein ZipA | 1.32187 | 8.62174 |
| C0J56_RS09575 | C0J56_RS09575 | upstream | Uncharacterized protein | 1.99318 | 80.34252 |
| C0J56_RS09630 | C0J56_RS09630 | upstream | adenine phosphoribosyltransferase | 5.27968 | 9.61065 |
| C0J56_RS09660 | C0J56_RS09660 | inside | membrane protein | 2.93403 | 73.57399 |
| C0J56_RS09705 | C0J56_RS09705 | inside | cytochrome oxidase | 4.10386 | 1074.47705 |
| C0J56_RS09825 | C0J56_RS09825 | inside | Uncharacterized protein | 1.7075 | 260.73456 |
| C0J56_RS09870 | C0J56_RS09870 | inside | MFS transporter | 1.37879 | 668.10333 |
| C0J56_RS09930 | C0J56_RS09930 | upstream | chromosome segregation ATPase | 1.76144 | 42.90434 |
| C0J56_RS09945 | C0J56_RS09945 | inside | fused response regulator/phosphatase | 1.29206 | 14.56693 |
| C0J56_RS09950 | C0J56_RS09950 | inside | anti-anti-sigma factor | 1.45867 | 47.21013 |
| C0J56_RS09970 | C0J56_RS09970 | upstream | Uncharacterized protein | 7.28548 | 8.67008 |
| C0J56_RS10025 | C0J56_RS10025 | inside | HNH endonuclease | 13.16434 | 17.16953 |
| C0J56_RS10210 | C0J56_RS10210 | inside | Abasic site processing protein | 6.18518 | 1453.02734 |
| C0J56_RS10225 | C0J56_RS10225 | upstream | Uncharacterized protein | 12.74775 | 3862.04272 |
| C0J56_RS10325 | C0J56_RS10325 | upstream | Uncharacterized protein | 2.07163 | 1080.86243 |
| C0J56_RS10440 | C0J56_RS10440 | upstream | tRNA-Arg | 5.75389 | 3672.80298 |
| C0J56_RS10480 | C0J56_RS10480 | upstream | DNA-binding protein HU-beta | 4.18549 | 87.22434 |
| C0J56_RS10500 | C0J56_RS10500 | inside | AraC family transcriptional regulator | 1.41829 | 944.13165 |
| C0J56_RS10665 | C0J56_RS10665 | upstream | oxaloacetate decarboxylase | 4.49956 | 502.1955 |
| C0J56_RS10935 | C0J56_RS10935 | upstream | Uncharacterized protein | 5.00374 | 14.99774 |
| C0J56_RS11010 | C0J56_RS11010 | inside | acetyl-CoA acetyltransferase | 1.84684 | 618.63782 |
| C0J56_RS11055 | C0J56_RS11055 | upstream | General stress protein | 2.19698 | 942.97937 |
| C0J56_RS11235 | C0J56_RS11235 | upstream | transcriptional regulator | 4.11803 | 57.84609 |
| C0J56_RS11305 | C0J56_RS11305 | inside | cobalamin biosynthesis protein CbiG | 1.72179 | 99.02349 |
| C0J56_RS11480 | C0J56_RS11480 | inside | GNAT family N-acetyltransferase | 2.72516 | 563.84998 |
| C0J56_RS11500 | C0J56_RS11500 | upstream | tRNA-Gly | 1.59041 | 43.37629 |
| C0J56_RS11590 | C0J56_RS11590 | inside | LysR family transcriptional regulator | 1.38701 | 186.6555 |
| C0J56_RS11875 | C0J56_RS11875 | upstream | Uncharacterized protein | 1.3527 | 26.56007 |
| C0J56_RS12005 | C0J56_RS12005 | upstream | ABC transporter permease | 5.20896 | 12.086 |
| C0J56_RS12185 | galU | upstream | UTP--glucose-1-phosphate uridylyltransferase | 1.72668 | 10.1399 |
| C0J56_RS12215 | C0J56_RS12215 | upstream | transcriptional regulator | 3.19551 | 790.75171 |
| C0J56_RS12350 | C0J56_RS12350 | inside | branched-chain-amino-acid aminotransferase | 1.33448 | 39.31748 |
| C0J56_RS12370 | C0J56_RS12370 | upstream | 2-oxoisovalerate dehydrogenase subunit alpha | 3.76873 | 270.02011 |
| C0J56_RS12450 | hydA | upstream | D-hydantoinase/dihydropyrimidinase | 2.18099 | 9.89195 |
| C0J56_RS12485 | hcnA | upstream | hydrogen cyanide synthase subunit HcnA | 2.15182 | 404.36777 |
| C0J56_RS12570 | C0J56_RS12570 | upstream | methyl-accepting chemotaxis protein | 2.19438 | 92.84928 |
| C0J56_RS12600 | C0J56_RS12600 | upstream | membrane protein | 2.40511 | 93.04712 |
| C0J56_RS12645 | C0J56_RS12645 | upstream | Uncharacterized protein | 9.22386 | 103.59146 |
| C0J56_RS12655 | C0J56_RS12655 | upstream | Rhs family protein / insecticidal toxin protein | 9.20671 | 143.82074 |
| C0J56_RS12740 | C0J56_RS12740 | inside | Type VI secretion protein | 1.80524 | 2178.08887 |
| C0J56_RS12770 | C0J56_RS12770 | upstream | Uncharacterized protein | 2.74216 | 2171.33081 |
| C0J56_RS12780 | C0J56_RS12780 | inside | EamA domain-containing protein | 5.59705 | 50.80619 |
| C0J56_RS12820 | C0J56_RS12820 | upstream | Antibiotic biosynthesis monooxygenase | 10.91964 | 182.4801 |
| C0J56_RS12855 | acnB | upstream | aconitate hydratase B | 1.33688 | 895.81256 |
| C0J56_RS12865 | C0J56_RS12865 | upstream | Universal stress protein | 4.41406 | 2872.34668 |
| C0J56_RS12915 | C0J56_RS12915 | upstream | Uncharacterized protein | 5.12655 | 9.33607 |
| C0J56_RS12985 | C0J56_RS12985 | upstream | transcriptional regulator | 1.58567 | 624.80634 |
| C0J56_RS13195 | C0J56_RS13195 | upstream | transcriptional regulator | 3.28373 | 755.6189 |
| C0J56_RS13530 | C0J56_RS13530 | upstream | 2-hydroxymuconate tautomerase | 8.08926 | 28.00403 |
| C0J56_RS13545 | C0J56_RS13545 | upstream | Uncharacterized protein | 7.77559 | 288.43365 |
| C0J56_RS13620 | C0J56_RS13620 | upstream | Uncharacterized protein | 2.20304 | 1743.81714 |
| C0J56_RS13715 | C0J56_RS13715 | inside | molybdopterin molybdenumtransferase MoeA | 2.20549 | 1628.5741 |
| C0J56_RS13720 | C0J56_RS13720 | upstream | protease | 1.3641 | 95.92782 |
| C0J56_RS13745 | narX | upstream | histidine kinase | 3.57243 | 96.27231 |
| C0J56_RS13795 | C0J56_RS13795 | upstream | anaerobic ribonucleoside triphosphate reductase | 1.38224 | 12.68863 |
| C0J56_RS13840 | C0J56_RS13840 | inside | Iron transporter | 1.37292 | 356.9035 |
| C0J56_RS13850 | C0J56_RS13850 | upstream | Uncharacterized protein | 2.58898 | 11.99156 |
| C0J56_RS13855 | C0J56_RS13855 | upstream | dipeptidase | 1.86477 | 11.56191 |
| C0J56_RS14025 | C0J56_RS14025 | upstream | ABC transporter permease | 3.06319 | 159.5415 |
| C0J56_RS14260 | C0J56_RS14260 | upstream | Uncharacterized protein | 1.56345 | 53.46954 |
| C0J56_RS14360 | lpxO | upstream | LPS biosynthetic protein LpxO | 1.43357 | 243.21185 |
| C0J56_RS14390 | C0J56_RS14390 | upstream | Uncharacterized protein | 3.42097 | 24.36481 |
| C0J56_RS14490 | C0J56_RS14490 | upstream | Uncharacterized protein | 3.07544 | 14.96324 |
| C0J56_RS14675 | C0J56_RS14675 | upstream | 3-hydroxyisobutyrate dehydrogenase | 1.70558 | 317.90826 |
| C0J56_RS14710 | C0J56_RS14710 | upstream | alcohol dehydrogenase | 3.05828 | 245.65216 |
| C0J56_RS14745 | C0J56_RS14745 | upstream | Uncharacterized protein | 5.64361 | 36.82708 |
| C0J56_RS14760 | C0J56_RS14760 | upstream | serralysin family metalloprotease | 4.72711 | 242.23833 |
| C0J56_RS14770 | C0J56_RS14770 | upstream | ABC transporter ATP-binding protein | 4.06056 | 910.07739 |
| C0J56_RS14800 | C0J56_RS14800 | inside | YgdI/YgdR family lipoprotein | 1.34535 | 642.58319 |
| C0J56_RS15040 | C0J56_RS15040 | upstream | Uncharacterized protein | 1.338 | 467.57318 |
| C0J56_RS15115 | C0J56_RS15115 | upstream | transcriptional regulator | 1.29389 | 9.74287 |
| C0J56_RS15235 | C0J56_RS15235 | upstream | Putative hydro-lyase | 1.5011 | 9.35274 |
| C0J56_RS15290 | C0J56_RS15290 | upstream | AMP-binding protein | 2.49221 | 7.16028 |
| C0J56_RS15455 | C0J56_RS15455 | inside | LysE family translocator | 3.77384 | 19.61854 |
| C0J56_RS15505 | C0J56_RS15505 | upstream | peptide ABC transporter substrate-binding protein | 1.38456 | 139.76764 |
| C0J56_RS15510 | C0J56_RS15510 | inside | Uncharacterized protein | 1.38211 | 398.09283 |
| C0J56_RS15525 | C0J56_RS15525 | upstream | Uncharacterized protein | 1.3625 | 11.942 |
| C0J56_RS15625 | C0J56_RS15625 | upstream | BAX inhibitor protein | 2.09522 | 11.7987 |
| C0J56_RS15720 | valS | upstream | valine--tRNA ligase | 4.15368 | 10.68046 |
| C0J56_RS15800 | C0J56_RS15800 | upstream | tRNA-Cys | 1.60756 | 81.25595 |
| C0J56_RS16085 | C0J56_RS16085 | upstream | Uncharacterized protein | 1.46788 | 490.9252 |
| C0J56_RS16110 | C0J56_RS16110 | upstream | oxygen-independent coproporphyrinogen III oxidase | 3.21557 | 27.99854 |
| C0J56_RS16130 | C0J56_RS16130 | inside | channel protein TolC | 1.64677 | 17.25275 |
| C0J56_RS16130 | C0J56_RS16130 | inside | channel protein TolC | 1.59197 | 288.48465 |
| C0J56_RS16130 | C0J56_RS16130 | inside | channel protein TolC | 1.3625 | 27.84087 |
| C0J56_RS16135 | C0J56_RS16135 | inside | metalloprotease secretion protein | 1.30124 | 31.40657 |
| C0J56_RS16140 | C0J56_RS16140 | upstream | alkaline protease secretion ATP-binding protein AprD | 1.26673 | 10.68046 |
| C0J56_RS16155 | C0J56_RS16155 | inside | TetR-family transcriptional regulator | 1.29879 | 7.50759 |
| C0J56_RS16380 | C0J56_RS16380 | inside | Uncharacterized protein | 1.60511 | 6.21546 |
| C0J56_RS16440 | C0J56_RS16440 | inside | Uncharacterized protein | 1.54139 | 7.39109 |
| C0J56_RS16550 | C0J56_RS16550 | upstream | phosphoribosyltransferase | 1.39436 | 27.79105 |
| C0J56_RS16565 | C0J56_RS16565 | upstream | Hsp20/alpha crystallin family protein | 1.84213 | 22.62959 |
| C0J56_RS16615 | C0J56_RS16615 | upstream | alcohol dehydrogenase | 1.80322 | 12.5224 |
| C0J56_RS16640 | C0J56_RS16640 | upstream | membrane protein | 1.9567 | 50.85485 |
| C0J56_RS16760 | C0J56_RS16760 | upstream | two-component sensor | 4.82513 | 50.69379 |
| C0J56_RS16915 | C0J56_RS16915 | upstream | TetR/AcrR family transcriptional regulator | 3.28864 | 66.43597 |
| C0J56_RS16925 | fadD | upstream | long-chain-fatty-acid--CoA ligase | 4.74671 | 669.78546 |
| C0J56_RS16940 | C0J56_RS16940 | upstream | Polyketide synthase | 3.77139 | 289.46912 |
| C0J56_RS16960 | C0J56_RS16960 | inside | DNA-binding response regulator | 3.96432 | 647.99518 |
| C0J56_RS17095 | C0J56_RS17095 | upstream | diaminobutyrate--2-oxoglutarate transaminase | 1.49238 | 397.51538 |
| C0J56_RS17110 | C0J56_RS17110 | inside | Peptide synthase | 1.54875 | 450.20129 |
| C0J56_RS17355 | C0J56_RS17355 | upstream | resistance-nodulation-cell division (RND) multidrug efflux membrane fusion protein MexC | 1.92308 | 18.97469 |
| C0J56_RS17370 | C0J56_RS17370 | inside | ureidoglycolate lyase | 1.28975 | 23.20186 |
| C0J56_RS17420 | phlF | upstream | TetR/AcrR family transcriptional regulator | 1.51199 | 67.9269 |
| C0J56_RS17440 | phlD | upstream | type III polyketide synthase | 3.51409 | 7.2536 |
| C0J56_RS17455 | C0J56_RS17455 | inside | toxin | 2.62699 | 20.40312 |
| C0J56_RS17470 | C0J56_RS17470 | upstream | Uncharacterized protein | 2.06091 | 338.46796 |
| C0J56_RS17470 | C0J56_RS17470 | inside | Cable pili-associated 22 kDa adhesin protein | 1.6954 | 162.27409 |
| C0J56_RS17620 | C0J56_RS17620 | upstream | Anthranilate synthase, aminase component | 2.62454 | 76.79199 |
| C0J56_RS17670 | C0J56_RS17670 | upstream | LysR family transcriptional regulator | 2.31822 | 37.20412 |
| C0J56_RS17800 | C0J56_RS17800 | upstream | ABC transporter ATP-binding protein | 8.45439 | 161.85396 |
| C0J56_RS17830 | C0J56_RS17830 | upstream | Uncharacterized protein | 1.36005 | 112.6319 |
| C0J56_RS17840 | C0J56_RS17840 | upstream | MexE family multidrug efflux RND transporter periplasmic adaptor subunit | 1.38701 | 1880.67773 |
| C0J56_RS17845 | C0J56_RS17845 | inside | multidrug efflux RND transporter permease subunit | 8.11376 | 10.54413 |
| C0J56_RS17855 | C0J56_RS17855 | upstream | socitrate lyase/phosphoenolpyruvate mutase family protein | 12.06405 | 12.086 |
| C0J56_RS17895 | C0J56_RS17895 | upstream | Polyketide cyclase | 5.65341 | 1752.91272 |
| C0J56_RS17915 | C0J56_RS17915 | upstream | TetR/AcrR family transcriptional regulator | 10.23594 | 3367.55225 |
| C0J56_RS17975 | C0J56_RS17975 | upstream | TetR family transcriptional regulator | 1.82321 | 913.08917 |
| C0J56_RS18070 | mupI | upstream | acyl-homoserine-lactone synthase | 6.34095 | 2586.40845 |
| C0J56_RS18085 | mupR | upstream | LuxR family transcriptional regulator | 8.93222 | 48.72731 |
| C0J56_RS18090 | mupW | inside | aromatic ring-hydroxylating dioxygenase subunit alpha | 13.19837 | 1656.66687 |
| C0J56_RS18235 | mmpD | inside | polyketide synthase | 1.5561 | 23918.85352 |
| C0J56_RS18250 | mmpB | upstream | polyketide synthase | 6.69245 | 4519.00537 |
| C0J56_RS18475 | C0J56_RS18475 | upstream | sigma-54-dependent Fis family transcriptional regulator | 1.65314 | 23.78027 |
| C0J56_RS18910 | C0J56_RS18910 | upstream | DNA translocase FtsK | 1.44019 | 1248.46887 |
| C0J56_RS18930 | infA | upstream | translation initiation factor IF-1 | 1.55053 | 34.19755 |
| C0J56_RS18935 | clpA | inside | ATP-dependent Clp protease ATP-binding subunit ClpA | 1.20531 | 16.59132 |
| C0J56_RS18945 | C0J56_RS18945 | upstream | cold shock domain protein CspD | 1.83368 | 23.68004 |
| C0J56_RS19015 | C0J56_RS19015 | upstream | NADH-quinone oxidoreductase subunit A | 3.54476 | 4.29394 |
| C0J56_RS19280 | C0J56_RS19280 | upstream | AsnC family transcriptional regulator | 2.94556 | 49.90113 |
| C0J56_RS19295 | C0J56_RS19295 | upstream | tRNA-Pro | 3.97878 | 354.01181 |
| C0J56_RS19330 | infC | inside | translation initiation factor IF-3 | 4.7026 | 220.23271 |
| C0J56_RS19345 | C0J56_RS19345 | upstream | cold-shock protein | 1.80536 | 459.01392 |
| C0J56_RS19725 | C0J56_RS19725 | upstream | membrane protein | 8.94205 | 635.83881 |
| C0J56_RS19805 | C0J56_RS19805 | upstream | Uncharacterized protein | 4.56782 | 47.92154 |
| C0J56_RS19820 | C0J56_RS19820 | upstream | tRNA-Asp | 2.19484 | 44.99542 |
| C0J56_RS19850 | C0J56_RS19850 | upstream | colicin V production protein CvpA | 1.33736 | 2067.79224 |
| C0J56_RS19955 | C0J56_RS19955 | upstream | TetR family transcriptional regulator | 5.0313 | 599.1499 |
| C0J56_RS20015 | C0J56_RS20015 | upstream | AI-2E family transporter | 2.92692 | 106.58196 |
| C0J56_RS20150 | C0J56_RS20150 | upstream | two-component system sensor histidine kinase/response regulator | 1.58241 | 11.6207 |
| C0J56_RS20315 | C0J56_RS20315 | inside | Uncharacterized protein | 2.55592 | 817.58679 |
| C0J56_RS20335 | C0J56_RS20335 | upstream | 23S ribosomal RNA | 14.9949 | 264.79251 |
| C0J56_RS20420 | C0J56_RS20420 | upstream | PepSY domain-containing protein | 1.26584 | 29.79398 |
| C0J56_RS20580 | C0J56_RS20580 | upstream | Uncharacterized protein | 2.60493 | 150.25076 |
| C0J56_RS20620 | C0J56_RS20620 | upstream | Uncharacterized protein | 1.74724 | 4720.29883 |
| C0J56_RS20625 | C0J56_RS20625 | upstream | Uncharacterized protein | 3.28619 | 7.41971 |
| C0J56_RS20745 | C0J56_RS20745 | upstream | Ribonuclease E inhibitor RraB | 1.43203 | 158.50563 |
| C0J56_RS20945 | acpP | upstream | acyl carrier protein | 1.8668 | 40.8832 |
| C0J56_RS20970 | C0J56_RS20970 | upstream | Large ribosomal RNA subunit accumulation protein YceD | 3.35809 | 288.95129 |
| C0J56_RS20995 | rne | upstream | Ribonuclease E | 1.91951 | 17.11411 |
| C0J56_RS21180 | C0J56_RS21180 | upstream | Flp fap pilin component | 1.51558 | 63.72324 |
| C0J56_RS21195 | C0J56_RS21195 | upstream | alcohol dehydrogenase | 8.0525 | 355.06708 |
| C0J56_RS21225 | C0J56_RS21225 | upstream | tRNA-Gly | 3.27638 | 70.89191 |
| C0J56_RS21425 | sucA | upstream | 2-oxoglutarate dehydrogenase subunit E1 | 2.83019 | 21.77668 |
| C0J56_RS21715 | minE | inside | cell division topological specificity factor | 1.84001 | 1730.19885 |
| C0J56_RS21735 | C0J56_RS21735 | upstream | Uncharacterized protein | 1.68225 | 286.88324 |
| C0J56_RS21785 | C0J56_RS21785 | upstream | Uncharacterized protein | 3.16356 | 246.05017 |
| C0J56_RS21815 | C0J56_RS21815 | upstream | Putative autotransporter | 12.02974 | 60.88121 |
| C0J56_RS21875 | C0J56_RS21875 | inside | peptidyl-prolyl cis-trans isomerase | 1.60456 | 41.84266 |
| C0J56_RS21900 | C0J56_RS21900 | upstream | tRNA-Asp | 2.05069 | 297.39453 |
| C0J56_RS21960 | C0J56_RS21960 | inside | Uncharacterized protein | 3.12563 | 3352.41553 |
| C0J56_RS21960 | C0J56_RS21960 | inside | Uncharacterized protein | 2.92672 | 1158.50708 |
| C0J56_RS21995 | C0J56_RS21995 | upstream | Uncharacterized protein | 2.28823 | 33.53072 |
| C0J56_RS22085 | C0J56_RS22085 | upstream | peptidase P60 | 1.91173 | 91.6011 |
| C0J56_RS22160 | C0J56_RS22160 | upstream | Uncharacterized protein | 6.49078 | 242.92308 |
| C0J56_RS22365 | C0J56_RS22365 | inside | flagellar synthesis chaperone protein FlgN | 1.43213 | 284.38577 |
| C0J56_RS22395 | C0J56_RS22395 | inside | Uncharacterized protein | 1.52424 | 127.42865 |
| C0J56_RS22405 | C0J56_RS22405 | inside | Uncharacterized protein | 11.10098 | 72.33149 |
| C0J56_RS22455 | C0J56_RS22455 | upstream | DNA-binding protein | 2.79168 | 1270.54199 |
| C0J56_RS22480 | C0J56_RS22480 | upstream | tRNA-Arg | 4.00553 | 17.54687 |
| C0J56_RS22555 | C0J56_RS22555 | inside | acetylornithine aminotransferase 1 | 1.32879 | 21.31876 |
| C0J56_RS22580 | C0J56_RS22580 | upstream | ABC transporter substrate-binding protein（argT6） | 1.99045 | 2949.44238 |
| C0J56_RS22600 | C0J56_RS22600 | upstream | ribonucleoside-diphosphate reductase subunit beta/nrdB | 9.23121 | 218.14394 |
| C0J56_RS22615 | C0J56_RS22615 | upstream | Type II toxin-antitoxin system RelE/ParE family toxin | 2.12217 | 489.22925 |
| C0J56_RS22695 | C0J56_RS22695 | upstream | PepSY domain-containing protein | 1.40464 | 12.07437 |
| C0J56_RS22725 | C0J56_RS22725 | upstream | aromatic amino acid transporter AroP | 2.1223 | 78.45442 |
| C0J56_RS22910 | C0J56_RS22910 | upstream | Uncharacterized protein | 5.0962 | 2180.98755 |
| C0J56_RS22950 | C0J56_RS22950 | upstream | (2Fe-2S)-binding protein | 1.78853 | 84.83382 |
| C0J56_RS22960 | grxD | inside | glutaredoxin | 1.32542 | 15.76463 |
| C0J56_RS22995 | glpF | upstream | aquaporin | 1.92177 | 100.40361 |
| C0J56_RS23005 | C0J56_RS23005 | upstream | DeoR family transcriptional regulator | 1.71118 | 828.2738 |
| C0J56_RS23015 | glpD | upstream | glycerol-3-phosphate dehydrogenase | 4.00127 | 51.99342 |
| C0J56_RS23100 | C0J56_RS23100 | upstream | cold-shock protein | 3.07729 | 10.21644 |
| C0J56_RS23160 | ybgF | inside | Cell division coordinator CpoB | 2.33794 | 72.63177 |
| C0J56_RS23250 | C0J56_RS23250 | upstream | porin | 2.05233 | 46.87104 |
| C0J56_RS23255 | C0J56_RS23255 | inside | mechanosensitive ion channel protein MscS | 1.42651 | 677.42413 |
| C0J56_RS23335 | C0J56_RS23335 | upstream | transcriptional regulator | 3.91479 | 293.65472 |
| C0J56_RS23510 | C0J56_RS23510 | upstream | alpha/beta hydrolase | 2.49222 | 154.22226 |
| C0J56_RS23605 | C0J56_RS23605 | upstream | Uncharacterized protein | 6.4817 | 82.2808 |
| C0J56_RS23660 | C0J56_RS23660 | upstream | Uncharacterized protein | 2.79455 | 16.08181 |
| C0J56_RS23705 | C0J56_RS23705 | upstream | Uncharacterized protein | 3.57022 | 496.01669 |
| C0J56_RS23870 | lepB | inside | signal peptidase I | 1.52078 | 179.51749 |
| C0J56_RS23910 | rlmF | upstream | ribosomal RNA large subunit methyltransferase F | 2.06451 | 1177.97705 |
| C0J56_RS23990 | rhlB | inside | ATP-dependent RNA helicase RhlB | 9.63801 | 198.75885 |
| C0J56_RS23995 | C0J56_RS23995 | inside | Rhs family protein / insecticidal toxin protein | 9.54489 | 385.42453 |
| C0J56_RS24000 | C0J56_RS24000 | upstream | Rhs family protein / insecticidal toxin protein | 10.24329 | 24.65784 |
| C0J56_RS24005 | C0J56_RS24005 | upstream | Rhs family protein / insecticidal toxin protein | 10.14772 | 85.30912 |
| C0J56_RS24015 | C0J56_RS24015 | inside | Uncharacterized protein | 12.36302 | 2342.91553 |
| C0J56_RS24095 | algD | upstream | GDP-mannose 6-dehydrogenase | 4.04776 | 2305.57617 |
| C0J56_RS24245 | lpxC | upstream | UDP-3-O-acyl-N-acetylglucosamine deacetylase | 1.49088 | 2589.44141 |
| C0J56_RS24310 | ftsL | inside | cell division protein FtsL | 2.17096 | 2550.08691 |
| C0J56_RS24360 | sspA | upstream | stringent starvation protein A/sspA | 7.63242 | 3500.20435 |
| C0J56_RS24480 | cyoA | upstream | ubiquinol oxidase subunit 2 | 1.57871 | 503.40448 |
| C0J56_RS24545 | C0J56_RS24545 | upstream | ribosomal subunit interface protein | 1.46301 | 22.98792 |
| C0J56_RS24625 | C0J56_RS24625 | upstream | Uncharacterized protein | 3.91475 | 108.69107 |
| C0J56_RS24645 | C0J56_RS24645 | upstream | Colicin/Pyocin-S2 | 8.60387 | 1740.62061 |
| C0J56_RS24650 | C0J56_RS24650 | inside | pyocin-S2 immunity protein | 9.96883 | 27.43415 |
| C0J56_RS24685 | C0J56_RS24685 | inside | Uncharacterized protein | 1.74315 | 17.70248 |
| C0J56_RS24865 | C0J56_RS24865 | upstream | Uncharacterized protein | 2.94338 | 487.9314 |
| C0J56_RS24875 | C0J56_RS24875 | upstream | Uncharacterized protein | 3.19385 | 1937.51965 |
| C0J56_RS24935 | C0J56_RS24935 | upstream | short-chain dehydrogenase | 3.54827 | 2476.85229 |
| C0J56_RS24990 | C0J56_RS24990 | upstream | tRNA-Gln | 4.28539 | 45.46806 |
| C0J56_RS25170 | C0J56_RS25170 | upstream | paraquat-inducible protein A | 18.42812 | 235.2742 |
| C0J56_RS25230 | ilvB | upstream | acetolactate synthase | 1.38088 | 293.71402 |
| C0J56_RS25335 | C0J56_RS25335 | upstream | poly(A) polymerase I | 1.92826 | 429.48065 |
| C0J56_RS25420 | C0J56_RS25420 | upstream | Uncharacterized protein | 3.7844 | 563.23407 |
| C0J56_RS25455 | C0J56_RS25455 | upstream | tRNA-Pro | 2.88126 | 6433.73926 |
| C0J56_RS25490 | C0J56_RS25490 | inside | outer membrane protein assembly factor BamD | 1.84616 | 12.06701 |
| C0J56_RS25620 | rpsT | upstream | 30S ribosomal protein S20 | 1.84594 | 64.23833 |
| C0J56_RS25650 | C0J56_RS25650 | upstream | octaprenyl diphosphate synthase | 2.8434 | 431.00192 |
| C0J56_RS25735 | C0J56_RS25735 | upstream | Uncharacterized protein | 4.10401 | 211.05612 |
| C0J56_RS25785 | katB | inside | catalase | 2.49711 | 53.81037 |
| C0J56_RS25795 | C0J56_RS25795 | upstream | ferredoxin--NADP(+) reductase | 3.25433 | 53.85853 |
| C0J56_RS25960 | asnB | upstream | asparagine synthetase B | 1.35515 | 205.89288 |
| C0J56_RS25970 | C0J56_RS25970 | upstream | Uncharacterized protein | 2.08787 | 932.73798 |
| C0J56_RS26020 | wbpA | upstream | UDP-N-acetyl-D-glucosamine 6-dehydrogenase | 2.04131 | 486.47134 |
| C0J56_RS26115 | C0J56_RS26115 | upstream | Uncharacterized protein | 5.19271 | 140.56393 |
| C0J56_RS26390 | C0J56_RS26390 | upstream | transcriptional regulator | 2.42849 | 282.24902 |
| C0J56_RS26395 | C0J56_RS26395 | upstream | Uncharacterized protein | 1.95554 | 10.27386 |
| C0J56_RS26405 | C0J56_RS26405 | inside | OmpA family protein | 2.86714 | 80.291 |
| C0J56_RS26690 | C0J56_RS26690 | upstream | outer membrane protein W | 9.80949 | 74.28659 |
| C0J56_RS26950 | rplB | upstream | 50S ribosomal protein L2 | 4.21739 | 774.88702 |
| C0J56_RS26985 | fusA | inside | elongation factor G | 2.75442 | 129.57323 |
| C0J56_RS27040 | C0J56_RS27040 | upstream | tRNA-Trp | 5.92298 | 63.7169 |
| C0J56_RS27085 | C0J56_RS27085 | upstream | 23S ribosomal RNA | 17.10483 | 205.37383 |
| C0J56_RS27120 | C0J56_RS27120 | upstream | Cell wall endopeptidase, family M23/M37 | 2.79117 | 2761.52637 |
| C0J56_RS27165 | coq7 | upstream | 2-nonaprenyl-3-methyl-6-methoxy-1,4-benzoquinol hydroxylase | 1.47278 | 507.11591 |
| C0J56_RS27360 | rpsU | upstream | 30S ribosomal protein S21 | 2.62699 | 184.681 |
| C0J56_RS27370 | rpoD | upstream | RNA polymerase sigma factor RpoD | 2.20059 | 997.07544 |
| C0J56_RS27395 | C0J56_RS27395 | inside | tryptophan synthase subunit alpha | 1.52899 | 5758.57715 |
| C0J56_RS27440 | C0J56_RS27440 | upstream | UPF0057 membrane protein | 2.18589 | 191.34102 |
| C0J56_RS27470 | C0J56_RS27470 | inside | Uncharacterized protein | 1.34045 | 17.59159 |
| C0J56_RS27625 | C0J56_RS27625 | upstream | cold shock domain-containing protein | 1.52669 | 162.27409 |
| C0J56_RS27690 | C0J56_RS27690 | upstream | Uncharacterized protein | 1.94819 | 95.584 |
| C0J56_RS27790 | C0J56_RS27790 | upstream | alginate lyase family protein | 4.57027 | 21.71492 |
| C0J56_RS28380 | rpoH | upstream | RNA polymerase sigma factor RpoH | 2.09767 | 93.53008 |
| C0J56_RS28435 | C0J56_RS28435 | inside | putative coniferyl aldehyde dehydrogenase | 1.38835 | 9.48195 |
| C0J56_RS28710 | C0J56_RS28710 | upstream | Auxin Efflux Carrier /AEC family transporter | 10.0448 | 21.50401 |
| C0J56_RS29000 | rho | upstream | transcription termination factor Rho | 1.71014 | 62.84139 |
| C0J56_RS29180 | C0J56_RS29180 | upstream | TIGR02647 family protein/DNA-binding protein | 1.59286 | 599.81091 |
| C0J56_RS29335 | dadA | inside | D-amino acid dehydrogenase | 1.46264 | 81.57881 |
| C0J56_RS29395 | rpmB | upstream | 50S ribosomal protein L28 | 2.59196 | 12.96038 |
| C0J56_RS29440 | gltS | upstream | sodium/glutamate symporter | 3.95273 | 2507.88379 |
| C0J56_RS29465 | rpoZ | upstream | DNA-directed RNA polymerase subunit omega | 2.41609 | 37.6922 |
| C0J56_RS29520 | C0J56_RS29520 | inside | Uncharacterized protein | 1.79625 | 26.76377 |
| C0J56_RS29540 | C0J56_RS29540 | upstream | Uncharacterized protein | 4.34482 | 19.21673 |
| C0J56_RS29610 | tagH | upstream | type VI secretion system-associated FHA domain protein TagH | 1.76439 | 156.34879 |
| C0J56_RS29710 | C0J56_RS29710 | upstream | Uncharacterized protein | 1.44582 | 441.00876 |
| C0J56_RS30010 | atpC | inside | ATP synthase epsilon chain | 1.48013 | 152.71208 |
| C0J56_RS30015 | atpD | upstream | ATP synthase subunit beta | 1.69045 | 45.87835 |
| C0J56_RS30030 | atpH | inside | ATP synthase subunit delta | 2.29861 | 540.00781 |
| C0J56_RS30095 | rnpA | upstream | ribonuclease P protein component | 3.8155 | 42.60405 |
| C0J56_RS30115 | C0J56_RS30115 | upstream | Uncharacterized protein | 1.8568 | 15.76444 |
| C0J56_RS30150 | C0J56_RS30150 | upstream | Uncharacterized protein | 10.29218 | 18.10474 |
| C0J56_RS30295 | C0J56_RS30295 | inside | Uncharacterized protein | 1.73053 | 37.2341 |
| C0J56_RS30335 | C0J56_RS30335 | upstream | Uncharacterized protein | 5.71713 | 109.71577 |
| C0J56_RS30760 | C0J56_RS30760 | inside | CdiI_2 domain-containing protein | 5.94039 | 407.95569 |

**Sample 2 vs input**

| geneID  (target genes) | Gene name | Position | Gene description | Fold_enrichment | -log10(pvalue) |
| --- | --- | --- | --- | --- | --- |
| C0J56_RS00005 | dnaA | inside | chromosomal replication initiator protein DnaA | 1.35018 | 11.9483 |
| C0J56_RS00120 | C0J56_RS00120 | upstream | ATPase | 7.89629 | 1826.68481 |
| C0J56_RS00200 | C0J56_RS00200 | upstream | Cytochrome c5 | 1.63534 | 33.13492 |
| C0J56_RS00425 | C0J56_RS00425 | upstream | AAA family ATPase | 8.68144 | 2148.32227 |
| C0J56_RS00470 | C0J56_RS00470 | upstream | RNA-directed DNA polymerase | 2.831 | 216.85939 |
| C0J56_RS00595 | C0J56_RS00595 | upstream | Uncharacterized protein | 5.8796 | 1073.93237 |
| C0J56_RS00630 | pycA | upstream | pyruvate carboxylase subunit A/2-oxoglutarate carboxylase small subunit | 2.20962 | 105.69859 |
| C0J56_RS00775 | aspA | inside | aspartate ammonia-lyase | 1.64656 | 34.21801 |
| C0J56_RS00985 | C0J56_RS00985 | upstream | Uncharacterized protein | 9.53612 | 2514.01001 |
| C0J56_RS00995 | C0J56_RS00995 | upstream | Succinate CoA transferase | 1.85742 | 57.21311 |
| C0J56_RS01035 | C0J56_RS01035 | upstream | 16S ribosomal RNA | 15.79707 | 5580.61523 |
| C0J56_RS01275 | C0J56_RS01275 | upstream | Heme oxygenase-like protein | 3.64755 | 402.56137 |
| C0J56_RS01315 | C0J56_RS01315 | inside | LapA family giant adhesin | 1.54602 | 8.98086 |
| C0J56_RS01315 | C0J56_RS01315 | upstream | LapA family giant adhesin | 1.39082 | 30.07907 |
| C0J56_RS01315 | C0J56_RS01315 | inside | LapA family giant adhesin | 1.30013 | 8.5921 |
| C0J56_RS01315 | C0J56_RS01315 | inside | LapA family giant adhesin | 1.28843 | 9.20336 |
| C0J56_RS01315 | C0J56_RS01315 | inside | LapA family giant adhesin | 1.28479 | 13.40394 |
| C0J56_RS01355 | C0J56_RS01355 | inside | ABC transporter permease | 1.27193 | 6.70955 |
| C0J56_RS01370 | C0J56_RS01370 | inside | alpha/beta hydrolase | 1.2551 | 6.08214 |
| C0J56_RS01370 | C0J56_RS01370 | inside | alpha/beta hydrolase | 1.20946 | 4.2474 |
| C0J56_RS01380 | C0J56_RS01380 | inside | Multidrug resistance efflux pump | 1.22956 | 5.20499 |
| C0J56_RS01565 | serA | upstream | D-3-phosphoglycerate dehydrogenase | 1.7991 | 50.36646 |
| C0J56_RS01620 | C0J56_RS01620 | upstream | Uncharacterized protein | 2.72108 | 195.09001 |
| C0J56_RS02210 | C0J56_RS02210 | inside | lipocalin | 1.24197 | 5.58458 |
| C0J56_RS02540 | gbpA | upstream | GlcNAc-binding protein A | 1.8148 | 52.17409 |
| C0J56_RS02615 | C0J56_RS02615 | upstream | Uncharacterized protein | 3.5623 | 381.36774 |
| C0J56_RS02620 | C0J56_RS02620 | inside | lipoprotein | 2.06605 | 84.51837 |
| C0J56_RS02625 | C0J56_RS02625 | upstream | Uncharacterized protein | 4.8118 | 727.3457 |
| C0J56_RS02830 | C0J56_RS02830 | upstream | acyl-CoA dehydrogenase | 1.3835 | 13.37546 |
| C0J56_RS02835 | putA | inside | bifunctional protein PutA | 1.71334 | 43.37177 |
| C0J56_RS02845 | C0J56_RS02845 | upstream | Uncharacterized protein | 2.47881 | 150.19173 |
| C0J56_RS03210 | hflX | inside | GTPase HflX | 1.56877 | 28.07536 |
| C0J56_RS03245 | C0J56_RS03245 | upstream | tRNA-Leu | 1.8148 | 52.17409 |
| C0J56_RS03280 | C0J56_RS03280 | inside | Uncharacterized protein | 1.98061 | 78.47625 |
| C0J56_RS03340 | azu | upstream | Azurin | 3.92585 | 486.53873 |
| C0J56_RS03580 | C0J56_RS03580 | upstream | TonB-dependent copper receptor | 1.32373 | 10.71489 |
| C0J56_RS03640 | C0J56_RS03640 | upstream | Toxin A | 3.06866 | 281.28879 |
| C0J56_RS03645 | C0J56_RS03645 | upstream | UPF0056 inner membrane protein | 4.12761 | 528.87701 |
| C0J56_RS03980 | C0J56_RS03980 | upstream | 16S ribosomal RNA | 16.62035 | 64.06477 |
| C0J56_RS04190 | C0J56_RS04190 | upstream | proline iminopeptidase | 1.30195 | 6024.79639 |
| C0J56_RS04220 | C0J56_RS04220 | inside | addiction module antidote protein, HigA family | 1.58465 | 8.87574 |
| C0J56_RS04375 | ssrA | upstream | transfer-messenger RNA | 2.31505 | 28.69039 |
| C0J56_RS04415 | smpB | inside | SsrA-binding protein | 1.48293 | 122.41071 |
| C0J56_RS04435 | C0J56_RS04435 | upstream | outer membrane protein assembly factor BamE | 1.57467 | 22.52946 |
| C0J56_RS04455 | dnaK | inside | chaperone protein DnaK | 1.44284 | 29.05354 |
| C0J56_RS04470 | carA | upstream | carbamoyl-phosphate synthase small chain | 2.85803 | 17.54866 |
| C0J56_RS04525 | C0J56_RS04525 | upstream | tRNA-Leu | 3.14134 | 239.87943 |
| C0J56_RS04545 | infB | inside | translation initiation factor IF-2 | 1.39532 | 303.52628 |
| C0J56_RS04550 | rbfA | upstream | ribosome-binding factor A | 1.34352 | 17.68035 |
| C0J56_RS04785 | C0J56_RS04785 | upstream | Uncharacterized protein | 1.93349 | 12.54868 |
| C0J56_RS04860 | mreC | upstream | rod shape-determining protein MreC | 1.73169 | 72.35832 |
| C0J56_RS04945 | raiA | upstream | ribosomal subunit interface protein | 1.34045 | 49.63364 |
| C0J56_RS05010 | murA | inside | UDP-N-acetylglucosamine 1-carboxyvinyltransferase | 1.22885 | 11.76631 |
| C0J56_RS05175 | C0J56_RS05175 | upstream | Reverse transcriptase domain-containing protein | 12.5892 | 5.89773 |
| C0J56_RS05245 | C0J56_RS05245 | upstream | Putative exported protein | 1.58627 | 3933.64673 |
| C0J56_RS05400 | guaA | upstream | GMP synthase [glutamine-hydrolyzing] | 1.46575 | 33.2089 |
| C0J56_RS05510 | rimM | inside | ribosome maturation factor RimM | 3.03085 | 22.28651 |
| C0J56_RS05885 | rpsB | upstream | 30S ribosomal protein S2 | 3.26461 | 300.82361 |
| C0J56_RS05925 | bamA | upstream | outer membrane protein assembly factor BamA | 1.31723 | 364.4382 |
| C0J56_RS06260 | recA | inside | protein RecA | 1.45344 | 10.40438 |
| C0J56_RS06300 | C0J56_RS06300 | upstream | LysR family transcriptional regulator | 3.1919 | 21.72662 |
| C0J56_RS06405 | C0J56_RS06405 | upstream | cold shock protein CapB | 1.69282 | 368.71799 |
| C0J56_RS06455 | C0J56_RS06455 | upstream | acyltransferase | 3.8229 | 45.38553 |
| C0J56_RS06685 | arcA | inside | arginine deiminase | 4.59267 | 527.65112 |
| C0J56_RS06975 | C0J56_RS06975 | upstream | superoxide dismutase | 2.1283 | 791.0531 |
| C0J56_RS07025 | C0J56_RS07025 | upstream | Uncharacterized protein | 1.35927 | 131.56087 |
| C0J56_RS07125 | C0J56_RS07125 | upstream | secretion protein HlyD | 3.65084 | 2569.33008 |
| C0J56_RS07240 | yiaY | upstream | alcohol dehydrogenase | 1.59089 | 13.54318 |
| C0J56_RS07295 | C0J56_RS07295 | upstream | Uncharacterized protein | 2.45205 | 467.25253 |
| C0J56_RS07460 | C0J56_RS07460 | inside | sigma factor AlgU negative regulatory protein | 3.16578 | 44.80056 |
| C0J56_RS07595 | C0J56_RS07595 | upstream | phosphoethanolamine transferase | 1.42684 | 152.77667 |
| C0J56_RS07705 | C0J56_RS07705 | upstream | MFS transporter | 1.48585 | 408.87314 |
| C0J56_RS07720 | C0J56_RS07720 | inside | Biotin-dependent carboxyltransferase | 1.27681 | 19.04456 |
| C0J56_RS07875 | C0J56_RS07875 | upstream | Uncharacterized protein | 6.97655 | 27.01349 |
| C0J56_RS07875 | C0J56_RS07875 | inside | Uncharacterized protein | 3.53087 | 9.27221 |
| C0J56_RS07895 | C0J56_RS07895 | upstream | IS4 family transposase | 7.92321 | 402.9874 |
| C0J56_RS07960 | C0J56_RS07960 | upstream | Uncharacterized protein | 2.38252 | 1469.22205 |
| C0J56_RS08045 | C0J56_RS08045 | inside | Uncharacterized protein | 3.19799 | 1837.47131 |
| C0J56_RS08060 | C0J56_RS08060 | inside | inner membrane protein | 1.47311 | 139.83792 |
| C0J56_RS08130 | C0J56_RS08130 | inside | Putative NAD(P)H nitroreductase | 1.27913 | 330.11292 |
| C0J56_RS08140 | C0J56_RS08140 | upstream | pterin-4-alpha-carbinolamine dehydratase | 1.35748 | 25.00138 |
| C0J56_RS08220 | ntrC1 | upstream | sigma-54-dependent Fis family transcriptional regulator | 4.85856 | 9.13443 |
| C0J56_RS08355 | cheY | upstream | chemotaxis protein CheY | 1.79875 | 13.85863 |
| C0J56_RS08470 | C0J56_RS08470 | upstream | Uncharacterized protein | 1.65475 | 833.5603 |
| C0J56_RS08605 | gyrA | upstream | DNA gyrase subunit A | 1.32598 | 63.15466 |
| C0J56_RS08635 | rpsA | upstream | 30S ribosomal protein S1 | 3.13012 | 42.88778 |
| C0J56_RS08650 | C0J56_RS08650 | inside | LapA family protein | 11.28587 | 11.98508 |
| C0J56_RS08800 | C0J56_RS08800 | upstream | methyl-accepting chemotaxis protein | 2.63223 | 355.93024 |
| C0J56_RS08820 | C0J56_RS08820 | upstream | Class I SAM-dependent methyltransferase | 1.47887 | 3307.6416 |
| C0J56_RS08855 | oprI | upstream | major outer membrane lipoprotein | 3.44199 | 212.49336 |
| C0J56_RS08960 | C0J56_RS08960 | inside | ATP-dependent RNA helicase DeaD | 1.79681 | 24.65089 |
| C0J56_RS09030 | C0J56_RS09030 | inside | hybrid sensor histidine kinase/response regulator | 2.29849 | 409.89709 |
| C0J56_RS09085 | C0J56_RS09085 | upstream | lipoprotein | 1.41057 | 61.45666 |
| C0J56_RS09120 | C0J56_RS09120 | upstream | Sensor histidine kinase | 2.08633 | 136.72475 |
| C0J56_RS09240 | C0J56_RS09240 | inside | antibiotic synthesis protein MbtH | 1.55491 | 17.32671 |
| C0J56_RS09295 | C0J56_RS09295 | upstream | porin | 2.81365 | 108.38686 |
| C0J56_RS09420 | C0J56_RS09420 | upstream | Uncharacterized protein | 2.12845 | 31.48091 |
| C0J56_RS09515 | C0J56_RS09515 | upstream | GntR family transcriptional regulator | 1.82766 | 249.8981 |
| C0J56_RS09580 | ffs | upstream | SRP_RNA（signal recognition particle sRNA small type） | 1.72153 | 100.75565 |
| C0J56_RS09630 | C0J56_RS09630 | upstream | adenine phosphoribosyltransferase | 4.84636 | 66.95323 |
| C0J56_RS09660 | C0J56_RS09660 | inside | membrane protein | 2.156 | 45.26465 |
| C0J56_RS09705 | C0J56_RS09705 | inside | cytochrome oxidase | 3.98255 | 992.2514 |
| C0J56_RS09825 | C0J56_RS09825 | inside | Uncharacterized protein | 1.49643 | 123.78092 |
| C0J56_RS09930 | C0J56_RS09930 | upstream | chromosome segregation ATPase | 1.51918 | 681.83923 |
| C0J56_RS09970 | C0J56_RS09970 | upstream | Uncharacterized protein | 6.40676 | 24.40805 |
| C0J56_RS10025 | C0J56_RS10025 | inside | HNH endonuclease | 11.86911 | 25.53854 |
| C0J56_RS10210 | C0J56_RS10210 | inside | Abasic site processing protein | 3.38957 | 1259.36328 |
| C0J56_RS10225 | C0J56_RS10225 | upstream | Uncharacterized protein | 11.44514 | 3584.375 |
| C0J56_RS10325 | C0J56_RS10325 | upstream | Uncharacterized protein | 1.78145 | 339.64026 |
| C0J56_RS10440 | C0J56_RS10440 | upstream | tRNA-Arg | 4.6615 | 3382.63892 |
| C0J56_RS10465 | clpP | upstream | ATP-dependent Clp protease proteolytic subunit | 1.95053 | 56.08781 |
| C0J56_RS10480 | C0J56_RS10480 | upstream | DNA-binding protein HU-beta | 3.28869 | 682.0332 |
| C0J56_RS10675 | C0J56_RS10675 | inside | Uncharacterized protein | 4.97117 | 78.18239 |
| C0J56_RS10935 | C0J56_RS10935 | inside | Uncharacterized protein | 1.46274 | 319.48398 |
| C0J56_RS10945 | ccoG | upstream | Cytochrome c oxidase accessory protein CcoG | 3.653 | 829.14044 |
| C0J56_RS11010 | C0J56_RS11010 | inside | acetyl-CoA acetyltransferase | 1.50536 | 19.52337 |
| C0J56_RS11055 | C0J56_RS11055 | upstream | General stress protein | 2.21892 | 533.86206 |
| C0J56_RS11235 | C0J56_RS11235 | upstream | transcriptional regulator | 4.03338 | 24.63676 |
| C0J56_RS11305 | C0J56_RS11305 | inside | cobalamin biosynthesis protein CbiG | 1.50478 | 111.9637 |
| C0J56_RS11480 | C0J56_RS11480 | inside | GNAT family N-acetyltransferase | 2.54231 | 593.04138 |
| C0J56_RS11500 | C0J56_RS11500 | upstream | tRNA-Gly | 1.49401 | 25.19972 |
| C0J56_RS11590 | C0J56_RS11590 | inside | LysR family transcriptional regulator | 1.45588 | 166.73648 |
| C0J56_RS11915 | C0J56_RS11915 | upstream | LysR family transcriptional regulator | 1.47158 | 20.79786 |
| C0J56_RS12005 | C0J56_RS12005 | upstream | ABC transporter permease | 4.19366 | 17.90033 |
| C0J56_RS12215 | C0J56_RS12215 | upstream | transcriptional regulator | 2.56405 | 5.7552 |
| C0J56_RS12370 | C0J56_RS12370 | upstream | 2-oxoisovalerate dehydrogenase subunit alpha | 3.15082 | 19.07066 |
| C0J56_RS12450 | hydA | upstream | D-hydantoinase/dihydropyrimidinase | 1.77891 | 561.30536 |
| C0J56_RS12485 | hcnA | upstream | hydrogen cyanide synthase subunit HcnA | 1.86749 | 165.48862 |
| C0J56_RS12570 | C0J56_RS12570 | upstream | methyl-accepting chemotaxis protein | 1.96194 | 291.13937 |
| C0J56_RS12600 | C0J56_RS12600 | upstream | membrane protein | 2.41492 | 48.08168 |
| C0J56_RS12645 | C0J56_RS12645 | upstream | Uncharacterized protein | 8.08024 | 61.39108 |
| C0J56_RS12655 | C0J56_RS12655 | upstream | Rhs family protein / insecticidal toxin protein | 8.37859 | 77.01669 |
| C0J56_RS12770 | C0J56_RS12770 | upstream | Uncharacterized protein | 2.62462 | 162.15565 |
| C0J56_RS12780 | C0J56_RS12780 | inside | EamA domain-containing protein | 5.92671 | 1900.73743 |
| C0J56_RS12820 | C0J56_RS12820 | upstream | Antibiotic biosynthesis monooxygenase | 10.6757 | 2022.56262 |
| C0J56_RS12865 | C0J56_RS12865 | upstream | Universal stress protein | 3.82998 | 176.69193 |
| C0J56_RS12915 | C0J56_RS12915 | upstream | Uncharacterized protein | 4.54261 | 1090.14136 |
| C0J56_RS12985 | C0J56_RS12985 | upstream | transcriptional regulator | 1.32019 | 3024.44409 |
| C0J56_RS13200 | C0J56_RS13200 | upstream | Uncharacterized protein | 1.73629 | 506.81784 |
| C0J56_RS13530 | C0J56_RS13530 | upstream | 2-hydroxymuconate tautomerase | 5.00472 | 646.84991 |
| C0J56_RS13545 | C0J56_RS13545 | upstream | Uncharacterized protein | 4.84097 | 9.88592 |
| C0J56_RS13615 | C0J56_RS13615 | inside | protein NirF | 1.84845 | 43.40299 |
| C0J56_RS13715 | C0J56_RS13715 | inside | molybdopterin molybdenumtransferase MoeA | 2.13248 | 786.83502 |
| C0J56_RS13720 | C0J56_RS13720 | upstream | protease | 1.32619 | 736.24329 |
| C0J56_RS13745 | narX | upstream | histidine kinase | 3.14777 | 56.13649 |
| C0J56_RS13835 | C0J56_RS13835 | inside | Iron permease, FTR1 family | 1.66055 | 94.12497 |
| C0J56_RS13850 | C0J56_RS13850 | upstream | Uncharacterized protein | 2.26629 | 11.12094 |
| C0J56_RS14025 | C0J56_RS14025 | upstream | ABC transporter permease | 3.34919 | 289.31409 |
| C0J56_RS14260 | C0J56_RS14260 | upstream | Uncharacterized protein | 1.36615 | 36.7695 |
| C0J56_RS14360 | lpxO | upstream | LPS biosynthetic protein LpxO | 1.39531 | 114.75809 |
| C0J56_RS14360 | lpxO | inside | LPS biosynthetic protein LpxO | 1.33474 | 330.12906 |
| C0J56_RS14390 | C0J56_RS14390 | upstream | Uncharacterized protein | 3.45911 | 11.84902 |
| C0J56_RS14490 | C0J56_RS14490 | upstream | Uncharacterized protein | 2.02567 | 13.69572 |
| C0J56_RS14675 | C0J56_RS14675 | upstream | 3-hydroxyisobutyrate dehydrogenase | 1.52542 | 9.99376 |
| C0J56_RS14710 | C0J56_RS14710 | upstream | alcohol dehydrogenase | 1.49177 | 356.23947 |
| C0J56_RS14715 | C0J56_RS14715 | upstream | Uncharacterized protein | 2.8983 | 78.90315 |
| C0J56_RS14745 | C0J56_RS14745 | upstream | Uncharacterized protein | 4.44166 | 23.32413 |
| C0J56_RS14760 | C0J56_RS14760 | upstream | serralysin family metalloprotease | 4.52915 | 20.62225 |
| C0J56_RS14770 | C0J56_RS14770 | upstream | ABC transporter ATP-binding protein | 4.46185 | 230.59796 |
| C0J56_RS14990 | C0J56_RS14990 | upstream | Uncharacterized protein | 1.44733 | 617.45374 |
| C0J56_RS15025 | C0J56_RS15025 | upstream | Uncharacterized protein | 1.31904 | 642.90485 |
| C0J56_RS15040 | C0J56_RS15040 | upstream | Uncharacterized protein | 1.50523 | 623.29858 |
| C0J56_RS15055 | C0J56_RS15055 | inside | Lipocalin-like domain-containing protein | 1.48953 | 17.58984 |
| C0J56_RS15060 | C0J56_RS15060 | upstream | multidrug efflux outer membrane protein OprN | 1.29885 | 9.11864 |
| C0J56_RS15060 | C0J56_RS15060 | upstream | multidrug efflux outer membrane protein OprN | 1.29212 | 21.686 |
| C0J56_RS15065 | C0J56_RS15065 | inside | acriflavine resistance protein B | 1.35045 | 20.44722 |
| C0J56_RS15065 | C0J56_RS15065 | inside | acriflavine resistance protein B | 1.34596 | 7.70182 |
| C0J56_RS15080 | C0J56_RS15080 | inside | Histidine kinase | 1.24737 | 8.04602 |
| C0J56_RS15105 | C0J56_RS15105 | inside | aldehyde oxidase | 1.26071 | 10.64026 |
| C0J56_RS15115 | C0J56_RS15115 | upstream | transcriptional regulator | 1.39531 | 10.90384 |
| C0J56_RS15240 | C0J56_RS15240 | inside | Uncharacterized protein | 1.29212 | 5.64434 |
| C0J56_RS15245 | C0J56_RS15245 | inside | LysR family transcriptional regulator | 1.31455 | 6.1842 |
| C0J56_RS15290 | C0J56_RS15290 | upstream | AMP-binding protein | 2.10867 | 13.69572 |
| C0J56_RS15455 | C0J56_RS15455 | inside | LysE family translocator | 3.08449 | 7.70182 |
| C0J56_RS15505 | C0J56_RS15505 | upstream | peptide ABC transporter substrate-binding protein | 1.36166 | 8.87502 |
| C0J56_RS15515 | C0J56_RS15515 | inside | acyl-homoserine lactone acylase PvdQ | 1.37736 | 90.61046 |
| C0J56_RS15520 | C0J56_RS15520 | upstream | Fe2+ zn2+ uptake regulation protein | 1.50972 | 270.16458 |
| C0J56_RS15590 | C0J56_RS15590 | upstream | IS110 family transposase | 1.30334 | 11.5755 |
| C0J56_RS15625 | C0J56_RS15625 | upstream | BAX inhibitor protein | 1.90005 | 12.5453 |
| C0J56_RS15680 | C0J56_RS15680 | upstream | NUDIX hydrolase | 1.32577 | 22.04553 |
| C0J56_RS15720 | valS | upstream | valine--tRNA ligase | 3.07327 | 8.27913 |
| C0J56_RS15725 | C0J56_RS15725 | upstream | oxidoreductase molybdopterin-binding protein | 1.2652 | 62.44039 |
| C0J56_RS15745 | C0J56_RS15745 | inside | two-component sensor histidine kinase | 1.3127 | 9.48939 |
| C0J56_RS15745 | C0J56_RS15745 | inside | two-component sensor histidine kinase | 1.28315 | 267.71826 |
| C0J56_RS15755 | C0J56_RS15755 | inside | SDR family oxidoreductase | 1.27193 | 6.39205 |
| C0J56_RS15965 | pedF | inside | cytochrome c-550 PedF | 1.28093 | 7.2533 |
| C0J56_RS16010 | C0J56_RS16010 | inside | transport permease protein | 1.28763 | 8.9517 |
| C0J56_RS16025 | C0J56_RS16025 | inside | glycerol metabolism activator | 1.27193 | 6.70955 |
| C0J56_RS16040 | C0J56_RS16040 | upstream | Transketolase domain-containing protein | 1.29661 | 7.59275 |
| C0J56_RS16055 | C0J56_RS16055 | upstream | Cyclic di-GMP phosphodiesterase response regulator RpfG | 1.43847 | 7.47614 |
| C0J56_RS16070 | C0J56_RS16070 | upstream | membrane protein | 1.62637 | 6.70955 |
| C0J56_RS16110 | C0J56_RS16110 | upstream | oxygen-independent coproporphyrinogen III oxidase | 3.06664 | 7.93041 |
| C0J56_RS16120 | C0J56_RS16120 | upstream | Mannuronan epimerase | 1.27642 | 16.66124 |
| C0J56_RS16130 | C0J56_RS16130 | inside | channel protein TolC | 2.31135 | 32.27947 |
| C0J56_RS16160 | C0J56_RS16160 | inside | spermidine/putrescine-binding protein | 1.55683 | 284.50027 |
| C0J56_RS16170 | C0J56_RS16170 | inside | 6-aminohexanoate-dimer hydrolase | 1.25623 | 6.92475 |
| C0J56_RS16180 | C0J56_RS16180 | inside | ABC transporter permease | 1.31231 | 125.29359 |
| C0J56_RS16195 | C0J56_RS16195 | inside | ABC transporter ATP-binding protein | 1.36391 | 25.97554 |
| C0J56_RS16200 | C0J56_RS16200 | inside | acyl-CoA synthetase | 1.34596 | 5.97941 |
| C0J56_RS16220 | C0J56_RS16220 | upstream | acyl-CoA synthetase | 1.34147 | 8.75442 |
| C0J56_RS16225 | C0J56_RS16225 | upstream | Caax amino terminal protease family protein | 1.3998 | 11.71194 |
| C0J56_RS16230 | C0J56_RS16230 | inside | Uncharacterized protein | 1.2847 | 10.64026 |
| C0J56_RS16275 | C0J56_RS16275 | upstream | thiol-disulfide oxidoreductase | 1.30782 | 10.37945 |
| C0J56_RS16310 | xylF | upstream | D-xylose ABC transporter substrate-binding protein | 1.30109 | 13.99012 |
| C0J56_RS16315 | xylG | inside | xylose import ATP-binding protein XylG | 1.30782 | 7.43674 |
| C0J56_RS16375 | C0J56_RS16375 | inside | Uncharacterized protein | 1.36391 | 8.51533 |
| C0J56_RS16380 | C0J56_RS16380 | inside | Uncharacterized protein | 1.69142 | 8.16221 |
| C0J56_RS16440 | C0J56_RS16440 | inside | Uncharacterized protein | 1.57253 | 8.51533 |
| C0J56_RS16545 | C0J56_RS16545 | upstream | diguanylate phosphodiesterase | 1.30109 | 11.71194 |
| C0J56_RS16565 | C0J56_RS16565 | upstream | Hsp20/alpha crystallin family protein | 1.66412 | 38.69583 |
| C0J56_RS16615 | C0J56_RS16615 | upstream | alcohol dehydrogenase | 1.83245 | 27.34765 |
| C0J56_RS16640 | C0J56_RS16640 | upstream | membrane protein | 1.77963 | 8.16221 |
| C0J56_RS16765 | C0J56_RS16765 | upstream | DNA-binding response regulator | 3.60717 | 35.95128 |
| C0J56_RS16915 | C0J56_RS16915 | upstream | TetR/AcrR family transcriptional regulator | 4.01096 | 58.85631 |
| C0J56_RS16940 | C0J56_RS16940 | upstream | Polyketide synthase | 2.75024 | 50.4909 |
| C0J56_RS16960 | C0J56_RS16960 | inside | DNA-binding response regulator | 3.02683 | 392.47363 |
| C0J56_RS17095 | C0J56_RS17095 | upstream | diaminobutyrate--2-oxoglutarate transaminase | 1.37063 | 497.13766 |
| C0J56_RS17100 | C0J56_RS17100 | inside | TonB-dependent receptor | 1.45588 | 200.78307 |
| C0J56_RS17110 | C0J56_RS17110 | inside | Peptide synthase | 1.64207 | 262.37115 |
| C0J56_RS17355 | C0J56_RS17355 | upstream | resistance-nodulation-cell division (RND) multidrug efflux membrane fusion protein MexC | 1.9538 | 12.12548 |
| C0J56_RS17370 | C0J56_RS17370 | inside | ureidoglycolate lyase | 1.33156 | 17.90033 |
| C0J56_RS17440 | phlD | upstream | type III polyketide synthase | 3.10019 | 33.78304 |
| C0J56_RS17455 | C0J56_RS17455 | inside | toxin | 1.67796 | 78.5349 |
| C0J56_RS17470 | C0J56_RS17470 | upstream | Uncharacterized protein | 1.85967 | 10.21443 |
| C0J56_RS17470 | C0J56_RS17470 | inside | Cable pili-associated 22 kDa adhesin protein | 1.44024 | 273.60281 |
| C0J56_RS17620 | C0J56_RS17620 | upstream | Anthranilate synthase, aminase component | 3.00373 | 37.32782 |
| C0J56_RS17670 | C0J56_RS17670 | upstream | LysR family transcriptional regulator | 2.81754 | 57.48356 |
| C0J56_RS17715 | C0J56_RS17715 | inside | multidrug resistance protein | 1.22424 | 17.38729 |
| C0J56_RS17720 | C0J56_RS17720 | inside | Urease accessory protein | 1.23048 | 252.72801 |
| C0J56_RS17735 | C0J56_RS17735 | inside | Amino acid ABC transporter substrate-binding protein | 1.30175 | 214.14868 |
| C0J56_RS17775 | C0J56_RS17775 | inside | urease subunit gamma/beta | 1.2441 | 4.75519 |
| C0J56_RS17800 | C0J56_RS17800 | upstream | ABC transporter ATP-binding protein | 6.94739 | 4.93906 |
| C0J56_RS17815 | C0J56_RS17815 | inside | glycine/betaine ABC transporter substrate-binding protein | 1.30782 | 8.65572 |
| C0J56_RS17830 | C0J56_RS17830 | upstream | Uncharacterized protein | 1.52766 | 5.79926 |
| C0J56_RS17840 | C0J56_RS17840 | upstream | MexE family multidrug efflux RND transporter periplasmic adaptor subunit | 1.30109 | 1458.25623 |
| C0J56_RS17845 | C0J56_RS17845 | inside | multidrug efflux RND transporter permease subunit | 7.52391 | 8.51533 |
| C0J56_RS17855 | C0J56_RS17855 | upstream | socitrate lyase/phosphoenolpyruvate mutase family protein | 10.53437 | 23.50947 |
| C0J56_RS17895 | C0J56_RS17895 | upstream | Polyketide cyclase | 4.93967 | 8.16221 |
| C0J56_RS17915 | C0J56_RS17915 | upstream | TetR/AcrR family transcriptional regulator | 8.23503 | 1679.3208 |
| C0J56_RS17975 | C0J56_RS17975 | upstream | TetR family transcriptional regulator | 1.72507 | 2959.80957 |
| C0J56_RS18070 | mupI | upstream | acyl-homoserine-lactone synthase | 3.61574 | 766.61017 |
| C0J56_RS18085 | mupR | upstream | LuxR family transcriptional regulator | 8.93221 | 1963.67957 |
| C0J56_RS18090 | mupW | inside | aromatic ring-hydroxylating dioxygenase subunit alpha | 1.35123 | 42.20542 |
| C0J56_RS18095 | mupV | inside | SDR family oxidoreductase | 9.96329 | 579.84644 |
| C0J56_RS18150 | mupN | inside | 4'-phosphopantetheinyl transferase superfamily protein | 1.52766 | 26115.35938 |
| C0J56_RS18160 | mupL | inside | alpha/beta hydrolase | 1.49515 | 16.66194 |
| C0J56_RS18160 | mupL | inside | alpha/beta hydrolase | 1.37397 | 2877.09595 |
| C0J56_RS18160 | mupL | inside | alpha/beta hydrolase | 1.27866 | 23.50947 |
| C0J56_RS18170 | mmpE | inside | FAD-binding protein | 15.43366 | 21.08847 |
| C0J56_RS18235 | mmpD | inside | SDR family NAD(P)-dependent oxidoreductase | 1.53664 | 7.03354 |
| C0J56_RS18250 | mmpB | upstream | KR domain-containing protein | 6.19814 | 13.16065 |
| C0J56_RS18910 | C0J56_RS18910 | inside | DNA translocase FtsK | 1.25379 | 5387.19238 |
| C0J56_RS18930 | infA | upstream | translation initiation factor IF-1 | 1.36596 | 24.25705 |
| C0J56_RS18945 | C0J56_RS18945 | upstream | cold shock domain protein CspD | 1.489 | 1184.93726 |
| C0J56_RS19015 | C0J56_RS19015 | upstream | NADH-quinone oxidoreductase subunit A | 2.86127 | 6.4799 |
| C0J56_RS19280 | C0J56_RS19280 | upstream | AsnC family transcriptional regulator | 2.831 | 12.12792 |
| C0J56_RS19295 | C0J56_RS19295 | upstream | tRNA-Pro | 3.45143 | 20.56556 |
| C0J56_RS19330 | infC | inside | translation initiation factor IF-3 | 3.88085 | 229.5289 |
| C0J56_RS19345 | C0J56_RS19345 | upstream | cold-shock protein | 1.52369 | 216.85939 |
| C0J56_RS19720 | C0J56_RS19720 | upstream | branched-chain amino acid ABC transporter permease | 8.92819 | 357.01501 |
| C0J56_RS19805 | C0J56_RS19805 | upstream | Uncharacterized protein | 4.03115 | 462.51236 |
| C0J56_RS19820 | C0J56_RS19820 | upstream | tRNA-Asp | 1.88613 | 23.84965 |
| C0J56_RS19955 | C0J56_RS19955 | upstream | TetR family transcriptional regulator | 3.68563 | 50.63049 |
| C0J56_RS20015 | C0J56_RS20015 | upstream | AI-2E family transporter | 2.61 | 2252.3064 |
| C0J56_RS20150 | C0J56_RS20150 | upstream | two-component system sensor histidine kinase/response regulator | 1.60677 | 502.58496 |
| C0J56_RS20315 | C0J56_RS20315 | inside | Uncharacterized protein | 2.02118 | 68.46468 |
| C0J56_RS20335 | C0J56_RS20335 | upstream | 23S ribosomal RNA | 14.48252 | 464.41464 |
| C0J56_RS20420 | C0J56_RS20420 | inside | PepSY domain-containing protein | 1.27498 | 225.20801 |
| C0J56_RS20580 | C0J56_RS20580 | upstream | Uncharacterized protein | 2.00997 | 35.18874 |
| C0J56_RS20620 | C0J56_RS20620 | upstream | Uncharacterized protein | 1.7475 | 78.28877 |
| C0J56_RS20625 | C0J56_RS20625 | upstream | Uncharacterized protein | 2.97233 | 4888.82764 |
| C0J56_RS20945 | acpP | upstream | acyl carrier protein | 1.51615 | 8.53226 |
| C0J56_RS20970 | C0J56_RS20970 | upstream | Large ribosomal RNA subunit accumulation protein YceD | 2.65089 | 15.81416 |
| C0J56_RS20995 | rne | upstream | Ribonuclease E | 1.52549 | 76.76137 |
| C0J56_RS21185 | C0J56_RS21185 | upstream | Flp fap pilin component | 1.47566 | 44.61494 |
| C0J56_RS21195 | C0J56_RS21195 | upstream | alcohol dehydrogenase | 8.37859 | 246.06061 |
| C0J56_RS21225 | C0J56_RS21225 | upstream | tRNA-Gly | 2.62013 | 26.96267 |
| C0J56_RS21425 | sucA | upstream | 2-oxoglutarate dehydrogenase subunit E1 | 2.38263 | 213.1841 |
| C0J56_RS21430 | sdhB | inside | succinate dehydrogenase iron-sulfur subunit | 1.43389 | 27.84405 |
| C0J56_RS21435 | sdhA | inside | succinate dehydrogenase flavoprotein subunit | 2.10255 | 20.34705 |
| C0J56_RS21715 | minE | inside | cell division topological specificity factor | 1.56068 | 2022.56262 |
| C0J56_RS21735 | C0J56_RS21735 | upstream | Uncharacterized protein | 1.62334 | 175.85231 |
| C0J56_RS21785 | C0J56_RS21785 | inside | Uncharacterized protein | 2.38664 | 165.36375 |
| C0J56_RS21785 | C0J56_RS21785 | upstream | Uncharacterized protein | 2.13084 | 20.95661 |
| C0J56_RS21815 | C0J56_RS21815 | upstream | Putative autotransporter | 10.94489 | 115.17775 |
| C0J56_RS21875 | C0J56_RS21875 | inside | peptidyl-prolyl cis-trans isomerase | 1.35172 | 32.60899 |
| C0J56_RS21900 | C0J56_RS21900 | upstream | tRNA-Asp | 1.75619 | 39.62607 |
| C0J56_RS21960 | C0J56_RS21960 | inside | Uncharacterized protein | 2.7362 | 152.87389 |
| C0J56_RS21960 | C0J56_RS21960 | inside | Uncharacterized protein | 2.55727 | 106.31994 |
| C0J56_RS21995 | C0J56_RS21995 | upstream | Uncharacterized protein | 1.59679 | 3148.56079 |
| C0J56_RS22085 | C0J56_RS22085 | upstream | peptidase P60 | 1.68268 | 531.15186 |
| C0J56_RS22160 | C0J56_RS22160 | upstream | Uncharacterized protein | 5.46184 | 13.42298 |
| C0J56_RS22395 | C0J56_RS22395 | inside | Uncharacterized protein | 1.5658 | 55.49204 |
| C0J56_RS22405 | C0J56_RS22405 | inside | Uncharacterized protein | 9.61239 | 184.89326 |
| C0J56_RS22455 | C0J56_RS22455 | upstream | DNA-binding protein | 2.42849 | 220.69679 |
| C0J56_RS22480 | C0J56_RS22480 | upstream | tRNA-Arg | 3.23515 | 34.8739 |
| C0J56_RS22580 | C0J56_RS22580 | upstream | ABC transporter substrate-binding protein（argT6） | 1.74029 | 46.83662 |
| C0J56_RS22605 | C0J56_RS22605 | upstream | Uncharacterized protein | 8.55133 | 1023.42206 |
| C0J56_RS22620 | C0J56_RS22620 | inside | transcriptional regulator | 1.82602 | 26.75587 |
| C0J56_RS22695 | C0J56_RS22695 | upstream | PepSY domain-containing protein | 1.43191 | 2547.38477 |
| C0J56_RS22725 | C0J56_RS22725 | upstream | aromatic amino acid transporter AroP | 1.81596 | 153.95413 |
| C0J56_RS22910 | C0J56_RS22910 | upstream | Uncharacterized protein | 3.90645 | 329.1348 |
| C0J56_RS22995 | glpF | upstream | aquaporin | 1.69756 | 50.78957 |
| C0J56_RS23005 | C0J56_RS23005 | upstream | DeoR family transcriptional regulator | 1.4978 | 2094.03809 |
| C0J56_RS23015 | glpD | upstream | glycerol-3-phosphate dehydrogenase | 3.3594 | 53.48183 |
| C0J56_RS23100 | C0J56_RS23100 | upstream | cold-shock protein | 2.58968 | 19.5025 |
| C0J56_RS23160 | ybgF | inside | Cell division coordinator CpoB | 1.9355 | 62.07924 |
| C0J56_RS23250 | C0J56_RS23250 | upstream | porin | 1.9465 | 529.59314 |
| C0J56_RS23335 | C0J56_RS23335 | upstream | transcriptional regulator | 3.61152 | 47.83582 |
| C0J56_RS23515 | metE | inside | 5-methyltetrahydropteroyltriglutamate--homocystei ne methyltransferase | 1.76497 | 26.26139 |
| C0J56_RS23605 | C0J56_RS23605 | upstream | Uncharacterized protein | 6.35068 | 499.65002 |
| C0J56_RS23660 | C0J56_RS23660 | upstream | Uncharacterized protein | 2.84817 | 203.72005 |
| C0J56_RS23705 | C0J56_RS23705 | upstream | Uncharacterized protein | 3.00711 | 90.56843 |
| C0J56_RS23910 | rlmF | upstream | ribosomal RNA large subunit methyltransferase F | 1.36736 | 74.54491 |
| C0J56_RS23990 | rhlB | inside | ATP-dependent RNA helicase RhlB | 7.56205 | 453.98965 |
| C0J56_RS23995 | C0J56_RS23995 | inside | Rhs family protein / insecticidal toxin protein | 7.05058 | 59.78823 |
| C0J56_RS24000 | C0J56_RS24000 | upstream | Rhs family protein / insecticidal toxin protein | 8.96633 | 1239.22461 |
| C0J56_RS24005 | C0J56_RS24005 | upstream | Rhs family protein / insecticidal toxin protein | 7.31304 | 229.55154 |
| C0J56_RS24015 | C0J56_RS24015 | inside | Uncharacterized protein | 10.6757 | 278.39426 |
| C0J56_RS24020 | C0J56_RS24020 | inside | Uncharacterized protein | 11.08173 | 13.21108 |
| C0J56_RS24095 | algD | upstream | GDP-mannose 6-dehydrogenase | 4.6595 | 1694.25305 |
| C0J56_RS24245 | lpxC | upstream | UDP-3-O-acyl-N-acetylglucosamine deacetylase | 1.33838 | 1497.16431 |
| C0J56_RS24310 | ftsL | inside | cell division protein FtsL | 1.48975 | 2268.49585 |
| C0J56_RS24320 | mraZ | upstream | Transcriptional regulator MraZ | 1.87876 | 1597.43811 |
| C0J56_RS24365 | C0J56_RS24365 | inside | cytochrome c1 | 1.39065 | 3024.44409 |
| C0J56_RS24370 | C0J56_RS24370 | inside | cytochrome b | 6.15961 | 3212.1543 |
| C0J56_RS24625 | C0J56_RS24625 | upstream | Uncharacterized protein | 4.31128 | 740.62604 |
| C0J56_RS24645 | C0J56_RS24645 | upstream | Colicin/Pyocin-S2 | 7.76394 | 12.19015 |
| C0J56_RS24650 | C0J56_RS24650 | inside | pyocin-S2 immunity protein | 9.16374 | 25.76393 |
| C0J56_RS24685 | C0J56_RS24685 | inside | Uncharacterized protein | 1.4935 | 71.24523 |
| C0J56_RS24865 | C0J56_RS24865 | upstream | Uncharacterized protein | 3.3462 | 14.5066 |
| C0J56_RS24875 | C0J56_RS24875 | upstream | Uncharacterized protein | 2.75368 | 1310.61426 |
| C0J56_RS24935 | C0J56_RS24935 | upstream | short-chain dehydrogenase | 3.03827 | 657.84106 |
| C0J56_RS24990 | C0J56_RS24990 | upstream | tRNA-Gln | 3.2458 | 1773.9115 |
| C0J56_RS25170 | C0J56_RS25170 | upstream | paraquat-inducible protein A | 16.60465 | 2352.78345 |
| C0J56_RS25335 | C0J56_RS25335 | upstream | poly(A) polymerase I | 1.76118 | 23.84835 |
| C0J56_RS25420 | C0J56_RS25420 | upstream | Uncharacterized protein | 3.79209 | 353.70459 |
| C0J56_RS25455 | C0J56_RS25455 | upstream | tRNA-Pro | 2.23702 | 219.82709 |
| C0J56_RS25490 | C0J56_RS25490 | inside | outer membrane protein assembly factor BamD | 1.68374 | 322.79434 |
| C0J56_RS25620 | rpsT | upstream | 30S ribosomal protein S20 | 1.58707 | 329.41528 |
| C0J56_RS25645 | rplU | upstream | 50S ribosomal protein L21 | 2.62516 | 6016.24756 |
| C0J56_RS25735 | C0J56_RS25735 | upstream | Uncharacterized protein | 4.00654 | 49.1337 |
| C0J56_RS25785 | katB | inside | catalase | 2.60667 | 473.97971 |
| C0J56_RS25795 | C0J56_RS25795 | upstream | ferredoxin--NADP(+) reductase | 2.48329 | 112.00813 |
| C0J56_RS25970 | C0J56_RS25970 | upstream | Uncharacterized protein | 1.72283 | 40.03628 |
| C0J56_RS26020 | wbpA | upstream | UDP-N-acetyl-D-glucosamine 6-dehydrogenase | 1.52991 | 30.22926 |
| C0J56_RS26115 | C0J56_RS26115 | upstream | Uncharacterized protein | 3.82028 | 181.71718 |
| C0J56_RS26390 | C0J56_RS26390 | upstream | transcriptional regulator | 2.55957 | 772.17334 |
| C0J56_RS26395 | C0J56_RS26395 | upstream | Uncharacterized protein | 2.11316 | 505.07532 |
| C0J56_RS26405 | C0J56_RS26405 | inside | OmpA family protein | 3.18544 | 173.34369 |
| C0J56_RS26690 | C0J56_RS26690 | upstream | outer membrane protein W | 9.44251 | 150.98273 |
| C0J56_RS26950 | rplB | upstream | 50S ribosomal protein L2 | 3.64979 | 41.96733 |
| C0J56_RS26985 | fusA | inside | elongation factor G | 2.27916 | 23.69562 |
| C0J56_RS27040 | C0J56_RS27040 | upstream | tRNA-Trp | 4.52095 | 446.68088 |
| C0J56_RS27085 | C0J56_RS27085 | upstream | 23S ribosomal RNA | 15.60191 | 164.66983 |
| C0J56_RS27120 | C0J56_RS27120 | upstream | Cell wall endopeptidase, family M23/M37 | 1.73853 | 91.26173 |
| C0J56_RS27165 | coq7 | upstream | 2-nonaprenyl-3-methyl-6-methoxy-1,4-benzoquinol hydroxylase | 1.45492 | 292.53333 |
| C0J56_RS27360 | rpsU | upstream | 30S ribosomal protein S21 | 2.333 | 2858.96777 |
| C0J56_RS27370 | rpoD | upstream | RNA polymerase sigma factor RpoD | 1.88883 | 403.12436 |
| C0J56_RS27395 | C0J56_RS27395 | inside | tryptophan synthase subunit alpha | 1.40353 | 116.61417 |
| C0J56_RS27625 | C0J56_RS27625 | upstream | cold shock domain-containing protein | 1.65553 | 734.85223 |
| C0J56_RS27690 | C0J56_RS27690 | upstream | Uncharacterized protein | 1.6488 | 5476.53174 |
| C0J56_RS27790 | C0J56_RS27790 | upstream | alginate lyase family protein | 5.13708 | 43.64438 |
| C0J56_RS28380 | rpoH | upstream | RNA polymerase sigma factor RpoH | 1.85294 | 18.30431 |
| C0J56_RS28435 | C0J56_RS28435 | inside | putative coniferyl aldehyde dehydrogenase | 1.27342 | 125.34921 |
| C0J56_RS28550 | C0J56_RS28550 | upstream | sigma-54-dependent Fis family transcriptional regulator | 1.40204 | 61.04649 |
| C0J56_RS28710 | C0J56_RS28710 | upstream | Auxin Efflux Carrier /AEC family transporter | 9.44863 | 14.26783 |
| C0J56_RS28955 | C0J56_RS28955 | upstream | Efflux RND transporter periplasmic adaptor subunit | 1.42475 | 35.09508 |
| C0J56_RS29000 | rho | upstream | transcription termination factor Rho | 1.63345 | 34.43646 |
| C0J56_RS29180 | C0J56_RS29180 | upstream | TIGR02647 family protein/DNA-binding protein | 1.41999 | 828.48389 |
| C0J56_RS29395 | rpmB | upstream | 50S ribosomal protein L28 | 2.47636 | 56.67374 |
| C0J56_RS29440 | gltS | upstream | sodium/glutamate symporter | 2.75249 | 7.29664 |
| C0J56_RS29465 | rpoZ | upstream | DNA-directed RNA polymerase subunit omega | 2.31602 | 14.13851 |
| C0J56_RS29520 | C0J56_RS29520 | inside | Uncharacterized protein | 1.7834 | 2475.87378 |
| C0J56_RS29540 | C0J56_RS29540 | upstream | Uncharacterized protein | 3.49949 | 16.40737 |
| C0J56_RS29610 | tagH | upstream | type VI secretion system-associated FHA domain protein TagH | 1.59496 | 33.44014 |
| C0J56_RS30010 | C0J56_RS30010 | inside | ATP synthase epsilon chain | 1.31945 | 15.34897 |
| C0J56_RS30020 | atpG | inside | ATP synthase gamma chain | 1.58003 | 150.31313 |
| C0J56_RS30030 | atpH | inside | ATP synthase subunit delta | 2.0324 | 201.22353 |
| C0J56_RS30095 | rnpA | upstream | ribonuclease P protein component | 3.15852 | 127.59269 |
| C0J56_RS30115 | C0J56_RS30115 | upstream | Uncharacterized protein | 1.88879 | 48.58558 |
| C0J56_RS30150 | C0J56_RS30150 | upstream | Uncharacterized protein | 8.72172 | 366.00241 |
| C0J56_RS30295 | C0J56_RS30295 | inside | Uncharacterized protein | 1.74214 | 29.35968 |
| C0J56_RS30305 | C0J56_RS30305 | upstream | Uncharacterized protein | 1.42672 | 9.16944 |
| C0J56_RS30335 | C0J56_RS30335 | upstream | Uncharacterized protein | 4.95762 | 29.26035 |
| C0J56_RS30545 | C0J56_RS30545 | upstream | Transcriptional regulator, AraC family | 1.24688 | 79.82843 |
| C0J56_RS30760 | C0J56_RS30760 | inside | CdiI_2 domain-containing protein | 3.86571 | 286.50662 |

**Sample 3 vs input**

| geneID  (target genes) | Gene name | Position | Gene description | Fold_enrichment | -log10(pvalue) |
| --- | --- | --- | --- | --- | --- |
| C0J56_RS00005 | dnaA | upstream | chromosomal replication initiator protein DnaA | 1.78435 | 46.13997 |
| C0J56_RS00120 | C0J56_RS00120 | upstream | ATPase | 13.98776 | 4087.73999 |
| C0J56_RS00200 | C0J56_RS00200 | upstream | Cytochrome c5 | 2.90288 | 204.40048 |
| C0J56_RS00210 | C0J56_RS00210 | upstream | thiol:disulfide interchange protein DsbA | 1.62785 | 29.90252 |
| C0J56_RS00425 | C0J56_RS00425 | upstream | AAA family ATPase | 11.45124 | 2986.27905 |
| C0J56_RS00455 | C0J56_RS00455 | upstream | DUF4065 domain-containing protein | 5.32237 | 783.29639 |
| C0J56_RS00575 | C0J56_RS00575 | inside | membrane protein | 1.58556 | 25.51917 |
| C0J56_RS00595 | C0J56_RS00595 | upstream | Uncharacterized protein | 9.59909 | 2241.82104 |
| C0J56_RS00625 | C0J56_RS00625 | upstream | LysR family transcriptional regulator | 2.98175 | 218.96443 |
| C0J56_RS00775 | aspA | inside | aspartate ammonia-lyase | 1.99207 | 65.70437 |
| C0J56_RS00865 | C0J56_RS00865 | upstream | HlyC/CorC family transporter | 1.76819 | 41.46943 |
| C0J56_RS00985 | C0J56_RS00985 | upstream | Uncharacterized protein | 14.41772 | 4282.66455 |
| C0J56_RS00995 | C0J56_RS00995 | upstream | Succinate CoA transferase | 1.81907 | 46.57463 |
| C0J56_RS01035 | C0J56_RS01035 | upstream | 16S ribosomal RNA | 38.69404 | 17726.8457 |
| C0J56_RS01275 | C0J56_RS01275 | upstream | Heme oxygenase-like protein | 4.92039 | 671.07416 |
| C0J56_RS01315 | C0J56_RS01315 | inside | LapA family giant adhesin | 1.33521 | 10.19002 |
| C0J56_RS01515 | C0J56_RS01515 | upstream | RNA pyrophosphohydrolase | 1.32805 | 8.539 |
| C0J56_RS01555 | C0J56_RS01555 | inside | FAD-binding oxidoreductase | 2.36352 | 115.17287 |
| C0J56_RS01620 | C0J56_RS01620 | upstream | Uncharacterized protein | 4.54386 | 571.04083 |
| C0J56_RS01925 | C0J56_RS01925 | upstream | peptide ABC transporter substrate-binding protein | 2.95376 | 213.75534 |
| C0J56_RS02010 | C0J56_RS02010 | upstream | Uncharacterized protein | 2.91051 | 205.79405 |
| C0J56_RS02145 | glnA1 | upstream | glutamine synthetase | 2.21341 | 93.8885 |
| C0J56_RS02160 | typA | upstream | GTP-binding protein | 1.46034 | 16.16412 |
| C0J56_RS02395 | PhaI | upstream | Phasin family protein | 1.90303 | 55.52401 |
| C0J56_RS02460 | C0J56_RS02460 | upstream | primosomal protein N' | 2.52889 | 140.49806 |
| C0J56_RS02470 | C0J56_RS02470 | inside | malate dehydrogenase | 1.49087 | 18.21703 |
| C0J56_RS02525 | gltB | upstream | glutamate synthase large subunit | 1.52904 | 20.93189 |
| C0J56_RS02540 | gbpA | upstream | GlcNAc-binding protein A | 3.26924 | 274.99515 |
| C0J56_RS02615 | C0J56_RS02615 | upstream | Uncharacterized protein | 6.00166 | 984.53253 |
| C0J56_RS02620 | C0J56_RS02620 | inside | lipoprotein | 3.57708 | 339.77448 |
| C0J56_RS02625 | C0J56_RS02625 | upstream | Uncharacterized protein | 8.64758 | 1882.43994 |
| C0J56_RS02845 | C0J56_RS02845 | upstream | Uncharacterized protein | 4.84916 | 651.76062 |
| C0J56_RS02890 | aceE | upstream | pyruvate dehydrogenase E1 component | 1.46798 | 16.66748 |
| C0J56_RS02945 | C0J56_RS02945 | upstream | carbamoyltransferase | 1.72668 | 38.8709 |
| C0J56_RS03115 | C0J56_RS03115 | upstream | Lipoprotein | 1.54353 | 22.94707 |
| C0J56_RS03210 | hflX | inside | GTPase HflX | 2.38859 | 122.17709 |
| C0J56_RS03215 | hflK | upstream | protease modulator HflK | 1.26645 | 5.89284 |
| C0J56_RS03220 | hflC | inside | Modulator of FtsH protease HflC | 1.28797 | 6.90077 |
| C0J56_RS03225 | C0J56_RS03225 | upstream | ATP phosphoribosyltransferase regulatory subunit | 1.64656 | 31.34211 |
| C0J56_RS03245 | C0J56_RS03245 | upstream | tRNA-Leu | 6.41636 | 1113.9436 |
| C0J56_RS03280 | C0J56_RS03280 | inside | Uncharacterized protein | 2.64539 | 168.32593 |
| C0J56_RS03340 | azu | upstream | Azurin | 2.136 | 84.80834 |
| C0J56_RS03640 | C0J56_RS03640 | upstream | Toxin A | 5.72446 | 939.05103 |
| C0J56_RS03645 | C0J56_RS03645 | upstream | UPF0056 inner membrane protein | 5.81594 | 928.14233 |
| C0J56_RS03685 | dusB | upstream | tRNA-dihydrouridine synthase B | 1.60767 | 28.13058 |
| C0J56_RS03705 | accC | inside | acetyl-CoA carboxylase biotin carboxylase subunit | 1.23805 | 5.25835 |
| C0J56_RS03745 | C0J56_RS03745 | upstream | translation initiation factor | 1.78773 | 45.80294 |
| C0J56_RS03980 | C0J56_RS03980 | upstream | 16S ribosomal RNA | 40.70901 | 65.63268 |
| C0J56_RS04190 | C0J56_RS04190 | upstream | proline iminopeptidase | 1.52596 | 18993.63672 |
| C0J56_RS04220 | C0J56_RS04220 | inside | addiction module antidote protein, HigA family | 1.41722 | 21.49762 |
| C0J56_RS04375 | ssrA | upstream | transfer-messenger RNA | 4.41156 | 13.47036 |
| C0J56_RS04415 | smpB | inside | SsrA-binding protein | 1.98919 | 537.13501 |
| C0J56_RS04435 | C0J56_RS04435 | upstream | outer membrane protein assembly factor BamE | 2.5426 | 73.07957 |
| C0J56_RS04455 | dnaK | inside | chaperone protein DnaK | 1.59779 | 149.43663 |
| C0J56_RS04470 | carA | upstream | carbamoyl-phosphate synthase small chain | 4.50377 | 26.84402 |
| C0J56_RS04505 | folP | upstream | dihydropteroate synthase | 1.63883 | 598.18549 |
| C0J56_RS04525 | C0J56_RS04525 | upstream | tRNA-Leu | 7.36284 | 31.57315 |
| C0J56_RS04560 | rpsO | upstream | 30S ribosomal protein S15 | 1.33509 | 1514.01099 |
| C0J56_RS04735 | C0J56_RS04735 | inside | ABC transporter substrate-binding protein | 1.50343 | 10.16745 |
| C0J56_RS04785 | C0J56_RS04785 | upstream | Uncharacterized protein | 3.76862 | 20.91061 |
| C0J56_RS04850 | gatC | upstream | Glutamyl-tRNA(Gln) amidotransferase subunit C | 2.45391 | 410.60196 |
| C0J56_RS04945 | raiA | upstream | ribosomal subunit interface protein | 1.69213 | 147.27551 |
| C0J56_RS04950 | RpoN | upstream | RNA polymerase sigma-54 factor | 1.81006 | 38.68144 |
| C0J56_RS05005 | C0J56_RS05005 | upstream | BolA family transcriptional regulator | 1.49412 | 51.20888 |
| C0J56_RS05040 | C0J56_RS05040 | upstream | UPF0016 family membrane protein | 1.80407 | 21.03367 |
| C0J56_RS05175 | C0J56_RS05175 | upstream | Reverse transcriptase domain-containing protein | 14.66705 | 54.64911 |
| C0J56_RS05215 | C0J56_RS05215 | upstream | tRNA-Leu | 2.74326 | 4396.71338 |
| C0J56_RS05250 | suhB | upstream | inositol monophosphatase | 1.7357 | 179.90918 |
| C0J56_RS05265 | iscR | upstream | Fe-S cluster assembly transcriptional regulator IscR | 2.03658 | 43.10324 |
| C0J56_RS05310 | rlmN | inside | Dual-specificity RNA methyltransferase RlmN | 1.26821 | 82.1773 |
| C0J56_RS05395 | guaB | upstream | inosine-5'-monophosphate dehydrogenase | 2.44036 | 7.19965 |
| C0J56_RS05400 | guaA | upstream | GMP synthase [glutamine-hydrolyzing] | 1.33835 | 148.71645 |
| C0J56_RS05495 | ccsA | upstream | cytochrome c assembly protein | 1.62013 | 12.07949 |
| C0J56_RS05510 | rimM | inside | ribosome maturation factor RimM | 4.67184 | 35.49825 |
| C0J56_RS05885 | rpsB | upstream | 30S ribosomal protein S2 | 5.34771 | 696.89404 |
| C0J56_RS05930 | C0J56_RS05930 | inside | Outer membrane chaperone skp | 2.44404 | 909.0777 |
| C0J56_RS05975 | C0J56_RS05975 | upstream | CTP synthase | 2.18436 | 145.47009 |
| C0J56_RS06000 | C0J56_RS06000 | upstream | LysR family transcriptional regulator | 2.03175 | 107.26468 |
| C0J56_RS06040 | rpoS | upstream | RNA polymerase sigma factor RpoS | 1.57589 | 85.27361 |
| C0J56_RS06050 | C0J56_RS06050 | upstream | Ferredoxin 1 | 1.48718 | 28.32799 |
| C0J56_RS06260 | recA | inside | protein RecA | 1.25455 | 20.03682 |
| C0J56_RS06300 | C0J56_RS06300 | upstream | LysR family transcriptional regulator | 2.96551 | 6.49512 |
| C0J56_RS06365 | C0J56_RS06365 | upstream | tRNA-Met | 2.38946 | 270.00174 |
| C0J56_RS06405 | C0J56_RS06405 | upstream | cold shock protein CapB | 3.15294 | 139.50505 |
| C0J56_RS06455 | C0J56_RS06455 | upstream | acyltransferase | 5.57415 | 291.15527 |
| C0J56_RS06685 | arcA | inside | arginine deiminase | 4.29463 | 998.52832 |
| C0J56_RS06920 | alaC | upstream | Alanine transaminase | 1.83032 | 600.93262 |
| C0J56_RS06980 | C0J56_RS06980 | inside | amino acid transporter LysE | 1.32312 | 33.2653 |
| C0J56_RS07020 | C0J56_RS07020 | upstream | Uncharacterized protein | 1.86338 | 63.19754 |
| C0J56_RS07125 | C0J56_RS07125 | upstream | secretion protein HlyD | 5.68151 | 11.30465 |
| C0J56_RS07295 | C0J56_RS07295 | upstream | Uncharacterized protein | 3.4517 | 2051.28052 |
| C0J56_RS07315 | C0J56_RS07315 | upstream | tRNA-Gly | 2.19043 | 60.04724 |
| C0J56_RS07460 | C0J56_RS07460 | inside | sigma factor AlgU negative regulatory protein | 2.37368 | 1018.36292 |
| C0J56_RS07500 | dapA | upstream | 4-hydroxy-tetrahydrodipicolinate synthase | 1.35549 | 324.82526 |
| C0J56_RS07510 | C0J56_RS07510 | inside | MBL fold metallo-hydrolase | 1.36124 | 102.60326 |
| C0J56_RS07520 | C0J56_RS07520 | upstream | tRNA-Ser | 2.97815 | 160.87325 |
| C0J56_RS07595 | C0J56_RS07595 | upstream | phosphoethanolamine transferase | 2.05879 | 12.25161 |
| C0J56_RS07705 | C0J56_RS07705 | upstream | MFS transporter | 1.70106 | 13.95376 |
| C0J56_RS07760 | C0J56_RS07760 | upstream | DMT family transporter | 1.95488 | 256.76907 |
| C0J56_RS07875 | C0J56_RS07875 | upstream | Uncharacterized protein | 8.16165 | 87.67565 |
| C0J56_RS07875 | C0J56_RS07875 | inside | Uncharacterized protein | 5.11402 | 46.91179 |
| C0J56_RS07895 | C0J56_RS07895 | upstream | IS4 family transposase | 10.76177 | 75.73511 |
| C0J56_RS07960 | C0J56_RS07960 | upstream | Uncharacterized protein | 3.81126 | 772.21417 |
| C0J56_RS08045 | C0J56_RS08045 | inside | Uncharacterized protein | 4.9465 | 1705.68567 |
| C0J56_RS08130 | C0J56_RS08130 | inside | Putative NAD(P)H nitroreductase | 1.37806 | 2702.69189 |
| C0J56_RS08140 | C0J56_RS08140 | upstream | pterin-4-alpha-carbinolamine dehydratase | 1.47895 | 406.17383 |
| C0J56_RS08220 | ntrC1 | upstream | sigma-54-dependent Fis family transcriptional regulator | 4.52807 | 749.39984 |
| C0J56_RS08355 | cheY | upstream | chemotaxis protein CheY | 1.42943 | 14.27017 |
| C0J56_RS08470 | C0J56_RS08470 | upstream | Uncharacterized protein | 2.18791 | 21.05137 |
| C0J56_RS08605 | gyrA | upstream | DNA gyrase subunit A | 1.7789 | 630.52191 |
| C0J56_RS08635 | rpsA | upstream | 30S ribosomal protein S1 | 5.00149 | 18.15582 |
| C0J56_RS08650 | C0J56_RS08650 | inside | LapA family protein | 12.97264 | 109.53655 |
| C0J56_RS08800 | C0J56_RS08800 | upstream | methyl-accepting chemotaxis protein | 1.98444 | 51.95082 |
| C0J56_RS08820 | C0J56_RS08820 | upstream | Class I SAM-dependent methyltransferase | 2.43836 | 869.12347 |
| C0J56_RS08860 | C0J56_RS08860 | inside | Acetyltransferase-like protein | 3.33171 | 3636.60693 |
| C0J56_RS08905 | C0J56_RS08905 | inside | Efp Translation elongation factor P | 1.38977 | 76.41113 |
| C0J56_RS08960 | C0J56_RS08960 | inside | ATP-dependent RNA helicase DeaD | 2.42978 | 150.18129 |
| C0J56_RS08980 | htpX | inside | Protease HtpX | 1.25429 | 330.72729 |
| C0J56_RS09030 | C0J56_RS09030 | inside | hybrid sensor histidine kinase/response regulator | 3.63415 | 13.5798 |
| C0J56_RS09120 | C0J56_RS09120 | upstream | Sensor histidine kinase | 2.31795 | 151.7511 |
| C0J56_RS09295 | C0J56_RS09295 | upstream | porin | 3.14447 | 6.85337 |
| C0J56_RS09420 | C0J56_RS09420 | upstream | Uncharacterized protein | 3.16642 | 397.87531 |
| C0J56_RS09510 | C0J56_RS09510 | upstream | membrane protein | 1.6713 | 125.67268 |
| C0J56_RS09550 | zipA | upstream | cell division protein ZipA | 1.80891 | 289.00143 |
| C0J56_RS09575 | C0J56_RS09575 | upstream | Uncharacterized protein | 3.3698 | 271.20724 |
| C0J56_RS09630 | C0J56_RS09630 | upstream | adenine phosphoribosyltransferase | 2.01504 | 38.00616 |
| C0J56_RS09645 | C0J56_RS09645 | inside | Sulfite exporter TauE/SafE family protein | 2.64997 | 50.91897 |
| C0J56_RS09670 | C0J56_RS09670 | inside | Type II toxin-antitoxin system VapC family toxin | 2.30769 | 321.96017 |
| C0J56_RS09720 | C0J56_RS09720 | upstream | Alpha/beta hydrolase | 2.32814 | 82.31133 |
| C0J56_RS09825 | C0J56_RS09825 | inside | Uncharacterized protein | 1.55876 | 209.20068 |
| C0J56_RS09865 | gap1 | upstream | glyceraldehyde-3-phosphate dehydrogenase | 1.93667 | 129.78458 |
| C0J56_RS09945 | C0J56_RS09945 | inside | fused response regulator/phosphatase | 1.55485 | 146.74745 |
| C0J56_RS09970 | C0J56_RS09970 | upstream | Uncharacterized protein | 8.85111 | 26.59986 |
| C0J56_RS10025 | C0J56_RS10025 | inside | HNH endonuclease | 14.37702 | 69.76194 |
| C0J56_RS10210 | C0J56_RS10210 | inside | Abasic site processing protein | 2.9156 | 27.35426 |
| C0J56_RS10225 | C0J56_RS10225 | upstream | Uncharacterized protein | 12.72332 | 1957.87366 |
| C0J56_RS10440 | C0J56_RS10440 | upstream | tRNA-Arg | 14.50422 | 4264.11865 |
| C0J56_RS10480 | C0J56_RS10480 | upstream | DNA-binding protein HU-beta | 4.43701 | 206.72481 |
| C0J56_RS10600 | C0J56_RS10600 | upstream | Zinc carboxypeptidase domain protein | 1.7994 | 3527.83813 |
| C0J56_RS10640 | C0J56_RS10640 | upstream | Uncharacterized protein | 2.5976 | 4322.15234 |
| C0J56_RS10675 | C0J56_RS10675 | inside | Uncharacterized protein | 7.86849 | 543.60321 |
| C0J56_RS10935 | C0J56_RS10935 | upstream | Uncharacterized protein | 3.7827 | 51.47334 |
| C0J56_RS10935 | C0J56_RS10935 | inside | Uncharacterized protein | 1.6997 | 164.40305 |
| C0J56_RS11055 | C0J56_RS11055 | upstream | General stress protein | 2.61269 | 1690.93604 |
| C0J56_RS11235 | C0J56_RS11235 | upstream | transcriptional regulator | 1.56756 | 36.69497 |
| C0J56_RS11240 | C0J56_RS11240 | upstream | Methionyl-tRNA formyltransferase | 2.64787 | 480.18204 |
| C0J56_RS11305 | C0J56_RS11305 | inside | cobalamin biosynthesis protein CbiG | 1.78789 | 157.77991 |
| C0J56_RS11475 | C0J56_RS11475 | upstream | carbon-nitrogen hydrolase family protein | 2.09958 | 30.04703 |
| C0J56_RS11485 | gacA | upstream | response regulator GacA | 1.55355 | 179.44379 |
| C0J56_RS11500 | C0J56_RS11500 | upstream | tRNA-Gly | 3.07588 | 49.84687 |
| C0J56_RS11590 | C0J56_RS11590 | inside | LysR family transcriptional regulator | 1.44762 | 82.94879 |
| C0J56_RS11915 | C0J56_RS11915 | upstream | LysR family transcriptional regulator | 1.5214 | 23.69395 |
| C0J56_RS12005 | C0J56_RS12005 | upstream | ABC transporter permease | 5.4384 | 236.81122 |
| C0J56_RS12215 | C0J56_RS12215 | upstream | transcriptional regulator | 3.52111 | 15.34012 |
| C0J56_RS12235 | hppD | upstream | 4-hydroxyphenylpyruvate dioxygenase | 1.79872 | 20.37583 |
| C0J56_RS12350 | C0J56_RS12350 | inside | branched-chain-amino-acid aminotransferase | 1.38831 | 832.44299 |
| C0J56_RS12370 | C0J56_RS12370 | upstream | 2-oxoisovalerate dehydrogenase subunit alpha | 3.47494 | 327.64807 |
| C0J56_RS12450 | hydA | upstream | D-hydantoinase/dihydropyrimidinase | 2.62557 | 44.50311 |
| C0J56_RS12570 | C0J56_RS12570 | upstream | methyl-accepting chemotaxis protein | 1.82161 | 12.71262 |
| C0J56_RS12645 | C0J56_RS12645 | upstream | Uncharacterized protein | 10.14354 | 321.26816 |
| C0J56_RS12655 | C0J56_RS12655 | upstream | Rhs family protein / insecticidal toxin protein | 9.30652 | 156.16585 |
| C0J56_RS12770 | C0J56_RS12770 | upstream | Uncharacterized protein | 3.95616 | 46.83638 |
| C0J56_RS12780 | C0J56_RS12780 | inside | EamA domain-containing protein | 6.7242 | 2454.80835 |
| C0J56_RS12820 | C0J56_RS12820 | upstream | Antibiotic biosynthesis monooxygenase | 12.60883 | 2129.52466 |
| C0J56_RS12855 | acnB | upstream | aconitate hydratase B | 1.52405 | 425.72534 |
| C0J56_RS12865 | C0J56_RS12865 | upstream | Universal stress protein | 1.89836 | 1212.98486 |
| C0J56_RS12915 | C0J56_RS12915 | upstream | Uncharacterized protein | 6.32477 | 3478.177 |
| C0J56_RS12955 | C0J56_RS12955 | upstream | lytic transglycosylase | 1.90575 | 20.66858 |
| C0J56_RS13200 | C0J56_RS13200 | inside | Uncharacterized protein | 2.37878 | 55.94707 |
| C0J56_RS13530 | C0J56_RS13530 | upstream | 2-hydroxymuconate tautomerase | 5.10612 | 1084.95703 |
| C0J56_RS13545 | C0J56_RS13545 | upstream | Uncharacterized protein | 4.30471 | 56.41026 |
| C0J56_RS13745 | narX | upstream | histidine kinase | 3.60224 | 117.4301 |
| C0J56_RS13950 | C0J56_RS13950 | upstream | NAD(P)/FAD-dependent oxidoreductase | 1.3942 | 722.25153 |
| C0J56_RS14025 | C0J56_RS14025 | upstream | ABC transporter permease | 3.51857 | 510.24185 |
| C0J56_RS14245 | zwf1 | upstream | glucose-6-phosphate 1-dehydrogenase | 1.62497 | 347.73004 |
| C0J56_RS14260 | C0J56_RS14260 | upstream | Uncharacterized protein | 1.48324 | 12.08724 |
| C0J56_RS14355 | C0J56_RS14355 | upstream | ABC-F family ATPase | 1.84451 | 327.10071 |
| C0J56_RS14390 | C0J56_RS14390 | upstream | Uncharacterized protein | 1.78345 | 17.69384 |
| C0J56_RS14490 | C0J56_RS14490 | upstream | Uncharacterized protein | 2.41695 | 49.21824 |
| C0J56_RS14715 | C0J56_RS14715 | upstream | Uncharacterized protein | 4.30471 | 42.97501 |
| C0J56_RS14745 | C0J56_RS14745 | upstream | Uncharacterized protein | 2.73242 | 123.14643 |
| C0J56_RS14760 | C0J56_RS14760 | upstream | serralysin family metalloprotease | 4.99672 | 510.24185 |
| C0J56_RS14770 | C0J56_RS14770 | upstream | ABC transporter ATP-binding protein | 4.6558 | 174.18663 |
| C0J56_RS15290 | C0J56_RS15290 | upstream | AMP-binding protein | 2.14472 | 691.9635 |
| C0J56_RS15460 | C0J56_RS15460 | upstream | transcriptional regulator | 1.52395 | 600.24353 |
| C0J56_RS15625 | C0J56_RS15625 | upstream | BAX inhibitor protein | 2.09384 | 84.71865 |
| C0J56_RS15720 | valS | upstream | valine--tRNA ligase | 3.22599 | 20.56041 |
| C0J56_RS15795 | C0J56_RS15795 | upstream | tRNA-Leu | 4.39121 | 78.16811 |
| C0J56_RS16090 | C0J56_RS16090 | upstream | Uncharacterized protein | 1.66388 | 266.28043 |
| C0J56_RS16110 | C0J56_RS16110 | upstream | oxygen-independent coproporphyrinogen III oxidase | 1.44795 | 531.97803 |
| C0J56_RS16380 | C0J56_RS16380 | inside | Uncharacterized protein | 1.85723 | 31.78893 |
| C0J56_RS16760 | C0J56_RS16760 | upstream | two-component sensor | 4.90513 | 17.247 |
| C0J56_RS16915 | C0J56_RS16915 | upstream | TetR/AcrR family transcriptional regulator | 4.98654 | 50.56216 |
| C0J56_RS16940 | C0J56_RS16940 | upstream | Polyketide synthase | 2.77313 | 666.92126 |
| C0J56_RS16960 | C0J56_RS16960 | inside | DNA-binding response regulator | 2.44748 | 689.1665 |
| C0J56_RS17355 | C0J56_RS17355 | upstream | resistance-nodulation-cell division (RND) multidrug efflux membrane fusion protein MexC | 3.38456 | 181.24098 |
| C0J56_RS17370 | C0J56_RS17370 | inside | ureidoglycolate lyase | 1.37328 | 127.79281 |
| C0J56_RS17440 | phlD | upstream | type III polyketide synthase | 3.05044 | 316.96454 |
| C0J56_RS17455 | C0J56_RS17455 | inside | toxin | 2.31009 | 11.23065 |
| C0J56_RS17470 | C0J56_RS17470 | upstream | Uncharacterized protein | 2.58995 | 231.93896 |
| C0J56_RS17620 | C0J56_RS17620 | upstream | Anthranilate synthase, aminase component | 4.47771 | 107.4052 |
| C0J56_RS17670 | C0J56_RS17670 | upstream | LysR family transcriptional regulator | 3.5491 | 150.32112 |
| C0J56_RS17800 | C0J56_RS17800 | upstream | ABC transporter ATP-binding protein | 8.29394 | 554.00446 |
| C0J56_RS17845 | C0J56_RS17845 | inside | multidrug efflux RND transporter permease subunit | 9.72885 | 333.69263 |
| C0J56_RS17855 | C0J56_RS17855 | upstream | socitrate lyase/phosphoenolpyruvate mutase family protein | 11.44361 | 1753.33057 |
| C0J56_RS17895 | C0J56_RS17895 | upstream | Polyketide cyclase | 6.98879 | 2292.11084 |
| C0J56_RS17915 | C0J56_RS17915 | upstream | TetR/AcrR family transcriptional regulator | 9.06482 | 2983.10303 |
| C0J56_RS17975 | C0J56_RS17975 | upstream | TetR family transcriptional regulator | 1.69695 | 1300.04089 |
| C0J56_RS18070 | mupI | upstream | acyl-homoserine-lactone synthase | 3.9682 | 2037.93909 |
| C0J56_RS18085 | mupR | upstream | LuxR family transcriptional regulator | 11.15507 | 34.74095 |
| C0J56_RS18090 | mupW | inside | aromatic ring-hydroxylating dioxygenase subunit alpha | 1.56284 | 622.70972 |
| C0J56_RS18095 | mupV | inside | SDR family oxidoreductase | 13.04701 | 31884.85742 |
| C0J56_RS18160 | mupL | inside | alpha/beta hydrolase | 15.42752 | 34.08069 |
| C0J56_RS18160 | mupL | inside | alpha/beta hydrolase | 1.36609 | 3864.81885 |
| C0J56_RS18235 | mmpD | inside | SDR family NAD(P)-dependent oxidoreductase | 1.43745 | 10.80485 |
| C0J56_RS18235 | mmpD | inside | SDR family NAD(P)-dependent oxidoreductase | 1.42473 | 5412.29932 |
| C0J56_RS18235 | mmpD | inside | SDR family NAD(P)-dependent oxidoreductase | 1.23524 | 14.69464 |
| C0J56_RS18250 | mmpB | upstream | KR domain-containing protein | 6.45198 | 13.90454 |
| C0J56_RS18930 | infA | upstream | translation initiation factor IF-1 | 2.6332 | 4.62908 |
| C0J56_RS18945 | C0J56_RS18945 | upstream | cold shock domain protein CspD | 1.57229 | 1125.27698 |
| C0J56_RS19015 | C0J56_RS19015 | upstream | NADH-quinone oxidoreductase subunit A | 3.1246 | 159.69588 |
| C0J56_RS19280 | C0J56_RS19280 | upstream | AsnC family transcriptional regulator | 4.38103 | 24.20335 |
| C0J56_RS19295 | C0J56_RS19295 | upstream | tRNA-Pro | 4.38612 | 250.40205 |
| C0J56_RS19330 | infC | inside | translation initiation factor IF-3 | 6.23104 | 529.40582 |
| C0J56_RS19345 | C0J56_RS19345 | upstream | cold-shock protein | 1.87684 | 530.69135 |
| C0J56_RS19720 | C0J56_RS19720 | upstream | branched-chain amino acid ABC transporter permease | 12.34169 | 1062.76965 |
| C0J56_RS19805 | C0J56_RS19805 | upstream | Uncharacterized protein | 6.71148 | 53.46167 |
| C0J56_RS19820 | C0J56_RS19820 | upstream | tRNA-Asp | 6.4591 | 125.24159 |
| C0J56_RS19850 | C0J56_RS19850 | upstream | colicin V production protein CvpA | 1.86132 | 3362.9917 |
| C0J56_RS19955 | C0J56_RS19955 | upstream | TetR family transcriptional regulator | 11.81749 | 1208.84399 |
| C0J56_RS20000 | C0J56_RS20000 | upstream | tRNA-Asn | 2.15525 | 1263.65942 |
| C0J56_RS20015 | C0J56_RS20015 | upstream | AI-2E family transporter | 4.14515 | 62.88855 |
| C0J56_RS20315 | C0J56_RS20315 | inside | Uncharacterized protein | 4.68633 | 3502.30396 |
| C0J56_RS20335 | C0J56_RS20335 | upstream | 23S ribosomal RNA | 36.11681 | 96.67271 |
| C0J56_RS20580 | C0J56_RS20580 | upstream | Uncharacterized protein | 2.48564 | 573.37817 |
| C0J56_RS20620 | C0J56_RS20620 | upstream | Uncharacterized protein | 2.41949 | 608.28766 |
| C0J56_RS20620 | C0J56_RS20620 | inside | Uncharacterized protein | 1.44508 | 16132.82422 |
| C0J56_RS20625 | C0J56_RS20625 | upstream | Uncharacterized protein | 4.21566 | 133.69186 |
| C0J56_RS20685 | C0J56_RS20685 | inside | ketosteroid isomerase | 3.44623 | 15.17755 |
| C0J56_RS20945 | acpP | upstream | acyl carrier protein | 2.5054 | 123.53122 |
| C0J56_RS20955 | fabD | upstream | Malonyl CoA-acyl carrier protein transacylase | 1.38965 | 488.17532 |
| C0J56_RS20970 | C0J56_RS20970 | upstream | Large ribosomal RNA subunit accumulation protein YceD | 4.49677 | 322.98788 |
| C0J56_RS20990 | rluC | upstream | 23S rRNA pseudouridine synthase RluC | 2.89124 | 161.65514 |
| C0J56_RS21195 | C0J56_RS21195 | upstream | alcohol dehydrogenase | 10.84319 | 13.76497 |
| C0J56_RS21225 | C0J56_RS21225 | upstream | tRNA-Gly | 10.27075 | 653.91772 |
| C0J56_RS21425 | sucA | upstream | 2-oxoglutarate dehydrogenase subunit E1 | 3.75953 | 238.85339 |
| C0J56_RS21450 | gltA | upstream | citrate synthase | 2.41644 | 2735.79346 |
| C0J56_RS21710 | minD | upstream | septum site-determining protein MinD | 1.65647 | 2505.2998 |
| C0J56_RS21785 | C0J56_RS21785 | upstream | Uncharacterized protein | 3.99402 | 462.69656 |
| C0J56_RS21815 | C0J56_RS21815 | upstream | Putative autotransporter | 14.17603 | 154.4904 |
| C0J56_RS21890 | C0J56_RS21890 | upstream | tRNA-Asp | 7.70032 | 37.9395 |
| C0J56_RS21960 | C0J56_RS21960 | inside | Uncharacterized protein | 4.09675 | 489.31192 |
| C0J56_RS21960 | C0J56_RS21960 | inside | Uncharacterized protein | 3.9135 | 4172.82031 |
| C0J56_RS21995 | C0J56_RS21995 | upstream | Uncharacterized protein | 3.1197 | 652.76923 |
| C0J56_RS22085 | C0J56_RS22085 | upstream | peptidase P60 | 2.40793 | 1858.93127 |
| C0J56_RS22160 | C0J56_RS22160 | upstream | Uncharacterized protein | 8.27137 | 462.5192 |
| C0J56_RS22360 | flgM | upstream | flagellar biosynthesis protein FlgM | 1.28446 | 506.0209 |
| C0J56_RS22395 | C0J56_RS22395 | inside | Uncharacterized protein | 2.06331 | 286.64539 |
| C0J56_RS22405 | C0J56_RS22405 | inside | Uncharacterized protein | 12.77929 | 149.58327 |
| C0J56_RS22455 | C0J56_RS22455 | upstream | DNA-binding protein | 2.31445 | 1891.35876 |
| C0J56_RS22470 | mgtE | upstream | magnesium transporter MgtE | 1.47262 | 7.58901 |
| C0J56_RS22480 | C0J56_RS22480 | upstream | tRNA-Arg | 10.06032 | 74.33904 |
| C0J56_RS22505 | alaS | upstream | alanine--tRNA ligase | 1.44487 | 3552.18555 |
| C0J56_RS22555 | C0J56_RS22555 | inside | acetylornithine aminotransferase 1 | 1.57849 | 116.43514 |
| C0J56_RS22580 | C0J56_RS22580 | upstream | ABC transporter substrate-binding protein（argT6） | 2.75928 | 17.90733 |
| C0J56_RS22600 | C0J56_RS22600 | upstream | ribonucleoside-diphosphate reductase subunit beta/nrdB | 12.67243 | 2594.76978 |
| C0J56_RS22615 | C0J56_RS22615 | upstream | Type II toxin-antitoxin system RelE/ParE family toxin | 1.62571 | 17.74549 |
| C0J56_RS22695 | C0J56_RS22695 | upstream | PepSY domain-containing protein | 2.02202 | 32.9905 |
| C0J56_RS22725 | C0J56_RS22725 | upstream | aromatic amino acid transporter AroP | 2.20901 | 204.96895 |
| C0J56_RS22850 | C0J56_RS22850 | upstream | 10 kDa chaperonin | 1.47464 | 3505.74414 |
| C0J56_RS22910 | C0J56_RS22910 | upstream | Uncharacterized protein | 6.77409 | 28.52114 |
| C0J56_RS22950 | C0J56_RS22950 | upstream | (2Fe-2S)-binding protein | 2.26716 | 82.60564 |
| C0J56_RS22995 | glpF | upstream | aquaporin | 1.82642 | 109.46468 |
| C0J56_RS23005 | C0J56_RS23005 | upstream | DeoR family transcriptional regulator | 1.71397 | 20.03277 |
| C0J56_RS23015 | glpD | upstream | glycerol-3-phosphate dehydrogenase | 3.36041 | 1372.23926 |
| C0J56_RS23100 | C0J56_RS23100 | upstream | cold-shock protein | 4.1858 | 115.9715 |
| C0J56_RS23145 | C0J56_RS23145 | upstream | tRNA-Lys | 3.12295 | 57.05687 |
| C0J56_RS23165 | pal | upstream | peptidoglycan-associated lipoprotein | 2.18894 | 45.1045 |
| C0J56_RS23220 | C0J56_RS23220 | upstream | Uncharacterized protein | 1.91449 | 430.29684 |
| C0J56_RS23250 | C0J56_RS23250 | upstream | porin | 2.94673 | 569.3031 |
| C0J56_RS23295 | secA | upstream | protein translocase subunit SecA | 1.6888 | 292.86917 |
| C0J56_RS23335 | C0J56_RS23335 | upstream | transcriptional regulator | 6.10989 | 120.40922 |
| C0J56_RS23515 | metE | upstream | 5-methyltetrahydropteroyltriglutamate--homocystei ne methyltransferase | 2.01435 | 64.37996 |
| C0J56_RS23595 | C0J56_RS23595 | inside | Uncharacterized protein | 8.56362 | 229.01566 |
| C0J56_RS23660 | C0J56_RS23660 | upstream | Uncharacterized protein | 5.23163 | 40.59221 |
| C0J56_RS23705 | C0J56_RS23705 | upstream | Uncharacterized protein | 4.80566 | 1160.521 |
| C0J56_RS23870 | lepB | inside | signal peptidase I | 1.62885 | 86.85853 |
| C0J56_RS23875 | lepA | upstream | translation elongation factor 4 | 2.20265 | 1851.56042 |
| C0J56_RS23910 | rlmF | upstream | ribosomal RNA large subunit methyltransferase F | 1.81978 | 778.31085 |
| C0J56_RS23990 | rhlB | inside | ATP-dependent RNA helicase RhlB | 8.54581 | 695.14667 |
| C0J56_RS23995 | C0J56_RS23995 | inside | Rhs family protein / insecticidal toxin protein | 8.52037 | 33.43334 |
| C0J56_RS24000 | C0J56_RS24000 | upstream | Rhs family protein / insecticidal toxin protein | 12.38494 | 111.0937 |
| C0J56_RS24005 | C0J56_RS24005 | upstream | Rhs family protein / insecticidal toxin protein | 9.21238 | 51.0807 |
| C0J56_RS24015 | C0J56_RS24015 | inside | Uncharacterized protein | 13.16854 | 1845.02869 |
| C0J56_RS24095 | algD | upstream | GDP-mannose 6-dehydrogenase | 7.79966 | 1835.70728 |
| C0J56_RS24245 | lpxC | upstream | UDP-3-O-acyl-N-acetylglucosamine deacetylase | 1.86715 | 3381.57227 |
| C0J56_RS24305 | C0J56_RS24305 | inside | peptidoglycan glycosyltransferase | 2.93997 | 2093.72437 |
| C0J56_RS24360 | sspA | upstream | stringent starvation protein A/sspA | 9.06898 | 3722.64624 |
| C0J56_RS24480 | cyoA | upstream | ubiquinol oxidase subunit 2 | 2.09362 | 1692.87109 |
| C0J56_RS24620 | C0J56_RS24620 | upstream | transcriptional regulator | 1.80411 | 64.01805 |
| C0J56_RS24625 | C0J56_RS24625 | upstream | Uncharacterized protein | 7.06686 | 248.91458 |
| C0J56_RS24645 | C0J56_RS24645 | upstream | Colicin/Pyocin-S2 | 10.95513 | 2228.74194 |
| C0J56_RS24650 | C0J56_RS24650 | inside | pyocin-S2 immunity protein | 12.83017 | 83.4213 |
| C0J56_RS24865 | C0J56_RS24865 | upstream | Uncharacterized protein | 6.08461 | 50.44401 |
| C0J56_RS24875 | C0J56_RS24875 | upstream | Uncharacterized protein | 4.15572 | 1487.20935 |
| C0J56_RS24935 | C0J56_RS24935 | upstream | short-chain dehydrogenase | 4.72383 | 2781.48022 |
| C0J56_RS24990 | C0J56_RS24990 | upstream | tRNA-Gln | 15.66198 | 3574.35327 |
| C0J56_RS25070 | C0J56_RS25070 | upstream | tRNA-Met | 2.32081 | 1072.13062 |
| C0J56_RS25170 | C0J56_RS25170 | upstream | paraquat-inducible protein A | 39.79821 | 510.92673 |
| C0J56_RS25230 | ilvB | upstream | acetolactate synthase | 2.43039 | 768.32031 |
| C0J56_RS25265 | C0J56_RS25265 | inside | Regulator of competence-specific genes | 1.67919 | 5169.41113 |
| C0J56_RS25310 | dksA | upstream | RNA polymerase-binding transcription factor DksA | 1.82704 | 118.64397 |
| C0J56_RS25335 | C0J56_RS25335 | upstream | poly(A) polymerase I | 4.98084 | 18418.97266 |
| C0J56_RS25420 | C0J56_RS25420 | upstream | Uncharacterized protein | 1.86339 | 128.01065 |
| C0J56_RS25450 | C0J56_RS25450 | upstream | methyl-accepting chemotaxis protein | 8.17021 | 35.42273 |
| C0J56_RS25490 | C0J56_RS25490 | inside | outer membrane protein assembly factor BamD | 2.11297 | 50.19514 |
| C0J56_RS25610 | murJ | upstream | putative lipid II flippase MurJ | 3.87052 | 724.45441 |
| C0J56_RS25650 | C0J56_RS25650 | upstream | octaprenyl diphosphate synthase | 4.42763 | 54.71912 |
| C0J56_RS25730 | C0J56_RS25730 | upstream | serine hydroxymethyltransferase 2 | 7.06845 | 1721.84106 |
| C0J56_RS25785 | katB | inside | catalase | 3.92309 | 84.11717 |
| C0J56_RS25795 | C0J56_RS25795 | upstream | ferredoxin--NADP(+) reductase | 3.43461 | 422.39328 |
| C0J56_RS25960 | asnB | upstream | asparagine synthetase B | 1.71985 | 550.41736 |
| C0J56_RS25970 | C0J56_RS25970 | upstream | Uncharacterized protein | 2.86726 | 1550.73718 |
| C0J56_RS26015 | C0J56_RS26015 | upstream | UDP-N-acetyl-2-amino-2-deoxy-D-glucuronate oxidase | 1.3255 | 1335.96802 |
| C0J56_RS26020 | wbpA | upstream | UDP-N-acetyl-D-glucosamine 6-dehydrogenase | 1.5214 | 417.97006 |
| C0J56_RS26115 | C0J56_RS26115 | upstream | Uncharacterized protein | 5.89481 | 309.2074 |
| C0J56_RS26145 | C0J56_RS26145 | upstream | TPR repeat, SEL1 subfamily | 2.05002 | 36.84922 |
| C0J56_RS26280 | C0J56_RS26280 | upstream | gamma-glutamyl phosphate reductase | 1.52063 | 197.94272 |
| C0J56_RS26390 | C0J56_RS26390 | upstream | transcriptional regulator | 3.29977 | 8.41346 |
| C0J56_RS26395 | C0J56_RS26395 | upstream | Uncharacterized protein | 5.44195 | 20.37583 |
| C0J56_RS26610 | thiL | inside | thiamine-monophosphate kinase | 1.3232 | 951.96729 |
| C0J56_RS26690 | C0J56_RS26690 | upstream | outer membrane protein W | 7.19741 | 73.61086 |
| C0J56_RS26985 | fusA | inside | elongation factor G | 6.99388 | 21.36999 |
| C0J56_RS27000 | rpoC | upstream | DNA-directed RNA polymerase subunit beta' | 1.43236 | 281.20593 |
| C0J56_RS27040 | C0J56_RS27040 | upstream | tRNA-Trp | 9.59087 | 817.6983 |
| C0J56_RS27085 | C0J56_RS27085 | upstream | 23S ribosomal RNA | 38.83397 | 8.93051 |
| C0J56_RS27120 | C0J56_RS27120 | upstream | Cell wall endopeptidase, family M23/M37 | 3.31249 | 1369.88806 |
| C0J56_RS27285 | C0J56_RS27285 | upstream | LPS-assembly protein LptD | 2.02006 | 1301.73181 |
| C0J56_RS27360 | rpsU | upstream | 30S ribosomal protein S21 | 5.40124 | 14.37631 |
| C0J56_RS27370 | rpoD | upstream | RNA polymerase sigma factor RpoD | 3.44478 | 2541.09033 |
| C0J56_RS27395 | C0J56_RS27395 | inside | tryptophan synthase subunit alpha | 1.47561 | 17814.14062 |
| C0J56_RS27440 | C0J56_RS27440 | upstream | UPF0057 membrane protein | 4.78301 | 283.80807 |
| C0J56_RS27470 | C0J56_RS27470 | inside | Uncharacterized protein | 1.70204 | 69.04587 |
| C0J56_RS27625 | C0J56_RS27625 | upstream | cold shock domain-containing protein | 2.5874 | 805.93652 |
| C0J56_RS27690 | C0J56_RS27690 | upstream | Uncharacterized protein | 2.51871 | 311.35773 |
| C0J56_RS27790 | C0J56_RS27790 | upstream | alginate lyase family protein | 9.44899 | 17.17736 |
| C0J56_RS28005 | C0J56_RS28005 | upstream | ArsR family transcriptional regulator | 1.62571 | 633.98596 |
| C0J56_RS28075 | ahcY | upstream | adenosylhomocysteinase | 1.99462 | 35.20497 |
| C0J56_RS28080 | metF | upstream | methylenetetrahydrofolate reductase | 1.68677 | 149.90665 |
| C0J56_RS28380 | rpoH | upstream | RNA polymerase sigma factor RpoH | 1.66642 | 138.88498 |
| C0J56_RS28435 | C0J56_RS28435 | inside | putative coniferyl aldehyde dehydrogenase | 1.44762 | 2184.01367 |
| C0J56_RS28455 | C0J56_RS28455 | upstream | 4Fe-4S ferredoxin | 1.69441 | 28.52114 |
| C0J56_RS28550 | C0J56_RS28550 | upstream | sigma-54-dependent Fis family transcriptional regulator | 2.08621 | 66.0053 |
| C0J56_RS28710 | C0J56_RS28710 | upstream | Auxin Efflux Carrier /AEC family transporter | 11.02636 | 33.82088 |
| C0J56_RS28940 | ssrS | upstream | 6S RNA | 1.75243 | 32.01207 |
| C0J56_RS29000 | rho | upstream | transcription termination factor Rho | 4.24194 | 15.34012 |
| C0J56_RS29125 | rsd | upstream | sigma D regulator | 1.41964 | 34.50999 |
| C0J56_RS29315 | C0J56_RS29315 | upstream | cytochrome C5 | 1.64655 | 77.20374 |
| C0J56_RS29335 | dadA | inside | D-amino acid dehydrogenase | 1.46616 | 2810.65405 |
| C0J56_RS29395 | rpmB | upstream | 50S ribosomal protein L28 | 5.62004 | 40.69545 |
| C0J56_RS29435 | C0J56_RS29435 | upstream | exodeoxyribonuclease III | 1.79617 | 496.33145 |
| C0J56_RS29440 | gltS | upstream | sodium/glutamate symporter | 4.19022 | 13.59397 |
| C0J56_RS29465 | rpoZ | upstream | DNA-directed RNA polymerase subunit omega | 3.8134 | 31.66469 |
| C0J56_RS29520 | C0J56_RS29520 | inside | Uncharacterized protein | 2.86726 | 16.68068 |
| C0J56_RS29540 | C0J56_RS29540 | upstream | Uncharacterized protein | 4.94075 | 869.76105 |
| C0J56_RS29610 | tagH | upstream | type VI secretion system-associated FHA domain protein TagH | 3.12677 | 44.24679 |
| C0J56_RS29710 | C0J56_RS29710 | upstream | Uncharacterized protein | 1.55448 | 481.9296 |
| C0J56_RS30010 | C0J56_RS30010 | inside | ATP synthase epsilon chain | 3.48549 | 404.1048 |
| C0J56_RS30095 | rnpA | upstream | ribonuclease P protein component | 5.78795 | 197.94272 |
| C0J56_RS30115 | C0J56_RS30115 | upstream | Uncharacterized protein | 1.97044 | 676.62524 |
| C0J56_RS30150 | C0J56_RS30150 | upstream | Uncharacterized protein | 8.2851 | 246.66359 |
| C0J56_RS30295 | C0J56_RS30295 | inside | Uncharacterized protein | 2.32894 | 7.4603 |
| C0J56_RS30335 | C0J56_RS30335 | upstream | Uncharacterized protein | 7.72405 | 22.83167 |
| C0J56_RS30760 | C0J56_RS30760 | inside | CdiI_2 domain-containing protein | 4.54066 | 320.01028 |
| C0J56_RS30850 | C0J56_RS30850 | inside | protein hcp1（Hcp Type VI protein secretion system component Hcp (secreted cytotoxin)） | 1.30156 | 919.73224 |

**Sample 4 vs input**

| geneID  (target genes) | Gene name | Position | Gene description | Fold_enrichment | -log10(pvalue) |
| --- | --- | --- | --- | --- | --- |
| C0J56_RS00005 | dnaA | upstream | chromosomal replication initiator protein DnaA | 1.795 | 48.25461 |
| C0J56_RS00120 | C0J56_RS00120 | upstream | ATPase | 17.31754 | 6183.28662 |
| C0J56_RS00200 | C0J56_RS00200 | upstream | Cytochrome c5 | 2.89943 | 222.92249 |
| C0J56_RS00425 | C0J56_RS00425 | upstream | AAA family ATPase | 15.7318 | 5352.0166 |
| C0J56_RS00455 | C0J56_RS00455 | upstream | DUF4065 domain-containing protein | 5.65006 | 961.34039 |
| C0J56_RS00490 | C0J56_RS00490 | upstream | Uncharacterized protein | 1.82987 | 52.15134 |
| C0J56_RS00575 | C0J56_RS00575 | inside | membrane protein | 1.40924 | 14.56274 |
| C0J56_RS00595 | C0J56_RS00595 | upstream | Uncharacterized protein | 12.36502 | 3690.53394 |
| C0J56_RS00625 | C0J56_RS00625 | upstream | LysR family transcriptional regulator | 2.81573 | 206.46692 |
| C0J56_RS00775 | aspA | inside | aspartate ammonia-lyase | 2.13214 | 90.87312 |
| C0J56_RS00985 | C0J56_RS00985 | upstream | Uncharacterized protein | 14.62504 | 4789.40479 |
| C0J56_RS00995 | C0J56_RS00995 | upstream | Succinate CoA transferase | 1.72757 | 41.08219 |
| C0J56_RS01035 | C0J56_RS01035 | upstream | 16S ribosomal RNA | 32.58199 | 15314.68652 |
| C0J56_RS01275 | C0J56_RS01275 | upstream | Heme oxygenase-like protein | 7.19626 | 1498.4176 |
| C0J56_RS01555 | C0J56_RS01555 | upstream | FAD-binding oxidoreductase | 1.94613 | 66.02203 |
| C0J56_RS01620 | C0J56_RS01620 | upstream | Uncharacterized protein | 6.58941 | 1279.39221 |
| C0J56_RS01760 | C0J56_RS01760 | inside | Uncharacterized protein | 1.57876 | 27.01378 |
| C0J56_RS01930 | C0J56_RS01930 | upstream | tRNA-Thr | 2.55531 | 158.30772 |
| C0J56_RS02010 | C0J56_RS02010 | upstream | Uncharacterized protein | 2.40185 | 132.2182 |
| C0J56_RS02155 | thiI | upstream | tRNA sulfurtransferase | 2.19492 | 99.95568 |
| C0J56_RS02395 | PhaI | upstream | Uncharacterized protein | 2.27862 | 112.58512 |
| C0J56_RS02460 | C0J56_RS02460 | upstream | Uncharacterized protein | 2.2856 | 113.66385 |
| C0J56_RS02470 | C0J56_RS02470 | inside | malate dehydrogenase | 1.41368 | 14.46179 |
| C0J56_RS02525 | gltB | upstream | glutamate synthase large subunit | 1.43925 | 16.18307 |
| C0J56_RS02540 | gbpA | upstream | GlcNAc-binding protein A | 4.89671 | 727.18304 |
| C0J56_RS02615 | C0J56_RS02615 | upstream | Uncharacterized protein | 8.83548 | 2135.81226 |
| C0J56_RS02625 | C0J56_RS02625 | upstream | Uncharacterized protein | 11.72793 | 3394.14233 |
| C0J56_RS02845 | C0J56_RS02845 | upstream | Uncharacterized protein | 6.81029 | 1357.95251 |
| C0J56_RS02890 | aceE | upstream | pyruvate dehydrogenase E1 component | 1.55086 | 24.65865 |
| C0J56_RS03210 | hflX | inside | GTPase HflX | 2.30019 | 119.15977 |
| C0J56_RS03225 | C0J56_RS03225 | upstream | ATP phosphoribosyltransferase regulatory subunit | 1.6358 | 32.9898 |
| C0J56_RS03245 | C0J56_RS03245 | upstream | tRNA-Leu | 5.56868 | 935.0946 |
| C0J56_RS03280 | C0J56_RS03280 | inside | Uncharacterized protein | 2.58152 | 174.5713 |
| C0J56_RS03340 | azu | upstream | Azurin | 2.01093 | 75.43762 |
| C0J56_RS03640 | C0J56_RS03640 | upstream | Toxin A | 7.8994 | 1839.56763 |
| C0J56_RS03645 | C0J56_RS03645 | upstream | UPF0056 inner membrane protein | 8.57041 | 2028.59033 |
| C0J56_RS03685 | dusB | upstream | tRNA-dihydrouridine synthase B | 1.4097 | 14.77323 |
| C0J56_RS03745 | C0J56_RS03745 | upstream | translation initiation factor | 1.47078 | 19.46875 |
| C0J56_RS03980 | C0J56_RS03980 | upstream | 16S ribosomal RNA | 34.25608 | 64.83891 |
| C0J56_RS04190 | C0J56_RS04190 | upstream | proline iminopeptidase | 1.73373 | 16412.42773 |
| C0J56_RS04220 | C0J56_RS04220 | inside | addiction module antidote protein, HigA family | 1.32067 | 43.39662 |
| C0J56_RS04375 | ssrA | upstream | transfer-messenger RNA | 4.56422 | 8.9305 |
| C0J56_RS04415 | smpB | inside | SsrA-binding protein | 1.9032 | 630.53815 |
| C0J56_RS04435 | C0J56_RS04435 | upstream | outer membrane protein assembly factor BamE | 2.58007 | 68.20604 |
| C0J56_RS04455 | dnaK | inside | chaperone protein DnaK | 1.55844 | 170.6022 |
| C0J56_RS04470 | carA | upstream | carbamoyl-phosphate synthase small chain | 4.11239 | 25.91334 |
| C0J56_RS04500 | ftsH | upstream | ATP-dependent zinc metalloprotease FtsH | 1.39792 | 540.59448 |
| C0J56_RS04525 | C0J56_RS04525 | upstream | tRNA-Leu | 6.66999 | 15.99892 |
| C0J56_RS04785 | C0J56_RS04785 | upstream | Uncharacterized protein | 5.29076 | 1389.02319 |
| C0J56_RS04850 | gatC | upstream | Glutamyl-tRNA(Gln) amidotransferase subunit C | 2.41437 | 910.84753 |
| C0J56_RS04945 | raiA | upstream | ribosomal subunit interface protein | 1.61102 | 153.62947 |
| C0J56_RS04950 | RpoN | upstream | RNA polymerase sigma-54 factor | 1.71373 | 33.64304 |
| C0J56_RS05005 | C0J56_RS05005 | upstream | BolA family transcriptional regulator | 1.38101 | 44.51054 |
| C0J56_RS05175 | C0J56_RS05175 | upstream | Reverse transcriptase domain-containing protein | 19.11486 | 15.04497 |
| C0J56_RS05215 | C0J56_RS05215 | upstream | tRNA-Leu | 2.6617 | 7158.20117 |
| C0J56_RS05265 | iscR | upstream | Fe-S cluster assembly transcriptional regulator IscR | 1.76848 | 181.14844 |
| C0J56_RS05395 | guaB | upstream | inosine-5'-monophosphate dehydrogenase | 2.24525 | 52.54428 |
| C0J56_RS05400 | guaA | upstream | GMP synthase [glutamine-hydrolyzing] | 1.2755 | 126.19351 |
| C0J56_RS05495 | ccsA | upstream | cytochrome c assembly protein | 1.50228 | 8.89264 |
| C0J56_RS05510 | rimM | inside | ribosome maturation factor RimM | 4.12452 | 26.26639 |
| C0J56_RS05885 | rpsB | upstream | 30S ribosomal protein S2 | 4.81456 | 587.1059 |
| C0J56_RS05925 | bamA | upstream | outer membrane protein assembly factor BamA | 2.19878 | 808.62213 |
| C0J56_RS05930 | C0J56_RS05930 | inside | Outer membrane chaperone skp | 1.6773 | 114.80485 |
| C0J56_RS05975 | C0J56_RS05975 | upstream | CTP synthase | 1.99195 | 41.12207 |
| C0J56_RS06000 | C0J56_RS06000 | upstream | LysR family transcriptional regulator | 2.06705 | 85.67321 |
| C0J56_RS06040 | rpoS | upstream | RNA polymerase sigma factor RpoS | 1.54658 | 99.06904 |
| C0J56_RS06050 | C0J56_RS06050 | inside | Ferredoxin 1 | 1.44249 | 28.15854 |
| C0J56_RS06300 | C0J56_RS06300 | upstream | LysR family transcriptional regulator | 3.09315 | 18.30261 |
| C0J56_RS06365 | C0J56_RS06365 | upstream | tRNA-Met | 2.16811 | 325.74887 |
| C0J56_RS06405 | C0J56_RS06405 | upstream | cold shock protein CapB | 3.10164 | 112.54382 |
| C0J56_RS06455 | C0J56_RS06455 | upstream | acyltransferase | 7.61584 | 305.76944 |
| C0J56_RS06685 | arcA | inside | arginine deiminase | 5.59792 | 1931.18176 |
| C0J56_RS06920 | alaC | upstream | tRNA-Gly | 1.82424 | 1118.79895 |
| C0J56_RS07020 | C0J56_RS07020 | upstream | Uncharacterized protein | 2.02182 | 67.7271 |
| C0J56_RS07125 | C0J56_RS07125 | upstream | secretion protein HlyD | 7.64343 | 1683.47839 |
| C0J56_RS07295 | C0J56_RS07295 | upstream | Uncharacterized protein | 4.66532 | 89.00631 |
| C0J56_RS07315 | C0J56_RS07315 | upstream | tRNA-Gly | 2.1641 | 1911.11853 |
| C0J56_RS07460 | C0J56_RS07460 | inside | sigma factor AlgU negative regulatory protein | 2.45199 | 684.86462 |
| C0J56_RS07520 | C0J56_RS07520 | upstream | tRNA-Ser | 2.5845 | 107.79512 |
| C0J56_RS07595 | C0J56_RS07595 | upstream | phosphoethanolamine transferase | 2.80418 | 195.16104 |
| C0J56_RS07705 | C0J56_RS07705 | upstream | MFS transporter | 1.82397 | 192.33479 |
| C0J56_RS07710 | C0J56_RS07710 | upstream | 5-oxoprolinase subunit A | 1.29072 | 243.30742 |
| C0J56_RS07715 | pxpB | inside | 5-oxoprolinase subunit PxpB | 1.30939 | 63.86386 |
| C0J56_RS07720 | C0J56_RS07720 | inside | Biotin-dependent carboxyltransferase | 1.34561 | 9.20075 |
| C0J56_RS07760 | C0J56_RS07760 | upstream | DMT family transporter | 1.81345 | 11.62458 |
| C0J56_RS07875 | C0J56_RS07875 | upstream | Uncharacterized protein | 14.62969 | 13.62887 |
| C0J56_RS07875 | C0J56_RS07875 | inside | Uncharacterized protein | 6.6724 | 60.13858 |
| C0J56_RS07960 | C0J56_RS07960 | upstream | Uncharacterized protein | 4.86081 | 1395.34485 |
| C0J56_RS08045 | C0J56_RS08045 | inside | Uncharacterized protein | 7.31702 | 4791.73535 |
| C0J56_RS08145 | phhA | inside | phenylalanine-4-hydroxylase | 1.46635 | 743.00708 |
| C0J56_RS08220 | ntrC1 | upstream | sigma-54-dependent Fis family transcriptional regulator | 4.83057 | 1705.67163 |
| C0J56_RS08350 | fliA | inside | RNA polymerase sigma factor FliA | 1.46315 | 21.90234 |
| C0J56_RS08470 | C0J56_RS08470 | upstream | Uncharacterized protein | 3.00826 | 786.89893 |
| C0J56_RS08605 | gyrA | upstream | DNA gyrase subunit A | 1.64173 | 22.76098 |
| C0J56_RS08640 | C0J56_RS08640 | upstream | lipoprotein | 16.33866 | 297.03732 |
| C0J56_RS08800 | C0J56_RS08800 | upstream | methyl-accepting chemotaxis protein | 1.93119 | 39.92462 |
| C0J56_RS08820 | C0J56_RS08820 | upstream | Class I SAM-dependent methyltransferase | 2.33261 | 5666.76465 |
| C0J56_RS08860 | C0J56_RS08860 | inside | Acetyltransferase-like protein | 3.40136 | 75.68061 |
| C0J56_RS08905 | C0J56_RS08905 | inside | Efp Translation elongation factor P | 1.41243 | 144.73627 |
| C0J56_RS08960 | C0J56_RS08960 | inside | ATP-dependent RNA helicase DeaD | 1.78537 | 379.73581 |
| C0J56_RS08980 | htpX | inside | Protease HtpX | 1.20825 | 16.51859 |
| C0J56_RS09030 | C0J56_RS09030 | inside | hybrid sensor histidine kinase/response regulator | 5.17346 | 57.24842 |
| C0J56_RS09120 | C0J56_RS09120 | upstream | Sensor histidine kinase | 2.29808 | 5.29375 |
| C0J56_RS09295 | C0J56_RS09295 | upstream | porin | 3.10285 | 915.87677 |
| C0J56_RS09420 | C0J56_RS09420 | upstream | Uncharacterized protein | 4.36752 | 133.91339 |
| C0J56_RS09510 | C0J56_RS09510 | upstream | membrane protein | 1.46169 | 305.15692 |
| C0J56_RS09550 | zipA | upstream | cell division protein ZipA | 1.8994 | 613.83331 |
| C0J56_RS09575 | C0J56_RS09575 | upstream | Uncharacterized protein | 4.0022 | 20.81119 |
| C0J56_RS09630 | C0J56_RS09630 | upstream | adenine phosphoribosyltransferase | 2.06057 | 67.45959 |
| C0J56_RS09645 | C0J56_RS09645 | inside | Sulfite exporter TauE/SafE family protein | 2.46921 | 511.32373 |
| C0J56_RS09670 | C0J56_RS09670 | inside | Type II toxin-antitoxin system VapC family toxin | 2.22886 | 97.40338 |
| C0J56_RS09720 | C0J56_RS09720 | upstream | Alpha/beta hydrolase | 2.08603 | 182.2476 |
| C0J56_RS09825 | C0J56_RS09825 | inside | Uncharacterized protein | 1.5845 | 127.55956 |
| C0J56_RS09945 | C0J56_RS09945 | inside | fused response regulator/phosphatase | 1.46712 | 110.34836 |
| C0J56_RS09950 | C0J56_RS09950 | inside | anti-anti-sigma factor | 1.32959 | 31.63598 |
| C0J56_RS09970 | C0J56_RS09970 | upstream | Uncharacterized protein | 11.61633 | 21.81661 |
| C0J56_RS10025 | C0J56_RS10025 | inside | HNH endonuclease | 18.1104 | 9.65798 |
| C0J56_RS10210 | C0J56_RS10210 | inside | Abasic site processing protein | 5.92907 | 3342.87744 |
| C0J56_RS10225 | C0J56_RS10225 | upstream | Uncharacterized protein | 15.51091 | 6609.26758 |
| C0J56_RS10440 | C0J56_RS10440 | upstream | tRNA-Arg | 13.06721 | 1052.96997 |
| C0J56_RS10480 | C0J56_RS10480 | upstream | DNA-binding protein HU-beta | 4.26893 | 5238.53223 |
| C0J56_RS10600 | C0J56_RS10600 | upstream | Zinc carboxypeptidase domain protein | 1.80649 | 4024.29785 |
| C0J56_RS10640 | C0J56_RS10640 | upstream | Uncharacterized protein | 2.41172 | 548.49738 |
| C0J56_RS10665 | C0J56_RS10665 | upstream | oxaloacetate decarboxylase | 9.91768 | 57.2281 |
| C0J56_RS10935 | C0J56_RS10935 | upstream | Uncharacterized protein | 4.09569 | 145.22023 |
| C0J56_RS11055 | C0J56_RS11055 | upstream | General stress protein | 3.34735 | 2732.48193 |
| C0J56_RS11240 | C0J56_RS11240 | upstream | Methionyl-tRNA formyltransferase | 2.26507 | 618.1778 |
| C0J56_RS11305 | C0J56_RS11305 | inside | cobalamin biosynthesis protein CbiG | 1.86781 | 329.21301 |
| C0J56_RS11475 | C0J56_RS11475 | upstream | carbon-nitrogen hydrolase family protein | 1.88531 | 123.99699 |
| C0J56_RS11485 | gacA | upstream | response regulator GacA | 1.50978 | 64.80363 |
| C0J56_RS11500 | C0J56_RS11500 | upstream | tRNA-Gly | 2.46231 | 61.68946 |
| C0J56_RS11590 | C0J56_RS11590 | inside | LysR family transcriptional regulator | 1.69502 | 21.86898 |
| C0J56_RS11915 | C0J56_RS11915 | inside | LysR family transcriptional regulator | 1.86708 | 142.28394 |
| C0J56_RS12005 | C0J56_RS12005 | upstream | ABC transporter permease | 7.1138 | 37.7931 |
| C0J56_RS12215 | C0J56_RS12215 | upstream | transcriptional regulator | 4.08757 | 56.44442 |
| C0J56_RS12235 | hppD | upstream | 4-hydroxyphenylpyruvate dioxygenase | 1.71362 | 1497.50769 |
| C0J56_RS12370 | C0J56_RS12370 | upstream | 2-oxoisovalerate dehydrogenase subunit alpha | 3.37927 | 499.98459 |
| C0J56_RS12450 | hydA | upstream | D-hydantoinase/dihydropyrimidinase | 3.62255 | 39.65849 |
| C0J56_RS12570 | C0J56_RS12570 | upstream | methyl-accepting chemotaxis protein | 1.67899 | 329.30069 |
| C0J56_RS12645 | C0J56_RS12645 | upstream | Uncharacterized protein | 13.40202 | 382.62918 |
| C0J56_RS12655 | C0J56_RS12655 | upstream | Rhs family protein / insecticidal toxin protein | 12.47895 | 36.75677 |
| C0J56_RS12695 | C0J56_RS12695 | inside | Uncharacterized protein | 1.32107 | 4185.9502 |
| C0J56_RS12770 | C0J56_RS12770 | upstream | Uncharacterized protein | 5.1827 | 3744.18896 |
| C0J56_RS12780 | C0J56_RS12780 | inside | EamA domain-containing protein | 8.59599 | 9.38681 |
| C0J56_RS12820 | C0J56_RS12820 | upstream | Antibiotic biosynthesis monooxygenase | 15.2412 | 813.69562 |
| C0J56_RS12855 | acnB | upstream | aconitate hydratase B | 1.4332 | 2038.87036 |
| C0J56_RS12915 | C0J56_RS12915 | upstream | Uncharacterized protein | 8.95406 | 5100.76367 |
| C0J56_RS12955 | C0J56_RS12955 | upstream | lytic transglycosylase | 1.68853 | 15.85946 |
| C0J56_RS13195 | C0J56_RS13195 | upstream | transcriptional regulator | 3.08544 | 2184.26514 |
| C0J56_RS13530 | C0J56_RS13530 | upstream | 2-hydroxymuconate tautomerase | 8.38673 | 37.54639 |
| C0J56_RS13545 | C0J56_RS13545 | upstream | Uncharacterized protein | 7.42645 | 261.08649 |
| C0J56_RS13745 | narX | upstream | histidine kinase | 3.022 | 1955.18433 |
| C0J56_RS13955 | C0J56_RS13955 | upstream | Uncharacterized protein | 1.77872 | 1584.04517 |
| C0J56_RS14025 | C0J56_RS14025 | upstream | ABC transporter permease | 5.01762 | 249.59047 |
| C0J56_RS14360 | lpxO | upstream | LPS biosynthetic protein LpxO | 1.61131 | 46.47938 |
| C0J56_RS14390 | C0J56_RS14390 | upstream | Uncharacterized protein | 1.66712 | 763.38763 |
| C0J56_RS14490 | C0J56_RS14490 | upstream | Uncharacterized protein | 3.93876 | 29.87665 |
| C0J56_RS14715 | C0J56_RS14715 | upstream | Uncharacterized protein | 6.25924 | 35.06737 |
| C0J56_RS14745 | C0J56_RS14745 | upstream | Uncharacterized protein | 2.71807 | 461.29974 |
| C0J56_RS14760 | C0J56_RS14760 | upstream | serralysin family metalloprotease | 7.01258 | 1164.55615 |
| C0J56_RS14770 | C0J56_RS14770 | upstream | ABC transporter ATP-binding protein | 6.54755 | 187.85762 |
| C0J56_RS15290 | C0J56_RS15290 | upstream | AMP-binding protein | 2.10191 | 1431.07397 |
| C0J56_RS15460 | C0J56_RS15460 | upstream | transcriptional regulator | 1.66479 | 1264.66174 |
| C0J56_RS15505 | C0J56_RS15505 | upstream | peptide ABC transporter substrate-binding protein | 1.30207 | 86.62243 |
| C0J56_RS15625 | C0J56_RS15625 | upstream | BAX inhibitor protein | 1.81592 | 34.84402 |
| C0J56_RS15720 | valS | upstream | valine--tRNA ligase | 3.58069 | 7.96536 |
| C0J56_RS15795 | C0J56_RS15795 | upstream | tRNA-Leu | 3.86203 | 50.57777 |
| C0J56_RS15955 | C0J56_RS15955 | inside | Quinoprotein dehydrogenase-associated SoxYZ-like carrier | 2.15302 | 372.59329 |
| C0J56_RS16020 | C0J56_RS16020 | upstream | Rhodanese-related sulfurtransferase | 2.17167 | 441.76019 |
| C0J56_RS16090 | C0J56_RS16090 | upstream | Uncharacterized protein | 1.87173 | 99.69098 |
| C0J56_RS16110 | C0J56_RS16110 | upstream | oxygen-independent coproporphyrinogen III oxidase | 1.49288 | 96.55238 |
| C0J56_RS16380 | C0J56_RS16380 | inside | Uncharacterized protein | 2.23212 | 56.99065 |
| C0J56_RS16760 | C0J56_RS16760 | upstream | two-component sensor | 5.32221 | 22.58977 |
| C0J56_RS16915 | C0J56_RS16915 | upstream | TetR/AcrR family transcriptional regulator | 6.58243 | 105.49628 |
| C0J56_RS16940 | C0J56_RS16940 | upstream | Polyketide synthase | 4.1178 | 856.9751 |
| C0J56_RS16965 | C0J56_RS16965 | upstream | Uncharacterized protein | 1.46948 | 1276.93372 |
| C0J56_RS17355 | C0J56_RS17355 | upstream | resistance-nodulation-cell division (RND) multidrug efflux membrane fusion protein MexC | 2.2727 | 507.9671 |
| C0J56_RS17440 | phlD | upstream | type III polyketide synthase | 4.52702 | 18.32603 |
| C0J56_RS17455 | C0J56_RS17455 | inside | toxin | 3.39701 | 122.17197 |
| C0J56_RS17470 | C0J56_RS17470 | upstream | Cable pili-associated 22 kDa adhesin protein | 3.48071 | 620.00098 |
| C0J56_RS17620 | C0J56_RS17620 | upstream | Anthranilate synthase, aminase component | 5.57798 | 329.64172 |
| C0J56_RS17670 | C0J56_RS17670 | upstream | LysR family transcriptional regulator | 4.56655 | 348.99115 |
| C0J56_RS17800 | C0J56_RS17800 | upstream | ABC transporter ATP-binding protein | 10.39796 | 938.08295 |
| C0J56_RS17830 | C0J56_RS17830 | upstream | Uncharacterized protein | 1.50901 | 631.19885 |
| C0J56_RS17845 | C0J56_RS17845 | inside | multidrug efflux RND transporter permease subunit | 12.05345 | 2796.70264 |
| C0J56_RS17855 | C0J56_RS17855 | upstream | socitrate lyase/phosphoenolpyruvate mutase family protein | 13.80892 | 21.30026 |
| C0J56_RS17895 | C0J56_RS17895 | upstream | Polyketide cyclase | 8.76572 | 3544.79834 |
| C0J56_RS17915 | C0J56_RS17915 | upstream | TetR/AcrR family transcriptional regulator | 11.30476 | 4384.4917 |
| C0J56_RS17960 | C0J56_RS17960 | upstream | Alkene reductase | 1.42995 | 2107.44995 |
| C0J56_RS17975 | C0J56_RS17975 | upstream | TetR family transcriptional regulator | 1.97403 | 3200.83179 |
| C0J56_RS18070 | mupI | upstream | acyl-homoserine-lactone synthase | 6.37218 | 15.54725 |
| C0J56_RS18085 | mupR | upstream | LuxR family transcriptional regulator | 11.96373 | 69.54604 |
| C0J56_RS18090 | mupW | inside | aromatic ring-hydroxylating dioxygenase subunit alpha | 1.53465 | 1749.5697 |
| C0J56_RS18095 | mupV | inside | SDR family oxidoreductase | 14.22536 | 39616.94922 |
| C0J56_RS18160 | mupL | inside | alpha/beta hydrolase | 1.52106 | 33.86195 |
| C0J56_RS18170 | mmpE | inside | FAD-binding protein | 19.86355 | 4836.41602 |
| C0J56_RS18235 | mmpD | inside | SDR family NAD(P)-dependent oxidoreductase | 1.30207 | 23.28018 |
| C0J56_RS18250 | mmpB | upstream | KR domain-containing protein | 5.54077 | 7573.79346 |
| C0J56_RS18260 | mmpA | inside | SDR family NAD(P)-dependent oxidoreductase | 8.63552 | 7.96536 |
| C0J56_RS18930 | infA | upstream | translation initiation factor IF-1 | 2.38817 | 4.81088 |
| C0J56_RS18945 | C0J56_RS18945 | upstream | cold shock domain protein CspD | 1.72292 | 26.81402 |
| C0J56_RS19015 | C0J56_RS19015 | upstream | NADH-quinone oxidoreductase subunit A | 3.03799 | 926.14722 |
| C0J56_RS19280 | C0J56_RS19280 | upstream | AsnC family transcriptional regulator | 6.09415 | 2054.78467 |
| C0J56_RS19295 | C0J56_RS19295 | upstream | tRNA-Pro | 4.12942 | 131.72418 |
| C0J56_RS19330 | infC | inside | translation initiation factor IF-3 | 5.64931 | 40.60511 |
| C0J56_RS19345 | C0J56_RS19345 | upstream | cold-shock protein | 2.09725 | 255.62518 |
| C0J56_RS19720 | C0J56_RS19720 | upstream | branched-chain amino acid ABC transporter permease | 15.1947 | 1108.34497 |
| C0J56_RS19805 | C0J56_RS19805 | upstream | Uncharacterized protein | 8.18909 | 511.0484 |
| C0J56_RS19820 | C0J56_RS19820 | upstream | tRNA-Asp | 5.29541 | 968.32758 |
| C0J56_RS19850 | C0J56_RS19850 | upstream | colicin V production protein CvpA | 1.61846 | 87.29803 |
| C0J56_RS19955 | C0J56_RS19955 | upstream | TetR family transcriptional regulator | 9.86435 | 273.00967 |
| C0J56_RS20000 | C0J56_RS20000 | upstream | tRNA-Asn | 1.83661 | 5077.10205 |
| C0J56_RS20015 | C0J56_RS20015 | upstream | AI-2E family transporter | 3.80486 | 21.12004 |
| C0J56_RS20315 | C0J56_RS20315 | inside | Uncharacterized protein | 4.27358 | 1877.04309 |
| C0J56_RS20335 | C0J56_RS20335 | upstream | 23S ribosomal RNA | 30.70793 | 952.11285 |
| C0J56_RS20580 | C0J56_RS20580 | upstream | Uncharacterized protein | 2.7204 | 37.62068 |
| C0J56_RS20620 | C0J56_RS20620 | upstream | Uncharacterized protein | 2.8413 | 2864.25659 |
| C0J56_RS20620 | C0J56_RS20620 | inside | Uncharacterized protein | 1.55086 | 59.42722 |
| C0J56_RS20625 | C0J56_RS20625 | upstream | Uncharacterized protein | 5.71051 | 514.44684 |
| C0J56_RS20630 | C0J56_RS20630 | upstream | phosphoesterase | 1.52993 | 549.76056 |
| C0J56_RS20685 | C0J56_RS20685 | inside | ketosteroid isomerase | 3.28519 | 14104.99512 |
| C0J56_RS20945 | acpP | upstream | acyl carrier protein | 2.44088 | 188.29332 |
| C0J56_RS20970 | C0J56_RS20970 | upstream | Large ribosomal RNA subunit accumulation protein YceD | 3.9025 | 24.65865 |
| C0J56_RS20990 | rluC | upstream | 23S rRNA pseudouridine synthase RluC | 2.68394 | 211.44635 |
| C0J56_RS21180 | C0J56_RS21180 | upstream | Flp fap pilin component | 2.18713 | 980.98016 |
| C0J56_RS21195 | C0J56_RS21195 | upstream | alcohol dehydrogenase | 14.13444 | 22.95286 |
| C0J56_RS21225 | C0J56_RS21225 | upstream | tRNA-Gly | 8.73782 | 315.14304 |
| C0J56_RS21425 | sucA | upstream | 2-oxoglutarate dehydrogenase subunit E1 | 3.08332 | 164.04556 |
| C0J56_RS21430 | sdhB | inside | succinate dehydrogenase iron-sulfur subunit | 1.54917 | 527.86395 |
| C0J56_RS21450 | gltA | upstream | citrate synthase | 2.48836 | 214.44858 |
| C0J56_RS21710 | minD | upstream | septum site-determining protein MinD | 1.60611 | 102.18283 |
| C0J56_RS21785 | C0J56_RS21785 | upstream | Uncharacterized protein | 5.42191 | 4544.94141 |
| C0J56_RS21815 | C0J56_RS21815 | upstream | Putative autotransporter | 18.23131 | 2096.13354 |
| C0J56_RS21875 | C0J56_RS21875 | inside | peptidyl-prolyl cis-trans isomerase | 1.28331 | 316.76907 |
| C0J56_RS21890 | C0J56_RS21890 | upstream | tRNA-Asp | 5.99394 | 31.08207 |
| C0J56_RS21960 | C0J56_RS21960 | inside | Uncharacterized protein | 5.73581 | 183.05327 |
| C0J56_RS21995 | C0J56_RS21995 | upstream | Uncharacterized protein | 3.02892 | 35.3495 |
| C0J56_RS22085 | C0J56_RS22085 | upstream | peptidase P60 | 2.3876 | 1000.45819 |
| C0J56_RS22160 | C0J56_RS22160 | upstream | Uncharacterized protein | 10.81647 | 6674.80029 |
| C0J56_RS22395 | C0J56_RS22395 | inside | Uncharacterized protein | 17.11525 | 2140.44165 |
| C0J56_RS22455 | C0J56_RS22455 | upstream | DNA-binding protein | 2.16946 | 8.46461 |
| C0J56_RS22480 | C0J56_RS22480 | upstream | tRNA-Arg | 8.99624 | 1295.48999 |
| C0J56_RS22580 | C0J56_RS22580 | upstream | ABC transporter substrate-binding protein（argT6） | 2.4224 | 1101.68408 |
| C0J56_RS22600 | C0J56_RS22600 | upstream | ribonucleoside-diphosphate reductase subunit beta/nrdB | 16.01779 | 291.40738 |
| C0J56_RS22615 | C0J56_RS22615 | upstream | Type II toxin-antitoxin system RelE/ParE family toxin | 1.68804 | 159.55475 |
| C0J56_RS22695 | C0J56_RS22695 | upstream | PepSY domain-containing protein | 2.78367 | 3232.92554 |
| C0J56_RS22725 | C0J56_RS22725 | upstream | aromatic amino acid transporter AroP | 2.88256 | 6075.68115 |
| C0J56_RS22850 | C0J56_RS22850 | upstream | 10 kDa chaperonin | 1.53826 | 20.05223 |
| C0J56_RS22910 | C0J56_RS22910 | upstream | Uncharacterized protein | 9.07404 | 103.79954 |
| C0J56_RS22950 | C0J56_RS22950 | upstream | (2Fe-2S)-binding protein | 2.06536 | 2362.80591 |
| C0J56_RS22995 | glpF | upstream | aquaporin | 1.65015 | 155.41435 |
| C0J56_RS23005 | C0J56_RS23005 | upstream | DeoR family transcriptional regulator | 1.81738 | 5499.80713 |
| C0J56_RS23015 | glpD | upstream | glycerol-3-phosphate dehydrogenase | 3.15639 | 37.10368 |
| C0J56_RS23100 | C0J56_RS23100 | upstream | cold-shock protein | 4.73634 | 238.85794 |
| C0J56_RS23145 | C0J56_RS23145 | upstream | tRNA-Lys | 2.67359 | 257.8512 |
| C0J56_RS23165 | pal | upstream | peptidoglycan-associated lipoprotein | 1.96849 | 27.76348 |
| C0J56_RS23220 | C0J56_RS23220 | upstream | Uncharacterized protein | 1.93895 | 2493.77148 |
| C0J56_RS23250 | C0J56_RS23250 | upstream | porin | 3.55837 | 93.40641 |
| C0J56_RS23255 | C0J56_RS23255 | inside | mechanosensitive ion channel protein MscS | 1.56451 | 40.26548 |
| C0J56_RS23295 | secA | upstream | protein translocase subunit SecA | 1.72742 | 60.27768 |
| C0J56_RS23335 | C0J56_RS23335 | upstream | transcriptional regulator | 6.79829 | 405.11005 |
| C0J56_RS23515 | metE | upstream | 5-methyltetrahydropteroyltriglutamate--homocystei ne methyltransferase | 1.52104 | 805.50488 |
| C0J56_RS23595 | C0J56_RS23595 | inside | Uncharacterized protein | 11.67446 | 214.09549 |
| C0J56_RS23660 | C0J56_RS23660 | upstream | Uncharacterized protein | 7.02748 | 92.40122 |
| C0J56_RS23690 | C0J56_RS23690 | inside | Uncharacterized protein | 1.59056 | 73.80168 |
| C0J56_RS23705 | C0J56_RS23705 | upstream | Uncharacterized protein | 7.06355 | 396.10638 |
| C0J56_RS23870 | lepB | inside | signal peptidase I | 1.47828 | 28.45458 |
| C0J56_RS23875 | lepA | upstream | translation elongation factor 4 | 2.0589 | 49.05254 |
| C0J56_RS23910 | rlmF | upstream | ribosomal RNA large subunit methyltransferase F | 3.51307 | 1544.05261 |
| C0J56_RS23990 | rhlB | inside | ATP-dependent RNA helicase RhlB | 13.32762 | 28.47821 |
| C0J56_RS23995 | C0J56_RS23995 | inside | Rhs family protein / insecticidal toxin protein | 13.76474 | 3369.55054 |
| C0J56_RS24000 | C0J56_RS24000 | upstream | Rhs family protein / insecticidal toxin protein | 16.90134 | 1479.94312 |
| C0J56_RS24005 | C0J56_RS24005 | upstream | Rhs family protein / insecticidal toxin protein | 13.49735 | 28.96508 |
| C0J56_RS24015 | C0J56_RS24015 | inside | Uncharacterized protein | 18.51265 | 1576.37732 |
| C0J56_RS24095 | algD | upstream | GDP-mannose 6-dehydrogenase | 10.18549 | 22.03965 |
| C0J56_RS24245 | lpxC | upstream | UDP-3-O-acyl-N-acetylglucosamine deacetylase | 1.81247 | 97.0285 |
| C0J56_RS24305 | C0J56_RS24305 | inside | peptidoglycan glycosyltransferase | 2.74411 | 390.90924 |
| C0J56_RS24360 | sspA | upstream | stringent starvation protein A/sspA | 8.10871 | 4149.89014 |
| C0J56_RS24480 | cyoA | upstream | ubiquinol oxidase subunit 2 | 2.19884 | 4362.82617 |
| C0J56_RS24615 | hmgA | upstream | homogentisate 1,2-dioxygenase | 1.57764 | 5962.38184 |
| C0J56_RS24625 | C0J56_RS24625 | upstream | Uncharacterized protein | 9.30741 | 4232.26074 |
| C0J56_RS24645 | C0J56_RS24645 | upstream | Colicin/Pyocin-S2 | 17.47099 | 6827.87207 |
| C0J56_RS24865 | C0J56_RS24865 | upstream | Uncharacterized protein | 8.08273 | 2905.23413 |
| C0J56_RS24875 | C0J56_RS24875 | upstream | Uncharacterized protein | 3.76148 | 62.0785 |
| C0J56_RS24935 | C0J56_RS24935 | upstream | short-chain dehydrogenase | 4.32311 | 227.16104 |
| C0J56_RS24990 | C0J56_RS24990 | upstream | tRNA-Gln | 13.48849 | 2023.27466 |
| C0J56_RS25050 | C0J56_RS25050 | upstream | chemotaxis protein | 2.26545 | 107.42427 |
| C0J56_RS25070 | C0J56_RS25070 | upstream | tRNA-Met | 2.29268 | 30.10935 |
| C0J56_RS25170 | C0J56_RS25170 | upstream | paraquat-inducible protein A | 33.69804 | 2613.99072 |
| C0J56_RS25230 | ilvB | upstream | acetolactate synthase | 2.25651 | 6265.21777 |
| C0J56_RS25310 | dksA | upstream | RNA polymerase-binding transcription factor DksA | 1.73921 | 1949.03906 |
| C0J56_RS25335 | C0J56_RS25335 | upstream | poly(A) polymerase I | 4.88287 | 449.74438 |
| C0J56_RS25420 | C0J56_RS25420 | upstream | Uncharacterized protein | 1.7769 | 696.05371 |
| C0J56_RS25450 | C0J56_RS25450 | upstream | methyl-accepting chemotaxis protein | 7.21012 | 4499.26807 |
| C0J56_RS25490 | C0J56_RS25490 | inside | outer membrane protein assembly factor BamD | 2.2142 | 120.09876 |
| C0J56_RS25620 | rpsT | upstream | 30S ribosomal protein S20 | 3.09363 | 125.04474 |
| C0J56_RS25650 | C0J56_RS25650 | upstream | octaprenyl diphosphate synthase | 3.62817 | 16044.77441 |
| C0J56_RS25730 | C0J56_RS25730 | upstream | serine hydroxymethyltransferase 2 | 9.11026 | 111.70643 |
| C0J56_RS25785 | katB | inside | catalase | 5.4687 | 44.80132 |
| C0J56_RS25795 | C0J56_RS25795 | upstream | ferredoxin--NADP(+) reductase | 5.74306 | 762.54163 |
| C0J56_RS25965 | C0J56_RS25965 | inside | Group 1 glycosyl transferase | 3.98759 | 49.44683 |
| C0J56_RS26020 | wbpA | upstream | UDP-N-acetyl-D-glucosamine 6-dehydrogenase | 1.59039 | 1515.16663 |
| C0J56_RS26115 | C0J56_RS26115 | upstream | Uncharacterized protein | 8.99126 | 107.60354 |
| C0J56_RS26145 | C0J56_RS26145 | upstream | TPR repeat, SEL1 subfamily | 1.77083 | 274.11694 |
| C0J56_RS26280 | C0J56_RS26280 | inside | gamma-glutamyl phosphate reductase | 1.27644 | 390.74527 |
| C0J56_RS26390 | C0J56_RS26390 | upstream | transcriptional regulator | 4.65723 | 3188.18799 |
| C0J56_RS26395 | C0J56_RS26395 | upstream | Uncharacterized protein | 6.95678 | 2265.04761 |
| C0J56_RS26690 | C0J56_RS26690 | upstream | outer membrane protein W | 6.33829 | 903.15393 |
| C0J56_RS26985 | fusA | inside | elongation factor G | 6.28016 | 991.60413 |
| C0J56_RS27040 | C0J56_RS27040 | upstream | tRNA-Trp | 8.21653 | 473.87961 |
| C0J56_RS27085 | C0J56_RS27085 | upstream | 23S ribosomal RNA | 32.75637 | 28.02217 |
| C0J56_RS27120 | C0J56_RS27120 | upstream | Cell wall endopeptidase, family M23/M37 | 3.02266 | 2199.52661 |
| C0J56_RS27285 | C0J56_RS27285 | upstream | LPS-assembly protein LptD | 1.89265 | 47.54935 |
| C0J56_RS27360 | rpsU | upstream | 30S ribosomal protein S21 | 4.71303 | 7.00905 |
| C0J56_RS27370 | rpoD | upstream | RNA polymerase sigma factor RpoD | 3.26215 | 657.12897 |
| C0J56_RS27440 | C0J56_RS27440 | upstream | UPF0057 membrane protein | 4.42239 | 1410.79187 |
| C0J56_RS27470 | C0J56_RS27470 | inside | Uncharacterized protein | 1.77872 | 1191.76331 |
| C0J56_RS27625 | C0J56_RS27625 | upstream | cold shock domain-containing protein | 3.6179 | 1171.74023 |
| C0J56_RS27690 | C0J56_RS27690 | upstream | Uncharacterized protein | 3.00406 | 2130.01489 |
| C0J56_RS27790 | C0J56_RS27790 | upstream | alginate lyase family protein | 11.72096 | 15428.28516 |
| C0J56_RS28005 | C0J56_RS28005 | upstream | ArsR family transcriptional regulator | 1.62991 | 247.96526 |
| C0J56_RS28075 | ahcY | upstream | adenosylhomocysteinase | 1.96938 | 59.47613 |
| C0J56_RS28080 | metF | upstream | methylenetetrahydrofolate reductase | 1.73454 | 673.24988 |
| C0J56_RS28380 | rpoH | upstream | RNA polymerase sigma factor RpoH | 1.77175 | 299.28391 |
| C0J56_RS28435 | C0J56_RS28435 | inside | putative coniferyl aldehyde dehydrogenase | 1.3416 | 590.67267 |
| C0J56_RS28550 | C0J56_RS28550 | upstream | sigma-54-dependent Fis family transcriptional regulator | 2.60182 | 46.47938 |
| C0J56_RS28710 | C0J56_RS28710 | upstream | Auxin Efflux Carrier /AEC family transporter | 15.28538 | 381.50928 |
| C0J56_RS28940 | ssrS | upstream | 6S RNA | 1.6628 | 244.12396 |
| C0J56_RS29000 | rho | upstream | transcription termination factor Rho | 3.74288 | 3390.93066 |
| C0J56_RS29125 | rsd | upstream | sigma D regulator | 1.42298 | 31.56759 |
| C0J56_RS29315 | C0J56_RS29315 | upstream | cytochrome C5 | 1.61875 | 68.95367 |
| C0J56_RS29335 | dadA | inside | D-amino acid dehydrogenase | 1.79138 | 41.80161 |
| C0J56_RS29395 | rpmB | upstream | 50S ribosomal protein L28 | 4.93392 | 45.7271 |
| C0J56_RS29435 | C0J56_RS29435 | upstream | exodeoxyribonuclease III | 1.95543 | 10.07362 |
| C0J56_RS29440 | gltS | upstream | sodium/glutamate symporter | 6.67544 | 166.55817 |
| C0J56_RS29465 | rpoZ | upstream | DNA-directed RNA polymerase subunit omega | 3.75031 | 5123.26562 |
| C0J56_RS29520 | C0J56_RS29520 | inside | Uncharacterized protein | 3.87831 | 35.3172 |
| C0J56_RS29540 | C0J56_RS29540 | upstream | Uncharacterized protein | 7.73104 | 413.57541 |
| C0J56_RS29610 | tagH | upstream | type VI secretion system-associated FHA domain protein TagH | 2.91571 | 15.07778 |
| C0J56_RS29710 | C0J56_RS29710 | upstream | Uncharacterized protein | 1.51133 | 32.27335 |
| C0J56_RS30010 | C0J56_RS30010 | inside | ATP synthase epsilon chain | 3.19937 | 51.52057 |
| C0J56_RS30095 | rnpA | upstream | ribonuclease P protein component | 5.20131 | 738.26465 |
| C0J56_RS30115 | C0J56_RS30115 | upstream | Uncharacterized protein | 1.91675 | 67.18852 |
| C0J56_RS30150 | C0J56_RS30150 | upstream | Uncharacterized protein | 7.02531 | 1309.82812 |
| C0J56_RS30295 | C0J56_RS30295 | inside | Uncharacterized protein | 2.98362 | 426.10086 |
| C0J56_RS30335 | C0J56_RS30335 | upstream | Uncharacterized protein | 11.27686 | 445.88132 |
| C0J56_RS30395 | C0J56_RS30395 | inside | Uncharacterized protein | 1.50668 | 1699.39355 |
| C0J56_RS30700 | mmpD | inside | SDR family NAD(P)-dependent oxidoreductase | 1.57644 | 226.17484 |
| C0J56_RS30700 | mmpD | inside | SDR family NAD(P)-dependent oxidoreductase | 1.23205 | 21.48134 |
| C0J56_RS30760 | C0J56_RS30760 | inside | Uncharacterized protein | 8.16416 | 285.50327 |
| C0J56_RS30795 | C0J56_RS30795 | inside | Uncharacterized protein | 1.49273 | 819.42651 |

**Table S4. Binding sequence analysis of MupR regulated genes identified via ChIP-seq**

| **Gene ID** | **Gene name** | **Gene product** | **MEME motif** | **Distance to TSS^a^** |
| --- | --- | --- | --- | --- |
| C0J56_RS18270 | *mupZ* | hypothetical protein | AGTGCTGATAGGAGG | 167 bp |
| C0J56_RS18250 | *mmpB* | KR domain-containing protein | ACAGCTCATAGGTTT | -37 bp |
| C0J56_RS18200 | *mupF* | NAD-dependent epimerase/dehydratase family protein | AGGCCTTATAGGTTT | -40 bp |
| C0J56_RS18195 | *macpC* | acyl carrier protein | AGGCCTTATAGGTTT | -31 bp |
| C0J56_RS18145 | *mupO* | cytochrome P450 | AGTGCTTATAGGTTT | -32 bp |
| C0J56_RS18110 | *macpE* | hypothetical protein | GAGGCTTATAGGTTG | -32 bp |
| C0J56_RS18070 | *mupI* | acyl-homoserine-lactone synthase | AGTGTTGATAGGTTT | -13 bp |
| C0J56_RS18085 | *mupR* | LuxR family transcriptional regulator | GCCAGCGGTCCGCAG | 66 bp |
| C0J56_RS17425 | *phlA* | 2,4-diacetylphloroglucinol biosynthesis protein | GCCGGGGGCCGTGAC | 137 bp |
| C0J56_RS09865 | *gap1* | glyceraldehyde-3-phosphate dehydrogenase | TTTTTTC | 0 bp |
| C0J56_RS23015 | *glpD* | glycerol-3-phosphate dehydrogenase | TTTTTTG | -114 bp |
| C0J56_RS02145 | *glnA1* | glutamine synthetase | TTTTTTC | -125 bp |
| C0J56_RS02525 | *gltB* | glutamate synthase large subunit | TTTTTTG | -110 bp |
| C0J56_RS21445 | *sdhC* | succinate dehydrogenase, cytochrome b556 subunit | TTTTTTC | 117 bp |
| C0J56_RS00630 | *pycA* | pyruvate carboxylase subunit A/2-oxoglutarate carboxylase small subunit | TTTTGTT | 0 bp |
| C0J56_RS17420 | *phlF* | TetR/AcrR family transcriptional regulator | TTTTGTC | 141 bp |
| C0J56_RS12855 | *acnB* | aconitate hydratase B | TTTTTTC | -15 bp |
| C0J56_RS20955 | *fabD* | Malonyl CoA-acyl carrier protein transacylase | TTTTGTC | -5 bp |
| C0J56_RS00200 |  | Cytochrome c5 | TTTTGTG | 4 bp |
| C0J56_RS02890 | *aceE* | pyruvate dehydrogenase E1 component | TTTTTTC | -131 bp |
| C0J56_RS21450 | *gltA* | citrate synthase | TTTTTTC | -11 bp |
| C0J56_RS00995 | *aarC* | Succinate CoA transferase | TGTTTTG | -2 bp |
| C0J56_RS19015 | *nuoA* | NADH-quinone oxidoreductase subunit A | TTTTTTG | -509 bp |
| C0J56_RS21425 | *sucA* | 2-oxoglutarate dehydrogenase subunit E1 | TGTTTTC | 136 bp |
| C0J56_RS24480 | *cyoA* | ubiquinol oxidase subunit 2 | TTTTTTG | -445 bp |

a: TSS, transcriptional start site, predected by SAPPHIRE.CNN

**Table S5. Genes differentially expressed in the Δ*mupR* mutant compared to *P. fluorescens* 2P24** .

| LocusTag | Gene | function | log2FoldChange | *P* value |
| --- | --- | --- | --- | --- |
| C0J56_RS18150 | mupN | 4'-phosphopantetheinyl transferase superfamily protein | -1.472071382 | 0.034358218 |
| C0J56_RS18255 | mupB | hypothetical protein | -2.050840348 | 0.007223881 |
| C0J56_RS18170 | mmpE | FAD-binding protein | -2.088788166 | 0.022315809 |
| C0J56_RS18230 | mmpD | SDR family NAD(P)-dependent oxidoreductase | -2.095744883 | 0.010656038 |
| C0J56_RS18180 | mupJ | enoyl-CoA hydratase/isomerase | -2.122820443 | 0.046273581 |
| C0J56_RS18260 | mmpA | SDR family NAD(P)-dependent oxidoreductase | -2.201046354 | 0.004137906 |
| C0J56_RS18085 | mupR | LuxR family transcriptional regulator | -2.587614314 | 0.015344628 |
| C0J56_RS18185 | mupH | hydroxymethylglutaryl-CoA synthase family protein | -2.642215944 | 0.00318284 |
| C0J56_RS18250 | mmpB | KR domain-containing protein | -2.652651374 | 0.002030432 |
| C0J56_RS18135 | mupP | hypothetical protein | -2.726875941 | 0.002509938 |
| C0J56_RS18235 | mmpD | hypothetical protein | -2.7329682 | 0.000613224 |
| C0J56_RS18160 | mupL | alpha/beta hydrolase | -2.897482053 | 0.002076438 |
| C0J56_RS18265 | mupA | LLM class flavin-dependent oxidoreductase | -3.029522582 | 0.002266974 |
| C0J56_RS18210 | mupE | NADPH:quinone oxidoreductase | -3.082446881 | 0.00018227 |
| C0J56_RS18245 | mmpC | ACP S-malonyltransferase | -3.347659775 | 8.66605E-05 |
| C0J56_RS18100 | mupU | hypothetical protein | -3.379345303 | 0.000538086 |
| C0J56_RS18225 | mupC | NADH:flavin oxidoreductase/NADH oxidase family protein | -3.639248444 | 1.21818E-05 |
| C0J56_RS18190 | mupG | polyketide beta-ketoacyl:ACP synthase | -3.651807422 | 4.72325E-05 |
| C0J56_RS18165 | mmpE | FAD-dependent monooxygenase | -3.654849217 | 4.98891E-06 |
| C0J56_RS18090 | mupW | aromatic ring-hydroxylating dioxygenase subunit alpha | -3.662901308 | 0.000575629 |
| C0J56_RS18200 | mupF | NAD-dependent epimerase/dehydratase family protein | -3.749221809 | 3.93284E-07 |
| C0J56_RS18110 | macpE | hypothetical protein | -3.805002322 | 0.00028799 |
| C0J56_RS18115 | mmpF | polyketide synthase | -3.87741689 | 1.57204E-06 |
| C0J56_RS18215 | mupD | SDR family oxidoreductase | -3.952055781 | 1.88823E-06 |
| C0J56_RS18105 | mupT | Rieske (2Fe-2S) protein | -4.015055952 | 1.59484E-05 |
| C0J56_RS18175 | mupK | enoyl-CoA hydratase | -4.072072773 | 6.42788E-06 |
| C0J56_RS18095 | mupV | NAD-dependent epimerase/dehydratase family protein | -4.219653692 | 2.93526E-05 |
| C0J56_RS18130 | mupQ | long-chain fatty acid--CoA ligase | -4.245754943 | 1.03116E-05 |
| C0J56_RS18145 | mupO | cytochrome P450 | -4.342666736 | 3.71563E-05 |
| C0J56_RS18120 | macpD | hypothetical protein | -4.358705999 | 8.3339E-07 |
| C0J56_RS18195 | macpC | acyl carrier protein | -4.406974169 | 3.86421E-05 |
| C0J56_RS18125 | mupS | SDR family oxidoreductase | -4.583434737 | 1.86273E-06 |
| C0J56_RS18220 | macpA | acyl carrier protein | -4.592623459 | 1.50963E-05 |
| C0J56_RS17415 | phlG | 2,4-diacetylphloroglucinol hydrolase | -2.698777552 | 0.005532691 |
| C0J56_RS17425 | phlA | 2,4-diacetylphloroglucinol biosynthesis protein | -2.816696208 | 0.01226183 |
| C0J56_RS17445 | phlE | MFS transporter | -3.306140071 | 0.001844075 |
| C0J56_RS17430 | phlC | thiolase family protein | -3.420383619 | 0.001069757 |
| C0J56_RS17435 | phlB | 2,4-diacetylphloroglucinol biosynthesis protein | -3.796180868 | 0.000369882 |
| C0J56_RS17440 | phlD | type III polyketide synthase | -4.227057425 | 0.000118086 |
| C0J56_RS17450 | phlI | fumarylacetoacetate hydrolase | -4.411148352 | 9.85087E-06 |
| C0J56_RS15520 |  | fe2+ zn2+ uptake regulation protein | 1.919356072 | 0.028240592 |
| C0J56_RS20155 | pvdA | ornithine monooxygenase | 1.818811313 | 0.043095446 |
| C0J56_RS20260 |  | cupin | 1.692146309 | 0.03079275 |
| C0J56_RS20250 |  | thioesterase | 1.452696073 | 0.033278835 |
| C0J56_RS20220 | pvdE | cyclic peptide export ABC transporter | 1.371276837 | 0.042812429 |
| C0J56_RS25455 |  | tRNA-Pro | 3.313899821 | 0.026336754 |
| C0J56_RS28940 |  | 6S RNA | 3.014700056 | 0.007035687 |
| C0J56_RS21235 |  | tRNA-Gly | 2.68346065 | 0.018136345 |
| C0J56_RS24990 |  | tRNA-Gln | 2.580183391 | 0.023706981 |
| C0J56_RS21240 |  | tRNA-Glu | 2.541782064 | 0.02739964 |
| C0J56_RS27060 |  | tRNA-Tyr | 2.499988265 | 0.024770862 |
| C0J56_RS19970 |  | tRNA-Glu | 2.318878301 | 0.026333182 |
| C0J56_RS25460 |  | tRNA-Lys | 2.30813366 | 0.037597385 |
| C0J56_RS25470 |  | tRNA-Asn | 2.257862533 | 0.031338431 |
| C0J56_RS25465 |  | tRNA-Pro | 2.255662923 | 0.037255684 |
| C0J56_RS04520 | secG | preprotein translocase subunit SecG | 2.079927982 | 0.042175531 |
| C0J56_RS13270 | gspI | type II secretion system protein GspI | -1.981688997 | 0.034213589 |
| C0J56_RS28640 |  | YscQ/HrcQ family type III secretion apparatus protein | -2.039854559 | 0.031000558 |
| C0J56_RS02595 |  | type II secretory protein pull | -2.068554436 | 0.039333306 |
| C0J56_RS28650 |  | type III secretion protein | -2.077849386 | 0.020261022 |
| C0J56_RS13280 |  | general secretion pathway protein GspK | -2.128949061 | 0.043998869 |
| C0J56_RS09350 |  | YopN family type III secretion system gatekeeper subunit | -2.158767878 | 0.044997257 |
| C0J56_RS28590 |  | type III secretion protein | -2.432594589 | 0.037559678 |
| C0J56_RS13300 | gspE | type II secretion system protein GspE | -2.533348998 | 0.012026731 |
| C0J56_RS02580 | gspI | type II secretion system protein GspIAdd | -3.023656554 | 0.011795025 |
| C0J56_RS09365 |  | type III secretion protein | -3.055654169 | 0.014702186 |
| C0J56_RS06830 |  | FUSC family protein | -1.52768547 | 0.048271564 |
| C0J56_RS04725 |  | OprD family porin | -1.53990456 | 0.045691539 |
| C0J56_RS19450 |  | DHA2 family efflux MFS transporter permease subunit | -1.585361648 | 0.008724832 |
| C0J56_RS19460 |  | efflux transporter outer membrane subunit | -1.631814365 | 0.023392038 |
| C0J56_RS23490 |  | ABC transporter substrate-binding protein | -1.689032887 | 0.040494715 |
| C0J56_RS25110 |  | short-chain fatty acid transporter | -1.692731799 | 0.044674949 |
| C0J56_RS17020 | phnE | phosphonate ABC transporter, permease protein PhnE | -1.867294139 | 0.01930621 |
| C0J56_RS26680 |  | ABC transporter permease | -1.892298465 | 0.043600259 |
| C0J56_RS01595 |  | ATP-binding cassette domain-containing protein | -1.917719418 | 0.044835464 |
| C0J56_RS17015 | phnE | phosphonate ABC transporter, permease protein PhnE | -1.919133028 | 0.041951122 |
| C0J56_RS16695 |  | DMT family transporter | -1.923871035 | 0.041338509 |
| C0J56_RS02685 |  | sodium:proton antiporter | -1.929197988 | 0.039554455 |
| C0J56_RS13505 |  | MFS transporter | -1.981838304 | 0.038551565 |
| C0J56_RS04675 |  | ABC transporter permease | -1.990216075 | 0.03535067 |
| C0J56_RS11165 |  | nitrate ABC transporter substrate-binding protein | -2.034229088 | 0.046160887 |
| C0J56_RS18385 |  | ABC transporter ATP-binding protein | -2.053231969 | 0.033343237 |
| C0J56_RS10560 |  | ABC transporter permease | -2.094576716 | 0.049385401 |
| C0J56_RS19455 |  | HlyD family efflux transporter periplasmic adaptor subunit | -2.140553221 | 0.007911519 |
| C0J56_RS03540 | urtB | Urea ABC transporter permease subunit UrtB | -2.147975604 | 0.035221764 |
| C0J56_RS26675 |  | ABC transporter ATP-binding protein | -2.188664154 | 0.019677723 |
| C0J56_RS16010 |  | multidrug ABC transporter permease | -2.190369597 | 0.040931636 |
| C0J56_RS18370 |  | MotA/TolQ/ExbB proton channel family protein | -2.338765521 | 0.017886163 |
| C0J56_RS07990 |  | sodium:solute symport protein | -2.370128686 | 0.019936636 |
| C0J56_RS14560 |  | metal ion ABC transporter substrate-binding protein | -2.448984368 | 0.017584314 |
| C0J56_RS16465 |  | amino acid ABC transporter permease | -2.517759749 | 0.024146762 |
| C0J56_RS13450 |  | amino acid ABC transporter permease | -2.576447279 | 0.020574975 |
| C0J56_RS18815 |  | DMT family transporter | -2.763933167 | 0.014096386 |
| C0J56_RS13655 | norC | cytochrome c | 3.389733724 | 3.40854E-06 |
| C0J56_RS24475 | cyoB | cytochrome o ubiquinol oxidase subunit I | 2.133102132 | 0.031476896 |
| C0J56_RS13660 | norB | nitric-oxide reductase large subunitAdd | 2.054534262 | 0.000854831 |
| C0J56_RS13630 | nirS | nitrite reductase | 2.045715325 | 0.001826702 |
| C0J56_RS13675 |  | Crp/Fnr family transcriptional regulator | 1.990694112 | 0.040577724 |
| C0J56_RS24480 | cyoA | ubiquinol oxidase subunit II | 1.985460733 | 0.047811719 |
| C0J56_RS13635 | nirQ | CbbQ/NirQ/NorQ/GpvN family protein | 1.980409282 | 0.008428451 |
| C0J56_RS13695 | ytfE | iron-sulfur cluster repair protein YtfE | 1.967829377 | 0.003811607 |
| C0J56_RS24460 | cyoE | Protoheme IX farnesyltransferase | 1.929853749 | 0.017321162 |
| C0J56_RS24500 | hmpA | NO-inducible flavohemoprotein | 1.765019931 | 0.012749349 |
| C0J56_RS13765 | narH | nitrate reductase subunit beta | 1.605886992 | 0.009064397 |
| C0J56_RS13670 |  | protein DnrP | 1.525936662 | 0.01724175 |
| C0J56_RS27730 |  | electron transfer flavoprotein subunit beta | -1.978360689 | 0.028125788 |
| C0J56_RS09305 | nasA | nitrate reductase | -2.019240181 | 0.039532511 |
| C0J56_RS28135 |  | FAD-dependent oxidoreductase | -1.789708954 | 0.037254112 |
| C0J56_RS08005 | speB | agmatinase | -1.812814952 | 0.040222844 |
| C0J56_RS07590 |  | glutamine synthetase | -1.880835977 | 0.028828196 |
| C0J56_RS24575 |  | FAD-binding oxidoreductase | -1.969496635 | 0.016420208 |
| C0J56_RS27670 |  | serine hydroxymethyltransferase | -2.000140878 | 0.042448551 |
| C0J56_RS17675 | argH | Argininosuccinate lyase | -2.085291052 | 0.036076642 |
| C0J56_RS10545 |  | FAD-binding oxidoreductase | -2.311231757 | 0.024527028 |
| C0J56_RS15445 |  | 2-dehydro-3-deoxyglucarate aldolase | -2.363837188 | 0.022423041 |
| C0J56_RS13490 |  | 4-hydroxyproline epimerase | -2.399066174 | 0.01589112 |
| C0J56_RS13485 |  | FAD-binding oxidoreductase | -2.520431101 | 0.013768473 |
| C0J56_RS00635 | oadA | oxaloacetate decarboxylase subunit alpha | 1.543348637 | 0.029453816 |
| C0J56_RS04810 | garD | galactarate dehydratase | -1.836741669 | 0.033712023 |
| C0J56_RS13085 |  | dihydroxy-acid dehydratase | -1.908439996 | 0.039842803 |
| C0J56_RS19585 |  | NAD-dependent epimerase | -1.973842691 | 0.048339969 |
| C0J56_RS04800 |  | Aldehyde dehydrogenase family protein | -2.078234916 | 0.042943848 |
| C0J56_RS10980 | gudD | glucarate dehydratase | -2.260766177 | 0.040299701 |
| C0J56_RS21515 |  | glycerate kinase | -2.416250925 | 0.015591685 |
| C0J56_RS11805 |  | SDR family NAD(P)-dependent oxidoreductase | -2.508566113 | 0.008719573 |
| C0J56_RS23805 | nagA | N-acetylglucosamine-6-phosphate deacetylase | -2.579993125 | 0.006767315 |
| C0J56_RS13890 |  | AP endonuclease | -2.648333016 | 0.011760217 |
| C0J56_RS13405 |  | thiamine pyrophosphate-dependent dehydrogenase E1 component subunit alpha | -1.933205107 | 0.034249771 |
| C0J56_RS13410 |  | alpha-ketoacid dehydrogenase subunit beta | -2.324108713 | 0.030452521 |
| C0J56_RS28195 | mdcA | malonate decarboxylase subunit alpha | -1.556871142 | 0.019971926 |
| C0J56_RS28190 |  | triphosphoribosyl-dephospho-CoA synthase | -2.132421817 | 0.016473948 |
| C0J56_RS28185 |  | malonate decarboxylase subunit delta | -2.446216236 | 0.01083747 |
| C0J56_RS17785 | ureG | urease accessory protein UreG | -2.194973258 | 0.035409771 |
| C0J56_RS03420 |  | urease accessory protein UreF | -2.214014757 | 0.024327596 |
| C0J56_RS17765 | ureE | urease accessory protein UreE | -2.214175357 | 0.049534429 |
| C0J56_RS22090 |  | Peptidase P60 | 2.2396219 | 0.009893017 |
| C0J56_RS28290 |  | NlpC/P60 family protein | 2.006102826 | 0.034355737 |
| C0J56_RS14760 |  | matrixin family metalloprotease | 1.700682784 | 0.008110332 |
| C0J56_RS04265 |  | M20 family peptidase | -2.424245032 | 0.017103121 |
| C0J56_RS23710 |  | PadR family transcriptional regulator | 1.481298852 | 0.029847323 |
| C0J56_RS15430 |  | LysR family transcriptional regulator | -1.437679766 | 0.026542503 |
| C0J56_RS14200 |  | LysR family transcriptional regulator | -1.564133547 | 0.036580197 |
| C0J56_RS04095 |  | MurR/RpiR family transcriptional regulator | -1.723935083 | 0.040024235 |
| C0J56_RS00760 |  | AraC family transcriptional regulator | -1.741056765 | 0.046841098 |
| C0J56_RS02765 |  | LysR family transcriptional regulator | -1.763164617 | 0.035723088 |
| C0J56_RS04055 |  | LysR family transcriptional regulator | -1.93517387 | 0.027458586 |
| C0J56_RS17545 |  | sigma-54-dependent Fis family transcriptional regulator | -2.181810365 | 0.044186635 |
| C0J56_RS19465 |  | MarR family transcriptional regulator | -2.273129536 | 0.003264927 |
| C0J56_RS01880 |  | protein BsmA | 3.432100964 | 0.000188461 |
| C0J56_RS22590 |  | DUF2790 domain-containing protein | 3.011165181 | 0.000313577 |
| C0J56_RS24845 |  | hypothetical protein | 2.677362969 | 0.011228443 |
| C0J56_RS22595 |  | hypothetical protein | 2.476807537 | 0.000221306 |
| C0J56_RS06405 |  | Cold shock protein | 2.145733573 | 0.045249386 |
| C0J56_RS18740 |  | aldo/keto reductase family oxidoreductase | -2.329582478 | 0.020142085 |
| C0J56_RS01895 |  | AzlD domain-containing protein | 1.798022287 | 0.015069213 |
| C0J56_RS24985 | ispE | 4-(cytidine 5'-diphospho)-2-C-methyl-D-erythritol kinase | 1.757445774 | 0.01585001 |
| C0J56_RS03595 |  | DUF2946 domain-containing protein | 1.75584853 | 0.039053642 |
| C0J56_RS26100 |  | DUF2788 domain-containing protein | 1.564559953 | 0.047491427 |
| C0J56_RS08960 |  | DEAD/DEAH box helicase | 1.466342971 | 0.013890955 |
| C0J56_RS07175 |  | class C beta-lactamase-related serine hydrolase | -1.678832171 | 0.043538715 |
| C0J56_RS02670 |  | hypothetical protein | -1.716970187 | 0.049584071 |
| C0J56_RS00720 |  | PA0069 family radical SAM protein | -1.730887367 | 0.045171688 |
| C0J56_RS16855 |  | hypothetical protein | -1.745242609 | 0.029043841 |
| C0J56_RS15360 |  | Sensor histidine kinase | -1.74621082 | 0.03282498 |
| C0J56_RS05155 |  | TonB-dependent receptor | -1.861749798 | 0.042311726 |
| C0J56_RS14895 |  | alpha/beta hydrolase | -1.86890704 | 0.040060151 |
| C0J56_RS19595 |  | cobalamin biosynthesis protein CobQ | -1.93493035 | 0.048393608 |
| C0J56_RS06225 |  | Baseplate J/gp47 family protein | -1.972409091 | 0.040505144 |
| C0J56_RS17555 |  | amidohydrolase | -2.007851214 | 0.042582553 |
| C0J56_RS14580 |  | hypothetical protein | -2.008362516 | 0.01595828 |
| C0J56_RS09300 | cobA | uroporphyrin-III C-methyltransferase | -2.012589283 | 0.033597203 |
| C0J56_RS13350 |  | DUF2063 domain-containing protein | -2.059947415 | 0.045404829 |
| C0J56_RS00420 |  | hypothetical protein | -2.094806894 | 0.018782585 |
| C0J56_RS13905 |  | alkene reductase | -2.100118436 | 0.042882528 |
| C0J56_RS19625 |  | glycosyltransferase family 2 protein | -2.115837884 | 0.029814987 |
| C0J56_RS07160 |  | 1-acyl-sn-glycerol-3-phosphate acyltransferase | -2.134068742 | 0.04142194 |
| C0J56_RS28695 |  | transglycosylase | -2.151817098 | 0.049245969 |
| C0J56_RS19115 |  | DoxX family protein | -2.15288414 | 0.040915119 |
| C0J56_RS14295 |  | adhesin | -2.154775159 | 0.035590871 |
| C0J56_RS10555 |  | (2Fe-2S)-binding protein | -2.162258781 | 0.031120284 |
| C0J56_RS15175 |  | sll0787 family AIR synthase-like protein | -2.167919692 | 0.038146714 |
| C0J56_RS28690 |  | class A beta-lactamase-related serine hydrolase | -2.181374328 | 0.034590945 |
| C0J56_RS06220 |  | hypothetical protein | -2.21633027 | 0.042023908 |
| C0J56_RS10550 |  | FAD/NAD(P)-binding oxidoreductase | -2.221931898 | 0.037336869 |
| C0J56_RS01615 |  | SfnB family sulfur acquisition oxidoreductase | -2.247778541 | 0.027764065 |
| C0J56_RS14590 |  | hypothetical protein | -2.312340108 | 0.041331877 |
| C0J56_RS03760 |  | DUF4123 domain-containing protein | -2.360254439 | 0.025540936 |
| C0J56_RS12665 |  | Gfo/Idh/MocA family oxidoreductase | -2.367131682 | 0.018414184 |
| C0J56_RS17550 | ssuD | FMNH2-dependent alkanesulfonate monooxygenase | -2.3708672 | 0.012908343 |
| C0J56_RS18440 |  | acyl-CoA dehydrogenase | -2.402735823 | 0.014091756 |
| C0J56_RS13455 |  | DUF521 domain-containing protein | -2.414212991 | 0.012658503 |
| C0J56_RS13435 |  | hypothetical protein | -2.427046051 | 0.02067989 |
| C0J56_RS13910 |  | pirin family protein | -2.438210614 | 0.043673166 |
| C0J56_RS29080 |  | DUF1425 domain-containing protein | -2.563936324 | 0.020776457 |
| C0J56_RS15890 |  | Energy transducer TonB | -2.572934741 | 0.023447673 |
| C0J56_RS13520 |  | SDR family oxidoreductase | -2.581993273 | 0.007089264 |
| C0J56_RS14665 |  | SDR family oxidoreductase | -2.596257549 | 0.016376016 |
| C0J56_RS13340 |  | EF-hand domain-containing protein | -2.692727774 | 0.042861603 |
| C0J56_RS15165 |  | MSMEG_0568 family radical SAM protein | -2.700006566 | 0.008763613 |
| C0J56_RS16295 |  | NAD-dependent epimerase/dehydratase family protein | -2.754780274 | 0.004204249 |
| C0J56_RS11855 |  | SDR family oxidoreductase | -2.764707721 | 0.013243519 |
| C0J56_RS17560 |  | hypothetical protein | -2.766634336 | 0.038305378 |
| C0J56_RS27805 | cdhC | 3-keto-5-aminohexanoate cleavage protein | -2.815528167 | 0.014662041 |
| C0J56_RS13925 |  | RidA family protein | -2.956127741 | 0.021385059 |
| C0J56_RS19610 |  | glycosyltransferase family 1 protein | -3.071926964 | 0.007901968 |
| Predicted_antisense_transcript-100 |  |  | -2.27940076 | 0.031069017 |
| Predicted_antisense_transcript-1000 |  |  | -3.500263419 | 0.016928132 |
| Predicted_antisense_transcript-1003 |  |  | -3.462325403 | 0.008589237 |
| Predicted_antisense_transcript-1004 |  |  | -2.7070657 | 0.026270475 |
| Predicted_antisense_transcript-1008 |  |  | -4.166122958 | 0.017812274 |
| Predicted_antisense_transcript-1009 |  |  | -2.364127322 | 0.048879305 |
| Predicted_antisense_transcript-1010 |  |  | -3.237331272 | 0.028219997 |
| Predicted_antisense_transcript-1011 |  |  | -3.696294493 | 0.036359585 |
| Predicted_antisense_transcript-1019 |  |  | -3.09256147 | 0.013328702 |
| Predicted_antisense_transcript-1021 |  |  | -2.61517585 | 0.015174512 |
| Predicted_antisense_transcript-1022 |  |  | -2.777575909 | 0.014374061 |
| Predicted_antisense_transcript-1024 |  |  | -3.04651102 | 0.022708723 |
| Predicted_antisense_transcript-1031 |  |  | -3.457950088 | 0.011482683 |
| Predicted_antisense_transcript-1034 |  |  | -2.559689735 | 0.028654164 |
| Predicted_antisense_transcript-1037 |  |  | -2.300548526 | 0.047110828 |
| Predicted_antisense_transcript-1042 |  |  | -2.982720333 | 0.034925223 |
| Predicted_antisense_transcript-1043 |  |  | -3.076006717 | 0.011085112 |
| Predicted_antisense_transcript-1044 |  |  | -2.627640524 | 0.014999522 |
| Predicted_antisense_transcript-1045 |  |  | -2.021767198 | 0.030743141 |
| Predicted_antisense_transcript-1049 |  |  | -3.100689043 | 0.018409313 |
| Predicted_antisense_transcript-1050 |  |  | -2.638686446 | 0.034534636 |
| Predicted_antisense_transcript-1051 |  |  | -4.272454336 | 0.007385664 |
| Predicted_antisense_transcript-1058 |  |  | -3.471370531 | 0.00236873 |
| Predicted_antisense_transcript-1059 |  |  | -2.277997548 | 0.034355071 |
| Predicted_antisense_transcript-1077 |  |  | -2.924656441 | 0.029069697 |
| Predicted_antisense_transcript-108 |  |  | -2.641185773 | 0.036169419 |
| Predicted_antisense_transcript-1080 |  |  | -2.810170897 | 0.006824472 |
| Predicted_antisense_transcript-1088 |  |  | -4.101171518 | 0.017252316 |
| Predicted_antisense_transcript-1095 |  |  | -3.915472277 | 0.012144967 |
| Predicted_antisense_transcript-110 |  |  | -2.687466426 | 0.012148306 |
| Predicted_antisense_transcript-1100 |  |  | -2.981431436 | 0.012620706 |
| Predicted_antisense_transcript-1102 |  |  | -2.330283004 | 0.025153432 |
| Predicted_antisense_transcript-1107 |  |  | -2.495642837 | 0.045691236 |
| Predicted_antisense_transcript-1115 |  |  | -3.237550945 | 0.025028542 |
| Predicted_antisense_transcript-1121 |  |  | -2.566847597 | 0.012434791 |
| Predicted_antisense_transcript-1122 |  |  | -2.668455827 | 0.040860475 |
| Predicted_antisense_transcript-1126 |  |  | -2.258343101 | 0.030347665 |
| Predicted_antisense_transcript-1129 |  |  | -2.790252708 | 0.015438519 |
| Predicted_antisense_transcript-1133 |  |  | -3.188843226 | 0.031131183 |
| Predicted_antisense_transcript-1134 |  |  | -3.484207595 | 0.019003286 |
| Predicted_antisense_transcript-1135 |  |  | -3.118764805 | 0.014672358 |
| Predicted_antisense_transcript-1136 |  |  | -3.432338623 | 0.038668691 |
| Predicted_antisense_transcript-1137 |  |  | -4.63062095 | 0.02899014 |
| Predicted_antisense_transcript-114 |  |  | -3.18775948 | 0.003384418 |
| Predicted_antisense_transcript-1140 |  |  | -2.533660783 | 0.017534377 |
| Predicted_antisense_transcript-1142 |  |  | 2.489751142 | 0.025937873 |
| Predicted_antisense_transcript-1145 |  |  | -1.936958302 | 0.024176182 |
| Predicted_antisense_transcript-1146 |  |  | -2.154763474 | 0.014949149 |
| Predicted_antisense_transcript-1147 |  |  | -3.39796636 | 0.044806076 |
| Predicted_antisense_transcript-1148 |  |  | -3.745742121 | 0.004123768 |
| Predicted_antisense_transcript-1151 |  |  | -3.260350794 | 0.039465392 |
| Predicted_antisense_transcript-1152 |  |  | -3.073182833 | 0.022279912 |
| Predicted_antisense_transcript-1161 |  |  | -3.990773596 | 0.009710996 |
| Predicted_antisense_transcript-1162 |  |  | -3.013162612 | 0.016297086 |
| Predicted_antisense_transcript-1166 |  |  | -2.461175727 | 0.027245683 |
| Predicted_antisense_transcript-1168 |  |  | -3.4478447 | 0.007834415 |
| Predicted_antisense_transcript-1172 |  |  | -3.076500029 | 0.008104125 |
| Predicted_antisense_transcript-1173 |  |  | -2.549134195 | 0.03975081 |
| Predicted_antisense_transcript-1175 |  |  | -3.009691409 | 0.019469293 |
| Predicted_antisense_transcript-1176 |  |  | -2.810092764 | 0.021983981 |
| Predicted_antisense_transcript-1179 |  |  | -3.9306401 | 0.027108264 |
| Predicted_antisense_transcript-118 |  |  | -2.338606037 | 0.036620002 |
| Predicted_antisense_transcript-1183 |  |  | -2.795800988 | 0.015242534 |
| Predicted_antisense_transcript-1184 |  |  | -4.475009903 | 0.000903293 |
| Predicted_antisense_transcript-1186 |  |  | -3.755202314 | 0.001333673 |
| Predicted_antisense_transcript-1188 |  |  | -2.869775459 | 0.032528728 |
| Predicted_antisense_transcript-119 |  |  | -3.057503728 | 0.031975907 |
| Predicted_antisense_transcript-1193 |  |  | -2.646201874 | 0.038095457 |
| Predicted_antisense_transcript-1196 |  |  | -2.08355791 | 0.042136574 |
| Predicted_antisense_transcript-120 |  |  | -3.153397715 | 0.009705492 |
| Predicted_antisense_transcript-1206 |  |  | -2.369603326 | 0.024996223 |
| Predicted_antisense_transcript-1208 |  |  | -2.59347431 | 0.0101144 |
| Predicted_antisense_transcript-1210 |  |  | -2.470285469 | 0.02321626 |
| Predicted_antisense_transcript-1215 |  |  | -3.279357949 | 0.011548265 |
| Predicted_antisense_transcript-1216 |  |  | -2.801865789 | 0.047191135 |
| Predicted_antisense_transcript-1217 |  |  | -2.127562086 | 0.028570807 |
| Predicted_antisense_transcript-1219 |  |  | -2.988881265 | 0.026825657 |
| Predicted_antisense_transcript-1223 |  |  | -2.776313357 | 0.010791989 |
| Predicted_antisense_transcript-1238 |  |  | -2.626449902 | 0.041130055 |
| Predicted_antisense_transcript-124 |  |  | -2.31146339 | 0.021124562 |
| Predicted_antisense_transcript-1241 |  |  | -2.383286884 | 0.040512175 |
| Predicted_antisense_transcript-1243 |  |  | -2.380145947 | 0.031587966 |
| Predicted_antisense_transcript-1249 |  |  | -2.686161838 | 0.031238814 |
| Predicted_antisense_transcript-1250 |  |  | -2.834488837 | 0.011363781 |
| Predicted_antisense_transcript-1253 |  |  | -3.896261107 | 0.023026398 |
| Predicted_antisense_transcript-1254 |  |  | -2.84965461 | 0.043406681 |
| Predicted_antisense_transcript-1257 |  |  | -3.150121556 | 0.00517259 |
| Predicted_antisense_transcript-1265 |  |  | -3.342156502 | 0.020638564 |
| Predicted_antisense_transcript-1266 |  |  | -2.541134065 | 0.045941266 |
| Predicted_antisense_transcript-1269 |  |  | -2.793430345 | 0.016041472 |
| Predicted_antisense_transcript-127 |  |  | -2.502931152 | 0.033711059 |
| Predicted_antisense_transcript-1270 |  |  | -2.68800911 | 0.040969114 |
| Predicted_antisense_transcript-1272 |  |  | -3.075676291 | 0.026531628 |
| Predicted_antisense_transcript-1277 |  |  | -2.794896078 | 0.024944023 |
| Predicted_antisense_transcript-1284 |  |  | -2.064026221 | 0.049244387 |
| Predicted_antisense_transcript-1289 |  |  | -2.398679824 | 0.038868636 |
| Predicted_antisense_transcript-1292 |  |  | -2.698073968 | 0.030892655 |
| Predicted_antisense_transcript-1294 |  |  | -2.894684548 | 0.019560322 |
| Predicted_antisense_transcript-1297 |  |  | -3.256663164 | 0.003321742 |
| Predicted_antisense_transcript-1298 |  |  | -3.064157601 | 0.039671271 |
| Predicted_antisense_transcript-1299 |  |  | -2.917243903 | 0.028662429 |
| Predicted_antisense_transcript-130 |  |  | -3.266439439 | 0.024557105 |
| Predicted_antisense_transcript-1307 |  |  | -3.162846041 | 0.021235478 |
| Predicted_antisense_transcript-1312 |  |  | -3.780063131 | 0.024195302 |
| Predicted_antisense_transcript-1313 |  |  | -3.139459118 | 0.028021552 |
| Predicted_antisense_transcript-1314 |  |  | -3.070424919 | 0.004286705 |
| Predicted_antisense_transcript-1315 |  |  | -2.407909569 | 0.048997474 |
| Predicted_antisense_transcript-1318 |  |  | -2.60668129 | 0.03824573 |
| Predicted_antisense_transcript-1322 |  |  | -3.284535737 | 0.015514304 |
| Predicted_antisense_transcript-1323 |  |  | -3.244751707 | 0.013341054 |
| Predicted_antisense_transcript-1327 |  |  | -2.527286836 | 0.046016092 |
| Predicted_antisense_transcript-1328 |  |  | -3.100077104 | 0.027318101 |
| Predicted_antisense_transcript-1333 |  |  | -2.914165803 | 0.025363986 |
| Predicted_antisense_transcript-1334 |  |  | -2.019591094 | 0.043206155 |
| Predicted_antisense_transcript-1336 |  |  | -2.481412486 | 0.037668987 |
| Predicted_antisense_transcript-1340 |  |  | -3.234635987 | 0.002617999 |
| Predicted_antisense_transcript-1343 |  |  | -2.392202373 | 0.038901705 |
| Predicted_antisense_transcript-1347 |  |  | -3.786279591 | 0.00757265 |
| Predicted_antisense_transcript-1348 |  |  | -3.490865258 | 0.001778581 |
| Predicted_antisense_transcript-1350 |  |  | -2.493145947 | 0.02391564 |
| Predicted_antisense_transcript-1353 |  |  | -3.001422816 | 0.014183858 |
| Predicted_antisense_transcript-1359 |  |  | -2.969201229 | 0.011558147 |
| Predicted_antisense_transcript-1360 |  |  | -3.855989791 | 0.02470649 |
| Predicted_antisense_transcript-1361 |  |  | -3.43568902 | 0.008707715 |
| Predicted_antisense_transcript-1363 |  |  | -3.512086348 | 0.014059331 |
| Predicted_antisense_transcript-1364 |  |  | -3.366129102 | 0.016093186 |
| Predicted_antisense_transcript-1365 |  |  | -3.013933607 | 0.013629743 |
| Predicted_antisense_transcript-1368 |  |  | -3.061629015 | 0.038453703 |
| Predicted_antisense_transcript-1369 |  |  | -2.237186176 | 0.03285157 |
| Predicted_antisense_transcript-1370 |  |  | -3.340695883 | 0.009971577 |
| Predicted_antisense_transcript-1372 |  |  | -2.678946349 | 0.024483155 |
| Predicted_antisense_transcript-1378 |  |  | -3.148262389 | 0.027648619 |
| Predicted_antisense_transcript-1379 |  |  | -3.628847141 | 0.004299698 |
| Predicted_antisense_transcript-1381 |  |  | -2.642527983 | 0.028531651 |
| Predicted_antisense_transcript-1385 |  |  | -2.751457169 | 0.028827718 |
| Predicted_antisense_transcript-139 |  |  | -2.795583828 | 0.049974675 |
| Predicted_antisense_transcript-1391 |  |  | -2.67595661 | 0.046402001 |
| Predicted_antisense_transcript-14 |  |  | -3.346029005 | 0.004548211 |
| Predicted_antisense_transcript-143 |  |  | -3.573788275 | 0.00464223 |
| Predicted_antisense_transcript-144 |  |  | -2.852768423 | 0.048429716 |
| Predicted_antisense_transcript-145 |  |  | -2.82246531 | 0.027470571 |
| Predicted_antisense_transcript-146 |  |  | -4.561612072 | 0.006046153 |
| Predicted_antisense_transcript-147 |  |  | -3.160336575 | 0.038504863 |
| Predicted_antisense_transcript-150 |  |  | -3.51201307 | 0.014540391 |
| Predicted_antisense_transcript-158 |  |  | -2.691184141 | 0.017678425 |
| Predicted_antisense_transcript-160 |  |  | -2.762834879 | 0.030997114 |
| Predicted_antisense_transcript-171 |  |  | -2.755319444 | 0.034205394 |
| Predicted_antisense_transcript-176 |  |  | -2.555236761 | 0.039639615 |
| Predicted_antisense_transcript-180 |  |  | -2.422247395 | 0.010764062 |
| Predicted_antisense_transcript-182 |  |  | -2.496675735 | 0.021251531 |
| Predicted_antisense_transcript-189 |  |  | -2.94083471 | 0.033484942 |
| Predicted_antisense_transcript-192 |  |  | -3.286879048 | 0.018677298 |
| Predicted_antisense_transcript-193 |  |  | -2.357977251 | 0.034946113 |
| Predicted_antisense_transcript-195 |  |  | -2.756468405 | 0.041954442 |
| Predicted_antisense_transcript-2 |  |  | -2.878217855 | 0.017456124 |
| Predicted_antisense_transcript-202 |  |  | -2.753951834 | 0.008889937 |
| Predicted_antisense_transcript-206 |  |  | -4.076810753 | 0.035080722 |
| Predicted_antisense_transcript-208 |  |  | -3.23328082 | 0.039692367 |
| Predicted_antisense_transcript-209 |  |  | -2.592311904 | 0.021206176 |
| Predicted_antisense_transcript-215 |  |  | -2.971307587 | 0.01692552 |
| Predicted_antisense_transcript-225 |  |  | -3.586395309 | 0.006209519 |
| Predicted_antisense_transcript-23 |  |  | -3.300008356 | 0.007535277 |
| Predicted_antisense_transcript-231 |  |  | -3.276688506 | 0.024006391 |
| Predicted_antisense_transcript-232 |  |  | -2.73197495 | 0.015931824 |
| Predicted_antisense_transcript-233 |  |  | -2.875437813 | 0.008105952 |
| Predicted_antisense_transcript-238 |  |  | -2.884469508 | 0.048652453 |
| Predicted_antisense_transcript-239 |  |  | -3.930788478 | 0.005521356 |
| Predicted_antisense_transcript-24 |  |  | -2.970888146 | 0.013164796 |
| Predicted_antisense_transcript-240 |  |  | -3.67247184 | 0.013384526 |
| Predicted_antisense_transcript-244 |  |  | -3.110106595 | 0.004496936 |
| Predicted_antisense_transcript-245 |  |  | -3.970547715 | 0.002547592 |
| Predicted_antisense_transcript-25 |  |  | -2.40326125 | 0.024509883 |
| Predicted_antisense_transcript-253 |  |  | -2.635573674 | 0.01251746 |
| Predicted_antisense_transcript-258 |  |  | -1.944495626 | 0.042397776 |
| Predicted_antisense_transcript-260 |  |  | -2.436316902 | 0.047389257 |
| Predicted_antisense_transcript-263 |  |  | -3.095218095 | 0.004149014 |
| Predicted_antisense_transcript-265 |  |  | -2.826030282 | 0.026204292 |
| Predicted_antisense_transcript-266 |  |  | -4.626062937 | 0.021507547 |
| Predicted_antisense_transcript-271 |  |  | -3.176176343 | 0.019417358 |
| Predicted_antisense_transcript-272 |  |  | -2.431999778 | 0.022022959 |
| Predicted_antisense_transcript-273 |  |  | -3.686157991 | 0.006440599 |
| Predicted_antisense_transcript-275 |  |  | -2.807082238 | 0.035748168 |
| Predicted_antisense_transcript-276 |  |  | -3.577717743 | 0.016207506 |
| Predicted_antisense_transcript-28 |  |  | -3.41240096 | 0.006394628 |
| Predicted_antisense_transcript-281 |  |  | -2.618482638 | 0.036507447 |
| Predicted_antisense_transcript-282 |  |  | -2.836109359 | 0.038496913 |
| Predicted_antisense_transcript-286 |  |  | -3.152457344 | 0.011160105 |
| Predicted_antisense_transcript-289 |  |  | -2.7259305 | 0.020854486 |
| Predicted_antisense_transcript-291 |  |  | -2.053814285 | 0.044629027 |
| Predicted_antisense_transcript-292 |  |  | -3.799455503 | 0.002546591 |
| Predicted_antisense_transcript-293 |  |  | -2.559059415 | 0.020159933 |
| Predicted_antisense_transcript-294 |  |  | -3.181810634 | 0.012737917 |
| Predicted_antisense_transcript-295 |  |  | -3.036305757 | 0.035967426 |
| Predicted_antisense_transcript-300 |  |  | -2.039506322 | 0.046189747 |
| Predicted_antisense_transcript-302 |  |  | -3.659916431 | 0.003989882 |
| Predicted_antisense_transcript-303 |  |  | -3.690767046 | 0.0137819 |
| Predicted_antisense_transcript-309 |  |  | -2.806218026 | 0.007839109 |
| Predicted_antisense_transcript-31 |  |  | -3.23408645 | 0.011555628 |
| Predicted_antisense_transcript-315 |  |  | -2.607443012 | 0.038806857 |
| Predicted_antisense_transcript-317 |  |  | -2.227937096 | 0.018054795 |
| Predicted_antisense_transcript-318 |  |  | -2.704577367 | 0.030370094 |
| Predicted_antisense_transcript-320 |  |  | -2.792314869 | 0.022903457 |
| Predicted_antisense_transcript-322 |  |  | -3.211101262 | 0.02490248 |
| Predicted_antisense_transcript-324 |  |  | -3.29830282 | 0.028503124 |
| Predicted_antisense_transcript-326 |  |  | -2.198588331 | 0.043294641 |
| Predicted_antisense_transcript-327 |  |  | -3.032239353 | 0.042621902 |
| Predicted_antisense_transcript-329 |  |  | -3.105615826 | 0.033905027 |
| Predicted_antisense_transcript-333 |  |  | -3.138863352 | 0.029776526 |
| Predicted_antisense_transcript-338 |  |  | -2.959547917 | 0.006083197 |
| Predicted_antisense_transcript-341 |  |  | -2.686591548 | 0.021243097 |
| Predicted_antisense_transcript-344 |  |  | -2.500038084 | 0.013113898 |
| Predicted_antisense_transcript-346 |  |  | -3.432992771 | 0.01590546 |
| Predicted_antisense_transcript-349 |  |  | -2.606390147 | 0.048126883 |
| Predicted_antisense_transcript-351 |  |  | -2.883490869 | 0.020047446 |
| Predicted_antisense_transcript-353 |  |  | -3.167153315 | 0.006303165 |
| Predicted_antisense_transcript-36 |  |  | -3.917916624 | 0.008407871 |
| Predicted_antisense_transcript-362 |  |  | -2.666275652 | 0.013915496 |
| Predicted_antisense_transcript-364 |  |  | -2.073574582 | 0.039055071 |
| Predicted_antisense_transcript-365 |  |  | -3.256804237 | 0.015033505 |
| Predicted_antisense_transcript-366 |  |  | -2.789784185 | 0.013478802 |
| Predicted_antisense_transcript-371 |  |  | -1.937868468 | 0.043572657 |
| Predicted_antisense_transcript-372 |  |  | -2.615925424 | 0.025722599 |
| Predicted_antisense_transcript-376 |  |  | -3.06969377 | 0.010000465 |
| Predicted_antisense_transcript-377 |  |  | -2.349524731 | 0.033696308 |
| Predicted_antisense_transcript-38 |  |  | -3.698801678 | 0.029298507 |
| Predicted_antisense_transcript-382 |  |  | -2.155496768 | 0.046495821 |
| Predicted_antisense_transcript-387 |  |  | -2.952107955 | 0.020002716 |
| Predicted_antisense_transcript-389 |  |  | -3.155488397 | 0.010742114 |
| Predicted_antisense_transcript-393 |  |  | -2.961430201 | 0.022784585 |
| Predicted_antisense_transcript-395 |  |  | -2.800612307 | 0.020035051 |
| Predicted_antisense_transcript-396 |  |  | -3.863846935 | 0.00510601 |
| Predicted_antisense_transcript-397 |  |  | -2.775481009 | 0.028227536 |
| Predicted_antisense_transcript-398 |  |  | -2.130814192 | 0.038490224 |
| Predicted_antisense_transcript-405 |  |  | -3.338904905 | 0.012483279 |
| Predicted_antisense_transcript-406 |  |  | -4.193930718 | 0.00236977 |
| Predicted_antisense_transcript-411 |  |  | -2.8091467 | 0.011274115 |
| Predicted_antisense_transcript-412 |  |  | -2.88079207 | 0.004350535 |
| Predicted_antisense_transcript-42 |  |  | -3.792058788 | 0.0089486 |
| Predicted_antisense_transcript-420 |  |  | -3.329803155 | 0.034618309 |
| Predicted_antisense_transcript-422 |  |  | -2.253148078 | 0.031442295 |
| Predicted_antisense_transcript-434 |  |  | -3.302662063 | 0.004579175 |
| Predicted_antisense_transcript-435 |  |  | -3.755123936 | 0.032888318 |
| Predicted_antisense_transcript-44 |  |  | -2.129049034 | 0.044151277 |
| Predicted_antisense_transcript-442 |  |  | -3.183211304 | 0.02972399 |
| Predicted_antisense_transcript-444 |  |  | -2.408242763 | 0.020650725 |
| Predicted_antisense_transcript-451 |  |  | -2.676112122 | 0.007561332 |
| Predicted_antisense_transcript-456 |  |  | -3.856437357 | 0.028397369 |
| Predicted_antisense_transcript-459 |  |  | -3.303570636 | 0.009658157 |
| Predicted_antisense_transcript-460 |  |  | 2.003125076 | 0.036756235 |
| Predicted_antisense_transcript-461 |  |  | -2.977916442 | 0.016964795 |
| Predicted_antisense_transcript-465 |  |  | -3.470325729 | 0.039625601 |
| Predicted_antisense_transcript-472 |  |  | -3.101501888 | 0.043976945 |
| Predicted_antisense_transcript-474 |  |  | -2.933751932 | 0.024790014 |
| Predicted_antisense_transcript-478 |  |  | -2.152530423 | 0.014025701 |
| Predicted_antisense_transcript-481 |  |  | -2.121587736 | 0.043429527 |
| Predicted_antisense_transcript-483 |  |  | -2.751499115 | 0.029724988 |
| Predicted_antisense_transcript-49 |  |  | -2.356536346 | 0.038865765 |
| Predicted_antisense_transcript-492 |  |  | -3.734779068 | 0.006569406 |
| Predicted_antisense_transcript-493 |  |  | -2.721996547 | 0.038660852 |
| Predicted_antisense_transcript-501 |  |  | -3.4471077 | 0.018467847 |
| Predicted_antisense_transcript-502 |  |  | -2.439901084 | 0.025233384 |
| Predicted_antisense_transcript-506 |  |  | -2.661756967 | 0.013465403 |
| Predicted_antisense_transcript-51 |  |  | -2.800288144 | 0.014566397 |
| Predicted_antisense_transcript-512 |  |  | -2.55662825 | 0.027637713 |
| Predicted_antisense_transcript-513 |  |  | -2.665504799 | 0.029254761 |
| Predicted_antisense_transcript-514 |  |  | -3.174041663 | 0.013160961 |
| Predicted_antisense_transcript-526 |  |  | -2.08226585 | 0.049230956 |
| Predicted_antisense_transcript-530 |  |  | -2.497221341 | 0.04267525 |
| Predicted_antisense_transcript-532 |  |  | -2.377977134 | 0.033447365 |
| Predicted_antisense_transcript-534 |  |  | -3.056067255 | 0.015269133 |
| Predicted_antisense_transcript-536 |  |  | -2.6958226 | 0.020214921 |
| Predicted_antisense_transcript-551 |  |  | -2.257350761 | 0.027811844 |
| Predicted_antisense_transcript-557 |  |  | -3.527938564 | 0.005775131 |
| Predicted_antisense_transcript-56 |  |  | -2.985591104 | 0.025402029 |
| Predicted_antisense_transcript-561 |  |  | -2.772523372 | 0.020013826 |
| Predicted_antisense_transcript-562 |  |  | -2.727241626 | 0.012733328 |
| Predicted_antisense_transcript-564 |  |  | -2.668985849 | 0.035509446 |
| Predicted_antisense_transcript-568 |  |  | -3.486046387 | 0.003052474 |
| Predicted_antisense_transcript-575 |  |  | -3.140237934 | 0.008226804 |
| Predicted_antisense_transcript-576 |  |  | -3.641438474 | 0.040658427 |
| Predicted_antisense_transcript-578 |  |  | -2.481486697 | 0.02392076 |
| Predicted_antisense_transcript-580 |  |  | -2.167111202 | 0.039985704 |
| Predicted_antisense_transcript-581 |  |  | -2.271156108 | 0.014881009 |
| Predicted_antisense_transcript-589 |  |  | -3.697751362 | 0.010009348 |
| Predicted_antisense_transcript-59 |  |  | -2.75687437 | 0.041409023 |
| Predicted_antisense_transcript-593 |  |  | -3.342660243 | 0.042963655 |
| Predicted_antisense_transcript-598 |  |  | -2.382753314 | 0.041466181 |
| Predicted_antisense_transcript-599 |  |  | -3.839889158 | 0.019327167 |
| Predicted_antisense_transcript-6 |  |  | -2.982965128 | 0.012503899 |
| Predicted_antisense_transcript-60 |  |  | -3.715377233 | 0.014975426 |
| Predicted_antisense_transcript-600 |  |  | -2.736361386 | 0.039301728 |
| Predicted_antisense_transcript-601 |  |  | -3.459812443 | 0.011974866 |
| Predicted_antisense_transcript-604 |  |  | -2.611586017 | 0.013109041 |
| Predicted_antisense_transcript-606 |  |  | -2.319072174 | 0.042723538 |
| Predicted_antisense_transcript-607 |  |  | -3.61737951 | 0.004767508 |
| Predicted_antisense_transcript-608 |  |  | -2.443064169 | 0.0406214 |
| Predicted_antisense_transcript-618 |  |  | -3.376462893 | 0.01277666 |
| Predicted_antisense_transcript-622 |  |  | -2.91597189 | 0.048524429 |
| Predicted_antisense_transcript-624 |  |  | -3.169780133 | 0.024986576 |
| Predicted_antisense_transcript-625 |  |  | -3.189100585 | 0.016820408 |
| Predicted_antisense_transcript-626 |  |  | -3.622516144 | 0.024067315 |
| Predicted_antisense_transcript-627 |  |  | -2.779605535 | 0.03613255 |
| Predicted_antisense_transcript-628 |  |  | -2.777221218 | 0.017268391 |
| Predicted_antisense_transcript-629 |  |  | -2.406426242 | 0.026714595 |
| Predicted_antisense_transcript-63 |  |  | -2.791965215 | 0.021676863 |
| Predicted_antisense_transcript-631 |  |  | -2.891149433 | 0.038715663 |
| Predicted_antisense_transcript-634 |  |  | -3.769712165 | 0.002672237 |
| Predicted_antisense_transcript-636 |  |  | -3.641585403 | 0.034083488 |
| Predicted_antisense_transcript-637 |  |  | -3.037146679 | 0.021697607 |
| Predicted_antisense_transcript-639 |  |  | -2.196489637 | 0.039320539 |
| Predicted_antisense_transcript-641 |  |  | -2.376278581 | 0.046998757 |
| Predicted_antisense_transcript-642 |  |  | -3.082066873 | 0.028553639 |
| Predicted_antisense_transcript-65 |  |  | -3.693096166 | 0.01506893 |
| Predicted_antisense_transcript-652 |  |  | -3.503544774 | 0.022637911 |
| Predicted_antisense_transcript-653 |  |  | -2.894625725 | 0.027807961 |
| Predicted_antisense_transcript-656 |  |  | -3.340295812 | 0.016364917 |
| Predicted_antisense_transcript-657 |  |  | -3.053845133 | 0.00651132 |
| Predicted_antisense_transcript-658 |  |  | -2.438253019 | 0.044395225 |
| Predicted_antisense_transcript-660 |  |  | -2.7118541 | 0.043553774 |
| Predicted_antisense_transcript-661 |  |  | -2.732873244 | 0.043593264 |
| Predicted_antisense_transcript-662 |  |  | -2.456558756 | 0.015819358 |
| Predicted_antisense_transcript-666 |  |  | -3.533219559 | 0.017582807 |
| Predicted_antisense_transcript-668 |  |  | -3.527039437 | 0.0132581 |
| Predicted_antisense_transcript-67 |  |  | -2.638938468 | 0.042923568 |
| Predicted_antisense_transcript-674 |  |  | -3.560597317 | 0.044688282 |
| Predicted_antisense_transcript-675 |  |  | -3.509450841 | 0.011842761 |
| Predicted_antisense_transcript-683 |  |  | -2.880275368 | 0.00862821 |
| Predicted_antisense_transcript-685 |  |  | -2.54254184 | 0.01078901 |
| Predicted_antisense_transcript-688 |  |  | -3.58583632 | 0.038276194 |
| Predicted_antisense_transcript-690 |  |  | -2.552687609 | 0.029530907 |
| Predicted_antisense_transcript-692 |  |  | -2.937539271 | 0.030684666 |
| Predicted_antisense_transcript-695 |  |  | -2.408091895 | 0.043695053 |
| Predicted_antisense_transcript-70 |  |  | -2.189639997 | 0.028108146 |
| Predicted_antisense_transcript-702 |  |  | -2.702939891 | 0.035563278 |
| Predicted_antisense_transcript-704 |  |  | -3.790002948 | 0.011871701 |
| Predicted_antisense_transcript-705 |  |  | -3.738750068 | 0.020294817 |
| Predicted_antisense_transcript-706 |  |  | -3.12993574 | 0.030212767 |
| Predicted_antisense_transcript-707 |  |  | -2.98562853 | 0.042289678 |
| Predicted_antisense_transcript-708 |  |  | -3.247252887 | 0.004331457 |
| Predicted_antisense_transcript-71 |  |  | -2.323348145 | 0.027077347 |
| Predicted_antisense_transcript-710 |  |  | -2.182597976 | 0.042033449 |
| Predicted_antisense_transcript-711 |  |  | -2.664289885 | 0.029797913 |
| Predicted_antisense_transcript-712 |  |  | -5.166712983 | 0.002814252 |
| Predicted_antisense_transcript-716 |  |  | -3.508926533 | 0.00942257 |
| Predicted_antisense_transcript-718 |  |  | -2.658745922 | 0.029045381 |
| Predicted_antisense_transcript-720 |  |  | -2.181069673 | 0.046841701 |
| Predicted_antisense_transcript-722 |  |  | -3.452853441 | 0.011845859 |
| Predicted_antisense_transcript-723 |  |  | -2.453473583 | 0.04665238 |
| Predicted_antisense_transcript-725 |  |  | -2.26540693 | 0.027137812 |
| Predicted_antisense_transcript-729 |  |  | -3.124779823 | 0.018320468 |
| Predicted_antisense_transcript-733 |  |  | -3.438696377 | 0.015437138 |
| Predicted_antisense_transcript-735 |  |  | -3.481717936 | 0.00446101 |
| Predicted_antisense_transcript-738 |  |  | -2.694025295 | 0.030722985 |
| Predicted_antisense_transcript-741 |  |  | -1.892761194 | 0.042714249 |
| Predicted_antisense_transcript-743 |  |  | -3.513486923 | 0.015598166 |
| Predicted_antisense_transcript-751 |  |  | -3.475071738 | 0.019060416 |
| Predicted_antisense_transcript-754 |  |  | -2.532307786 | 0.033779946 |
| Predicted_antisense_transcript-757 |  |  | -3.621273114 | 0.012692109 |
| Predicted_antisense_transcript-759 |  |  | -3.336839599 | 0.005311934 |
| Predicted_antisense_transcript-760 |  |  | -3.209223871 | 0.002385263 |
| Predicted_antisense_transcript-763 |  |  | -3.905996959 | 0.00245427 |
| Predicted_antisense_transcript-765 |  |  | -2.761480995 | 0.023859469 |
| Predicted_antisense_transcript-769 |  |  | -2.643588321 | 0.048898639 |
| Predicted_antisense_transcript-775 |  |  | -2.750174224 | 0.024116725 |
| Predicted_antisense_transcript-778 |  |  | -2.113878345 | 0.048573937 |
| Predicted_antisense_transcript-781 |  |  | -3.342260895 | 0.01813674 |
| Predicted_antisense_transcript-782 |  |  | -3.557749754 | 0.017908294 |
| Predicted_antisense_transcript-783 |  |  | -3.482504622 | 0.016903787 |
| Predicted_antisense_transcript-785 |  |  | -2.620656199 | 0.047448053 |
| Predicted_antisense_transcript-787 |  |  | -3.545025248 | 0.006798148 |
| Predicted_antisense_transcript-791 |  |  | -3.522207575 | 0.002589348 |
| Predicted_antisense_transcript-795 |  |  | -3.885309739 | 0.019725696 |
| Predicted_antisense_transcript-798 |  |  | -3.396483691 | 0.032677894 |
| Predicted_antisense_transcript-803 |  |  | -4.591249881 | 0.022258269 |
| Predicted_antisense_transcript-805 |  |  | -3.934086037 | 0.011839924 |
| Predicted_antisense_transcript-809 |  |  | -4.010129681 | 0.006833993 |
| Predicted_antisense_transcript-810 |  |  | -2.451529821 | 0.018777481 |
| Predicted_antisense_transcript-811 |  |  | -2.719444175 | 0.041286291 |
| Predicted_antisense_transcript-812 |  |  | -2.542901082 | 0.001585852 |
| Predicted_antisense_transcript-815 |  |  | -4.183664474 | 0.005371261 |
| Predicted_antisense_transcript-816 |  |  | -2.938921827 | 0.009817018 |
| Predicted_antisense_transcript-819 |  |  | -2.809331566 | 0.040339588 |
| Predicted_antisense_transcript-82 |  |  | -2.680592723 | 0.038366299 |
| Predicted_antisense_transcript-822 |  |  | -3.141673484 | 0.01204889 |
| Predicted_antisense_transcript-823 |  |  | -3.107669481 | 0.012725199 |
| Predicted_antisense_transcript-824 |  |  | -3.299306549 | 0.02170461 |
| Predicted_antisense_transcript-825 |  |  | -3.048341254 | 0.037033437 |
| Predicted_antisense_transcript-827 |  |  | -2.703581093 | 0.0433443 |
| Predicted_antisense_transcript-828 |  |  | -2.646931236 | 0.017867861 |
| Predicted_antisense_transcript-833 |  |  | -2.219813146 | 0.046316452 |
| Predicted_antisense_transcript-841 |  |  | -2.276269848 | 0.036325084 |
| Predicted_antisense_transcript-845 |  |  | -2.880626372 | 0.045632038 |
| Predicted_antisense_transcript-846 |  |  | -3.512602983 | 0.012298744 |
| Predicted_antisense_transcript-850 |  |  | -3.891980704 | 0.01031853 |
| Predicted_antisense_transcript-861 |  |  | -3.406013289 | 0.014702311 |
| Predicted_antisense_transcript-862 |  |  | -2.854519837 | 0.027412825 |
| Predicted_antisense_transcript-863 |  |  | -2.254516667 | 0.049468684 |
| Predicted_antisense_transcript-871 |  |  | -3.069443016 | 0.035180444 |
| Predicted_antisense_transcript-873 |  |  | -2.278861379 | 0.03463368 |
| Predicted_antisense_transcript-890 |  |  | -2.547661386 | 0.047725545 |
| Predicted_antisense_transcript-891 |  |  | -3.582432215 | 0.011964733 |
| Predicted_antisense_transcript-898 |  |  | -3.208063365 | 0.023765712 |
| Predicted_antisense_transcript-902 |  |  | -3.461497246 | 0.027065178 |
| Predicted_antisense_transcript-903 |  |  | -3.356985617 | 0.034221508 |
| Predicted_antisense_transcript-904 |  |  | -2.201524135 | 0.047003043 |
| Predicted_antisense_transcript-905 |  |  | -3.676777327 | 0.007742324 |
| Predicted_antisense_transcript-907 |  |  | -3.432896959 | 0.010739504 |
| Predicted_antisense_transcript-91 |  |  | -2.945981579 | 0.020478522 |
| Predicted_antisense_transcript-915 |  |  | -4.937888678 | 0.010843008 |
| Predicted_antisense_transcript-916 |  |  | -2.818136946 | 0.040368434 |
| Predicted_antisense_transcript-920 |  |  | 1.755449167 | 0.038581502 |
| Predicted_antisense_transcript-921 |  |  | -4.06781832 | 0.006912893 |
| Predicted_antisense_transcript-924 |  |  | -3.391832885 | 0.019729666 |
| Predicted_antisense_transcript-930 |  |  | -2.255798261 | 0.021989423 |
| Predicted_antisense_transcript-931 |  |  | -5.020958821 | 0.000998894 |
| Predicted_antisense_transcript-932 |  |  | -3.60107882 | 0.028651537 |
| Predicted_antisense_transcript-933 |  |  | -2.41470972 | 0.046851888 |
| Predicted_antisense_transcript-935 |  |  | -2.997279052 | 0.017533506 |
| Predicted_antisense_transcript-940 |  |  | -2.72186461 | 0.024302642 |
| Predicted_antisense_transcript-943 |  |  | -2.933125501 | 0.038471147 |
| Predicted_antisense_transcript-944 |  |  | -3.333808783 | 0.013235553 |
| Predicted_antisense_transcript-946 |  |  | -2.996883003 | 0.018295257 |
| Predicted_antisense_transcript-948 |  |  | -2.638075912 | 0.022741688 |
| Predicted_antisense_transcript-953 |  |  | -2.346792096 | 0.049045416 |
| Predicted_antisense_transcript-955 |  |  | -2.281681428 | 0.049555876 |
| Predicted_antisense_transcript-956 |  |  | -2.513348811 | 0.021209905 |
| Predicted_antisense_transcript-957 |  |  | -2.175798186 | 0.047485772 |
| Predicted_antisense_transcript-960 |  |  | -2.861623443 | 0.006734466 |
| Predicted_antisense_transcript-961 |  |  | -2.547302412 | 0.024912652 |
| Predicted_antisense_transcript-964 |  |  | -3.468991372 | 0.008207514 |
| Predicted_antisense_transcript-965 |  |  | -2.774510884 | 0.041415637 |
| Predicted_antisense_transcript-967 |  |  | -3.162353994 | 0.020919826 |
| Predicted_antisense_transcript-969 |  |  | -2.793089674 | 0.027114284 |
| Predicted_antisense_transcript-972 |  |  | -5.318518473 | 0.02767541 |
| Predicted_antisense_transcript-973 |  |  | -2.634394178 | 0.023935266 |
| Predicted_antisense_transcript-978 |  |  | -3.811692087 | 0.008570335 |
| Predicted_antisense_transcript-994 |  |  | -1.997687224 | 0.044337121 |
| Predicted_antisense_transcript-998 |  |  | -4.52085078 | 0.00075622 |
| Predicted_non_antisense_transcript-111 | |  | 2.300525495 | 0.041048099 |
| Predicted_non_antisense_transcript-128 | |  | -3.789026403 | 0.001647529 |
| Predicted_non_antisense_transcript-130 | |  | -2.778596276 | 0.033815469 |
| Predicted_non_antisense_transcript-132 | |  | 1.692799663 | 0.048179571 |
| Predicted_non_antisense_transcript-138 | |  | -2.943683658 | 0.034491827 |
| Predicted_non_antisense_transcript-150 | |  | -2.910477629 | 0.012136357 |
| Predicted_non_antisense_transcript-151 | |  | -2.228983952 | 0.042704028 |
| Predicted_non_antisense_transcript-153 | |  | -2.914124797 | 0.035803567 |
| Predicted_non_antisense_transcript-154 | |  | -3.023498315 | 0.010690853 |
| Predicted_non_antisense_transcript-159 | |  | -3.155820574 | 0.029438979 |
| Predicted_non_antisense_transcript-162 | |  | -2.704644013 | 0.02791965 |
| Predicted_non_antisense_transcript-164 | |  | -1.946402828 | 0.046346769 |
| Predicted_non_antisense_transcript-169 | |  | -2.948271448 | 0.014895557 |
| Predicted_non_antisense_transcript-171 | |  | -2.339514841 | 0.025038927 |
| Predicted_non_antisense_transcript-186 | |  | -4.266367371 | 0.002076515 |
| Predicted_non_antisense_transcript-187 | |  | -2.024313993 | 0.045192568 |
| Predicted_non_antisense_transcript-188 | |  | -3.313420669 | 0.008413037 |
| Predicted_non_antisense_transcript-189 | |  | -3.392568336 | 0.009781755 |
| Predicted_non_antisense_transcript-191 | |  | -3.215691635 | 0.031533647 |
| Predicted_non_antisense_transcript-192 | |  | -2.966568647 | 0.021550484 |
| Predicted_non_antisense_transcript-195 | |  | -2.394288616 | 0.045226182 |
| Predicted_non_antisense_transcript-197 | |  | -4.194596777 | 0.001903284 |
| Predicted_non_antisense_transcript-199 | |  | -2.565138473 | 0.046944857 |
| Predicted_non_antisense_transcript-213 | |  | -3.873155516 | 0.004872106 |
| Predicted_non_antisense_transcript-215 | |  | -4.861849145 | 0.000459936 |
| Predicted_non_antisense_transcript-217 | |  | -3.761187309 | 0.006420506 |
| Predicted_non_antisense_transcript-218 | |  | -2.808185333 | 0.008946474 |
| Predicted_non_antisense_transcript-223 | |  | 2.408833035 | 0.026908806 |
| Predicted_non_antisense_transcript-235 | |  | -2.221375248 | 0.029049709 |
| Predicted_non_antisense_transcript-239 | |  | -2.062996223 | 0.049888724 |
| Predicted_non_antisense_transcript-240 | |  | -2.895103164 | 0.035728378 |
| Predicted_non_antisense_transcript-246 | |  | 1.834984003 | 0.046023775 |
| Predicted_non_antisense_transcript-263 | |  | -2.564239348 | 0.038248464 |
| Predicted_non_antisense_transcript-264 | |  | -3.104123628 | 0.005966015 |
| Predicted_non_antisense_transcript-265 | |  | -4.088022926 | 0.001358863 |
| Predicted_non_antisense_transcript-268 | |  | -3.470601352 | 0.001393899 |
| Predicted_non_antisense_transcript-269 | |  | -4.915763384 | 0.000376061 |
| Predicted_non_antisense_transcript-270 | |  | -5.054174792 | 0.000240094 |
| Predicted_non_antisense_transcript-272 | |  | -3.979997166 | 0.001481063 |
| Predicted_non_antisense_transcript-273 | |  | -6.503659858 | 0.001445153 |
| Predicted_non_antisense_transcript-275 | |  | -5.12505561 | 4.25913E-06 |
| Predicted_non_antisense_transcript-276 | |  | -4.217348703 | 0.008958829 |
| Predicted_non_antisense_transcript-277 | |  | -4.582718784 | 0.000150779 |
| Predicted_non_antisense_transcript-278 | |  | -6.394622583 | 0.000245147 |
| Predicted_non_antisense_transcript-281 | |  | -4.01013676 | 3.79601E-05 |
| Predicted_non_antisense_transcript-282 | |  | -2.042441225 | 0.006851738 |
| Predicted_non_antisense_transcript-284 | |  | -4.365817501 | 5.77444E-06 |
| Predicted_non_antisense_transcript-297 | |  | 2.396443108 | 0.045685931 |
| Predicted_non_antisense_transcript-303 | |  | -2.900555349 | 0.022864596 |
| Predicted_non_antisense_transcript-304 | |  | -3.284966429 | 0.013313394 |
| Predicted_non_antisense_transcript-314 | |  | -1.60546237 | 0.042261743 |
| Predicted_non_antisense_transcript-317 | |  | -2.295948744 | 0.041360274 |
| Predicted_non_antisense_transcript-321 | |  | 3.083390393 | 0.002579629 |
| Predicted_non_antisense_transcript-336 | |  | -3.534029435 | 0.010263884 |
| Predicted_non_antisense_transcript-341 | |  | -2.692330678 | 0.018695249 |
| Predicted_non_antisense_transcript-347 | |  | 2.304170727 | 0.027775421 |
| Predicted_non_antisense_transcript-36 | |  | 2.443550747 | 0.034323161 |
| Predicted_non_antisense_transcript-374 | |  | 2.233349308 | 0.03357568 |
| Predicted_non_antisense_transcript-378 | |  | -2.821047312 | 0.048478914 |
| Predicted_non_antisense_transcript-379 | |  | 3.163912576 | 0.000700639 |
| Predicted_non_antisense_transcript-381 | |  | -2.800628969 | 0.016175115 |
| Predicted_non_antisense_transcript-388 | |  | -3.165840076 | 0.024408879 |
| Predicted_non_antisense_transcript-401 | |  | -2.281093065 | 0.044297505 |
| Predicted_non_antisense_transcript-406 | |  | -2.276587211 | 0.044269507 |
| Predicted_non_antisense_transcript-434 | |  | -2.222994102 | 0.033142896 |
| Predicted_non_antisense_transcript-437 | |  | -3.754086862 | 0.006005113 |
| Predicted_non_antisense_transcript-442 | |  | -2.361781383 | 0.037163991 |
| Predicted_non_antisense_transcript-443 | |  | -2.706952025 | 0.026436177 |
| Predicted_non_antisense_transcript-458 | |  | 3.291794205 | 0.041279306 |
| Predicted_non_antisense_transcript-53 | |  | -2.519315841 | 0.024691214 |
| Predicted_non_antisense_transcript-56 | |  | -2.497035615 | 0.013533594 |
| Predicted_non_antisense_transcript-57 | |  | -3.435112618 | 0.013326021 |
| Predicted_non_antisense_transcript-61 | |  | 1.808875869 | 0.044745889 |
| Predicted_non_antisense_transcript-62 | |  | 1.550723657 | 0.043030413 |
| Predicted_non_antisense_transcript-7 |  |  | 1.800239142 | 0.022010273 |
| Predicted_non_antisense_transcript-81 | |  | -3.027948519 | 0.032084691 |
| Predicted_non_antisense_transcript-82 | |  | -3.550980127 | 0.008541035 |
| Predicted_non_antisense_transcript-83 | |  | -2.602963237 | 0.039846942 |

The genes with fold change ≥2.0 with P value ≤0.05 as statistically significant in the performed RNA-seq analysis. The *P. fluorescens* 2P24 reference geneome (NZ_CP025542.1) was used for the analysis. The differentially expression level in the Δ*mupR* mutant was relative to that in the *P. fluorescens* 2P24.

Entries are shaded as follows:

Blue, proteins involved in mupirocin biosynthesis

Green, proteins involved in 2,4-DAPG biosynthesis

Red, proteins involved in pyoverdine biosynthesis

Orange, proteins involved in rRNA and tRNA biosynthesis

Pink, proteins involved in bacterial secretion system

Cyan, proteins involved in different type of transporters or membrane

Purple, proteins involved in nitrification, denitrification and electron transfer or redox

Brown, proteins involved in amino metabolism

Yellow, proteins involved in carbohydrate metabolsim

Dark red, proteins involved in malonate metabolism

Dark green, proteins involved in urea metabolism

Dark yellow, peptidase

Dark blue, transcriptional regulator

Gray, Non-annotated gene

**Table S6. The differentia intracellular metabolite concentrations of Δ*mupR* (mutant) compared to *P. fluorescence* 2P24 (wild-type) by metabolome analysis**

| **Metabolites** | **VIP** | **Average**  **(Δ*mupR*)** | **Average**  **(2P24)** | **log2FoldChange** | **Regulation** | **p-value** |
| --- | --- | --- | --- | --- | --- | --- |
| Berkeleylactone F | 2.170240878 | 0.006053133 | 155494.6892 | -24.61460813 | Down | 9.2E-10 |
| L-Olivosyl-Oleandolide | 1.978619862 | 0.006053133 | 128291.6573 | -24.33717018 | Down | 6.87E-10 |
| Daucic Acid | 1.257367742 | 0.006053133 | 52513.92483 | -23.04851475 | Down | 1.33E-08 |
| Meosuc-Ala-Ala-Pro-Val-Pna | 1.105226713 | 0.006053133 | 39742.09575 | -22.64648268 | Down | 1.82E-09 |
| L-Felinine | 1.30105026 | 10.72715476 | 55710.21343 | -12.34245866 | Down | 5.33E-10 |
| Acaulesterone | 1.652460601 | 27.11162222 | 88849.79493 | -11.67824129 | Down | 1.22E-09 |
| Val Asn Phe | 1.389268397 | 156.9112768 | 62643.73294 | -8.641077337 | Down | 8.49E-13 |
| Mupirocin | 3.370597832 | 1515.662047 | 371718.2741 | -7.938117698 | Down | 2.91E-11 |
| Benzoic Acid | 1.568523492 | 395.1028282 | 95915.31308 | -7.923389179 | Down | 0.001185 |
| Dioxinoacrimarine A | 1.278719439 | 295.235484 | 68789.97442 | -7.864188381 | Down | 0.002444 |
| Laricitrin 7-Glucoside | 1.190159284 | 262.429374 | 47516.41532 | -7.50035298 | Down | 1.23E-07 |
| 19:1(16z) | 1.05814952 | 455.8984576 | 46106.58561 | -6.660116493 | Down | 0.001296 |
| Isoferreirin | 2.034077806 | 2226.55748 | 169351.0679 | -6.249058418 | Down | 0.00142 |
| Peonidin 3-(6''-Malonylglucoside) | 1.514828809 | 1483.313892 | 102415.8093 | -6.109470696 | Down | 0.003919 |
| PC(P-16:0/20:3(6,8,11)-OH(5)) | 13.60600253 | 160633.8368 | 8711336.578 | -5.76104636 | Down | 0.004494 |
| 2,4-Diacetylphloroglucinol | 6.12386415 | 33430.20857 | 1485376.163 | -5.473532166 | Down | 0.000394 |
| Subaphylline | 1.281072747 | 1351.114704 | 58482.88248 | -5.435792356 | Down | 3.25E-07 |
| 7-Hydroxygranisetron | 1.439898544 | 1785.163869 | 69738.10458 | -5.287818736 | Down | 4.94E-11 |
| 8-Heptadecenal | 1.05463109 | 1051.10594 | 40218.24135 | -5.257870008 | Down | 1.95E-05 |
| 6-C-Methylquercetin 3,3'-Dimethyl Ether | 3.457067147 | 14772.78182 | 486709.2541 | -5.04204678 | Down | 0.000877 |
| N-Lactoylphenylalanine | 1.200290096 | 1565.419022 | 50955.22917 | -5.024609421 | Down | 1.28E-07 |
| 11-Cyclohexylundecanoic Acid | 1.586934367 | 3300.239398 | 92407.02937 | -4.807360015 | Down | 2.45E-05 |
| Vitisin B | 1.095572178 | 2608.365129 | 43428.43555 | -4.05742224 | Down | 1.09E-06 |
| PC(16:0/18:0) | 22.2279257 | 1154584.942 | 17466708.81 | -3.919161572 | Down | 6.58E-09 |
| B-D-Glucuronopyranosyl-(1->3)-A-D-Galacturonopyranosyl-(1->2)-L-Rhamnose | 1.751464414 | 7853.846711 | 111667.0856 | -3.829660757 | Down | 1.73E-06 |
| Melisimplexin | 19.00141033 | 1266465.136 | 14874301.52 | -3.553942655 | Down | 0.000305 |
| PE(14:0/24:1(15Z)) | 1.556896049 | 9014.290505 | 92211.85869 | -3.354666447 | Down | 1.23E-06 |
| Fluconazole | 1.236987403 | 9617.718174 | 58814.15143 | -2.612396771 | Down | 3.2E-10 |
| His Val Val | 1.078521045 | 7543.448024 | 46070.07056 | -2.610533789 | Down | 1.16E-06 |
| Andersoline | 1.66843769 | 22980.32929 | 128113.643 | -2.478952743 | Down | 0.000431 |
| Arginylproline | 4.765032866 | 170431.6874 | 931241.2448 | -2.449961364 | Down | 6.88E-07 |
| Sulpiride | 1.207405318 | 11053.60725 | 58073.12765 | -2.393353478 | Down | 3.71E-11 |
| PC(18:1(11Z)/22:6(4Z,7Z,10Z,13Z,16Z,19Z)) | 5.678125943 | 301931.7302 | 1578464.787 | -2.386227794 | Down | 0.000369 |
| Pisumoside B | 2.868121179 | 73037.0784 | 371718.0477 | -2.347507774 | Down | 0.000106 |
| Glu Phe | 1.918002237 | 39309.26659 | 172358.1461 | -2.132468134 | Down | 0.000155 |
| Serylthreonine | 1.424154891 | 26401.8339 | 105379.7588 | -1.996887733 | Down | 0.000417 |
| PC(20:4(8Z,11Z,14Z,17Z)/0:0) | 1.842219227 | 52951.49856 | 181854.5786 | -1.78004183 | Down | 0.000153 |
| PC(18:2(9Z,12Z)/0:0) | 2.235205696 | 80277.65399 | 270603.5748 | -1.753110537 | Down | 0.000169 |
| N,N-Bis(Allyl)-Tyr-Gly-Gly-Psi-Methylthio-Phe-Leu | 1.05848343 | 33005.73308 | 96720.14977 | -1.551099837 | Down | 0.039489 |
| Limocitrol 3-Neohesperidoside | 3.927287235 | 313540.9065 | 906595.5374 | -1.531805381 | Down | 0.000273 |
| 1-(2-Methoxy-20z-Heptacosenyl)-Sn-Glycero-3-Phosphoserine | 1.107431918 | 40087.16557 | 115065.8867 | -1.521247866 | Down | 0.030916 |
| S-Adenosylmethionine | 2.898076112 | 171159.1785 | 486713.5247 | -1.507734205 | Down | 8.31E-05 |
| PC(0:0/16:0) | 1.026606512 | 21996.58959 | 62077.06159 | -1.496780407 | Down | 0.000167 |
| Dextran-70 | 1.119260711 | 24152.75016 | 65580.50731 | -1.441079591 | Down | 1.41E-09 |
| N-Succinyl-Ala-Ala-Pro-Phe-P-Nitroanilide | 1.497491237 | 50520.88801 | 126983.3005 | -1.329686878 | Down | 2.05E-07 |
| 2-Methylhexanoyl-Coa | 1.257401991 | 44005.87816 | 108254.2622 | -1.298655676 | Down | 0.000918 |
| (S)C(S)S-S-Methylcysteine Sulfoxide | 1.188077725 | 35446.83521 | 85931.02235 | -1.277522234 | Down | 7.27E-06 |
| PC(16:0/0:0) | 2.738311908 | 208760.6216 | 504410.201 | -1.27274785 | Down | 0.000481 |
| PC(15:0/20:5(5Z,8Z,11Z,14Z,17Z)) | 1.874585041 | 109860.7866 | 248486.7928 | -1.177492646 | Down | 0.000245 |
| 5'-Methylthioadenosine | 3.509300168 | 343861.5668 | 761762.8625 | -1.14751408 | Down | 3.5E-08 |
| PC(18:1(9Z)/0:0) | 1.161080273 | 45892.38498 | 100429.1218 | -1.129850984 | Down | 0.001098 |
| Harman | 1.76833382 | 93051.13991 | 201075.8469 | -1.111644067 | Down | 4.99E-06 |
| Stearamide | 3.144222126 | 389696.4291 | 841213.1846 | -1.11012075 | Down | 0.004427 |
| Herbimycin A | 1.149019685 | 39948.12432 | 85809.0633 | -1.10300227 | Down | 4.76E-06 |
| PS(O-20:0/16:0) | 17.28668152 | 10230228.75 | 21748760.17 | -1.088094755 | Down | 0.000523 |
| PC(18:0/0:0) | 1.101350394 | 50381.54192 | 105146.1323 | -1.061428602 | Down | 0.006922 |
| (4r,5s,7r,11x)-11,12-Dihydroxy-1(10)-Spirovetiven-2-One 12-Glucoside | 1.016359588 | 32227.16709 | 67195.46059 | -1.0600864 | Down | 5.57E-09 |
| Anhydroicaritin 3-(6'''-Acetylgalactosyl)(1->3)-Rhamnoside-7-Glucoside | 1.487701238 | 83124.55543 | 170161.3012 | -1.033556345 | Down | 0.000332 |
| PG(18:0/6 keto-PGF1alpha) | 3.228848565 | 525008.6014 | 1041056.888 | -0.987635941 | Down | 0.019404 |
| Hydrocortamate | 1.076106091 | 47030.05687 | 89122.03842 | -0.922199154 | Down | 1.3E-05 |
| PG(20:1(11Z)/6 keto-PGF1alpha) | 3.650092672 | 731711.2224 | 1358343.333 | -0.892501888 | Down | 0.013415 |
| 4-O-Methyl-A-D-Glucosyl-(1->2)-B-D-Xylosyl-(1->4)-D-Xylose | 2.838308189 | 353211.2287 | 651489.1417 | -0.883209926 | Down | 0.000471 |
| Serylphenylalanine | 1.048630775 | 47226.51772 | 86659.61004 | -0.875762584 | Down | 2.3E-06 |
| (±)-(Z)-2-(5-Tetradecenyl)Cyclobutanone | 7.334536653 | 3159747.172 | 5742725.388 | -0.86192645 | Down | 0.01101 |
| Isosakuranin | 1.370305383 | 83383.64887 | 149069.4164 | -0.838147888 | Down | 1.9E-05 |
| PA(17:1(9Z)/19:0) | 5.171852981 | 1284398.861 | 2271317.598 | -0.822436161 | Down | 9.19E-05 |
| PA(14:0/20:0) | 3.117725959 | 465772.8653 | 822978.9668 | -0.821228965 | Down | 6.83E-05 |
| Glutamylisoleucine | 1.072967754 | 49660.29337 | 87207.99593 | -0.812367635 | Down | 2.54E-10 |
| 4-Methylene-L-Glutamine | 1.464047244 | 144866.286 | 245833.4996 | -0.762959642 | Down | 0.011678 |
| Citrulline | 1.014289398 | 71847.2581 | 121876.5873 | -0.762416003 | Down | 0.007325 |
| PS(O-18:0/17:1(9Z)) | 4.840732373 | 1370485.849 | 2311427.996 | -0.75409699 | Down | 0.000373 |
| Farnesyl Acetone | 2.633810389 | 483509.3758 | 814852.5062 | -0.75299508 | Down | 0.013414 |
| Palmitamide | 3.502427392 | 837483.3613 | 1398809.299 | -0.740066859 | Down | 0.006816 |
| N-Methyl-D-Aspartic Acid | 4.291649198 | 1006264.12 | 1675113.96 | -0.73525022 | Down | 0.000197 |
| Disodium Guanylate | 1.570863855 | 133436.8925 | 222049.4254 | -0.734723242 | Down | 1.27E-05 |
| Hirsutidin 3-Glucoside | 1.001525252 | 58104.682 | 96080.07192 | -0.725582812 | Down | 0.000196 |
| Beta-Carboline | 1.167183313 | 74023.78802 | 122069.1844 | -0.721638178 | Down | 2.3E-05 |
| Lactose-Lysine | 1.236210118 | 122830.9818 | 201000.2256 | -0.710522621 | Down | 0.01087 |
| N2-Acetylornithine | 2.601603094 | 399302.1012 | 639035.1448 | -0.678414613 | Down | 5E-05 |
| Termitomycesphin B | 4.929223077 | 1813773.85 | 2858948.761 | -0.656490179 | Down | 0.005063 |
| Dodecanamide | 1.395576051 | 147168.7649 | 230524.1477 | -0.647446376 | Down | 0.004771 |
| Trehalose 6-Phosphate | 2.584518879 | 433540.738 | 677651.8925 | -0.644376792 | Down | 2.03E-05 |
| Oxoglutaric Acid | 1.223417341 | 111985.0484 | 174759.1404 | -0.64206179 | Down | 0.000967 |
| Pelargonidin 3-(2g-Xylosylrutinoside) | 1.420012333 | 126414.9314 | 196731.132 | -0.6380584 | Down | 2.16E-05 |
| Cyaniden 3-Rutinoside-5,3'-Diglucoside | 1.283447302 | 109246.1911 | 168887.141 | -0.628476506 | Down | 2.65E-05 |
| N-Methyl-L-Glutamic Acid | 2.841087023 | 658750.3972 | 1018014.752 | -0.627954636 | Down | 0.002007 |
| Ptdins-(3,4,5)-P3 (1,2-Dioctanoyl) | 3.685178179 | 1046196.469 | 1565686.566 | -0.581641624 | Down | 0.000151 |
| Gamma-Glutamylglutamic Acid | 1.2124506 | 112400.877 | 167009.9365 | -0.571280648 | Down | 0.000489 |
| Thiobencarb | 2.374505281 | 542671.942 | 805752.3529 | -0.570256178 | Down | 0.015924 |
| N-Undecylbenzenesulfonic Acid | 1.249928624 | 194734.4452 | 288910.549 | -0.569114788 | Down | 0.04296 |
| Nad | 4.324810041 | 1518117.806 | 2248605.605 | -0.566746893 | Down | 0.000491 |
| P-Hpea-Eda | 1.589661601 | 210668.9851 | 308143.7215 | -0.548625461 | Down | 0.0003 |
| PG(17:1(9Z)/18:3(6Z,9Z,12Z)) | 4.855799583 | 1817428.611 | 2603762.516 | -0.518699173 | Down | 1.13E-07 |
| Armillarin | 2.483349516 | 783490.6054 | 1122137.612 | -0.518261729 | Down | 0.032462 |
| Thr Val Thr | 1.001665096 | 113451.609 | 161498.5801 | -0.509444411 | Down | 0.004872 |
| L-Glutamic Acid | 2.338291912 | 454470.9886 | 644418.6957 | -0.503810148 | Down | 1.01E-05 |
| Udp-Glcnac | 1.063390924 | 95396.58592 | 133339.2736 | -0.483092232 | Down | 6.52E-07 |
| Armillaripin | 3.590486553 | 1452997.423 | 2005761.839 | -0.465118168 | Down | 0.004641 |
| Beta-Nicotinamide Adenine Dinucleotide | 4.475771785 | 3049887.015 | 4204750.62 | -0.46326444 | Down | 0.029783 |
| Valylglutamic Acid | 1.212212515 | 155596.4584 | 209645.4832 | -0.430142525 | Down | 0.000112 |
| Beta-D-Galactopyranosyl-(1->4)-2-Amino-2-Deoxy-Beta-D-Glucopyranosyl-(1->6)-D-Mannose | 1.09757349 | 221047.0258 | 297703.3521 | -0.429522144 | Down | 0.025763 |
| PA(O-18:0/19:1(9Z)) | 1.032483656 | 150932.4757 | 202335.1163 | -0.422843469 | Down | 0.015778 |
| PA(18:0/17:1(9Z)) | 13.83683803 | 20406827.24 | 26875196.74 | -0.39722342 | Down | 5.92E-07 |
| Polyribosylribitolphosphate | 1.450519262 | 269044.63 | 351015.4355 | -0.383688961 | Down | 0.000882 |
| Thtc | 1.649378421 | 452240.2654 | 587013.017 | -0.376303047 | Down | 0.006718 |
| Iriediol | 4.665854829 | 3210594.631 | 4112875.232 | -0.357306785 | Down | 0.000917 |
| Citbismine C | 5.711367955 | 4540874.227 | 5809557.807 | -0.35545828 | Down | 0.000827 |
| Cimoxatone | 1.298126416 | 261194.0337 | 331313.4247 | -0.343074719 | Down | 0.001645 |
| PA(17:0/20:2(11Z,14Z)) | 4.926789707 | 3707372.291 | 4687198.936 | -0.338329031 | Down | 0.000897 |
| 3-Phenylpropionic Acid Sulfate | 1.269463896 | 252907.8463 | 319092.3771 | -0.335362348 | Down | 0.00287 |
| Harderoporphyrin | 1.931943421 | 634529.4375 | 795285.5907 | -0.325785936 | Down | 0.002658 |
| Kojibiose | 14.02510814 | 31273100.96 | 38779598.27 | -0.310375578 | Down | 0.000186 |
| 5-Hydroxy-3,3',7,8-Tetramethoxy-4',5'-Methylenedioxyflavone | 1.094637898 | 228890.2756 | 280513.6234 | -0.293414667 | Down | 0.001263 |
| All Trans Decaprenyl Diphosphate | 4.621301405 | 4316380.665 | 5252750.663 | -0.283251002 | Down | 0.001343 |
| 3-(5-Acetamido-2-Hydroxyphenyl)Sulfanyl-2-Oxopropanoic Acid | 2.729936881 | 1679951.217 | 1997829.934 | -0.250014438 | Down | 0.004396 |
| PI(20:1(11Z)/22:6(4Z,7Z,10Z,13Z,16Z,19Z)) | 1.192602804 | 317368.5742 | 375446.2381 | -0.242447057 | Down | 0.000925 |
| Disodium Phosphate | 2.117694085 | 1160026.383 | 1368615.87 | -0.238559964 | Down | 0.004827 |
| DGDG(16:0/18:3(9Z,12Z,15Z)) | 1.691908469 | 721236.3215 | 849063.0319 | -0.235399606 | Down | 0.002569 |
| Dimethyl Sulfoxide | 1.642341047 | 825078.3341 | 967304.1337 | -0.229438467 | Down | 0.010697 |
| (E)-C-Hdmapp | 1.013491276 | 238429.8741 | 279150.9077 | -0.227480238 | Down | 0.000177 |
| Benzamidine | 1.290762424 | 493190.4666 | 573117.7475 | -0.21668666 | Down | 0.007825 |
| Allantoxanamide | 4.055735888 | 5165855.956 | 5986453.989 | -0.212694275 | Down | 0.010988 |
| Camicinal | 1.189844399 | 344990.4235 | 399099.1765 | -0.210190987 | Down | 0.00021 |
| Hordatine A | 2.393861483 | 1397764.372 | 1611997.647 | -0.205728459 | Down | 0.000237 |
| Veranisatin A | 1.046272973 | 300321.3955 | 344074.9963 | -0.196215797 | Down | 0.001753 |
| Photinus Luciferin | 5.656129159 | 9439511.965 | 10776568.61 | -0.191113703 | Down | 0.001812 |
| 2-Amino-4-[(2-Amino-2-Carboxyethyl)Disulfanyl]Butanoic Acid | 2.475585901 | 1754605.397 | 1995147.054 | -0.185348474 | Down | 0.00039 |
| L-Oxalylalbizziine | 3.153546582 | 3223344.234 | 3653713.302 | -0.180805166 | Down | 0.002195 |
| S-(2-Benzothiazolyl)Cysteine | 2.555965714 | 3265403.724 | 3647457.793 | -0.159629911 | Down | 0.03421 |
| Cabotegravir | 2.94214966 | 3070654.079 | 3417928.92 | -0.154576399 | Down | 0.000646 |
| Cellotetraose | 1.872812622 | 1684404.694 | 1870594.772 | -0.15125826 | Down | 0.023713 |
| 3,4-Bis(Carboxymethyl)-3,4-Dihydroxyhexanedioic Acid | 1.026280958 | 237159.8769 | 197971.3293 | 0.260568442 | Up | 0.000114 |
| PC(18:0/P-18:0) | 1.646393187 | 761074.8337 | 620352.5259 | 0.294950031 | Up | 0.008269 |
| 5-Ethyluracil | 3.600228397 | 4043029.539 | 3240024.228 | 0.319432143 | Up | 0.042464 |
| Glucose 6-Phosphate | 1.404516338 | 391560.4951 | 313080.8444 | 0.322699977 | Up | 0.000989 |
| Hypoxanthine | 1.043925078 | 260687.664 | 205034.977 | 0.346452274 | Up | 0.021075 |
| 1,1,1-Trifluoro-2,4-Pentanedione | 2.16104518 | 842885.2136 | 662006.8275 | 0.348490078 | Up | 0.000475 |
| Lysine Phosphoester | 1.520918282 | 376848.4609 | 293612.5325 | 0.360070951 | Up | 4.58E-05 |
| D-Galactopyranosyl-(1->3)-D-Galactopyranosyl-(1->3)-L-Arabinose | 1.287219603 | 266237.2853 | 201520.4437 | 0.401786425 | Up | 0.00077 |
| N-(6-Aminohexyl)-1-Naphthalenesulfonamide | 1.473633375 | 275726.7653 | 201501.2439 | 0.452450574 | Up | 1.3E-06 |
| ((2r,3s)-3-Amino-2-Hydroxy-2-(1h-Imidazol-4-Ylmethyl)-5-Methyl)-5-Methylhexanoic Acid | 1.081781375 | 202853.4687 | 148202.6011 | 0.452867204 | Up | 0.010993 |
| Udp-D-Galactose | 1.068894834 | 173094.7994 | 125389.8033 | 0.465142347 | Up | 0.001452 |
| 5-Hydroxy-L-Tryptophan | 1.642298443 | 311337.7883 | 219862.0246 | 0.501882255 | Up | 8.51E-07 |
| Glutaric Acid | 3.272739947 | 1279696.28 | 890869.4512 | 0.522515507 | Up | 9.37E-05 |
| Alpha-Citronellol | 1.390029114 | 218443.5017 | 151212.2991 | 0.5306847 | Up | 1.66E-06 |
| 5',5''',8,8''-Tetrahydroxy-3',3''',4',4''',7',7''-Hexamethoxy-5,5''-Biflavan | 1.166836032 | 137329.9445 | 91096.56237 | 0.592177718 | Up | 6.86E-07 |
| Belinostat | 1.73764882 | 323431.9788 | 212975.4613 | 0.602775115 | Up | 7.67E-05 |
| Xanthine | 2.155079177 | 546813.9614 | 359897.7282 | 0.603463083 | Up | 0.001045 |
| Xanthone | 2.067978949 | 427780.9174 | 281532.2113 | 0.603572131 | Up | 1.79E-06 |
| Arginylglutamine | 3.154041921 | 943250.9872 | 609674.2155 | 0.629603173 | Up | 3.13E-07 |
| 2-Hydroxymyristic Acid | 3.085367432 | 913280.4713 | 589892.6487 | 0.630605554 | Up | 6.44E-07 |
| Isouramil | 3.051712628 | 949789.6146 | 612435.2556 | 0.633050646 | Up | 6.62E-05 |
| N-[4-[2-[Benzyl-[3-(3,4-Dimethoxyphenyl)Propanoyl]Amino]Ethyl]Phenyl]Quinoline-3-Carboxamide | 1.493878854 | 275881.9149 | 174917.0284 | 0.657380142 | Up | 0.003561 |
| Neridronic Acid | 1.050461194 | 99882.34717 | 62919.26357 | 0.666727939 | Up | 5.75E-07 |
| 2-Hydroxy-1,4-Benzoxazin-3-One Sulfate | 1.340279435 | 159227.7178 | 97662.48248 | 0.705215141 | Up | 8.2E-07 |
| Dacarbazine | 3.932529376 | 1418496.879 | 868825.4178 | 0.707224761 | Up | 2.85E-05 |
| DG(16:0/0:0/16:0) (d5) | 1.665606018 | 306902.9277 | 187281.1941 | 0.71257637 | Up | 0.001938 |
| 5-Ureido-4-Imidazole Carboxylate | 1.314787139 | 152988.9744 | 90645.58257 | 0.755119065 | Up | 3.38E-05 |
| Harmalol | 1.158122451 | 110561.484 | 65382.38167 | 0.75787505 | Up | 6.68E-07 |
| 6-Nitrotryptophan | 1.077076711 | 96898.77605 | 56669.23182 | 0.773912797 | Up | 1.43E-05 |
| Glutaminyl-Gamma-Glutamate | 1.989533946 | 356151.7669 | 207100.9083 | 0.782158266 | Up | 2.04E-05 |
| PG(22:4(7Z,10Z,13Z,16Z)/0:0) | 1.811793709 | 270027.5462 | 156753.3734 | 0.784610097 | Up | 2.43E-06 |
| Phosmet | 1.169741925 | 116620.3065 | 67632.25632 | 0.786035629 | Up | 1.73E-05 |
| Decarbamoylsaxitoxin | 5.319995075 | 2244835.533 | 1299922.773 | 0.788183834 | Up | 2.01E-07 |
| 9-Hydroxy Pelargonic Acid | 1.200443654 | 113610.1896 | 64378.5932 | 0.819439278 | Up | 6.64E-07 |
| N'-Hydroxyneosaxitoxin | 1.312411765 | 149172.7252 | 83551.55261 | 0.836245234 | Up | 0.00015 |
| Kinetin | 1.20541638 | 110566.6617 | 60598.38268 | 0.867565252 | Up | 8.93E-06 |
| L-2-Hydroxyglutaric Acid | 1.486105595 | 175957.6497 | 96389.39261 | 0.86828194 | Up | 5.52E-05 |
| N-(Dimethylamino)Methylene-9-((2-Hydroxy-1-(Hydroxymethyl)Ethoxy)Methyl)Guanine | 1.740492141 | 226040.3833 | 123674.9285 | 0.870027476 | Up | 1.13E-06 |
| N-Acetylkynurenine | 1.535060379 | 177136.574 | 96917.72055 | 0.870029742 | Up | 5.76E-07 |
| Glycerol Tripropanoate | 1.066999836 | 83562.82436 | 45146.19092 | 0.888256985 | Up | 2.3E-07 |
| 5,7,3',5'-Tetrahydroxy-3,6,8,4'-Tetramethoxyflavone 3'-Glucoside | 1.02148338 | 82679.85976 | 44059.9509 | 0.908068055 | Up | 0.000166 |
| N-Despropyl-Rotigotine | 2.263326689 | 365737.5492 | 194275.1655 | 0.912707261 | Up | 2.73E-07 |
| D-Sedoheptulose 7-Phosphate | 1.22174655 | 104014.0029 | 53541.22052 | 0.958055833 | Up | 1.6E-06 |
| Maltoheptaose | 3.850858594 | 990857.7467 | 493086.993 | 1.006835754 | Up | 2.51E-07 |
| 3s-Hydroxy-Dodecanoic Acid | 7.718672605 | 3835031.964 | 1856474.34 | 1.046673228 | Up | 9.93E-08 |
| Qynad | 1.07761274 | 75892.6873 | 36484.06509 | 1.056694395 | Up | 5.48E-07 |
| (S)-3-Hydroxyoctanoic Acid | 5.765452634 | 2222098.583 | 1068161.951 | 1.056792423 | Up | 7.72E-06 |
| 2-Ethyl-2-Hydroxy-Butyric Acid | 1.512257709 | 145665.0725 | 69775.48045 | 1.061862931 | Up | 8.52E-08 |
| Trichotetronine | 1.174308171 | 90686.47141 | 43304.28708 | 1.066377489 | Up | 6.86E-07 |
| Remdesivir | 1.596009159 | 162181.6295 | 76950.84955 | 1.075601254 | Up | 3.86E-07 |
| PA(14:1(9Z)/24:1(15Z)) | 2.39279694 | 641114.5257 | 303991.6496 | 1.076550402 | Up | 0.040104 |
| C-Quens | 1.197402269 | 93790.83829 | 44289.28915 | 1.082489162 | Up | 1.01E-06 |
| 5-(3-(4-(2-(4-Fluorophenyl)Ethoxy)Phenyl)Propyl)Furan-2-Carboxylic Acid | 2.812882396 | 486652.1718 | 227655.4538 | 1.096038972 | Up | 5.16E-08 |
| Modafinil | 1.380476056 | 123511.2438 | 56142.31925 | 1.137481814 | Up | 2.29E-05 |
| 10-Hydroxy Capric Acid | 10.28377308 | 6237955.425 | 2711890.735 | 1.201774191 | Up | 2.17E-07 |
| Butralin | 1.037069189 | 65285.36308 | 28374.73798 | 1.202152507 | Up | 2.15E-06 |
| PS(O-18:0/14:0) | 1.70346863 | 258501.5488 | 112044.7092 | 1.206098399 | Up | 0.01379 |
| Glycylprolylarginine | 2.162864383 | 266624.7815 | 109764.0815 | 1.280404846 | Up | 1.74E-07 |
| Aspartyllysine | 1.383845248 | 113646.0793 | 46316.148 | 1.294960735 | Up | 3.64E-06 |
| L-Valine | 1.577236921 | 148188.6567 | 60218.67333 | 1.299152189 | Up | 5.18E-05 |
| Hydroxyvalerylcarnitine | 1.673850213 | 156575.2801 | 63294.73894 | 1.306698967 | Up | 1.15E-07 |
| 1-Aminocyclohexanecarboxylic Acid | 2.042458469 | 228130.1192 | 91905.95284 | 1.311626719 | Up | 7.6E-10 |
| B-Raf Inhibitor | 2.220936237 | 271809.5016 | 108176.2855 | 1.329211624 | Up | 1.47E-07 |
| Tafluprost Free Acid | 2.277270219 | 296450.9196 | 117245.5556 | 1.338260036 | Up | 6.56E-06 |
| Riodipine | 1.038000245 | 58744.26074 | 23225.6011 | 1.338731973 | Up | 2.3E-07 |
| Artemisin | 5.393391496 | 1573804.119 | 619760.3757 | 1.344473565 | Up | 7.02E-08 |
| Metyrapone | 1.286147338 | 88567.85583 | 33894.02673 | 1.385752148 | Up | 2.39E-07 |
| 6-Oxo-Tetradecanoic Acid | 4.615706745 | 1137457.081 | 430599.3463 | 1.401394075 | Up | 4.55E-08 |
| Sarizotan | 1.732370003 | 164898.18 | 61281.24357 | 1.428057996 | Up | 1.15E-06 |
| Hydroxypropionylcarnitine | 3.271700078 | 557286.9287 | 202097.1265 | 1.463371506 | Up | 1.32E-07 |
| Ptemc | 1.005753305 | 51133.77008 | 18099.85725 | 1.49829808 | Up | 1.51E-09 |
| L-Arginine, L-Asparaginylglycyl- | 1.019258521 | 53204.60877 | 18785.89172 | 1.501901624 | Up | 5.85E-08 |
| Cyclo((2r)-2-Aminobutanoyl-N2-Methyl-L-Arginylglycyl-L-Alpha-Aspartyl-3-(Aminomethyl)Benzoyl) | 1.312578147 | 90884.94222 | 31924.76908 | 1.509365105 | Up | 1.42E-06 |
| Pinoresinol Diglucoside | 1.029144115 | 56621.40859 | 19870.9675 | 1.510685522 | Up | 8.84E-06 |
| Edulone A | 1.733311714 | 151149.58 | 52340.19442 | 1.529985781 | Up | 5.58E-09 |
| Cellulose, Microcrystalline | 1.013023002 | 55897.08903 | 19332.30767 | 1.531759293 | Up | 1.1E-05 |
| Pelargonidin 3-(2glu Glucosylrutinoside) | 2.199941262 | 240603.1415 | 82967.24908 | 1.536041622 | Up | 1.03E-08 |
| Glas#22 | 1.517303355 | 152570.4673 | 50782.22581 | 1.587080195 | Up | 0.00653 |
| Isopropyl-Beta-D-Thiogalactopyranoside | 1.44041964 | 99809.3042 | 32211.71779 | 1.631588711 | Up | 1.54E-08 |
| Methyl 6-Methyl-3-(2-Methylpropyl)-4-(3-Nitrophenyl)-4,7-Dihydrothieno[2,3-B]Pyridine-5-Carboxylate | 1.415346075 | 96839.89559 | 31114.39605 | 1.638019281 | Up | 1.37E-08 |
| Diphacinone | 2.32372644 | 254919.5221 | 79848.25874 | 1.674709009 | Up | 8.73E-10 |
| Saxitoxin | 2.586017008 | 321225.3105 | 100607.2646 | 1.67485109 | Up | 1.35E-07 |
| Vinyl-L-Nio | 4.756119376 | 1089612.945 | 339760.2018 | 1.681226973 | Up | 1.08E-07 |
| PE(18:4(6Z,9Z,12Z,15Z)/19:1(9Z)) | 6.159586508 | 2814152.185 | 869224.7175 | 1.694899244 | Up | 0.017919 |
| Urb754 | 1.451904647 | 105337.5583 | 32526.3507 | 1.69533905 | Up | 4.05E-07 |
| FAHFA(18:0/9-O-18:0) | 1.694230395 | 209105.1073 | 64480.5446 | 1.697292466 | Up | 0.015638 |
| Adipoylglycine | 1.408036915 | 91947.45647 | 27894.48633 | 1.720829682 | Up | 1.96E-12 |
| PA(8:0/18:2(10E,12Z)+=O(9)) | 1.746517137 | 144843.1575 | 43238.95377 | 1.744088011 | Up | 6.84E-09 |
| Thr Ser Val | 1.944874411 | 176404.3153 | 52648.48834 | 1.74442184 | Up | 1.79E-09 |
| Benzophenone | 1.941514144 | 175583.943 | 52383.87323 | 1.744966277 | Up | 1.14E-08 |
| Pyroglutamyl-Phenylalanyl-Prolinamide | 1.397908959 | 92729.5599 | 27142.12353 | 1.77249571 | Up | 7.6E-07 |
| N-Acetylproline | 1.255091929 | 71132.8427 | 20318.12634 | 1.807748451 | Up | 1.84E-12 |
| 3-Oxo-Dodecanoic Acid | 11.54228932 | 6157045.836 | 1728682.628 | 1.832565282 | Up | 3.7E-07 |
| PE(15:0/20:2(11Z,14Z)) | 3.76730787 | 918319.0068 | 257151.7411 | 1.836375484 | Up | 0.004596 |
| S-Lactoylglutathione | 2.029524049 | 211207.1659 | 57764.19863 | 1.87041127 | Up | 2.71E-05 |
| Pg-Pe | 2.480363205 | 276118.3697 | 75487.98568 | 1.870967916 | Up | 5.13E-10 |
| PS(O-18:0/15:0) | 1.431619395 | 136834.8688 | 37059.21605 | 1.884531643 | Up | 0.009832 |
| Somniferine | 1.386299462 | 87258.37369 | 23031.78423 | 1.921667407 | Up | 7.39E-07 |
| Resmethrin | 1.217166948 | 66218.54573 | 17437.74724 | 1.925021655 | Up | 1.27E-08 |
| 11-Oxahexadecanolide | 2.792039372 | 338380.1526 | 86780.38535 | 1.963204053 | Up | 2.02E-11 |
| PG(17:2(9Z,12Z)/0:0) | 1.189990092 | 64376.61366 | 16236.12891 | 1.987328991 | Up | 3.88E-07 |
| 1-(4-Hydroxy-3-Methoxyphenyl)-3-Decanone | 1.198102112 | 61794.69104 | 15214.97694 | 2.02199075 | Up | 2.74E-10 |
| 3,4-Dimethyl-5-Carboxyethyl-2-Furanpentanoic Acid | 1.116568967 | 53121.72874 | 12541.8031 | 2.082557322 | Up | 1.37E-09 |
| (Gamma-Glutamyl-Gamma-Glutamyl)-S-Methylcysteine | 1.353774014 | 76218.14279 | 16436.40504 | 2.213239666 | Up | 1.52E-10 |
| Diethofencarb | 1.409335007 | 82559.75589 | 17653.91189 | 2.225450803 | Up | 9.52E-09 |
| Cyclo(Arg-Gly-Asp-D-Phe-Val) | 1.767440211 | 127394.862 | 26229.9218 | 2.280021682 | Up | 3.04E-10 |
| Casopitant | 1.037303042 | 44097.88538 | 8577.471749 | 2.362085102 | Up | 9.35E-09 |
| PE(16:0/18:1(11Z)) | 18.9267912 | 17549826.04 | 3077294.204 | 2.511722447 | Up | 0.000663 |
| Asn-Arg-Ala-Ile | 3.110928983 | 383163.6667 | 60440.32231 | 2.664377506 | Up | 5.69E-07 |
| 4,4-Difluorovitamin D3 / 4,4-Difluorocholecalciferol | 1.648823148 | 103260.8761 | 15001.65277 | 2.783100383 | Up | 7.1E-10 |
| Glu Thr Thr | 1.003378488 | 39325.38799 | 5226.841628 | 2.911449648 | Up | 2.88E-08 |
| Sodium Stearoyl 2-Lactylate | 4.800572638 | 888934.3317 | 112816.3358 | 2.978100862 | Up | 1.22E-06 |
| Cohibin C | 2.006025191 | 188313.8394 | 22448.80066 | 3.068428754 | Up | 0.001129 |
| Lauroyl Peroxide | 1.236842659 | 56049.48768 | 5978.528425 | 3.228838863 | Up | 5.95E-09 |
| Thr Glu Thr | 1.459371629 | 80182.51572 | 8378.321724 | 3.258554493 | Up | 3.7E-08 |
| 2-Decylfuran | 1.05089714 | 37779.87555 | 2372.767716 | 3.99297517 | Up | 4.41E-13 |
| Tuftsin | 1.292506514 | 58639.60533 | 3538.559305 | 4.05064139 | Up | 7.89E-08 |
| Quilostigmine | 1.317778998 | 63205.33309 | 3469.867828 | 4.187093679 | Up | 6.47E-06 |
| N(G)-Nitroarginine-4-Nitroanilide | 1.097551765 | 42525.1709 | 2331.45825 | 4.189012533 | Up | 3.34E-08 |
| Sacubitril | 1.162051176 | 48135.21349 | 2412.709882 | 4.318366336 | Up | 1.76E-06 |
| PA(15:1(9Z)/22:6(4Z,7Z,10Z,13Z,16Z,19Z)) | 1.299915372 | 60698.94165 | 2794.495227 | 4.441011746 | Up | 4.49E-06 |
| Glyceraldehyde 3-Phosphate | 2.520137139 | 230221.5727 | 7623.804613 | 4.91636807 | Up | 9.99E-07 |
| PE(18:1(11Z)/24:1(15Z)) | 1.112254058 | 46084.65619 | 984.5616616 | 5.548661115 | Up | 5.13E-05 |
| Tamsulosin | 1.090682817 | 41103.61317 | 810.974273 | 5.66346526 | Up | 6.22E-07 |
| N-Nitro-N-Phenylnitramide | 1.232350363 | 72695.38558 | 602.2945772 | 6.915250711 | Up | 0.004645 |
| 6-Benzyl-1-(Benzyloxymethyl)-5-Iodouracil | 1.255922062 | 59344.72543 | 0.006053133 | 23.22493453 | Up | 3.23E-05 |

The differential metabolites were screened by VIP value >1 and p-value <0.05

Metabolites were decreased and increased in Δ*mupR* compare with WT*,* colored in Blue and rose-carmine, respectively.
